# Floridoside Phosphotriester Derivatives: Synthesis and Inhibition of Human Neutrophils’ Oxidative Burst

**DOI:** 10.3390/molecules30132850

**Published:** 2025-07-03

**Authors:** Luís Pinheiro, Catarina Cipriano, Filipe Santos, Patrícia Máximo, Eduarda Fernandes, Marisa Freitas, Paula S. Branco

**Affiliations:** 1 LAQV, REQUIMTE, Department of Chemistry, NOVA School of Science and Technology, Universidade Nova de Lisboa, Campus da Caparica, 2825-149 Caparica, Portugal; l.pinheiro@campus.fct.unl.pt (L.P.); ci.cipriano@campus.fct.unl.pt (C.C.);; 2 LAQV, REQUIMTE, Laboratory of Applied Chemistry, Department of Chemical Sciences, Faculty of Pharmacy, University of Porto, 4050-313 Porto, Portugal; egracas@ff.up.pt

**Keywords:** floridoside, phosphotriesters, phosphoglycolipid, oxidative burst, antioxidant, anti-inflammatory, glycosylation, phosphorylation

## Abstract

Floridoside (2-*O*-D-glycerol-*α*-D-galactopyranoside) is a natural product typically found in red algae. It serves as the algae’s carbon reserve and is produced as a protective response against osmotic and heat stress. Both floridoside and its acylated derivatives have been associated with modulating redox homeostasis and inflammatory responses. Therefore, we aimed to evaluate whether the newly synthesized floridoside phosphotriesters (**1b**–**1d**, **1f**–**1h**) and acylated floridoside derivative (**1e**) can modulate the oxidative burst in stimulated human neutrophils. Synthetic strategies included the glycosylation of the thioglycoside donor with glycerol derivatives, having NIS/TfOH as the promoter. Phosphorylation was achieved with POCl_3_ in the presence of pyridine. The compounds were analysed for their cytotoxicity, with **1b** and **1h** being cytotoxic at 50 μM, while the others showed no cytotoxicity in the tested concentrations. The detection of the neutrophils’ oxidative burst was performed using multiple probes [luminol, aminophenyl fluorescein (APF), and Amplex Red (AR)] to evaluate reactive species levels. Compound **1e** prevented the oxidative burst in activated human neutrophils (IC_50_ = 83 ± 7 μM). All the other tested compounds were ineffective in inhibiting APF and AR oxidation under the present experimental conditions. These findings highlight the potential of floridoside-based derivatives as candidates for targeting inflammatory pathways.

## 1. Introduction

**Figure 1:**
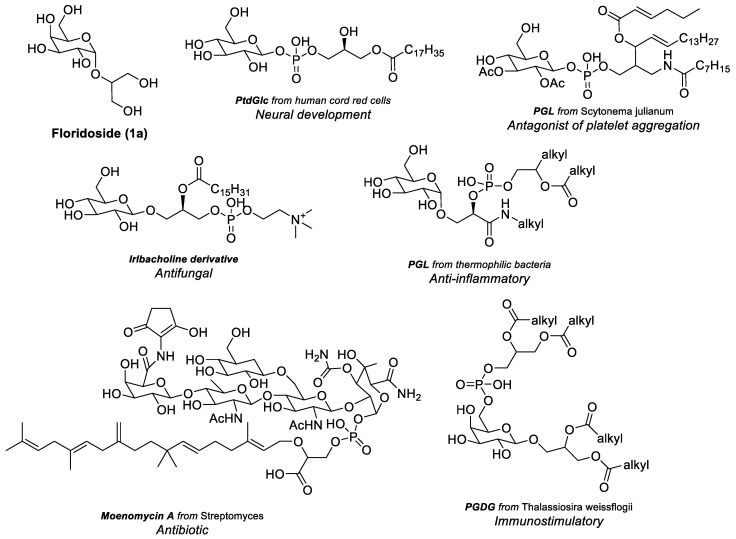
Structure of floridoside (**1a**) and selected examples of GPL and PGL compounds with different bioactivities.

Glycophospholipids (GPL) and phosphoglycolipids (PGL) are relatively underexplored subclasses of glycolipids [1]. They are distinguished by the presence of a phosphate group within the glycolipid molecule. Our recent review on this field [2] covers the existent reports on the synthesis and bioactivity of GPL and PGL (some examples in Figure 1). Their activities range from neurogenic [3,4,5], agonists and antagonists of platelet aggregation [6], antifungal [7], anti-inflammatory [8], antibiotic [9], and immunostimulatory [10]. These compounds can exhibit substantial structural diversity, depending on the position of the phosphate group and the specific combination of lipid and glycosyl moieties. Nevertheless, all reported structures share a common phosphodiester functionality, prompting interest in the potential biological effects of phosphotriesters structurally analogous to GPL and PGL. Considering the structure and biosynthesis, GPL and PGL appear to derive from a glycosyl or glycoglycerol core that undergoes phosphorylation via a phospholipid donor [11]. The galactoglycerol core, albeit rarer than the glucoglycerol in GPL and PGL according to our research [2], was identified in a natural PGL from the microalga *Thalassiosira weissflogii* and described as immunostimulatory (Figure 1) [10]. Another galactoglycerol core with interesting immunomodulating properties is floridoside.

Floridoside (**1a**) (2-*O*-D-glycerol-*α*-D-galactopyranoside) (Figure 1) is a natural product typically found in rhodophyta [12,13,14] (red algae) and chrysophyta (golden algae) [15], acting as a carbon reserve [12] and playing a role in osmotic balance since its production increases when the algae are exposed to high levels of salinity [16,17,18]. Its production appears also to be a defence mechanism against heat shock-induced stress responses, namely increased NADPH oxidase activity and hydrogen peroxide (H_2_O_2_) in *Pyropia haitanensis*, a red alga [19]. Floridoside (**1a**) is known to suppress the pro-inflammatory responses in lipopolysaccharide (LPS)-stimulated BV-2 microglia cells by inhibiting the production of nitric oxide and reactive oxygen species (ROS). The protein and gene expression levels of inducible nitric oxide synthase (iNOS) and cyclooxygenase-2 were also observed to be down-regulated [20]. In L-02 hepatocytes, hemoxygenase-1 (HO-1) expression was up-regulated, and the activity of superoxide dismutase (SOD) and glutathione peroxidase (GSH-Px) was increased. Ochsenkühn et al. attribute a dual role to floridoside in *Symbiodinium* dinoflagellates in that it serves as both an adaptive response to osmotic stress and a protector against reactive species resulting from osmotic and heat stress [21]. The most comprehensive study on floridoside’s antioxidant activity was conducted by Li and co-workers [22]. They demonstrated that floridoside (from *Laelia undulata*) exhibits considerable scavenging activity against generated free radicals [22]. Moreover, in RAW264.7 cells, floridoside displayed ROS scavenging activity, though less pronounced than in free radical assays [22]. Additionally, it was identified as a potent inhibitor of myeloperoxidase (MPO) in HL-60 cells [22], an enzyme responsible for the production of hypochlorous acid and other hypohalous acids from H_2_O_2_, which are the main microbicidal and host tissue harmers in oxidative burst [23,24]. Floridoside also up-regulated GSH band SOD in RAW264.7 cells, enhancing cellular antioxidant defence. Furthermore, it significantly downregulated matrix metalloproteinase-2 (MMP-2) expression in HT-1080 cells [22]. MMP-2 is an enzyme involved in extracellular matrix degradation and tissue remodelling [25].

Neutrophils are the central cells of acute inflammation [26]. Accordingly, in the event of an inflammatory process, an increase is observed in the number, lifespan, mobility, tissue influx ability, and phagocytic capacity of neutrophils [27]. One of the most important mechanisms used by neutrophils to protect the organism against the invaders is the production of an array of ROS and reactive nitrogen species (RNS) [28]. However, the sustained overproduction of reactive species or the impairment of antioxidant defences may result in a prooxidant status of the cells known as oxidative stress, leading to detrimental effects to the host, namely, alterations in the normal function of lipids, proteins, or DNA [29]. Hence, there is a need to moderate the level of oxidative stress generated with anti-inflammatory drugs [30].

Current anti-inflammatory drugs are often limited by undesirable side effects, which compromise their long-term use and efficacy. Therefore, there is ongoing interest in the development of new therapeutic agents that retain or improve anti-inflammatory efficacy while minimizing adverse effects. In this context, novel strategies targeting neutrophils’ activity, namely, the production of reactive species, will be useful for the treatment of both acute and chronic inflammatory conditions. Considering the known properties of floridoside **1a** and phosphorylated glycolipids, we focused on the synthesis and bioactivity of floridoside phosphotriesters. In this study, we present our synthetic approach to achieve floridoside (**1a**), acylated floridoside (**1e**), and its phosphotriester derivatives (**1b**–**d**, **1f**–**h**). Their structures are outlined in Table 1. Additionally, we evaluated the ability of compounds **1a**–**h** to modulate neutrophil-mediated inflammatory responses. To the best of our knowledge, this is the first report describing both the synthesis of floridoside-based phosphotriesters and their modulatory effects on human neutrophils’ oxidative burst. With this study, we attempt to extend the knowledge on floridoside and its analogues and their capacity for modulating physiological responses, as well as point in a new direction towards developing anti-inflammatory agents.

## 2. Results and Discussion

### 2.1. Synthesis of Floridoside Phosphotriesters ***1a**–**h***

The synthesis of PGL molecules presents a significant challenge due to the chemo- and stereoselectivity complexities inherent in sugar chemistry. Numerous studies in the literature have addressed this issue by developing methodologies to circumvent the similar reactivity of hydroxyl groups and control anomeric selectivity [31]. To tackle the first challenge, the widely accepted approach involves generating a sugar derivative with a suitable leaving group at the anomeric position, which reacts under specific conditions while all other hydroxyl groups are protected to prevent side reactions [32,33]. However, achieving anomeric stereoselectivity is more complex, as multiple mechanisms influence the stereoselective outcome of a glycosylation reaction, including (i) neighbouring group participation [34], (ii) steric hindrance from protective groups in the glycosyl donor and/or acceptor [35], (iii) metal coordination and catalysis [36], and (iv) enzymatic procedures [37]. The key challenge lies in designing a synthetic strategy that integrates the optimal conditions to ensure an efficient and highly stereoselective glycosylation reaction.

The general synthetic strategy and the compounds synthesized (**1a**–**h**) are outlined in Table 1. Benzylated thiogalactose **2** was selected as the glycosyl donor [32] to be coupled with the appropriate protected glycerol derivative **3**, originating the galactoglycerol **4**. The selective removal of the protective groups can be followed either by phosphorylation with POCl_3_ and nucleophilic attack with an alcohol to give the desired phosphotriester **5** or **5’** or by acylation. Full deprotection of these compounds gave compounds **1b**–**h**. Full deprotection of **4** gives floridoside **1a**.

The abundance of hydroxyl groups necessitated the use of orthogonal protecting groups throughout the synthesis. Benzyl (Bn) was chosen as the final group due to its stability during the synthesis and its ease of removal. Allyl, acetyl (Ac), and *t-*butyldiphenylsilyl (TBDPS) were chosen as preferred glycerol-protective groups. For glycosylation, the thioglycoside method was chosen over the halide method [33] and the imidate method [38] due to the stability of compound **2**. The use of ester-protecting groups at this stage was avoided to prevent anomeric selectivity issues caused by neighbouring group participation, which would undesirably yield 1,2-trans glycosides [34]. For phosphorylation, the most common methods in GPL and PGL synthesis are the *H-*phosphonate method and POCl_3_ method, as reviewed by us [2]. We chose the POCl_3_ method for its simplicity.

**Scheme 1:**
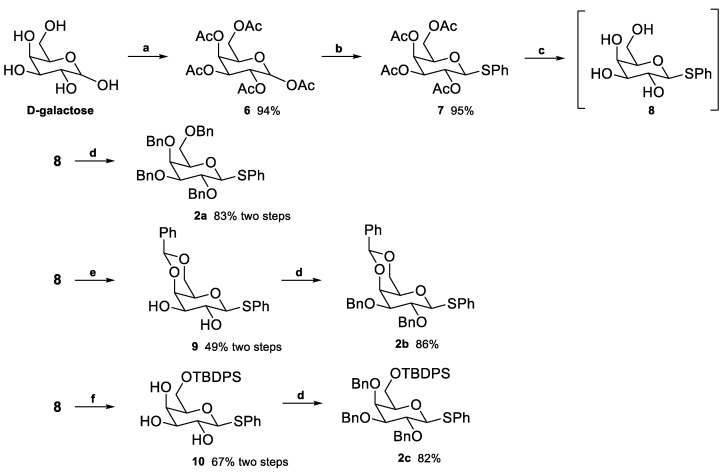
Synthesis of glycosyl donors **2a**–**c**. (a). Ac_2_O, NaOAc, reflux; (b) PhSH, BF_3_·Et_2_O, dry DCM, 0 °C → rt; (c) (i) NaOMe (cat.), MeOH, rt, **2h**; (ii) HCl 1M; (d) (i) NaH, dry DMF, 0 °C, N_2_; (ii) BnBr, 0 °C → rt; (e) benzaldehyde dimethyl ketal, TsOH, reflux, dry DMF; and (f) imidazole, TBDPSCl, dry DMF, 0 °C → rt.

The glycosyl donors **2a**, **2b**, and **2c** were synthesized as outlined in Scheme 1. D-Galactose was *per*-acetylated [39], yielding compound **6**, obtained in 94% yield (*α*/*β* 1:3) after purification. The two anomers are easily distinguished by ^1^H NMR since the anomeric proton (H-1) of the *α*-anomer typically appears downfield compared to the *β*-anomer. Moreover, the coupling constants of the corresponding doublets are substantially different, with the H-1*α* being ≈ 3 Hz and the H-1*β* being ≈ 7 Hz due to the dihedral angles. Compound **6** underwent BF_3_·Et_2_O-promoted thioglycosylation [40] with thiophenol, affording thiogalactose **7** in 95% yield. Subsequent deacetylation with catalytic NaOMe in MeOH yielded intermediate **8**. The yields at this stage were not determined, and **8** was directly used in the following reactions, with the reported yields reflecting two-step processes. Compound **2a** was obtained in 83% yield via Williamson etherification [41,42], using NaH as the base and benzyl bromide as the alkylating agent. Compound **2b** was synthesized by first protecting the 4- and 6-hydroxyl groups as a benzylidene acetal (**9**, 49% yield) [43], followed by benzylation to obtain **2b** in 86% yield. Compound **2c** was prepared by selectively protecting the 6-hydroxyl with the bulky TBDPS group (**10**, 67%) [44], which preferentially reacts with primary hydroxyls. Subsequent benzylation of the secondary hydroxyls afforded **2c** in 82% yield.

**Scheme 2:**
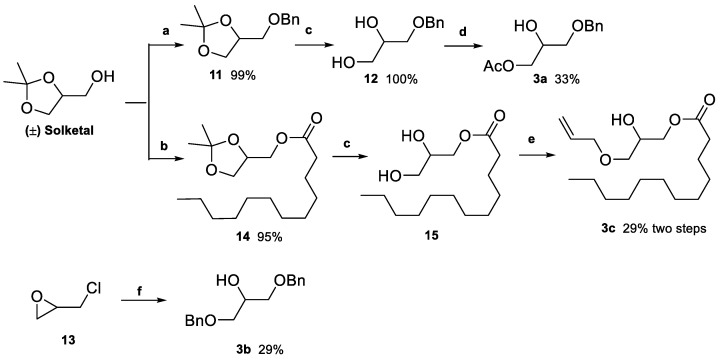
Synthesis of glycosyl donors **3a**–**c**. (a) (i) NaH, dry DMF, 0 °C, N_2_; (ii) BnBr, 0 °C → rt; (b) dodecanoic acid, DCC, DMAP, dry DCM, N_2_; (c) AcOH/H_2_O 4:1, rt; (d) (i) 3Å molecular sieves, toluene, reflux, 15 min; (ii) DBTO, reflux, **2h**; (iii) AcCl, dry DMF, rt.; (e) (i) 3Å molecular sieves, toluene, reflux, 15 min; (ii) DBTO, reflux, **2h**; (iii) AllylBr, dry DMF, rt. and (f) BnOH, NaOH, 40 °C.

The glycosyl acceptors **3a**, **3b**, and **3c** were synthesized as outlined in Scheme 2. For **3a**, (±)-solketal was first benzylated (**11**, 99% yield), followed by acetal hydrolysis with aqueous acetic acid, affording **12** in quantitative yield. The challenging regioselective acetylation of the primary position was solved using Aragozzini’s method [45], which involved the formation of a dioxastanollane intermediate with dibutyl tin oxide (DBTO), followed by ring-cleaving with acetyl chloride [46], yielding **3a** in 33% yield. 

Acylation of (±)-solketal with dodecanoic acid was achieved in the presence of the coupling reagent *N,N’*-dicylohexylcarbodiimide (DCC) and a catalytic amount of 4-dimethylaminopyridine (DMAP) [47], yielding **14**. Subsequent deprotection of the acetal afforded compound **15**. The same DBTO-assisted method [46] was then used to selectively introduce an allyl group at the terminal hydroxyl, affording **3c** in 29% yield.

Glycerol **3b** was prepared from epichlorohydrin (**13**) in 28% yield by reaction with benzyl alkoxide formed in situ with NaOH [48]. 

**Scheme 3:**
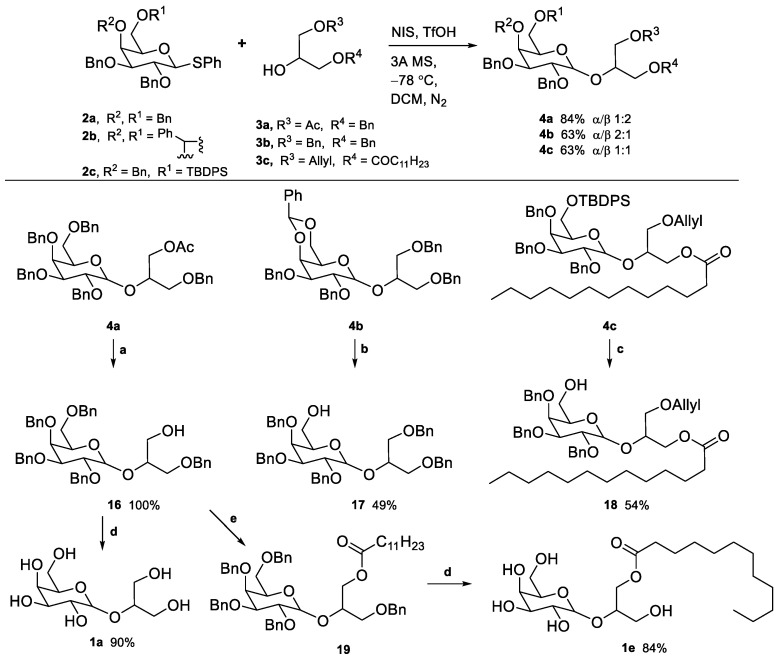
Glycosylation reaction to obtain **4a**–**c**, subsequent deprotection steps to obtain **16**–**18**, and synthesis of compounds **1a** and **1e**. (a) NaOMe, MeOH, rt; (b) (i) BH_3_·THF (1M), Cu(OTf)_2_; (ii) H_2_O; (c) TBAF 1M in THF, rt. (d) H_2_ 3 bar, 10% Pd/C, EtOH, rt, **3h**; and (e) DCC, DMAP, dodecanoic acid, DCM, N_2_, rt.

Galactoglycerols **4a**–**c** were synthesized using the iodonium promotion strategy [49] with *N*-iodosuccinimide (NIS) and a catalytic amount of trifluoromethanesulphonic acid (Scheme 3) [50]. Compound **2a** reacted with glycosyl acceptor **3a**, yielding **4a** in 84% yield (*α*/*β* 1:2). Both anomers could be separated chromatographically, although we proceeded with the mixture in the following reactions. Compound **2b** reacted with acceptor **3b** to yield **4b** in 63% yield (*α*/*β* 2:1), and compound **2c** reacted with acceptor **3c** to give **4c** in 63% yield (*α*/*β* 1:1). Next, selective deprotection was performed (Scheme 3). The acetyl group in **4a** was removed using NaOMe, yielding **16** in 100% yield. The benzylidene acetal in **4b** was converted into a benzyl group at position 4, leaving the 6-*O*-hydroxyl free via regioselective ring-opening reduction. Among several reported methods for this conversion involving diverse hydride sources and Lewis acid pairs, LiAlH_3_/AlCl_3_ [51], DIBAL-H [52], Me_2_NH·BH_3_/BF_3_·OEt_2_ [53], BH_3_·THF/V(O)(OTf)_2_ [54], and BH_3_·THF/Cu(OTf)_2_ [55], the last method was employed, yielding **17** in 49% yield. The TBDPS group in **4c** was removed using tetrabutylammonium fluoride (TBAF) in THF, affording **18** in 54% yield.

Compound **1a** was obtained from the hydrogenation of **16** over Pd/C in 90% yield (Scheme 3). This compound was obtained as a mixture of anomers rather than exclusively the natural product floridoside (*α*-anomer). To obtain compound **1e**, **16** was acylated with dodecanoic acid in the presence of DCC and DMAP, yielding **19** in 85% yield. Subsequent hydrogenation of **19** gave **1e** in 84% yield (Scheme 3). These non-phosphorylated compounds, **1a** and **1e**, were intended as controls in the biological assays, allowing the evaluation of the absence/presence of the phosphate group in activity.

**Scheme 4:**
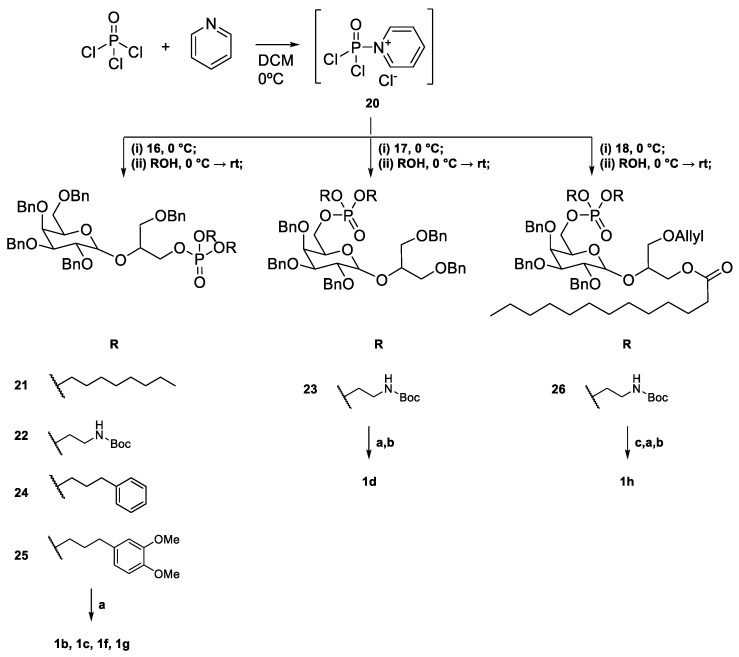
Phosphorylation reaction, synthesis of phosphorylated compounds **21**–**26** and deprotected final compounds **1b**–**d** and **1f**–**h**. (a) H_2_ 3 bar, 10% Pd/C, EtOH, rt, 3h; (b) TFA/DCM 1:1, rt; and (c) (i) cat. PdCl_2_, DCM/MeOH 1:1; (ii) Et_3_N.

Phosphate ester synthesis is well-established, particularly in the synthesis of nucleotides [56] and phospholipids [57]. Common phosphorylation techniques typically use trivalent (phosphites [58] and phosphoramidites [59,60]) or pentavalent (phosphoric acids [61,62]) phosphate sources. The former requires an oxidation step to convert them into pentavalent phosphate. The latter can be optimized with several strategies to enhance reactivity, like activation with trifluoromethanesulphonic anhydride [63] and trichloroacetonitrile [64] to generate good leaving groups or the use of coupling reagents like DCC to facilitate ester formation [61]. The phosphorylation of **16**–**18** was carried out using a modified [62,65] one-pot procedure where POCl_3_ first reacted with pyridine to generate the phosphoryl pyridinium intermediate **20** [63]. This intermediate was then sequentially reacted with **16**–**18**, followed by an excess amount of a chosen alcohol (Scheme 4). This approach yielded compounds **21**–**26**. However, this procedure proved suboptimal, as the products were obtained in low yields. Additionally, the excess of POCl_3_ and alcohol was detrimental to the purification process, with the trialkyl phosphate side product often co-eluting with the desired product. Four different alcohols were used in the phosphorylation process. *n*-Octanol yielded compound **21,** which, after deprotection, gave **1b**. *N*-*t*-Butyloxycarbonyl (Boc) ethanolamine led to the synthesis of compounds **22, 23**, and **26**, which, after deprotection, yielded **1c**, **1d**, and **1h**, respectively. 3-Phenylpropan-1-ol resulted in compound **24**, which, after deprotection, formed **1f**. 3’,4’-Dimethoxy-3-phenylpropan-1-ol gave compound **25**, which, after deprotection, produced **1g**. As for the deprotection step, benzyl groups were removed by hydrogenation and catalysed by palladium on activated charcoal under a 3 bar H_2_ atmosphere. The Boc group was removed using 50% trifluoroacetic acid (TFA) in DCM [66], and the allyl group was removed with a catalytic amount of PdCl_2_ [67].

### 2.2. Evaluation of Cell Death in Neutrophils

**Figure 2:**
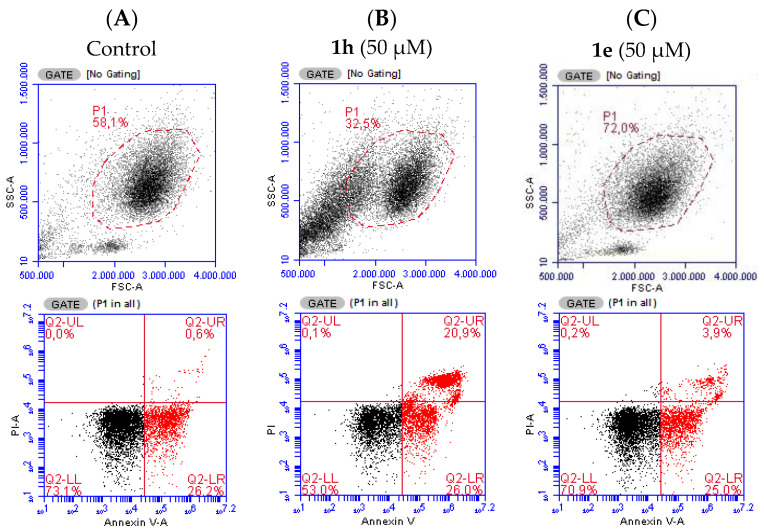
Representative flow cytometry plots of Annexin V [(Annexin V; x-axis)/PI (y-axis)] binding assay of control (**A**) and compounds **1h** (**B**) and **1e** (**C**) at 50 μM FSC—forward scatter area; SSC—side scatter area.

**Figure 3:**
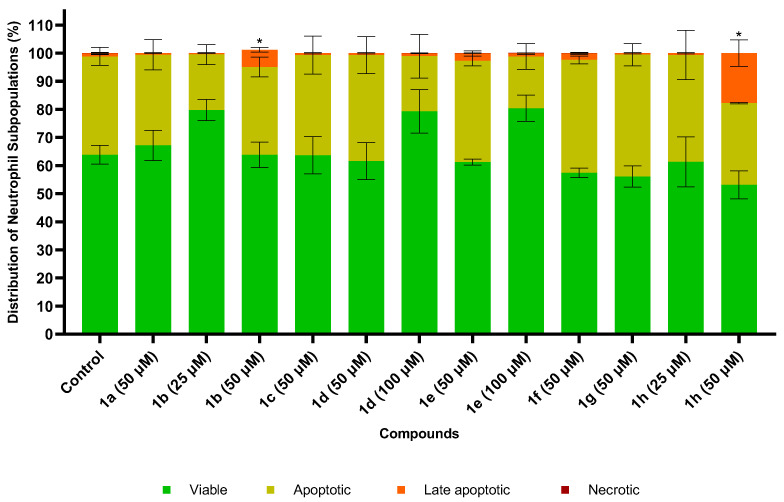
Effect of compounds **1a**–**h** on neutrophils’ viability measured by flow cytometry. Results are expressed as the percentages (%) of viable (AV^−^/PI^−^), apoptotic cells (AV^+^/PI^−^), late apoptotic cells (AV^+^/PI^+^), and necrotic cells (AV^−^/PI^+^) and presented as mean± SEM (*n* ≥ 3). * *p* < 0.05 when compared with control (untreated cells).

The effects of the compounds listed in Table 1 on neutrophil cell death were evaluated by analysing the percentage of neutrophil population defined by forward scatter (FSC) versus side scatter (SSC) parameters and the distribution among viable (AV^−^/PI^−^), apoptotic (AV^+^/PI^−^), late apoptotic (AV^+^/PI^+^), and necrotic (AV^−^/PI^+^) cells relative to the control (untreated cells). All the compounds were initially tested at 50 μM concentration, among which compounds **1b** and **1h** were found to be cytotoxic as evidenced by a reduction in the neutrophil population, measured by FSC versus SSC, and an increase in the number of cells in the late apoptotic state (Figure 2B) compared to the control (Figure 2A). Considering this result, the concentration of **1b** and **1h** was reduced to 25 μM, where they did not exhibit significant cytotoxicity. All other compounds showed no statistically relevant cytotoxicity up to 100 μM. For example, the flow cytometry plots for the non-cytotoxic compound **1e** at a concentration of 100 μM are presented in Figure 2C. The cytotoxicity reports regarding all compounds are shown in Figure 3.

Compounds **1b** and **1h** share structural similarity, as both feature long alkyl chains attached to the glycerol backbone. Interestingly, both compounds were found to induce late apoptosis in neutrophils (Figure S154). Given that compound **1e** also contains a myristate ester similar to **1h** but lacks the phosphotriester function, it would be expected to induce a comparable effect if this was only due to the low polarity substituent. However, further studies with a larger library of compounds are needed to determine whether this effect is attributed to the presence of a phosphate moiety and to evaluate the impact of chain length on cytotoxicity. There are just a few reports concerning the cytotoxicity of compounds structurally related to those synthesized in this study. Larsen et al. [68] tested a compound similar to **1e**, albeit with different acyl chains, in polymorphonuclear leukocytes and reported a cell viability close to 100% at 100 *μ*g/mL, assessed by the trypan blue exclusion method. Colombo et al. [69,70] tested floridoside monohexanoates, similar to **1e**, on Raji cells and reported no relevant cytotoxicity even at the highest tested concentrations (32 nM), also assessed by the trypan blue exclusion method. As corroborated by previous studies in MRC-5, RAW264.7, HL-60, and HT-1080 cells [22], compound **1a** displayed no cytotoxic activity.

### 2.3. Modulation of Neutrophils’ Oxidative Burst

The modulation of PMA-induced neutrophils’ oxidative burst by compounds **1a**–**h** was evaluated by the chemiluminescent probe luminol. The results are shown in Table 2. Among the tested compounds, only compound **1e** showed statistically relevant activity by inhibiting the oxidative burst, IC_50_ = 83 ± 7 μM (Figure S154). All the other compounds did not produce any significant effect. The positive controls quercetin and DPI presented an IC_50_ of 1.2 ± 0.2 μM and 0.08 ± 0.01 μM, respectively.

The generation of HOCl by neutrophils stimulated by PMA was assessed by the fluorescent probe aminophenyl fluorescein (APF). Treatment with compounds **1a**–**h** resulted in non-significant inhibition of HOCl production (Table 2). The best result obtained was 29 ± 10% inhibition for compound **1b** at 25 μM. Compound **1a** also gave a similar result with 27 ± 6% inhibition (50 μM). The IC_50_ obtained for the positive controls was 2.4 ± 0.2 μM for quercetin and 0.030 ± 0.003 μM for DPI.

The generation of H_2_O_2_ by PMA-stimulated neutrophils was evaluated by the fluorescent probe Amplex Red. Treatment with compounds **1a**–**h** did not result in significant inhibition of H_2_O_2_ production (Table 2). The IC_50_ of the positive controls was 4.1 ± 0.3 μM for quercetin and 0.060 ± 0.004 μM for DPI.

This is the first study to report on the effects of compounds **1a**–**h** on the oxidative burst in human neutrophils. Moreover, there are few reports of similar compounds regarding their effects on the production of reactive species. Compounds structurally similar to **1e**, as a monogalactosylmonoacylglycerol, are quite common, especially in lipidic extracts of natural sources [71]. Galactolipids (digalactosyldiacylglycerols, DGDG) from *Potentilla anserina* L. present comparable IC_50_ values (4.91 μM to 29.23 μM) for the inhibition of ^•^NO in RAW 264.7 macrophages, which are comparable to that of the control indomethacin (10.07 μM) [72,73]. The authors also concluded that the saturation of the fatty acid chains was shown to influence the activity, with increased unsaturation leading to reduced inhibition of ^•^NO production [73]. Banskota et al. [74,75] produced similar findings regarding monogalactosyldiacylglycerols (MGDG) and DGDG containing eicosapentaenoic alkyl chains. The compounds isolated from *Nannochloropsis granulate* [74] and *Porphyridium aerugineum* [75] showed mild inhibition of LPS-induced ^•^NO production in RAW 264.7 macrophages with potency comparable to that of N^G^-monomethyl-L-arginine acetate, a known ^•^NO inhibitor. Their study indicates that the additional galactose unit in DGDG did not significantly influence the observed effects. Similar results with RAW 264.7 cells were obtained by Liu et al. [76], who reported a reduction in ^•^NO production and iNOS gene expression in response to galactoglycerolipids isolated from *Perilla frutescens* (L.) Britton.

Matsufuji et al. [77] reported that a branched-chain MGDG, designated M874B, was capable of preventing the Fenton reaction by inhibiting Fe^2+^ oxidation and scavenging hydroxyl and alkoxyl radicals but not superoxide radical anion. Additionally, the compound protected *Bacilus subtilis* PS2248, a catalase-deficient strain, from oxidative damage induced by exogenous H_2_O_2_ [77].

## 3. Materials and Methods

### 3.1. General Information for Synthetic Procedures

All yields presented are isolated yields. When possible, the proportions of the anomers were determined by integrating the H-1 signals in the ^1^H NMR spectra. When only one of the H-1 peaks could be clearly identified, complementary signals from other protons were used to support the estimation of the anomeric ratio. The presence of the phosphate group was confirmed by ^31^P NMR. NMR signal attribution is given whenever it is possible to determine. In certain cases, only one of the anomers’ signals is attributed due to spectra complexity, even in cases where an anomeric mixture was obtained, which is mentioned.

All reagents used in synthesis were purchased from commercial suppliers and used without further purification, unless specified. Solvent drying was performed by using 3Å molecular sieves pre-activated in 5 × 2 min on −0.5 min off cycles under microwave heating and then placed in a vacuum [78]. Thin-layer chromatography analysis was performed on Merck (Darmstadt, Germany) Kieselgel GF 254 0.2 mm plates supported on aluminium and revealed under UV light (254 nm) and by staining with the appropriate staining spray (MeOH/H_2_SO_4_ 1:1 for carbohydrate compounds, ninhydrin for amine products, and phosphomolybdic acid solution in remaining cases) [79]. Normal phase column chromatography was performed on the bench with Kieselgel 60A from Carlo Erba (Cornaredo, Italy), 40–63 μm granulometry (“flash” chromatography), or with the Pure Essential Chromatography System (Pure Chromatography C-900, Pure Fraction Collector C-106, Pure UV Detector C-107) from Buchi (Flawil, Switzerland). Reverse-phase column chromatography was performed with LiChroprep RP-18 (Merck), 40–63 μm granulometry, or CHROMABOND^®^ C18 Hydra SPE polypropylene column cartridges (Carl Roth GmbH, Karlsruhe, Germany).

NMR spectra were acquired with Bruker ARX 400 or Bruker (Billerica, MA, USA) Avance III spectrometers. ^1^H NMR, ^13^C NMR, and ^31^P NMR spectra were acquired at 400, 101, and 162 MHz, respectively. Data were treated with MestreNova 14.2 software. Chemical shifts (*δ*) are reported in parts per million (ppm). Coupling constants (*J*) are reported in Hz. The following NMR abbreviations are used: s = singlet, d = doublet, t = triplet, q = quartet, quint = quintuplet, m = multiplet, dd = doublet of doublets, and dt = doublet of triplets. NMR spectra were calibrated according to the mentioned solvent in each spectrum.

Mass spectra (LCMS) were recorded at the Laboratório de Análises, Requimte, Faculdade de Ciências e Tecnologia, Universidade Nova de Lisboa, using an LC Agilent (Santa Clara, CA, USA) 1200 Series with a Binary pump/MS Agilent 6130B Single Quadrupole with an ESI source. High-resolution mass spectra (HRMS) were obtained at the University of Salamanca (Salamanca, Spain) through the Elemental Analysis, Chromatography, and Mass Spectrometry Service (NUCLEUS), using a Thermo (Waltham, MA, USA) QExactive focus spectrometer at a resolution of 30,000. For the ionization, ESI in alternating positive and negative mode was used (3.5 KV for positive mode and 3.0 KV for negative mode). Infusion was performed through a Thermo vanquish HPLC with a void union as a column with a flow of methanol of 0.2 mL/min.

### 3.2. Synthetic Procedures

General procedure for the synthesis of 4. In a Schlenk flask, dried overnight at 100 °C and under an N_2_ atmosphere with previously activated 3Å MS, were charged dry DCM, the galactose derivative (0.025 M), and the glycerol derivative (1.05 eq). The mixture was stirred for 30 min. at rt, then cooled to −78 °C, and stirred for another 10 min; after which NIS (1.5 eq) and TfOH (0.1 eq) were added sequentially. The reaction was left to slowly reach rt. After full consumption of the galactose derivative, Et_3_N was added dropwise to quench (until the reaction mixture turned orange). The sieves were filtered off, and 10% aqueous Na_2_S_2_O_3_ was added. The layers were separated, and the organic phase was washed three times with water and then brine. The mixture was purified by “flash” chromatography and eluted with an appropriate mixture of PE/EtOAc.

*1-O-Acetyl-3-O-benzyl-2-O-(2′,3′,4′,6′-tetra-O-benzyl-β-D-galactopyranosyl)-sn-glycerol* (**4a**) Eluted with PE/EtOAc 7:3. Obtained as a yellow-orange viscous oil in 84% yield (*α*/*β* 1:2). *α*-anomer (2 diastereomers): ^1^H NMR (400 MHz, CDCl_3_) δ 7.42–7.21 (m, 25H), 5.16–5.12 (m, 1H, H-1), 4.97–4.91 (m, 1H, OCH_2_Ar), 4.86–4.64 (m, 4H, OCH_2_Ar), 4.59–4.54 (m, 1H, OCH_2_Ar), 4.53–4.51 (m, 1H, OCH_2_Ar), 4.47–4.45 (m, 1H, OCH_2_Ar), 4.44–4.35 (m, 2H, OCH_2_Ar), 4.34–4.27 (m, 1H, H-7a), 4.21–4.00 (m, 4H, H-2, H-4, H-5, H-7b), 3.98–3.93 (m, 2H, H-3, H-8), 3.66–3.46 (m, 4H, H-6, H-9), 1.99–1.92 (m, 3H, OAc). ^13^C NMR (101 MHz, CDCl_3_) δ 171.04, 170.98, 139.09, 138.91, 138.84, 138.78, 138.28, 138.11, 128.62, 128.59, 128.56, 128.53, 128.51, 128.48, 128.44, 128.41, 128.08, 128.06, 127.96, 127.94, 127.91, 127.87, 127.83, 127.79, 127.75, 127.66, 127.64, 127.60, 97.37 (C-1), 97.27 (C-1), 79.13, 77.56, 77.44, 77.24, 76.92, 76.61, 76.38, 75.27, 75.10, 75.01, 74.97, 74.19, 74.03, 73.74, 73.65, 73.62, 73.39, 73.32, 73.26, 73.19, 70.02, 69.83, 69.71, 69.66, 69.24, 68.90, 64.84, 64.32, 60.61, 21.02, 20.92. *β*-anomer (2 diastereomers): ^1^H NMR (400 MHz, CDCl_3_) δ 7.38–7.22 (m, 25H, ArH), 4.96–4.90 (m, 2H, OCH_2_Ar), 4.78–4.68 (m, 3H, OCH_2_Ar), 4.61 (d, *J* = 11.6 Hz, 1H, OCH_2_Ar), 4.51 (s, 2H, OCH_2_Ar), 4.49 (d, *J* = 7.8 Hz, 1H, H-1), 4.44–4.39 (m, 2H, OCH_2_Ar), 4.35 (dd, *J* = 11.7, 3.4 Hz, 1H, H-7a), 4.22 (dd, *J* = 11.8, 6.3 Hz, 1H, H-7b), 4.10–4.04 (m, 1H, H-8), 3.90–3.87 (m, 1H, H-4), 3.80 (dd, *J* = 9.6, 7.8 Hz, 1H, H-2), 3.74 (dd, *J* = 9.9, 4.4 Hz, 1H, H-9a), 3.60 (dd, *J* = 7.6, 2.4 Hz, 1H, H-9b), 3.58–3.48 (m, 4H, H-6, H-5, H-3, 1.88 (s, 3H, OAc). ^13^C NMR (101 MHz, CDCl_3_) δ 138.85, 138.62, 138.49, 138.14, 137.91, 128.45, 128.41, 128.37, 128.34, 128.27, 128.24, 128.21, 128.07, 127.97, 127.91, 127.85, 127.82, 127.70, 127.67, 127.60, 127.56, 127.46, 103.99, 82.19, 79.38, 77.37, 77.25, 77.05, 76.73, 76.46, 75.00, 74.57, 73.54, 73.45, 73.33, 73.14, 73.09, 69.68, 68.88, 64.39, 50.84. HRMS-ESI: Calculated for [C_46_H_50_O_9_+Na^+^] 769.3347; found 769.3340.

*1,3-di-O-Benzyl-2-O-(2′,3′-di-O-benzyl-4′,6′-di-O-benzylidene-α-D-galactopyranosyl)-sn-glycerol* (**4b**) Eluted with PE/EtOAc 7:3. Both anomers were obtained as yellowish oils in 63% combined yield (*α*/*β* 2:1) *α*-anomer: ^1^H NMR (400 MHz, CDCl_3_) δ 7.52–7.48 (m, 2H, ArH), 7.43–7.39 (m, 2H, ArH), 7.39–7.22 (m, 21H, ArH), 5.40 (s, 1H, H-7), 5.25 (d, *J* = 3.4 Hz, 1H, H-1), 4.84–4.63 (m, 4H, CH_2_Ar), 4.55–4.41 (m, 4H, CH_2_Ar), 4.15–4.09 (m, 2H, H-3, H-5), 4.06–3.98 (m, 3H, H-2, H-4, H-8/10), 3.92–3.89 (m, 1H, H-9), 3.68 (dd, *J* = 12.5, 1.9 Hz, 1H, H-8/10), 3.65–3.62 (m, 2H, H-6), 3.59 (m, 2H, H-8/10). ^13^C NMR (101 MHz, CDCl3) δ 139.14, 138.88, 138.31, 138.23, 138.09, 128.91, 128.53, 128.51, 128.42, 128.40, 128.36, 128.25, 128.20, 127.93, 127.83, 127.76, 127.70, 127.65, 127.63, 127.60, 126.48, 101.13 (C-7), 97.27 (C-1), 76.21 (C-4), 75.59 (C-2), 74.90 (C-3), 74.79 (C-5), 73.54, 73.29, 73.26, 72.22, 71.01 (C-6), 70.08 (C-9), 69.52 (C-8), 62.60 (C-10). HRMS-ESI: Calculated for [C_44_H_46_O_8_+Na^+^]: 727.3085; found: 727.3094.

*3-(Allyloxy)-2-(((3R,4S,5S,6R)-3,4,5-tris(benzyloxy)-6-(((tert-butyldiphenylsilyl)oxy)methyl)tetrahydro-2H-pyran-2-yl)oxy)propyl dodecanoate* (**4c**) Eluted with a mixture of PE/EtOAc 9.5:0.5. The product was obtained as a colourless oil in 63% yield (*α*/*β* 1:1). Since the two anomers were unseparated, the ^1^H NMR nuclide count is doubled. ^1^H NMR (400 MHz, CDCl_3_) δ 7.73–7.56 (m, 8H, ArH), 7.50–7.18 (m, 42H, ArH), 5.93–5.73 (m, 2H, H-15), 5.30–5.06 (m, 5H, H-1*α*, H-16), 5.02–4.56 (m, 12H, CH_2_Ar), 4.53–4.43 (m, 1H, H-1*β*), 4.38–4.32 (m, 1H, H-7), 4.28–4.12 (m, 3H, H-7), 4.10–3.87 (m, 10H, H-2*α*, H-4, H-5*α*, H-8, H-14), 3.84–3.63 (m, 6H, H-2*β*, H-6, H-9_1H_), 3.61–3.47 (m, 5H, H-3, H-9_3H_), 3.43–3.37 (m, 1H, H-5*β*), 2.28–2.14 (m, 4H, H-10), 1.75–1.51 (m, 4H, H-11), 1.41–1.19 (m, 32H, H-12), 1.13–1.01 (m, 18H, C(CH_3_)_3_), 0.95–0.85 (m, 6H, H-13). ^13^C NMR (101 MHz, CDCl_3_) δ 173.75, 173.58, 173.49, 173.43, 139.01, 138.95, 138.82, 138.78, 138.74, 138.71, 138.69, 138.64, 135.64, 135.60, 135.57, 135.55, 135.33, 134.82, 134.58, 134.53, 133.37, 133.32, 133.27, 129.82, 129.78, 129.76, 129.74, 128.37, 128.35, 128.32, 128.28, 128.21, 128.16, 128.14, 128.11, 128.08, 128.01, 127.96, 127.94, 127.86, 127.78, 127.74, 127.68, 127.60, 127.57, 127.54, 127.47, 127.44, 127.40, 127.35, 117.04, 116.99, 116.91, 109.22, 103.98, 103.43, 96.87, 96.77, 82.19, 82.14, 79.50, 79.46, 78.91, 77.37, 77.25, 77.05, 76.73, 76.47, 76.38, 76.32, 76.10, 75.72, 75.23, 74.99, 74.93, 74.88, 74.83, 74.66, 73.89, 73.85, 73.53, 73.27, 73.24, 73.20, 73.13, 72.92, 72.81, 72.32, 72.24, 72.21, 71.22, 71.09, 69.75, 69.63, 69.54, 66.85, 64.59, 64.05, 63.91, 62.59, 62.41, 62.32, 34.19, 34.12, 31.93, 29.73, 29.64, 29.52, 29.49, 29.36, 29.29, 29.17. HRMS-ESI: Calculated for [C_61_H_80_O_9_Si+Na^+^] 1007.5459; found 1007.5446.

*3-O-benzyl-2-O-(2′,3′,4′,6′-tetra-O-benzyl-β-D-galactopyranosyl)-sn-glycerol* (**16**) The starting material **4a** was dissolved in MeOH, and 0.5 eq of NaOMe was added. The reaction proceeded at rt. After full consumption of the starting material, a few drops of HCl 1 M were added to neutralize the reaction. The solvent was evaporated, and the crude dried in a vacuum. The mixture was purified by “flash” chromatography and eluted with petroleum ether/EtOAc 6:4. *α*-anomer: ^1^H NMR (400 MHz, CDCl_3_) δ 7.41–7.24 (m, 25H, ArH), 5.01 (d, *J* = 3.7 Hz, 1H, H-1), 4.93 (d, *J* = 11.4 Hz, 1H, CH_2_Ar), 4.87 (d, *J* = 11.5 Hz, 1H, CH_2_Ar), 4.79–4.75 (m, 2H, CH_2_Ar), 4.70 (d, *J* = 11.5 Hz, 1H, CH_2_Ar), 4.57 (d, *J* = 11.5 Hz, 1H, CH_2_Ar), 4.50–4.42 (m, 2H, CH_2_Ar), 4.42–4.32 (m, 2H, CH_2_Ar), 4.15–4.10 (m, 1H, H-5), 4.10–4.04 (m, 1H, H-2), 4.01–3.95 (m, 2H, H-3, H-4), 3.89–3.81 (m, 1H, H-8), 3.75–3.66 (m, 1H, H-6a), 3.65–3.42 (m, 5H, H-6b, H-7, H-9). NMR data are consistent with the literature [80]. *β*-anomer: ^1^H NMR (400 MHz, CDCl_3_) δ 7.45–7.20 (m, 25H, ArH), 4.94 (d, *J* = 11.4 Hz, 1H, CH_2_Ar), 4.90–4.81 (m, 1H, CH_2_Ar), 4.79–4.67 (m, 3H, CH_2_Ar), 4.64–4.37 (m, 6H, CH_2_Ar, H-1), 3.97–3.81 (m, 4H), 3.81–3.42 (m, 7H).

*((2R,3S,4S,5R)-3,4,5-Tris(benzyloxy)-6-((1,3-bis(benzyloxy)propan-2-yl)oxy)tetrahydro-2H-pyran-2-yl)methanol* (**17**) In a dried round-bottom flask under N_2_ atmosphere, **4b** in THF was added, followed by a catalytic amount (0.15 eq) of Cu(OTf)_2_. Next, 3 eq of BH_3_.SMe_2_ (2M) or BH_3_·THF (1M) were quickly added. After completion, water was added dropwise to quench. After the gas evolution stopped, EtOAc was added, and the organic phase was washed with sat. NaHCO_3_ and brine and dried over Na_2_SO_4_. The mixture was purified by “flash” chromatography and eluted with a mixture of PE/EtOAc 75:25. The product was obtained in 49% yield as a colourless oil (*α/β* 1:1). *α*-anomer: ^1^H NMR (400 MHz, CDCl_3_) δ 7.43–7.22 (m, 25H, ArH), 5.24 (d, *J* = 3.8 Hz, 1H, H-1), 4.95 (d, *J* = 11.6 Hz, 1H, CH_2_Ar), 4.89 (d, *J* = 11.6 Hz, 1H, CH_2_Ar), 4.74 (d, *J* = 11.6 Hz, 1H, CH_2_Ar), 4.72–4.69 (m, 2H, CH_2_Ar), 4.62 (d, *J* = 11.6 Hz, 1H, CH_2_Ar), 4.54–4.44 (m, 4H, CH_2_Ar), 4.17–4.10 (m, 1H, H-5), 4.08–3.99 (m, 2H, H-2, H-8), 3.93 (dd, *J* = 10.1, 2.9 Hz, 1H, H-3), 3.86–3.83 (m, 1H, H-4), 3.63–3.55 (m, 5H, H-6, H-7, H-9a), 3.43–3.31 (m, 1H, H-9b). ^13^C NMR (101 MHz, CDCl_3_) δ 138.91, 138.55, 138.30, 138.08, 138.04, 128.50, 128.45, 128.42, 128.40, 128.27, 127.87, 127.80, 127.65, 127.55, 127.49, 96.69 (C-1), 79.07 (C-3), 77.25 (C-2), 76.33 (C-5), 75.38 (C-4), 74.64, 74.46, 73.45, 73.42, 73.28, 72.77, 70.73 (C-6), 70.37, 70.30, 62.64, 62.48. *β*-anomer: ^1^H NMR (400 MHz, CDCl_3_) δ 7.39–7.23 (m, 25H, ArH), 5.03–4.91 (m, 2H, CH_2_Ar), 4.81 (d, *J* = 11.8 Hz, 1H, CH_2_Ar), 4.77–4.71 (m, 2H, CH_2_Ar), 4.66 (d, *J* = 11.9 Hz, 1H, CH_2_Ar), 4.58 (d, *J* = 7.7 Hz, 1H, H-1), 4.54 (d, *J* = 2.6 Hz, 2H, CH_2_Ar), 4.51–4.46 (m, 2H, CH_2_Ar), 4.07 (p, *J* = 5.2 Hz, 1H, H-8), 3.84 (dd, *J* = 9.7, 7.6 Hz, 1H, H-3), 3.76–3.68 (m, 4H, H-4, H-5, H-7a, H-9a), 3.67–3.60 (m, 2H, H-7b, H-9b), 3.51 (dd, *J* = 9.7, 3.0 Hz, 1H, H-2), 3.47–3.38 (m, 1H, H-6a), 3.35–3.30 (m, 1H, H-6b). ^13^C NMR (101 MHz, CDCl_3_) δ 138.92, 138.49, 138.30, 138.24, 128.66, 128.45, 128.44, 128.33, 128.21, 128.07, 127.96, 127.67, 127.63, 127.57, 127.53, 127.41, 103.72 (C-1), 82.32, 79.68, 77.85, 77.23, 75.03, 74.66, 74.13, 73.50, 73.37, 73.31, 73.01, 70.45. HRMS-ESI: Calculated for [C_44_H_48_O_8_+Na^+^]: 727.3241; found: 727.3232. NMR data are consistent with the literature [81].

*3-(Allyloxy)-2-(((3R,4S,5S,6R)-3,4,5-tris(benzyloxy)-6-(hydroxymethyl)tetrahydro-2H-pyran-2-yl)oxy)propyl dodecanoate* (**18**) To starting material **4c** (500 mg, 0.51 mmol) were added 3 equivalents of tetrabutylammonium fluoride (1 M in THF). After 2h and completion of the reaction, the volatiles were evaporated, and the crude mixture was purified by “flash” chromatography and eluted with PE/EtOAc 7:3. The product was obtained as colourless oil in 54% yield (*α*/*β* 1:1). *α*-anomer: ^1^H NMR (400 MHz, CDCl_3_) δ 7.43–7.24 (m, 15H, ArH), 5.85 (ddt, *J* = 17.3, 10.3, 5.6 Hz, 1H, H-15), 5.24 (dq, *J* = 17.3, 1.7 Hz, 1H, H-16a), 5.19–5.13 (m, 2H, H-1, H-16b), 4.97 (d, *J* = 11.6 Hz, 1H, CH_2_Ar), 4.88 (d, *J* = 11.7 Hz, 1H, CH_2_Ar), 4.80–4.75 (m, 2H, CH_2_Ar), 4.72 (d, *J* = 11.8 Hz, 1H, CH_2_Ar), 4.64 (d, *J* = 11.7 Hz, 1H, CH_2_Ar), 4.25 (dd, *J* = 11.7, 4.8 Hz, 1H, H-7a), 4.15 (dd, *J* = 11.7, 6.0 Hz, 1H, H-7b), 4.10–3.90 (m, 7H, H-2, H-3, H-4, H-5, H-8, H-14), 3.70 (dd, *J* = 11.3, 6.5 Hz, 1H, H-6a), 3.60–3.52 (m, 2H, H-9), 3.52–3.46 (m, 1H, H-6b), 2.24 (dd, *J* = 8.1, 7.1 Hz, 2H, H-10), 1.60–1.54 (m, 2H, H-11), 1.25 (d, *J* = 5.3 Hz, 16H, H-12), 0.88 (t, *J* = 6.8 Hz, 3H, H-13). ^13^C NMR (101 MHz, CDCl_3_) δ 173.64, 138.89, 138.58, 138.34, 134.53 (C-15), 128.69, 128.61, 128.55, 128.51, 128.10, 127.93, 127.81, 127.71, 127.63, 117.50 (C-16), 96.93 (C-1), 79.19, 77.36, 76.92, 76.54, 75.23, 74.55, 74.06, 73.61, 73.26, 72.38 (C-14), 70.73, 70.32, 63.85, 62.79, 34.29, 32.04, 29.74, 29.60, 29.47, 29.41, 29.39, 29.29, 24.99, 22.82, 14.25. *β*-anomer: ^1^H NMR (400 MHz, CDCl_3_) δ 7.40–7.27 (m, 15H), 5.93–5.77 (m, 1H), 5.29–5.12 (m, 2H), 4.98–4.89 (m, 2H), 4.83–4.69 (m, 3H), 4.65 (dd, *J* = 11.8, 7.9 Hz, 1H), 4.51 (dd, *J* = 7.7, 2.6 Hz, 1H), 4.34 (ddd, *J* = 26.5, 11.6, 4.1 Hz, 1H), 4.22 (dt, *J* = 12.0, 6.1 Hz, 1H), 4.09–3.95 (m, 3H), 3.85–3.67 (m, 4H), 3.60–3.43 (m, 3H), 3.42–3.34 (m, 1H), 2.34–2.13 (m, 2H), 1.65–1.47 (m, 2H), 1.34–1.18 (m, 16H), 0.88 (t, *J* = 6.7 Hz, 3H). ^13^C NMR (101 MHz, CDCl_3_) δ 174.06, 173.74, 138.84, 138.77, 138.44, 138.39, 138.24, 138.21, 134.52, 128.69, 128.56, 128.48, 128.44, 128.41, 128.26, 128.09, 128.02, 127.92, 127.71, 127.65, 127.52, 127.50, 117.25, 117.15, 104.19, 103.90, 82.30, 82.26, 79.54, 79.47, 77.36, 77.24, 77.04, 76.78, 76.72, 75.49, 75.07, 75.03, 74.78, 74.21, 74.18, 73.52, 73.47, 73.31, 72.90, 72.40, 72.37, 70.02, 69.98, 64.10, 63.79, 62.48, 61.99, 34.32, 34.10, 31.92, 29.63, 29.48, 29.35, 29.28, 29.15, 29.13, 24.88, 24.84, 22.70, 14.14. HRMS-ESI: Calculated for [C_45_H_62_O_9_+Na^+^] 769.4292; found 769.4270.

*3-(benzyloxy)-2-(((3R,4S,5S,6R)-3,4,5-tris(benzyloxy)-6-((benzyloxy)methyl)tetrahydro-2H-pyran-2-yl)oxy)propyl dodecanoate* (**19**) The starting galactoglycerol **16** (62 mg, 0.088 mmol) was dissolved in dry DCM (0.25 M) under an N_2_ atmosphere. Dicyclohexyl carbodiimide (20 mg, 0.097 mmol), DMAP (2.2 mg, 0.018 mmol), and dodecanoic acid (19.4 mg, 0.097 mmol) were sequentially added. The reaction proceeded at room temperature. After completion of the reaction, DCM was added, and the precipitate was filtered. The filtrate was washed with water (3 × 10 mL) and brine (10 mL). The organic phase was dried over Na_2_SO_4_. The mixture was purified by “flash” chromatography and eluted with PE/EtOAc 7:3. The product was obtained as a white waxy solid in 85% yield (*α*/*β* 1:2). *α*-anomer: ^1^H NMR (400 MHz, CDCl_3_) δ 7.41–7.21 (m, 25H, ArH), 5.18–5.13 (m, 1H, H-1), 4.97–4.91 (m, 1H, CH_2_Ar), 4.87–4.81 (m, 1H, CH_2_Ar), 4.77–4.66 (m, 3H, CH_2_Ar), 4.65–4.44 (m, 3H, CH_2_Ar), 4.44–4.20 (m, 3H, CH_2_Ar, H-7a), 4.18–4.00 (m, 3H, H2, H-5, H-7b), 3.98–3.92 (m, 1H, H-3), 3.90–3.86 (m, 1H, H-8), 3.65–3.45 (m, 5H, H-4, H-6, H-9), 2.39–2.10 (m, 2H, H-10), 1.65–1.44 (m, 2H, H-11), 1.35–1-17 (m, 16H, H-12), 0.88 (t, *J* = 6.8 Hz, 3H, H-13). *β*-anomer: ^1^H NMR (400 MHz, CDCl_3_) δ 7.41–7.21 (m, 25H, ArH), 4.97–4.91 (m, 1H, CH_2_Ar), 4.77–4.66 (m, 4H, CH_2_Ar), 4.65–4.44 (m, 4H, CH_2_Ar, H-1), 4.44–4.20 (m, 4H, CH_2_Ar, H-7a), 4.18–4.00 (m, 1H, H-7b, H-8), 3.98–3.92 (m, 1H, H-4), 3.90–3.86 (m, 1H, H-3), 3.84–3.71 8m, 2H, H-2, H-9a) 3.65–3.45 (m, 4H, H-5, H-6, H-9b), 2.39–2.10 (m, 2H, H-10), 1.65–1.44 (m, 2H, H-11), 1.35–1-17 (m, 16H, H-12), 0.88 (t, *J* = 6.8 Hz, 3H, H-13). *αβ*-anomers: ^13^C NMR (101 MHz, CDCl_3_) δ 173.86, 173.75, 139.03, 138.85, 138.72, 138.30, 138.22, 138.13, 138.05, 128.57, 128.54, 128.48, 128.45, 128.43, 128.39, 128.33, 128.20, 128.06, 128.00, 127.97, 127.93, 127.91, 127.88, 127.86, 127.84, 127.80, 127.76, 127.74, 127.70, 127.67, 127.65, 127.56, 127.51, 104.12, 97.21, 97.15, 82.33, 79.51, 79.07, 76.32, 76.16, 75.20, 75.09, 74.95, 74.68, 74.09, 73.88, 73.66, 73.63, 73.58, 73.53, 73.28, 73.05, 70.02, 69.85, 69.55, 69.13, 68.99, 68.86, 64.47, 60.54, 34.28, 34.21, 34.16, 32.05, 29.76, 29.64, 29.61, 29.50, 29.48, 29.44, 29.41, 29.30, 29.26, 25.05, 24.95, 22.83, 14.34, 14.26. HRMS-ESI: Calculated for [C_56_H_70_O_9_+Na^+^]: 909.4912; found 909.4899.

The general procedure for the synthesis of phosphoesters **21**–**26** POCl_3_ (5 eq) was added to a 0 °C dry DCM solution of dry pyridine (10 eq) 0.25 M under an N_2_ atmosphere. After the fumes subsided, the clear solution obtained was added to a 0 °C dry DCM solution of dry pyridine (10 eq) and galactoglycerol (100 mg, 0.25 M) under an N_2_ atmosphere. After full consumption of the galactoglycerol, the appropriate alcohol (20 eq) was added, and the reaction stayed overnight. The mixture was diluted with DCM and washed with 0.1 M HCl three times. The organic phase was then washed with brine and dried over Na_2_SO_4_. Purification was performed/achieved by “flash” chromatography and eluted with an appropriate mixture of PE/EtOAc.

*3-O-Benzyl-2-O-(2′,3′,4′,6′-tetra-O-benzyl-D-galactopyranosyl)propyl dioctyl phosphate* (**21**): Starting with **16**, the alcohol used was *n*-octanol. The crude mixture was eluted with PE/EtOAc 8:2. The product was obtained as colourless oil in 52% yield (*α*/*β* 1:1). ***α*-anomer:** ^1^H NMR (400 MHz, CDCl_3_) δ 7.39–7.19 (m, 25H, ArH), 5.17 (d, *J* = 3.8 Hz, 1H, H-1), 4.92 (d, *J* = 11.4 Hz, 1H, CH_2_Ar), 4.82 (d, *J* = 11.7 Hz, 1H, CH_2_Ar), 4.73–4.63 (m, 3H, OCH_2_Ar), 4.57–4.48 (m, 4H, CH_2_Ar), 4.41 (d, *J* = 11.7 Hz, 1H, CH_2_Ar), 4.23–4.11 (m, 3H, H-6, H-8), 4.11–3.95 (m, 8H, H-2, H-3, H-4, H-5, H-10), 3.66–3.60 (m, 2H, H-9), 3.55 (dd, *J* = 6.7, 1.6 Hz, 2H, H-7), 1.63–1.55 (m, 4H, H-11), 1.30–1.19 (m, 20H, H-12), 0.87 (m, 6H, H-13). ^13^C NMR (101 MHz, CDCl_3_) δ 138.73–137.95, 128.39–127.36, 97.08 (C-1), 78.91 (C-2), 76.24 (C-3), 75.03 (C-4), 74.81, 74.47 (C-5), 73.47, 73.40 (CH_2_, CH_2_Ar), 73.11, 72.86, 69.42 (C-8), 69.22 (C-9), 68.74 (C-7), 67.91 (C-10), 67.10 (C-6), 31.79 (C-10), 30.35 (C-11), 30.28 (C-11), 29.20–22.64 (C-12), 14.09 (C-13). LCMS: 1009.5 [M+H]^+^; 1026.5 [M+NH_4_]^+^.

*Di-tert-butyl (2-(((3-(benzyloxy)-2-(((3R,4S,5S,6R)-3,4,5-tris(benzyloxy)-6-((benzyloxy)methyl)tetrahydro-2H-pyran-2-yl)oxy)propoxy)(2-(methylamino)ethoxy)phosphoryl)bis(oxy)bis(ethane-2,1-diyl)dicarbamate* (**22**) Starting with **16,** the alcohol used was *N*-Boc ethanolamine. The crude mixture was eluted with PE/EtOAc 6:4. The product was confirmed to be present by ^1^H, albeit heavily contaminated with *tris*(*N*-Boc ethanolamine) phosphate, which co-elutes with the intended product. The mixture was used in the next reaction without further purification. LCMS: 1088.5 [M+NH_4_]^+^; 1093.4 [M+Na]^+^.

*Di-tert-butyl (2-(((2R,3S,4S,5R)-3,4,5-tris(benzyloxy)-6-((1,3-bis(benzyloxy)propan-2-yl)oxy)tetrahydro-2H-pyran-2-yl)methoxy)phosphoryl) bis(oxy)bis(ethane-2,1-diyl)dicarbamate* (**23**) Starting with **17,** the alcohol used was *N*-Boc ethanolamine. The mixture was purified by flash chromatography and eluted with PE/EtOAc 6:4. The product was confirmed to be present by ^1^H, albeit heavily contaminated with *N*-Boc ethanolamine, which co-elutes with the intended product. The mixture was used in the next reaction without further purification.

*3-(Benzyloxy)-2-(((3R,4S,5S,6R)-3,4,5-tris(benzyloxy)-6-((benzyloxy)methyl)tetrahydro-2H-pyran-2-yl)oxy)propyl bis(3-phenylpropyl) phosphate* (**24**) Starting with **16,** the alcohol used was 3-phenylpropan-1-ol. The crude mixture was eluted with PE/EtOAc 7:3. The product was obtained as a colourless viscous oil (*α*/*β* 1:10). *β*-anomer: ^1^H NMR (400 MHz, CDCl_3_) δ 7.40–7.05 (m, 35H, ArH), 5.15 (d, *J* = 3.7 Hz, 1H, H-1*α*), 5.00–4.89 (m, 2H, CH_2_Ar), 4.76–4.65 (m, 3H, CH_2_Ar), 4.64–4.44 (m, 4H, CH_2_Ar, H-1*β*), 4.43–4.31 (m, 2H, H-7), 4.23–3.93 (m, 7H, H-8, H-9, H-10), 3.92–3.85 (m, 1H, H-4), 3.84–3.72 (m, 2H, H-2, H-6a), 3.71–3.42 (m, 5H, H-3, H-5, H-6b, H-9), 2.77–2.54 (m, 4H, H-12), 2.08–1.81 (m, 4H, H-11). ^13^C NMR (101 MHz, CDCl_3_) δ 141.04, 140.99, 140.97, 140.91, 140.71, 138.87, 138.82, 138.71, 138.65, 138.59, 138.53, 138.49, 138.16, 138.08, 137.99, 137.93, 137.90, 137.88, 128.54, 128.49, 128.46, 128.38, 128.35, 128.29, 128.22, 128.17, 128.07, 127.91, 127.88, 127.85, 127.82, 127.78, 127.70, 127.65, 127.63, 127.60, 127.56, 127.45, 127.40, 126.17, 126.12, 126.08, 126.04, 125.89, 103.94, 103.54, 97.55 (C-1*α*), 82.17, 82.13, 79.46, 79.42, 78.87, 76.51, 76.43, 76.37, 75.11, 75.06, 74.80, 74.68, 74.60, 73.57, 73.52, 73.48, 73.43, 73.38, 73.33, 73.23, 73.16, 73.10, 72.89, 69.58, 69.37, 69.05, 68.81, 68.59, 68.46, 68.40, 67.13, 67.08, 67.03, 66.90, 66.84, 34.26, 32.11, 31.88, 31.81, 31.73, 31.68, 31.64, 31.62, 31.51, 29.73. HRMS-ESI: Calculated for [C_62_H_69_O_11_P+Na^+^] 1043.4470; found 1043.4459.

*3-(Benzyloxy)-2-(((3R,4S,5S,6R)-3,4,5-tris(benzyloxy)-6-((benzyloxy)methyl)tetrahydro-2H-pyran-2-yl)oxy)propyl bis(3-(3,4-dimethoxyphenyl)propyl) phosphate* (**25**) The alcohol used was 3-(3,4-dimethoxyphenyl)propan-1-ol. The crude mixture was eluted with PE/EtOAc 7:3. The product was obtained as colourless viscous oil (*α*/*β* 1:1), contaminated with the tri-substituted phosphate of the alcohol used. ^1^H NMR (400 MHz, CDCl_3_) δ 7.42–7.17 (m, 50H, ArH), 6.84–6.62 (m, 12H, H-13, H-14, H-15), 5.20–5.12 (m, 1H, H-1*α*), 4.97–4.87 (m, 3H, CH_2_Ar), 4.82–4.64 (m, 7H), 4.61–4.29 (m, 11H, H-1*β*), 4.25–3.99 (m, 24H, H-2*α*, H-10), 3.96–3.72 (m, 40H, H-2*β*, -OMe), 3.71–3.42 (m, 12H), 2.79–2.54 (m, 8H, H-12), 2.10–1.85 (m, 8H, H-11). The nuclide count is doubled to account for both anomers. ^13^C NMR (101 MHz, CDCl_3_) δ 170.95, 148.73, 148.69, 147.20, 147.15, 140.70, 138.63, 138.45, 138.38, 138.25, 137.94, 137.81, 137.68, 133.30, 133.28, 133.06, 128.29, 128.23, 128.21, 128.15, 128.13, 128.10, 128.05, 128.03, 128.00, 127.96, 127.83, 127.66, 127.62, 127.60, 127.54, 127.48, 127.45, 127.41, 127.38, 127.35, 127.32, 127.24, 127.16, 127.13, 125.91, 120.07, 120.04, 111.58, 111.54, 111.51, 111.10, 111.08, 97.38, 96.94, 81.98, 79.20, 78.67, 77.55, 76.10, 74.86, 74.77, 74.58, 74.37, 73.28, 73.21, 73.01, 72.92, 72.83, 72.65, 69.38, 68.82, 68.22, 66.86, 66.75, 66.69, 60.19, 55.72, 55.70, 55.64, 55.62, 31.95, 31.88, 31.75, 31.69, 31.47, 31.05, 31.02, 30.90, 20.85, 14.00. HRMS-ESI: Calculated for [C_66_H_77_O_15_P+Na^+^] 1163.4892; found 1163.4875.

*3-(Allyloxy)-2-(((3R,4S,5S,6R)-3,4,5-tris(benzyloxy)-6-(((bis(2-((tert-butoxycarbonyl)amino)ethoxy)phosphoryl)oxy)methyl)tetrahydro-2H-pyran-2-yl)oxy)propyl dodecanoate* (**26**) Starting with **18,** the alcohol used was *N*-Boc ethanolamine. The product was eluted with PE/EtOAc 8:2. The product was obtained contaminated with *N*-Boc ethanolamine, and the mixture appeared as colourless oil. The product was used without further purification (*α*/*β* 1:1). *β*-anomer: ^1^H NMR (400 MHz, CDCl_3_) δ 7.40–7.25 (m, 15H, ArH), 5.93–5.76 (m, 1H, H-15), 5.28–5.10 (m, 3H, H-16, NH), 5.02–4.95 (m, 1H, CH_2_Ar), 4.93 (d, *J* = 10.7 Hz, 1H, CH_2_Ar), 4.80 (d, *J* = 11.8 Hz, 1H, CH_2_Ar), 4.77–4.69 (m, 2H, CH_2_Ar), 4.61 (d, *J* = 11.5 Hz, 1H, CH_2_Ar), 4.56–4.49 (m, 1H, H-1), 4.35–4.28 (m, 1H, H-9a), 4.21 (dd, *J* = 12.0, 6.0 Hz, 1H, H-9b), 4.18–3.92 (m, 9H, H-6, H-8, H-14, H-17), 3.84–3.77 (m, 2H, H-2, H-4), 3.72–3.67 (m, 1H, H-7a), 3.62–3.49 (m, 3H, H-3, H-5, H-7b), 3.42–3.27 (m, 4H, H-18), 2.30 (t, *J* = 7.6 Hz, 1H, H-10a), 2.19–2.12 (m, 1H, H-10b), 1.63–1.48 (m, 2H, H-11), 1.44 (m, 18H, *tert*-butyl), 1.35–1.18 (m, 16H, H-12), 0.88 (t, *J* = 6.7 Hz, 3H, H-13). ^13^C NMR (101 MHz, CDCl_3_) δ 173.74, 173.65, 155.84, 138.80, 138.73, 138.36, 138.32, 138.26, 134.55, 134.51, 128.43, 128.34, 128.24, 128.07, 127.89, 127.77, 127.70, 127.62, 127.51, 127.49, 117.17, 117.10, 103.91, 103.65, 81.88, 79.72, 79.19, 79.14, 77.24, 76.56, 76.34, 75.02, 74.98, 74.48, 73.53, 73.49, 73.24, 72.97, 72.35, 69.66, 69.61, 67.24, 67.18, 66.36, 66.31, 64.02, 63.82, 59.54, 40.90, 38.16, 34.23, 34.08, 31.94, 31.92, 31.26, 29.71, 29.67, 29.63, 29.51, 29.49, 29.38, 29.35, 29.31, 29.30, 29.19, 29.14, 28.40, 24.92, 24.83, 22.70. HRMS-ESI: Calculated for [C_59_H_89_N_2_O_16_P+Na^+^]: 1135.5842; found 1135.5824.

General procedure for hydrogenation. The starting material was dissolved in dry EtOH 0.1 M, and then 5% of Pd/C (10% *w*/*w*) was added. The reaction was conducted in a Parr Shaker 3900 series hydrogenation apparatus under a 3 bar H_2_ atmosphere and stirring. The system was purged 3 times with H_2_ before starting. Completion was followed by TLC and ^1^H NMR. After completion, the solvent was evaporated. If product **1** is the final product of hydrogenation, then **1** was purified by reverse chromatography and eluted with a gradient of water/MeOH in 10% increments.

General procedure for Boc removal. To this, 5 mL of DCM/TFA 1:1 was added, and the reaction was followed by TLC with ninhydrin staining. After 1h, the reaction was complete. The solvents were evaporated. The final product **1** was purified by reverse chromatography and eluted with a gradient of water/MeOH in 10% increments.

*2-O-D-(galactopyranosyl)glycerol* (**1a**) Galactoglycerol **16** was subjected to hydrogenation conditions. The product **1a** was obtained as a yellowish, highly hygroscopic oil (*α*/*β* 3:1). ^1^H NMR (400 MHz, MeOD) δ 5.03 (d, *J* = 3.2 Hz, 1H, H-1*α*), 4.38 (d, *J* = 7.6 Hz, 1H, H-1*β*), 4.04–3.99 (m, 1H), 3.90–3.88 (m, 1H), 3.85–3.64 (m, 16H), 3.61–3.47 (m, 4H). HRMS-ESI: Calculated for [C_9_H_18_O_8_+Na^+^] 277.0894; found 277.0891.

*3-Hydroxy-2-galactopyranosylpropyl dioctyl phosphate* (**1b**) Starting material **21** was subjected to hydrogenation conditions. Product **1b** was obtained as a colourless oil (*α*/*β* 1:1). ^1^H NMR (400 MHz, MeOD) δ 5.07–5.00 (m, 1H, H-1*α*), 4.38 (d, *J* = 7.5 Hz, 1H, H-1*β*), 4.25–4.14 (m, 4H, H-6), 4.13–4.04 (m, 9H, H-4, H-10), 4.04–3.94 (m, 3H, H-4, H-8), 3.93–3.86 (m, 2H, H-5), 3.84–3.63 (m, 9H, H-2*α*, H-7, H-9), 3.57–3.50 (m, 1H, H-2*β*), 3.50–3.43 (m, 2H, H-3*α*), 1.75–1.65 (m, 8H, H-11), 1.38–1.20 (m, 40H, H-12), 0.93–0.86 (m, 12H, H-13). ^13^C NMR (101 MHz, MeOD) δ 104.99, 100.60, 76.88, 74.81, 72.48, 71.49, 71.08, 70.23, 69.57, 62.82, 62.53, 40.43, 32.97, 31.37, 31.30, 30.75, 30.35, 30.23, 26.61, 23.71, 14.43. HRMS-ESI: Calculated for [C_25_H_51_O_11_P+Na^+^] 581.3061; found 581.3055.

*3-Hydroxy-2-galactopyranosylpropyl (bis(2-aminoethyl)) phosphate* (**1c**) Starting material **22** was subjected to hydrogenation conditions and then Boc removal conditions. The product **1c** was obtained as an orange viscous oil (*α*/*β* 1:1). ^1^H NMR (400 MHz, D_2_O) δ 5.21–5.17 (m, 1H, H-1), 4.49–4.42 (m, 5H, H-6a, H-10), 4.38–4.31 (m, 1H, H-6b), 4.10–4.04 (m, 2H, H-4, H-8), 4.03–3.99 (m, 1H, H-5), 3.93–3.84 (m, 2H, H-2, H-3), 3.84–3.73 (m, 4H, H-7, H-9), 3.43–3.38 (m, 4H, H-11). ^13^C NMR (101 MHz, D_2_O) δ 98.83, 76.54, 76.47, 71.94, 69.95, 69.84, 68.88, 68.00, 67.94, 65.52, 65.47, 61.81, 61.36, 40.12, 40.04. LCMS: 421 [M+H]^+^, 443 [M+Na]^+^.

*Bis(2-Aminoethyl) (galactopyranosyl)glycerol)-6-phosphate* (**1d**) Starting material **23** was subjected to hydrogenation conditions and then Boc removal conditions. Product **1d** was obtained as an orange viscous oil. Only the *α*-anomer was recovered. *α*-anomer: ^1^H NMR (400 MHz, MeOD) δ 5.08 (d, *J* = 3.5 Hz, 1H, H-1), 4.25–4.10 (m, 5H, H-5, H-10), 4.07–4.00 (m, 2H, H-6), 3.99–3.95 (m, 1H, H-4), 3.87–3.62 (m, 7H, H-2, H-7, H-8, H-9), 3.28–3.17 (m, 4H, H-11). ^13^C NMR (101 MHz, MeOD) δ 97.91, 78.83, 69.00, 68.92, 64.20, 61.36, 61.07, 60.63, 59.87, 39.28, 39.22. HRMS: Calculated for [C_11_H_24_O_11_NP–C_2_H_6_N]^−^: 376.1014; found 376.1009. Calculated for [C_11_H_24_O_11_NP + Na^+^–C_2_H_5_N]: 400.0985; found 400.0974.

*3-Hydroxy-2-(galactopyranosyl)propyl dodecanoate* (**1e**) Starting material **19** was subjected to hydrogenation conditions. The product **1e** was obtained as a colourless viscous oil (*α*/*β* 1:1). The ^1^H NMR signals of the diastereomers were assigned according to the anomers *α* and *β*. ^1^H NMR (400 MHz, MeOD) δ 5.09–5.02 (m, 2H, H-1*α*), 4.43–4.38 (m, 2H, H-1*β*), 4.34–4.17 (m, 4H, H-7), 4.05–3.89 (m, 4H, H-4, H-8), 3.87–3.62 (m, 10H, H-2*α*, H-3*α*, H-6, H-9), 3.60–3.46 (m, 4H, H-2*β*, H-3*β*, H-5), 2.43–2.32 (m, 4H, H-10), 1.70–1.57 (m, 4H, H-11), 1.41–1.18 (m, 32H, H-12), 0.96–0.87 (m, 6H, H-13). ^13^C NMR (101 MHz, MeOD) δ 174.04, 174.01, 103.72, 103.34, 98.96, 98.75, 78.12, 77.74, 76.79, 76.11, 75.43, 75.36, 73.46, 71.34, 71.15, 71.08, 70.03, 69.71, 69.58, 68.88, 68.77, 63.56, 63.34, 63.20, 62.95, 61.93, 61.59, 61.42, 61.08, 60.59, 33.56, 31.67, 29.34, 29.22, 29.07, 29.03, 28.83, 24.62, 24.57, 22.33, 13.04. HRMS-ESI: Calculated for [C_21_H_40_O_9_P+Na^+^] 459.2564; found 459.2558.

*3-Hydroxy-2-(galactopyranosyl)propyl bis(3-phenylpropyl) phosphate* (**1f**) Starting material **24** was subjected to hydrogenation conditions. The product **1f** was obtained as a colourless viscous oil (*α*/*β* 1:10). *β*-anomer: ^1^H NMR (400 MHz, MeOD) δ 7.32–7.13 (m, 10H, ArH), 4.42–4.35 (m, 1H, H-1), 4.27–4.18 (m, 2H, H-6), 4.14–4.05 (m, 4H, H-10), 4.01–3.93 (m, 1H, H-5), 3.84–3.80 (m, 1H, H-8), 3.78–3.66 (m, 4H, H-3, H-4, H-7), 3.59–3.43 (m, 3H, H-2, H-9), 2.73 (t, *J* = 7.6 Hz, 4H, H-12), 2.05–1.96 (m, 4H, H-11). ^13^C NMR (101 MHz, MeOD) δ 140.93, 129.17, 128.14, 128.12, 125.71, 103.74, 77.88, 71.08, 67.29, 31.68, 31.61, 31.20, 29.36. HRMS-ESI: Calculated for [C_27_H_39_O_11_P+Cl^−^]: 605.1924; found 605.1925.

*3-Hydroxy-2-(galactopyranosyl)propyl (bis(3-(3,4-dimethoxyphenyl)propyl) phosphate* (**1g**) Starting material **25** was subjected to hydrogenation conditions. The product was obtained as a colourless viscous oil and a mixture of 4 diastereomers (*α*/*β* 1:0.75). ^1^H NMR (400 MHz, MeOD) δ 6.86–6.70 (m, 12H, ArH), 5.05–5.01 (m, 1H, H-1*α*), 4.39–4.34 (m, 1H, H-1*β*), 4.28–4.19 (m, 4H, H-6), 4.13–4.03 (m, 8H, H-10), 4.00–3.93 (m, 2H), 3.92–3.85 (m, 4H), 3.85–3.63 (m, 33H, -OCH_3_, H-2*α*, H-7, H-9), 3.58–3.42 (m, 4H, H-2*β*), 2.66 (d, *J* = 3.8 Hz, 8H, H-11), 2.06–1.94 (m, 8H, H-12). ^13^C NMR (101 MHz, MeOD) δ 135.34, 135.32, 129.60, 121.88, 113.69, 113.32, 105.08, 100.69, 76.93, 74.90, 72.94, 72.56, 71.58, 71.16, 70.43, 68.82, 62.92, 62.75, 62.35, 56.63, 56.55, 33.20, 33.13, 32.20. HRMS-ESI: Calculated for [C_31_H_47_O_15_P+Na^+^]: 713.2545; found 713.2531.

*2-(6-(bis(2-Aminoethoxy))phosphoryl)galactopyranosyl-3-hydroxypropyl dodecanoate* (**1h**) The starting material **26** (119 mg) was dissolved in 2 mL of DCM/MeOH 1:1. A catalytic amount of PdCl_2_ (0.2 mol eq) was added, and the reaction proceeded at rt. After completion (followed by TLC), the reaction was quenched with a few drops of Et_3_N, and the black precipitate that formed was filtered. The orange solution obtained was diluted with DCM and washed with HCl 0.1M (2 × 10 mL), saturated NaHCO_3_ (10 mL), and brine (10 mL). The organic phase was dried over Na_2_SO_4_, and the solvent was evaporated. The resulting crude was subjected to hydrogenation and then Boc removal conditions. Product **1h** (23.2 mg, 36% yield) was obtained as an orange viscous oil (*α*/*β* 1:2). *α*-anomer: ^1^H NMR (400 MHz, MeOD) δ 5.13–5.07 (m, 1H, H-1), 4.49–4.15 (m, 10H, H-4, H-5, H-6, H-9, H-14), 4.04–3.83 (m, 2H, H-3, H-8), 3.83–3.64 (m, 3H, H-2, H-7), 3.39–3.27 (m, 4H, H-15), 2.41–2.33 (m, 2H, H-10), 1.69–1.58 (m, 2H, H-11), 1.41–1.25 (m, 16H, H-12), 0.92 (t, *J* = 6.7 Hz, 3H, H-13). *β*-anomer: ^1^H NMR (400 MHz, MeOD) δ 4.49–4.15 (m, 10H, H-1, H-5, H-6, H-9, H-14), 4.04–3.83 (m, 2H, H-4, H-8), 3.83–3.64 (m, 2H, H-7), 3.60–3.52 (m, 2H, H-2, H-3), 3.39–3.27 (m, 4H, H-15), 2.41–2.33 (m, 2H, H-10), 1.69–1.58 (m, 2H, H-11), 1.41–1.25 (m, 16H, H-12), 0.92 (t, *J* = 6.7 Hz, 3H, H-13). *αβ*-anomers: ^13^C NMR (101 MHz, MeOD) δ 175.48, 105.20, 104.90, 101.05, 100.22, 80.07, 79.85, 78.92, 77.76, 74.66, 74.58, 74.52, 74.44, 74.35, 72.44, 72.18, 70.86, 70.67, 70.07, 69.91, 69.33, 68.82, 65.87, 65.81, 64.66, 64.58, 64.44, 64.36, 63.07, 62.96, 62.42, 62.14, 40.86, 40.78, 35.06, 34.99, 34.95, 34.91, 33.04, 30.75, 30.72, 30.63, 30.60, 30.45, 30.41, 30.25, 30.23, 27.57, 27.56, 26.07, 26.00, 25.93, 23.71, 14.42. HRMS-ESI: Calculated for [C_23_H_46_NO_12_P+Na^+^]: 582.2650; found 582.2644.

### 3.3. General Information for Biological Assays

The following reagents were purchased from Sigma-Aldrich Co. LLC. (St. Louis, MO, USA): phorbol-12-myristate-13-acetate (PMA), Triton X-100, diphenyleneiodonium chloride (DPI), N-acetyl-3,7-dihydroxyphenoxazine (Amplex Red), horseradish peroxidase (HRP), histopaque 1077, histopaque 1119, Dulbecco’s Phosphate Buffer saline without calcium chloride and magnesium chloride (PBS), trypan blue solution 0.4%, dimethylsulphoxide (DMSO), trizma, D-(+)-glucose, and quercetin. 2-[6-(4-Aminophenoxy)-3-oxo-3H-xanthen-9-yl]benzoic acid (APF) was purchased from Invitrogen, Life Technologies Ltd. (Paisley, UK). Luminol was purchased from Fluka Chemie GmbH (Steinheim, Germany). Calcium chloride dihydrate and magnesium sulphate were purchased from Merck (Darmstadt, Germany). Potassium chloride was purchased from Pronalab (Abrunheira, Portugal). Sodium chloride from Honeywell Riedel-de Haën (Hanover, Germany). The FITC Annexin V Apoptosis Detection Kit was obtained from BD Pharmingen™ (Franklin Lakes, NJ, USA). All chemicals and solvents used in the synthesis procedures were obtained from commercial sources and used as received. Tris-glucose buffer (pH = 7.4): [CaCl_2_] = 1.26 mM, [KCl] = 5.37 mM, [MgSO_4_] = 0.81 mM, [NaCl] = 140 mM, [Trizma] = 25 mM, [D-glucose] = 5.5 mM.

### 3.4. Isolation of Human Neutrophils

All patient-related procedures and protocols were performed in accordance with the Declaration of Helsinki and approved by the Ethics Committee of the Centro Hospitalar do Porto, Portugal. After informed consent, venous blood was collected from healthy human volunteers by antecubital venipuncture into K_3_EDTA vacuum tubes, and the isolation of human neutrophils was performed as previously reported by the gradient density centrifugation method [82]. Approximately 3 mL of Histopaque 1077 was carefully added on top of approximately 3 mL of Histopaque 1119 in a centrifuge tube. The blood was slowly added to the top layer and centrifuged at 900× *g*, for 30 min at 20 °C. For the isolation of neutrophils, the granulocyte layer was collected and the rest discarded. PBS was added, and the tube was centrifuged at 850× *g* for 5 min at 4 °C. The supernatant was discarded, and the pellet was resuspended in 1.25 mL of PBS, and 4 mL of water was added to lyse red blood cells. The tube was homogenised and kept at room temperature for approximately 3 min, after which 2.2 mL of 3% NaCl was added to establish medium isotonicity. The cell suspension was centrifuged at 850× *g*, for 5 min at 4 °C. The supernatant was discarded, and the cells were suspended in Tris-glucose buffer and kept on ice under soft shaking. The trypan blue exclusion method was used to assess cell viability and determine cell yield, using a Neubauer chamber and an optical microscope. Cell density was further adjusted with Tris-glucose buffer according to the requirements of the performed assays.

### 3.5. Assessment of Neutrophils’ Apoptosis Versus Necrosis

The effect of the compounds under study on neutrophil viability was evaluated using flow cytometry. This assessment involved staining with Annexin V (AV) and propidium iodide (PI) from the BD Pharmingen™ FITC Annexin V Apoptosis Detection Kit, following the manufacturer’s protocol. Neutrophils (1 × 10^6^ cells/mL) were incubated in a 24-well plate, in the dark, at 37 °C for 45 min with positive control (Triton X-100, 0.025%) or the tested compounds (in DMSO, 5% in the well) under various concentrations (up to 100 μM). Afterwards, the content of each well was transferred to a conical microtube and centrifuged at 400× *g*, at 20 °C, for 5 min. The neutrophils’ pellets were resuspended in PBS and centrifuged again under the previous conditions. The supernatant was removed, and the solution of Annexin V and PI from the kit was added, resuspending the pellet. The neutrophils were incubated in the dark at room temperature for 15 min and then diluted (dilution factor = 6) with the diluted binding buffer. The measurements were performed in an Accuri C6 flow cytometer (BD, Becton, Dickinson and Company, Franklin Lakes, NJ, USA), and the results were treated with BD Accuri™ C6 software (version 1.0.264.21). In the flow cytometer, the fluorescence signal of at least 10,000 neutrophils per sample was collected in logarithmic mode and followed in channels 1 and 3. A polygon gate, corresponding to the neutrophil’s population, was drawn according to the light-scattering properties of neutrophils in a forward versus scatter plot. Thus, debris and other blood cells were unaccounted for in the samples. The green fluorescence corresponding to Annexin V was monitored in channel 1 and plotted as a histogram of FL1 staining, whereas the fluorescence corresponding to PI was monitored in channel 3 and plotted in the same histogram as FL3 staining. Neutrophils’ cell death was expressed as a relative percentage of viable, apoptotic, late apoptotic, and necrotic cells compared to a negative control (untreated cells).

### 3.6. Evaluation of Neutrophils’ Oxidative Burst

#### 3.6.1. Oxidation of Luminol

Luminol is easily oxidized by reactive species produced during neutrophils’ oxidative burst, resulting in a chemiluminescent signal [83]. The effect of the compounds under study on the modulation of neutrophil oxidative burst was determined after stimulation with PMA, a known neutrophil activator, because its effect is closely analogous to that obtained during the phagocytic process [84]. Following a previously reported method [85], in a 96-well plate, neutrophils were incubated in the dark at 37 °C for 5 min with luminol (500 μM) and the tested compounds (10–100 μM) or DMSO (final concentration 4%). Quercetin, a flavonoid known for its anti-inflammatory activity [82] (0.3–5.0 μM), and the inhibitor of NADPH oxidase, DPI (0.01–10 μM), were used as controls. After incubation, PMA (160 nM) was added, and the chemiluminescence was measured in a microplate reader (Synergy HT, BIO-TEK Instruments, Inc., Winooski, VT, USA). Kinetic readings were initiated immediately after cell stimulation. Measurements were taken at the peak of the curve. This peak was observed at around 10 min. Effects are expressed as the inhibition percentage of luminol oxidation. The results obtained represent at least three independent experiments.

#### 3.6.2. Oxidation of APF

APF is a non-fluorescent derivative of fluorescein, which is oxidized by HOCl [86], ONOO^−^, and HO^•^ [87], in a concentration-dependent manner. Its selectivity was tested using the myeloperoxidase (MPO) inhibitor, 4-aminobenzoic acid hydrazide (ABAH). The addition of ABAH to human neutrophils, stimulated with PMA, decreased the APF-dependent fluorescence signal to the level of the control assay, ruling out the involvement of HO^•^ and ONOO^−^ [88]. The previously established methodology for monitoring oxidative burst modulation was adapted [85]. In a 96-well plate, neutrophils (2 × 10^6^ cells/mL) were incubated in the dark at 37 °C for 5 min with APF (2 μM) and the tested compounds (10–100 μM) or DMSO (4%). Quercetin (0.3–5.0 μM) and DPI (0.01–10 μM) were used as controls. After incubation, PMA (160 nM) was added, and the fluorescence (excitation at 495 nm and emission at 528 nm) was measured in a microplate reader (Synergy HT, BIO-TEK Instruments, Inc., Winooski, VT, USA). Kinetic readings were initiated immediately after the addition of APF. Obtained values correspond to the slope measured between 5 and 15 min. Effects are expressed as the percentage inhibition of APF oxidation. The results obtained represent at least three independent experiments.

#### 3.6.3. Oxidation of Amplex Red

Amplex Red is a highly sensitive and chemically stable fluorescent probe for the extracellular detection of H_2_O_2_ [82]. The methodology used previously for monitoring the modulation of the oxidative burst was adapted [85]. In a 96-well plate, neutrophils (2 × 10^6^ cells/mL) were incubated in the dark at 37 °C for 5 min with Amplex Red (25 μM), horseradish peroxidase (0.25 U/mL), and the compounds under study (10–100 μM) or DMSO (4%). Quercetin (0.3–5.0 μM) and DPI (0.01–10 μM) were used as controls. After incubation, PMA (160 nM) was added, and the fluorescence (excitation at 530 nm and emission at 590 nm) was measured in a microplate reader (Synergy HT, BioTek Instruments, Inc., Winooski, VT, USA). Obtained values correspond to the slope measured between 5 and 10 min. Effects are expressed as the percentage inhibition of Amplex Red oxidation. The results obtained represent at least three independent experiments.

### 3.7. Statistical Analysis

GraphPad Prism 6 software was used to plot the inhibition percentage versus compound concentration curves, enabling the determination of the concentration required to achieve 50% inhibition (IC_50_). GraphPad Prism 6 software was also used to perform all statistical analyses. Statistical comparison among the most active 2-SC was estimated by applying the one-way analysis (ANOVA), followed by the Bonferroni’s multiple comparisons test. In all cases, *p*-values < 0.05 were considered statistically significant. The results are expressed as mean ± standard error of the mean (SEM).

## 4. Conclusions

A series of novel floridoside derivatives were synthetized using D-galactose and (±)-solketal as starting materials. Glycosylation of appropriate protected derivatives promoted by NIS and TfOH afforded the corresponding galactoglycerols in good yields. Subsequent phosphorylation was carried out using POCl_3_ followed by esterification with various alcohols to generate the desired phosphotriester derivatives. Final global deprotection provided the target compounds **1a**–**h**. These compounds were evaluated for cytotoxicity and their ability to modulate human neutrophils’ oxidative burst.

At a concentration of 50 μM, compounds **1b** and **1h** demonstrated cytotoxic effects, as evidenced by an increased proportion of neutrophils in the late apoptotic stage, suggesting potential detrimental effects associated with the phosphotriester moiety.

Notably, compound **1e**, an acylated floridoside derivative lacking the phosphate group, exhibited no cytotoxicity under the same conditions, highlighting the possibility that the phosphate ester contributes to cytotoxic outcomes.

Compound **1e** was the most effective in suppressing neutrophils oxidative burst as measured by luminol. It demonstrated an improvement over the floridoside *α*/*β* mixture **1a**, which displayed no relevant activity, albeit more notable than **1c**, **1d**, **1f**, and **1h**. Compounds **1b** and **1h** demonstrated similar levels of luminol oxidation inhibition comparable to **1a**, indicating that all these compounds might be promising in modulating the oxidative burst after structural improvements.

In this study, we report for the first time the synthesis and modulatory effect of human neutrophils’ oxidative burst of floridoside-based phosphotriesters. Although a comprehensive structure–activity relationship remains to be established, the current findings suggest that the presence and nature of the lipidic chains critically influence bioactivity. These preliminary results provide a foundation for the rational design and development of novel floridoside-derived anti-inflammatory agents.

## Figures and Tables

**Table 1:** General synthetic strategy to achieve the assigned compounds **1a**–**h**.

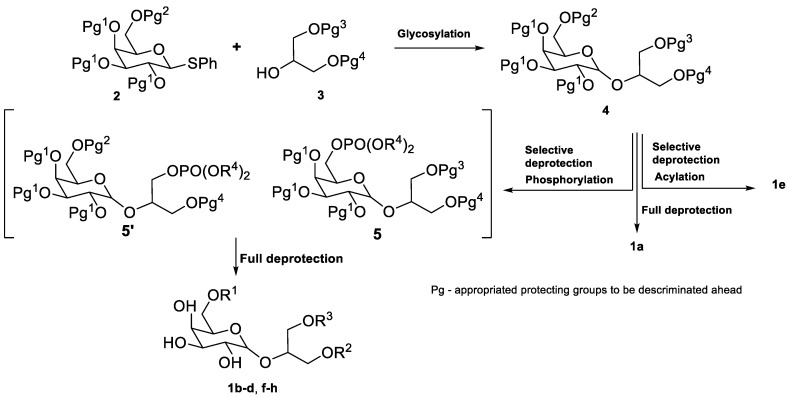				
	R^1^	R^2^	R^3^	*α/β*
**1a**	H	H	H	3:1
**1b**	H	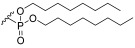	H	1:1
**1c**	H	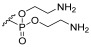	H	1:1
**1d**	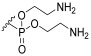	H	H	1:0
**1e**	H	H	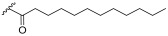	1:1
**1f**	H	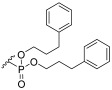	H	1:10
**1g**	H	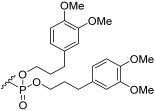	H	1.3:1
**1h**	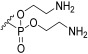	H	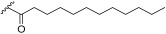	1:2

**Table 2:** Inhibition of PMA-stimulated neutrophils’ oxidative burst by tested compounds and positive controls. Results represent the highest obtained % of inhibition at the respective concentration (μM) or IC_50_ (μM, mean ± SEM).

Compounds	Luminol	APF	Amplex Red
**1a**	34 ± 7% ^100µM^	27 ± 6% ^50µM^	<20% ^50µM^
**1b**	36 ± 9% ^25µM^	29 ± 10% ^25µM^	<20% ^25µM^
**1c**	<20% ^100µM^	22 ± 8% ^50µM^	<20% ^50µM^
**1d**	<20% ^100µM^	21 ± 6% ^50µM^	30 ± 9% ^50µM^
**1e**	83 ± 7 μM	<20% ^50µM^	<20% ^50µM^
**1f**	<20% ^100µM^	<20% ^50µM^	<20% ^50µM^
**1g**	<20% ^100µM^	<20% ^50µM^	<20% ^50µM^
**1h**	27 ± 15% ^25µM^	<20% ^25µM^	<20% ^25µM^
Quercetin	1.2 ± 0.2 μM	2.4 ± 0.2 μM	4.1 ± 0.3 μM
DPI	0.08 ± 0.01 μM	0.030 ± 0.003 μM	0.060 ± 0.004 μM

## Data Availability

The original contributions presented in this study are included in the article/Supplementary Materials. Further inquiries can be directed to the corresponding authors.

## Supplementary Materials

The following supporting information can be downloaded at: https://www.mdpi.com/article/10.3390/molecules30132850/s1, Experimental details regarding synthetic procedures and characterization of the compounds (NMR and MS spectra). Refs. [40,78,79,82,83,84,85,86,87,88,89,90,91,92,93,94,95,96,97,98,99,100] are cited in the Supplementary Materials.

## Abbreviations

The following abbreviations are used in this manuscript:

Ac	Acetyl
Ac_2_O	Acetic Anhydride
AcOH	Acetic Acid
APF	Aminophenyl fluorescein
AR	Amplex Red
AV	Annexin V
Bn	Benzyl
Boc	*N-Tert*-Butyloxycarbonyl
DBTO	Dibutyl Tin Oxide
DCC	*N,N’*-Dicyclohexylcarbodiimide
DCM	Dichloromethane
DGDG	Digalactosyldiacylglycerol
DMAP	4-Dimethylaminopyridine
DMF	Dimethyl Formamide
DNA	Deoxyribonucleic Acid
DPI	Diphenyleneiodonium Chloride
Et_3_N	Triethylamine
EtOH	Ethanol
FSC	Forward Scatter
GPL	Glycophospholipids
GSH-Px	Glutathione Peroxidase
HO-1	Hemoxygenase-1
HRMS	High-Resolution Mass Spectra
iNOS	Inducible Nitric Oxide Synthase
LPS	Lipopolysaccharide
MeOH	Methanol
MGDG	Monogalactosyldiacylglycerol
MMP-2	Matrix Metalloproteinase-2
MPO	Myeloperoxidase
MS	Molecular Sieves
NADPH	Nicotinamide Adenine Dinucleotide Phosphate
NIS	N-iodosuccinimide
NMR	Nuclear Magnetic Resonance
OAc	Acetate
OMe	Methoxide
PGL	Phosphoglycolipids
PhSH	Thiophenol
PI	Propidium Iodide
PMA	Phorbol 12-Myristate 13-Acetate
RNS	Reactive Nitrogen Species
ROS	Reactive Oxygen Species
rt	Room Temperature
SEM	Standard Error of Mean
SOD	Superoxide Dismutase
SSC	Side Scatter
TBAF	Tetrabutylammonium Fluoride
TBDPS	*Tert*-Butyldiphenylsilyl
TFA	Trifluoroacetic Acid
TfOH	Trifluoromethanesulphonic Acid
THF	Tetrahydrofuran
TsOH	*P*-Toluenesulfonic Acid

## References

[1] Fischer W. (1990). Bacterial Phosphoglycolipids and Lipoteichoic Acids. Glycolipids, Phosphoglycolipids, and Sulfoglycolipids.

[2] Pinheiro L., Freitas M., Branco P.S. (2024). Phosphate-Containing Glycolipids: A Review on Synthesis and Bioactivity. ChemMedChem.

[3] Nagatsuka Y., Kasama T., Ohashi Y., Uzawa J., Ono Y., Shimizu K., Hirabayashi Y. (2001). A new phosphoglycerolipid; phosphatidylglucose’, found in human cord red cells by multi-reactive monoclonal anti-i cold agglutinin, mAb GL-1/GL-2. FEBS Lett..

[4] Guy A.T., Nagatsuka Y., Ooashi N., Inoue M., Nakata A., Greimel P., Inoue A., Nabetani T., Murayama A., Ohta K. (2015). Glycerophospholipid regulation of modality-specific sensory axon guidance in the spinal cord. Science.

[5] Li X., Hanafusa K., Kage M., Yokoyama N., Nakayama H., Hotta T., Oshima E., Kano K., Matsuo I., Nagatsuka Y. (2021). Iwabuchi, Lysophosphatidylglucoside is a GPR55-mediated chemotactic molecule for human monocytes and macrophages. Biochem. Biophys. Res. Commun..

[6] Antonopoulou S., Nomikos T., Oikonomou A., Kyriacou A., Andriotis M., Fragopoulou E. (2005). Pantazidou, Characterization of bioactive glycolipids from *Scytonema julianum* (cyanobacteria). Comp. Biochem. Physiol. B Biochem. Mol. Biol..

[7] Tempesta M.S., Jolad S.D., King S., Mao G., Bruening R.C., Kuo J.E., Truong T.V., Bierer D.E., Dener J.M. (1993). Novel Class of Phosphocholine Derivatives Having Antifungal Activity.

[8] Yang F.L., Hua K.F., Yang Y.L., Zou W., Chen Y.P., Liang S.M., Hsu H.Y., Wu S.H. (2008). TLR-independent induction of human monocyte IL-1 by phosphoglycolipids from thermophilic bacteria. Glycoconj. J..

[9] Ostash B., Makitrynskyy R., Yushchuk O., Fedorenko V. (2022). Structural diversity, bioactivity, and biosynthesis of phosphoglycolipid family antibiotics: Recent advances. BBA Adv..

[10] Manzo E., Gallo C., Sartorius R., Nuzzo G., Sardo A., De Berardinis P., Fontana A. (2019). Cutignano, Immunostimulatory Phosphatidylmonogalactosyldiacylglycerols (PGDG) from the Marine Diatom Thalassiosira weissflogii: Inspiration for a Novel Synthetic Toll-Like Receptor 4 Agonist. Mar. Drugs.

[11] Osawa T., Fujikawa K., Shimamoto K. (2024). Structures, functions, and syntheses of glycero-glycophospholipids. Front. Chem..

[12] Simon-Colin C., Kervarec N., Pichon R. (2002). Deslandes, Complete 1H and 13C spectral assignment of floridoside. Carbohydr. Res..

[13] Bondu S., Cerantola S., Kervarec N., Deslandes E. (2009). Impact of the salt stress on the photosynthetic carbon flux and 13C-label distribution within floridoside and digeneaside in Solieria chordalis. Phytochemistry.

[14] Courtois A. (2008). Floridoside Extracted from the Red Alga Mastocarpus stellatus Is a Potent Activator of the Classical Complement Pathway. Mar. Drugs.

[15] Hagemann M., Pade N. (2015). Heterosides–compatible solutes occurring in prokaryotic and eukaryotic phototrophs. Plant Biol..

[16] Bisson M.A., Kirst G.O. (1979). Osmotic Adaption in the Marine Alga *Griffithsia monilis* (Rhodophyceae): The Role of Ions and Organic Compounds. Funct. Plant Biol..

[17] Simon-Colin C., Kervarec N., Pichon R., Deslandes E. (2004). NMR ^13^C-isotopic enrichment experiments to study carbon-partitioning into organic solutes in the red alga *Grateloupia doryphora*. Plant Physiol. Biochem..

[18] Martinez-Garcia M., van der Maarel M.J.E.C. (2016). Floridoside production by the red microalga *Galdieria sulphuraria* under different conditions of growth and osmotic stress. AMB Express.

[19] Luo Q., Zhu Z., Zhu Z., Yang R., Qian F., Chen H., Yan X. (2014). Different Responses to Heat Shock Stress Revealed Heteromorphic Adaptation Strategy of *Pyropia haitanensis* (Bangiales, Rhodophyta). PLoS ONE.

[20] Kim M.J., Li Y.X., Dewapriya P., Ryu B., Kim S.K. (2013). Floridoside suppresses pro-inflammatory responses by blocking MAPK signaling in activated microglia. BMB Rep..

[21] Ochsenkühn M.A., Röthig T., D’Angelo C., Wiedenmann J., Voolstra C.R. (2017). The role of floridoside in osmoadaptation of coral-associated algal endosymbionts to high-salinity conditions. Sci. Adv..

[22] Li Y.X., Li Y., Lee S.H., Qian Z.J.I., Kim S.E.K. (2010). Inhibitors of oxidation and matrix metalloproteinases, floridoside, and d-lsofloridoslde from marine red alga *Laurencia undulata*. J. Agric. Food Chem..

[23] Pullar J.M., Vissers M.C.M., Winterbourn C.C. (2000). Living with a Killer: The Effects of Hypochlorous Acid on Mammalian Cells. IUBMB Life.

[24] Davies M.J. (2011). Myeloperoxidaseederived oxidation: Mechanisms of biological damage and its prevention Overview of the Action of Myeloperoxidase and Other Heme Peroxidases. J. Clin. Biochem. Nutr..

[25] Kelly P.J., Morrow J.D., Ning M., Koroshetz W., Lo E.H., Terry E., Milne G.L., Hubbard J., Lee H., Stevenson E. (2008). Oxidative Stress and Matrix Metalloproteinase-9 in Acute Ischemic Stroke. Stroke.

[26] Hampton M.B., Kettle A.J., Winterbourn C.C. (1998). Inside the Neutrophil Phagosome: Oxidants, Myeloperoxidase, and Bacterial Killing. Blood.

[27] Quinn M.T., Gauss K.A. (2004). Structure and regulation of the neutrophil respiratory burst oxidase: Comparison with nonphagocyte oxidases. J. Leukoc. Biol..

[28] Nauseef W.M. (2007). How human neutrophils kill and degrade microbes: An integrated view. Immunol. Rev..

[29] Nauseef W.M., Borregaard N. (2014). Neutrophils at work. Nat. Immunol..

[30] Halliwell B. (1996). Antioxidants in Human Health and Disease. Annu. Rev. Nutr..

[31] Mydock L.K., Demchenko A.V. (2010). Mechanism of chemical O-glycosylation: From early studies to recent discoveries. Org. Biomol. Chem..

[32] Escopy S., Demchenko A.V. (2022). Transition-Metal-Mediated Glycosylation with Thioglycosides. Chem. A Eur. J..

[33] Singh Y., Geringer S.A., Demchenko A.V. (2022). Synthesis and Glycosidation of Anomeric Halides: Evolution from Early Studies to Modern Methods of the 21st Century. Chem. Rev..

[34] Tokatly A.I., Vinnitskiy D.Z., Ustuzhanina N.E., Nifantiev N.E. (2021). Protecting Groups as a Factor of Stereocontrol in Glycosylation Reactions. Russ. J. Bioorganic Chem..

[35] Mukherjee M.M., Ghosh R., Hanover J.A. (2022). Recent Advances in Stereoselective Chemical O-Glycosylation Reactions. Front. Mol. Biosci..

[36] Chen A., Yang B., Zhou Z., Zhu F. (2022). Recent advances in transition-metal-catalyzed glycosyl cross-coupling reactions. Chem Catal..

[37] Andreu A., Ćorović M., Garcia-Sanz C., Santos A.S., Milivojević A., Ortega-Nieto C., Mateo C., Bezbradica D., Palomo J.M. (2023). Enzymatic Glycosylation Strategies in the Production of Bioactive Compounds. Catalysts.

[38] Schmidt R.R. (1986). New Methods for the Synthesis of Glycosides and Oligosaccharides—Are There Alternatives to the Koenigs-Knorr Method? [New Synthetic Methods (56)]. Angew. Chem. Int. Ed. Engl..

[39] Zhu Z.-Y., Cui D., Gao H., Dong F.-Y., Liu X., Liu F., Chen L., Zhang Y. (2016). Efficient synthesis and activity of beneficial intestinal flora of two lactulose-derived oligosaccharides. Eur. J. Med. Chem..

[40] Jacobson J.M., Kitov P.I., Bundle D.R. (2013). The synthesis of a multivalent heterobifunctional ligand for specific interaction with Shiga toxin 2 produced by *E. coli* O157:H7. Carbohydr. Res..

[41] Trinderup H.H., Andersen S.M., Heuckendorff M., Jensen H.H. (2021). How do Various Reaction Parameters Influence Anomeric Selectivity in Chemical Glycosylation with Thioglycosides and NIS/TfOH Activation?. Eur. J. Org. Chem..

[42] Dinkelaar J., De Jong A.R., Van Meer R., Somers M., Lodder G., Overkleeft H.S., Codée J.D.C., Van Der Marel G.A. (2009). Stereodirecting effect of the pyranosyl C-5 substituent in glycosylation reactions. J. Org. Chem..

[43] Crich D., Cai W. (1999). Chemistry of 4,6-O-benzylidene-D-glycopyranosyl triflates: Contrasting behavior between the gluco and manno series. J. Org. Chem..

[44] Tam P.H., Lowary T.L. (2010). Epimeric and amino disaccharide analogs as probes of an α-(1→6)-mannosyltransferase involved in mycobacterial lipoarabinomannanbiosynthesis. Org. Biomol. Chem..

[45] Aragozzini F., Maconi E., Potenza D., Scolastico C. (1989). Enantioselective Microbial Reduction of Monoesters of 1,3-Dihydroxypropanone: Synthesis of (S)- and (R)-1,2-O-Isopropylideneglycerol. Synthesis.

[46] Yoshida M., Saito K., Kato H., Tsukamoto S., Doi T. (2018). Total Synthesis and Biological Evaluation of Siladenoserinol A and its Analogues. Angew. Chem.-Int. Ed..

[47] Köhler P., Grosch W. (1999). Study of the Effect of DATEM. 1. Influence of Fatty Acid Chain Length on Rheology and Baking. J. Agric. Food Chem..

[48] Bondarev I. (2007). Prevention and Treatment of Cancer and Other Diseases.

[49] Veeneman G.H., van Leeuwen S.H., van Boom J.H. (1990). Iodonium ion promoted reactions at the anomeric centre. II An efficient thioglycoside mediated approach toward the formation of 1,2-trans linked glycosides and glycosidic esters. Tetrahedron Lett..

[50] Hada N., Morita T., Ueda T., Masuda K., Nakane H., Ogane M., Yamano K., Schweizer F., Kiuchi F. (2021). Synthesis of the Carbohydrate Moiety of Glycoproteins from the Parasite *Echinococcus granulosus* and Their Antigenicity against Human Sera. Molecules.

[51] Lipták A., Jodál I., Nánási P. (1975). Stereoselective ring-cleavage of 3-*O*-benzyl- and 2,3-di-*O*-benzyl-4,6-*O*-benzylidenehexopyranoside derivatives with the LiAlH_4_-AlCl_3_, reagent. Carbohydr. Res..

[52] Mikami T., Asano H., Mitsunobu O. (1987). Acetal-bond Cleavage of 4,6-O-Alkylidenehexopyranosides by Diisobutylaluminium Hydride and by Lithium Triethylborohydride-TiCl_4_. Chem. Lett..

[53] Oikawa M., Liu W.-C., Nakai Y., Koshida S., Fukase K., Kusumoto S. (1996). Regioselective Reductive Opening of 4,6-O-Benzylidene Acetals of Glucose or Glucosamine Derivatives by BH_3_⋅Me2NH-BF_3_⋅OEt_2_. Synlett.

[54] Wang C.-C., Luo S.-Y., Shie C.-R., Hung S.-C. (2002). Metal Trifluoromethanesulfonate- Catalyzed Regioselective Borane-Reductive Ring Opening of Benzylidene Acetals: A Concise Synthesis of 1,4-Dideoxy-1,4-imino-l-xylitol. Org. Lett..

[55] Shie C., Tzeng Z., Kulkarni S.S., Uang B., Hsu C., Hung S. (2005). Cu(OTf) 2 as an Efficient and Dual-Purpose Catalyst in the Regioselective Reductive Ring Opening of Benzylidene Acetals. Angew. Chem. Int. Ed..

[56] Ahmadipour S., Miller G.J. (2017). Recent advances in the chemical synthesis of sugar-nucleotides. Carbohydr. Res..

[57] Fiore M. (2018). The synthesis of mono-alkyl phosphates and their derivatives: An overview of their nature, preparation and use, including synthesis under plausible prebiotic conditions. Org. Biomol. Chem..

[58] Gan C.H., Wijaya H., Li L.H., Wei C.F., Peng Y.J., Wu S.H., Hua K.F., Lam Y. (2020). H-Phosphonate Synthesis and Biological Evaluation of an Immunomodulatory Phosphoglycolipid from Thermophilic Bacteria. Org. Lett..

[59] Fujimoto Y., Mitsunobe K., Fujiwara S., Mori M., Hashimoto M., Suda Y., Kusumoto S., Fukase K. (2013). Synthesis and biological activity of phosphoglycolipids from *Thermus thermophilus*. Org. Biomol. Chem..

[60] Lin H.J., Adak A.K., Reddy L.V.R., Wu S.H., Lin C.C. (2013). Total synthesis of an immunomodulatory phosphoglycolipid from thermophilic bacteria. Chem. A Eur. J..

[61] Martin S.F., Josey J.A., Wong Y.L., Dean D.W. (1994). General Method for the Synthesis of Phospholipid Derivatives of 1,2-O-Diacyl-sn-Glycerols. J. Org. Chem..

[62] Roussev C.D., Ivanova G.D., Bratovanova E.K., Petkov D.D. (2000). Medium-Controlled Intramolecular Catalysis in the Direct Synthesis of 5′-O-Protected Ribonucleoside 2′- and 3′-Dialkylphosphates. Angew. Chem. Int. Ed..

[63] Huang H., Denne J., Yang C., Wang H., Kang J.Y. (2018). Direct Aryloxylation/Alkyloxylation of Dialkyl Phosphonates for the Synthesis of Mixed Phosphonates. Angew. Chem. Int. Ed..

[64] Lira L.M., Vasilev D., Pilli R.A., Wessjohann L.A. (2013). One-pot synthesis of organophosphate monoesters from alcohols. Tetrahedron Lett..

[65] Huang H., Ash J., Kang J.Y. (2018). Tf_2_O-Promoted Activating Strategy of Phosphate Analogues: Synthesis of Mixed Phosphates and Phosphinate. Org. Lett..

[66] Yashunsky D.V., Borodkin V.S., Ferguson M.A.J., Nikolaev A.V. (2006). The Chemical Synthesis of Bioactive Glycosylphosphatidylinositols from *Trypanosoma cruzi* Containing an Unsaturated Fatty Acid in the Lipid. Angew. Chem. Int. Ed..

[67] Chen J., Hansen T., Zhang Q., Liu D., Sun Y., Yan H., Codée J.D.C., Schmidt R.R., Sun J. (2019). 1-Picolinyl-5-azido Thiosialosides: Versatile Donors for the Stereoselective Construction of Sialyl Linkages. Angew. Chem. Int. Ed..

[68] Larsen E., Kharazmi A., Christensen L.P., Christensen S.B. (2003). An antiinflammatory galactolipid from rose hip (*Rosa canina*) that inhibits chemotaxis of human peripheral blood neutrophils in vitro. J. Nat. Prod..

[69] Colombo D., Compostella F., Ronchetti F., Scala A., Toma L., Mukainaka T., Nagatsu A., Konoshima T., Tokuda H., Nishino H. (1999). Inhibitory effects of monoacylated 2-O-β-galactosylglycerols on Epstein-Barr virus activation: The significant role of the hexanoyl chain. Cancer Lett..

[70] Colombo D., Compostella F., Ronchetti F., Scala A., Toma L., Tokuda H., Nishino H. (2000). Glycoglycerolipid analogues active as anti-tumor-promoters: The influence of the anomeric configuration. Eur. J. Med. Chem..

[71] Stonik V.A., Stonik I.V. (2023). Carbohydrate-Containing Low Molecular Weight Metabolites of Microalgae. Mar. Drugs.

[72] Hrabák A., Vercruysse V., Kahán I.L., Vray B. (2001). Indomethacin prevents the induction of inducible nitric oxide synthase in murine peritoneal macrophages and decreases their nitric oxide production. Life Sci..

[73] Yang D., Liu Z., Li S., Han N., Zhai J., Yin J. (2025). Novel glycolipids from *Potentilla anserina* L. rhizomes: Anti-inflammatory, hepatoprotective activities, and structure-activity relationship studies. Food Biosci..

[74] Banskota A.H., Stefanova R., Gallant P., McGinn P.J. (2013). Mono- and digalactosyldiacylglycerols: Potent nitric oxide inhibitors from the marine microalga *Nannochloropsis granulata*. J. Appl. Phycol..

[75] Banskota A.H., Stefanova R., Sperker S., Melanson R., Osborne J.A., O’Leary S.J.B. (2013). Five new galactolipids from the freshwater microalga *Porphyridium aerugineum* and their nitric oxide inhibitory activity. J. Appl. Phycol..

[76] Liu D., Wang Y., Lu Z., Lv F., Bie X., Zhao H. (2023). Separation, characterization and anti-inflammatory activities of galactoglycerolipids from *Perilla frutescens* (L.) Britton. Nat. Prod. Res..

[77] Matsufuji M., Nagamatsu Y., Yoshimoto A. (2000). Protective effects of bacterial glyceroglycolipid M874B against cell death caused by exposure to heat and hydrogen peroxide. J. Biosci. Bioeng..

[78] Williams D.B.G., Lawton M. (2010). Drying of Organic Solvents: Quantitative Evaluation of the Efficiency of Several Desiccants. J. Org. Chem..

[79] Stahl E., Ashworth M.R.F. (1969). Thin-Layer Chromatography.

[80] Metten K.-H., Welzel P. (1990). Synthesis of the repeating unit of the capsular antigen of *Neisseria meningitidis* serogroup H. Tetrahedron.

[81] Compostella F., Colombo D., Ferraboschi P., Scala A., Toma L., Ronchetti F. (2002). Synthesis of Isosteric Analogues of Acylglycosylglycerols Active as Chemoprevention Agents. Eur. J. Org. Chem..

[82] Ribeiro D., Freitas M., Tomé S.M., Silva A.M.S., Porto G., Fernandes E. (2013). Modulation of human neutrophils’ oxidative burst by flavonoids. Eur. J. Med. Chem..

[83] Allen R.C., Loose L.D. (1976). Phagocytic activation of a luminol-dependent chemiluminescence in rabbit alveolar and peritoneal macrophages. Biochem. Biophys. Res. Commun..

[84] DeChatelet L., Shirley P., Johnston R.J. (1976). Effect of phorbol myristate acetate on the oxidative metabolism of human polymorphonuclear leukocytes. Blood.

[85] Freitas M., Costa V.M., Ribeiro D., Couto D., Porto G., Carvalho F., Fernandes E. (2013). Acetaminophen prevents oxidative burst and delays apoptosis in human neutrophils. Toxicol. Lett..

[86] Sumitomo K., Shishido N., Aizawa H., Hasebe N., Kikuchi K., Nakamura M. (2007). Effects of MCI-186 upon neutrophil-derived active oxygens. Redox Rep..

[87] Setsukinai K., Urano Y., Kakinuma K., Majima H.J., Nagano T. (2003). Development of Novel Fluorescence Probes That Can Reliably Detect Reactive Oxygen Species and Distinguish Specific Species. J. Biol. Chem..

[88] Freitas M., Lima J.L.F.C., Fernandes E. (2009). Optical probes for detection and quantification of neutrophils’ oxidative burst. A review. Anal. Chim. Acta.

[89] Vidadala S.R., Thadke S.A., Hotha S., Kashyap S. (2012). Synthesis of thioglycosides from propargyl glycosides exploiting alkynophilic gold catalyst. J. Carbohydr. Chem..

[90] Behera A., Rai D., Kushwaha D., Kulkarni S.S. (2018). Total Synthesis of Trisaccharide Repeating Unit of *O*-Specific Polysaccharide of *Pseudomonas fluorescens* BIM B-582. Org. Lett..

[91] López-Prados J., Cuevas F., Reichardt N.-C., de Paz J.-L., Morales E.Q., Martín-Lomas M. (2005). Design and synthesis of inositolphosphoglycan putative insulin mediators. Org. Biomol. Chem..

[92] Herradón B., Cueto S., Morcuende A., Valverde S. (1993). Regio- and enantioselective esterifications of polyoxygenated compounds catalyzed by lipases. Tetrahedron Asymmetry.

[93] Simas A.B.C., da Silva A.A.T., Filho T.J.D.S., Barroso P.T.W. (2009). Direct selective and controlled protection of multiple hydroxyl groups in polyols via iterative regeneration of stannylene acetals. Tetrahedron Lett..

[94] Ligthart N.A.M., de Geus M.A.R., van de Plassche M.A.T., García D.T., Isendoorn M.M.E., Reinalda L., Ofman D., van Leeuwen T., van Kasteren S.I. (2023). A Lysosome-Targeted Tetrazine for Organelle-Specific Click-to-Release Chemistry in Antigen Presenting Cells. J. Am. Chem. Soc..

[95] Häner M., Hammelev C.H., Pedersen C.M. (2018). Conformational Distortion Using a Molecular Lever: Synthesis and Conformational Studies of Galactoside Derivatives. Eur. J. Org. Chem..

[96] Lv J., Luo T., Zou D., Dong H. (2019). Using DMF as Both a Catalyst and Cosolvent for the Regioselective Silylation of Polyols and Diols. Eur. J. Org. Chem..

[97] Pan D., Sun J., Jin H., Li Y., Li L., Wu Y., Zhang L., Yang Z. (2015). Supramolecular assemblies of novel aminonucleoside phospholipids and their bonding to nucleic acids. Chem. Commun..

[98] Bernardoni B., Di Terlizzi L., Galathri E.M., Kokotos C.G., Fagnoni M., Protti S. (2024). Visible photons for the regioselective nucleophilic ring opening of epoxides. Green Chem..

[99] Batovska D.I., Tsubota S., Kato Y., Asano Y., Ubukata M. (2004). Lipase-mediated desymmetrization of glycerol with aromatic and aliphatic anhydrides. Tetrahedron Asymmetry.

[100] Sutter M., Dayoub W., Métay E., Raoul Y., Lemaire M. (2013). 1-O-Alkyl (di)glycerol ethers synthesis from methyl esters and triglycerides by two pathways: Catalytic reductive alkylation and transesterification/reduction. Green Chem..

