# Supplementary material for: Floridoside Phosphotriester Derivatives: Synthesis and Inhibition of Human Neutrophils’ Oxidative Burst
Source: Molecules. 2025 Jul 3;30(13):2850. doi: 10.3390/molecules30132850 (PMC12251482; doi:10.3390/molecules30132850)
Supplement: Supplementary file 1 [file molecules-30-02850-s001.zip › molecules-3720254-supplementary.pdf]

SUPPORTING INFORMATION

Floridoside Phosphotriester Derivatives: Synthesis and Inhibition of Human Neutrophils' Oxidative Burst

Luís Pinheiro [1], Catarina Cipriano [1], Filipe Santos [1], Patrícia Máximo [1], Eduarda Fernandes [2], Marisa Freitas [2,*] and Paula S. Branco [1,*]

[1] LAQV, REQUIMTE, Department of Chemistry, NOVA School of Science and Technology, Universidade Nova de Lisboa, Campus da Caparica, 2825-149 Caparica, Portugal; l.pinheiro@campus.fct.unl.pt (L.P.); ci.cipriano@campus.fct.unl.pt (C.C.)
[2] LAQV, REQUIMTE, Laboratory of Applied Chemistry, Department of Chemical Sciences, Faculty of Pharmacy, University of Porto, 4050-313 Porto, Portugal; egracas@ff.up.pt
* Correspondence: marysa.freitas@ff.up.pt (M.F.); paula.branco@fct.unl.pt (P.S.B.)

Contents

1. Experimental procedures for compounds 2a-c, 3a-c, and 6-15 .... S1
   1.1 *General information for synthetic procedures* S1
   1.2 *Synthetic Procedures* S2
2. NMR and mass spectra of compounds S22
3. Biological assays S140
   3.1 *General information for biological assays* S140
   3.2 *Isolation of human neutrophils* S140
   3.3 *Assessment of neutrophils' apoptosis versus necrosis* S140
   3.4 Evaluation of neutrophils' oxidative burst S141
      3.4.1 *Oxidation of luminol* S141
      3.4.2 *Oxidation of APF* S142
      3.4.3 *Oxidation of Amplex Red* S142
   3.5 *Statistical Analysis* S142
   3.6 *Cytotoxicity of **1b** and **1h*** S143
   3.7 *IC_{50} of **1e*** S143
4. References S144

1. Experimental procedures for compounds 2a-c, 3a-c, and 6-15

1.1 General information for synthetic procedures

All yields presented are isolated yields. In cases where possible, the proportions of the anomers were determined by calculating the respective integrals in H-1 peaks in the ^{1}H NMR spectra. The presence of the phosphate group was confirmed by ^{31}P NMR. NMR signal attribution is given whenever it was possible to determine. In certain cases, only one of the anomers' signals is attributed due to spectra complexity, even in cases where both anomers or an anomeric mixture were obtained, which is mentioned.

All reagents used in synthesis were purchased from commercial suppliers and used without further purification, unless specified. Solvent drying was performed by using 3Å molecular sieves pre-activated in 5 x 2 minutes on-0.5 minute off cycles under microwave heating and then placed in vacuum [1]. Thin-layer chromatography analysis was performed on Merck Kieselgel GF 254 0.2mm plates supported on aluminium and revealed under UV light (254 nm) and by staining with the appropriate staining spray (MeOH/H_2SO_4 1:1 for carbohydrate compounds, ninhydrin for amine products, and phosphomolybdic acid solution in remaining cases) [2]. Normal-phase column chromatography was performed on the bench with Kieselgel 60A (Carlo Erba), 40-63 µm granulometry ("flash" chromatography), or with the Pure Essential Chromatography System (Pure Chromatography C-900, Pure Fraction Collector C-106, Pure UV Detector C-107) from Buchi (Flawil, Switzerland). Reverse-phase column chromatography was performed with LiChroprep RP-18 (Merck), 40-63 µm granulometry, or CHROMABOND® C18 Hydra SPE polypropylene column cartridges (Carl Roth GmbH, Karlsruhe, Germany).

NMR spectra were acquired with Bruker ARX 400 or Bruker Avance III spectrometers. ^{1}H NMR, ^{13}C NMR, and ^{31}P NMR spectra were acquired at 400, 101, and 162 MHz, respectively. Data were treated with MestreNova software. Chemical shifts (δ) are reported in parts per million (ppm). Coupling constants (J) are reported in Hz. The following NMR abbreviations are used: s = singlet, d = doublet, t = triplet, q = quartet, quint = quintuplet, m = multiplet, dd = doublet of doublets, and dt = doublet of triplets. NMR spectra were calibrated according to the mentioned solvent in each spectrum.

Mass spectra (LCMS) were recorded at the Laboratório de Análises, Requimte, Faculdade de Ciências e Tecnologia, Universidade Nova de Lisboa, using an LC Agilent 1200 Series with Binary pump/MS Agilent 6130B Single Quadrupole with an ESI source. High-resolution mass spectra (HRMS) were obtained at the University of Salamanca (Spain) through the Elemental Analysis, Chromatography and Mass Spectrometry Service (NUCLEUS), using a Thermo QExactive focus spectrometer at a resolution of 30000. For the ionization, ESI in alternating positive and negative mode was used (3.5KV for positive mode and 3.0KV for negative mode).

Infusion was performed through a Thermo Vanquish HPLC with a void union as a column with a flow of methanol of 0.2 ml/min.

1.2 Synthetic Procedures

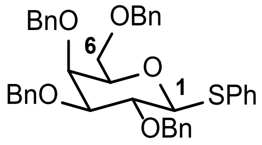

Phenyl 2,3,4,6-tetra-O-benzyl-1-thio-*β*-D-galactopyranoside (2a) 2.31 g (8.5 mmol) of **8** were dissolved in dry DMF (0.15 M) and cooled to 0 °C. NaH (10 eq, 60% w/w in mineral oil) was added in portions under an N_2 atmosphere. After gas evolution subsided, benzyl bromide (5 eq) was added dropwise. The mixture was allowed to reach rt and react overnight. After full consumption of the starting material, water was added, and DMF was co-evaporated with toluene. EtOAc (100 mL) was added, the layers were separated, and the organic phase was washed with water 3 x 50 mL and brine and dried over Na_2SO_4. The mixture was purified by "flash" chromatography and eluted with a petroleum ether (PE)/EtOAc gradient of 9:1 to 7:3. The product (4.46 g) was obtained as a white solid in 83% yield. ^{1}H NMR (400 MHz, $CDCl_3$) δ 7.60 – 7.53 (m, 2H, ArH), 7.41 – 7.23 (m, 20H, ArH), 7.18 (m, 3H, ArH), 4.97 (d, *J* = 11.5 Hz, 1H, C$\underline{H_2}$Ar), 4.81 – 4.71 (m, 4H, C$\underline{H_2}$Ar), 4.64 (d, *J* = 9.7 Hz, 1H, H-1), 4.60 (d, *J* = 11.5 Hz, 1H, C$\underline{H_2}$Ar), 4.48 (d, *J* = 11.7 Hz, 1H, C$\underline{H_2}$Ar), 4.42 (d, *J* = 11.7 Hz, 1H, C$\underline{H_2}$Ar), 3.98 (d, *J* = 2.7 Hz, 1H, H-4), 3.93 (t, *J* = 9.4 Hz, 1H, H-2), 3.69 – 3.56 (m, 4H, H-3, H-5, H-6). NMR data are consistent with the literature [3].

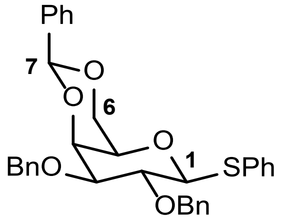

Phenyl 2,3-di-O-benzyl-4,6-di-O-benzylidene-1-thio-*β*-D-galactopyranoside (2b) Product **9** (378 mg, 1.05 mmol) was dissolved in dry DMF (0.15 M) and cooled to 0 °C. NaH (3 eq, 60% w/w dispersion in mineral oil) was added in portions under N_2. After gas evolution subsided, benzyl bromide (537 mg, 3.14 mmol) was added dropwise. The reaction was left to reach rt. After completion, water was added to quench, and DMF was co-evaporated with toluene. EtOAc (20 mL) was added. The organic phase was washed with water (2 x 15 mL) and brine and dried over Na_2SO_4. The mixture was purified by "flash" chromatography and eluted with PE/EtOAc 6:4. The product (488 mg) was obtained as a white solid in 86% yield. ^{1}H NMR (400 MHz, $CDCl_3$) δ 7.74 – 7.68 (m, 2H, ArH), 7.56 – 7.50 (m, 2H, ArH), 7.44 – 7.27 (m, 13H), 7.25 – 7.15 (m, 3H), 5.49 (s, 1H, H-1′), 4.71 (dd, *J* = 6.7, 2.5 Hz, 4H, CH_2Ar), 4.62 (d, *J* = 9.5 Hz, 1H, H-1), 4.38 (dd, *J* = 12.3, 1.7 Hz, 1H, H-6a), 4.16 (d, *J* =

3.4 Hz, 1H, H-4), 3.99 (dd, J = 12.3, 1.7 Hz, 1H, H-6b), 3.90 (t, J = 9.4 Hz, 1H, H-2), 3.63 (dd, J = 9.2, 3.4 Hz, 1H, H-3), 3.42 (m, 1H, H-5). NMR data are consistent with the literature [4].

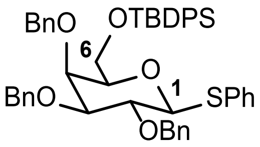

Phenyl 6-O-tert-butyldiphenylsilyl-2,3,4-tri-O-benzyl-1-thio-*β*-D-galactopyranoside (2c) Product **10** (4.61 g, 9.03 mmol) was dissolved in dry DMF (0.25 M) and put in an ice bath for 15 minutes. With strong stirring, NaH (1.62, 40.62 mmol) was added portionwise under N_2 over 30 minutes. After gas evolution subsided, benzyl bromide (5.56 g, 32.50 mmol) was added dropwise over 20 minutes, and the reaction was left in an ice bath to reach room temperature. After completion (confirmed by TLC), water was added slowly to quench. The mixture was added to 150 mL of DCM to dilute. Water (3 x 100mL) was added to wash the organic phase, followed by brine (100 mL). Then, the organic phase was dried over Na_2SO_4. The mixture was purified by "flash" chromatography and eluted with petroleum ether/EtOAc 9.5:0.5. The product (3.53 g) was obtained as a colourless oil in 50% yield. ^{1}H NMR (400 MHz, $CDCl_3$) δ 7.68 – 7.61 (m, 4H, ArH), 7.58 – 7.53 (m, 2H, ArH), 7.47 – 7.27 (m, 21H, ArH), 7.19 – 7.12 (m, 3H, ArH), 5.04 (d, J = 11.4 Hz, 1H, C$\underline{H_2}$Ar), 4.84 – 4.73 (m, 4H, C$\underline{H_2}$Ar), 4.67 (d, J = 11.5 Hz, 1H, C$\underline{H_2}$Ar), 4.61 (d, J = 9.6 Hz, 1H, H-1), 4.02 (d, J = 2.7 Hz, 1H, H-4), 3.97 (t, J = 9.4 Hz, 1H, H-2), 3.90 – 3.85 (m, 2H, H-6), 3.61 (dd, J = 9.2, 2.7 Hz, 1H, H-3), 3.48 (t, J = 6.7 Hz, 1H, H-5), 1.08 (s, 9H, $C(CH_3)_3$). ^{13}C NMR (101 MHz, $CDCl_3$) δ 138.94, 138.49, 138.42, 135.67, 135.62, 134.29, 133.27, 133.26, 131.34, 129.88, 129.82, 128.83, 128.54, 128.40, 128.21, 127.84, 127.82, 127.79, 127.75, 127.64, 127.38, 126.96, 87.63, 84.26, 78.76, 77.31, 75.66, 74.47, 73.71, 72.95, 62.44, 27.03, 27.00, 19.26. NMR data are consistent with the literature [5].

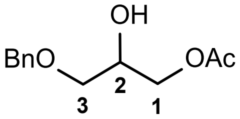

1-Acetoxy-3-benzyloxy-2-propanol (3a) 3-(Benzyloxy)propane-1,2-diol **12** (772 mg, 4.24 mmol) was dissolved in dry toluene (0.125 M), and previously activated 3Å MS were added. After stirring under reflux for 15 min., Bu_2SnO (211 mg, 0.85 mmol) was added and left to react for 2h under reflux. The reaction was left to cool down to rt before AcCl (350 mg, 4.45 mmol) was added dropwise. After reacting for 2h, the reaction was filtered, and the filtrate washed with water (2 x 30 mL), sat. H_2PO_4 and dried over Na_2SO_4. The mixture was purified by "flash" chromatography and eluted with PE/EtOAc 7:3. The product (314 mg) was obtained as a colourless oil in 33% yield. ^{1}H NMR (400 MHz, $CDCl_3$) δ 7.39 – 7.28 (m, 5H, ArH), 4.56 (s, 2H, OC$\underline{H_2}$Ar), 4.21 – 4.10 (m, 2H, H-1), 4.07 – 4.00 (m, 1H, H-2), 3.56 (dd, J = 9.6, 4.3 Hz, 1H, H-3a), 3.49 (dd, J = 9.6, 6.1 Hz, 1H, H-3b), 2.07 (s, 3H, OAc). NMR data are consistent with the literature [6].

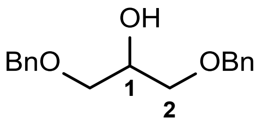

1,3-Benzyloxy-2-propanol (3b) To benzyl alcohol (3.51 g, 32.4 mmol), at 40 °C, was added 5 mL of water containing NaOH (1.08 g, 27.0 mmol). After 30 min., epichlorohydrin **13** (1 g, 10.8 mmol) was added dropwise. The reaction stayed overnight at 40 °C and turned yellow after. Toluene and HCl 1M were added until neutralization. The phases were separated, and the organic phase was washed with water and brine and then dried over Na_2SO_4. The solvent was evaporated, and the mixture was fractionally distilled under reduced pressure to remove excess benzyl alcohol (140-170 °C, 5 mbar). The product (824 mg) was recovered as a yellowish oil in 28% yield. ^{1}H NMR (400 MHz, $CDCl_3$) δ 7.41 – 7.31 (m, 10H, ArH), 4.58 (s, 4H, OC<u>H</u>$_2$Ar), 4.09 – 4.02 (m, 1H, H-1), 3.58 (m, 4H, H-2). NMR data are consistent with the literature [7].

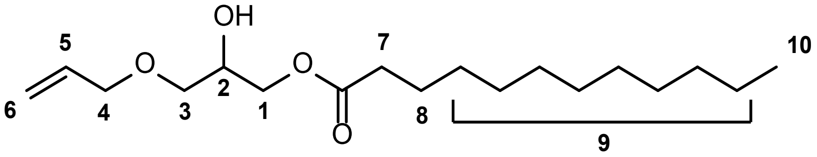

3-(Allyloxy)-2-hydroxypropyl dodecanoate (3c) 2,3-Dihydroxypropyl dodecanoate **15** (1.28 g, 4.66 mmol) was dissolved in dry toluene (0.125 M), and previously activated 3Å MS were added. After stirring under reflux for 15 min., Bu_2SnO (1.28 g mg, 5.14 mmol) was added and left to react for 2h under reflux. The solvent was evaporated, and dry DMF was added (0.125 M). Allyl bromide (596 mg, 4.92 mmol) was added dropwise, and then KF (406 mg, 6.99 mmol) was added in small portions over time. After completion, the reaction was filtered, and the filtrate was co-evaporated with toluene. DCM was added and was washed with water (2 x 30 mL) and sat. H_2PO_4 (30 mL) and dried over Na_2SO_4. The mixture was purified by "flash" chromatography and eluted with PE/EtOAc 8:2. The product (461 mg) was obtained as a colourless oil in 29% yield. ^{1}H NMR (400 MHz, $CDCl_3$) δ 5.89 (ddt, *J* = 17.3, 10.3, 5.7 Hz, 1H, H-5), 5.27 (dq, *J* = 17.3, 1.6 Hz, 1H, H-6a), 5.20 (dq, *J* = 10.4, 1.4 Hz, 1H, H-6b), 4.15 (qd, *J* = 11.5, 5.3 Hz, 2H, H-1), 4.04 – 3.96 (m, 3H, H-2, H-4), 3.51 (dd, *J* = 9.7, 4.3 Hz, 1H, H-3a), 3.45 (dd, *J* = 9.7, 6.2 Hz, 1H, H-3b), 2.33 (t, *J* = 7.6 Hz, 2H, H-7), 1.62 (p, *J* = 7.2 Hz, 2H, H-8), 1.27 (d, *J* = 16.5 Hz, 16H, H-8), 0.87 (t, *J* = 6.8 Hz, 3H, H-10). ^{13}C NMR (101 MHz, $CDCl_3$) δ 173.91, 134.24, 117.51, 72.40, 70.85, 65.38,

34.19, 31.92, 29.61, 29.47, 29.34, 29.27, 29.14, 24.94, 22.70. HRMS-ESI: Calculated for [$C_{18}H_{34}O_4+Na^+$]: 337.2349; found: 337.2345.

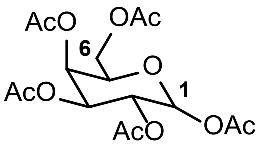

D-Galactose peracetate (6) To a mixture of sodium acetate (1.82 g, 22.2 mmol) and acetic anhydride (10.5 mL) previously refluxed for 15 minutes, D-galactose (2.0 g, 11.1 mmol) was added. The reaction was allowed to proceed at reflux overnight, after which the mixture was poured into 100 mL of icy water and stirred for 18h. EtOAc was used to extract the aqueous phase (3 x 50 mL). The combined organic phases were washed with brine (50 mL) and dried over Na_2SO_4. The crude mixture was purified by "flash" chromatography, using petroleum ether (PE)/EtOAc 2:1 as eluent, yielding the product (4.07 g) as a white waxy solid in 94% isolated yield (α/β 1:3). ^{1}H NMR (400 MHz, $CDCl_3$) δ 5.68 (d, J = 8.2 Hz, 1H, H-1 (***β*-anomer**), 5.42 – 5.38 (m, 1H, H-4), 5.31 (dd, J = 10.5, 8.3 Hz, 1H, H-2), 5.06 (dd, J = 10.4, 3.4 Hz, 1H, H-5), 4.18 – 4.01 (m, 3H, H-3, H-6), 2.14 (s, 3H, OAc), 2.10 (s, 3H, OAc), 2.02 (s, 6H, 2x OAc), 1.97 (s, 3H, OAc). NMR data are consistent with the literature [8].

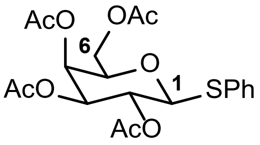

Phenyl 2,3,4,6-tetra-O-acetyl-1-thio-*β*-D-galactopyranoside (7) D-galactose peracetate **(6)** (3.518 g, 9.0 mmol) was dissolved in dry DCM and cooled to 0 °C. Thiophenol (1.19 g, 10.8 mmol) was added followed by the dropwise addition of $BF_3.Et_2O$ (1.91 g, 13.5 mmol, 1.69 mL). The reaction was left to react at rt. After completion, the reaction was diluted with DCM and quenched with sat. $NaHCO_3$. The organic phase was then washed with brine and dried over Na_2SO_4. The mixture was purified by "flash" chromatography and eluted with PE/EtOAc 1:1. The product (3.77 g) was obtained as a white solid in 95% yield. ^{1}H NMR (400 MHz, $CDCl_3$) δ 7.55 – 7.48 (m, 2H, ArH), 7.36 – 7.28 (m, 3H, ArH), 5.42 (dd, J = 3.3, 1.1 Hz, 1H, H-4), 5.24 (t, J = 10.0 Hz, 1H, H-2), 5.05 (dd, J = 9.9, 3.3 Hz, 1H, H-3), 4.72 (d, J = 10.0 Hz, 1H, H-1), 4.22 – 4.16 (m, 1H, H-6a), 4.16 – 4.07 (m, 1H, H-6b), 3.96 – 3.91 (m, 1H, H-5), 2.12 (s, 3H, OAc), 2.10 (s, 3H, OAc), 2.04 (s, 6H, 2x OAc), 1.97 (s, 3H, OAc). NMR data are consistent with the literature [9].

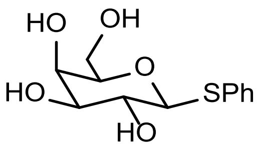

1-Thiophenyl-*β*-D-galactopyranoside (8) To 3.74 g (8.5 mmol) of **7** dry MeOH and 230 mg (4.25 mmol, 0.5 eq) of NaOMe were added. The reaction was allowed to proceed at rt., until full consumption of the starting material. After neutralization with a few drops of HCl 1M, the solvent was evaporated, and the crude was used without further purification.

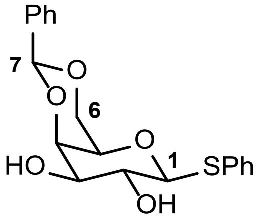

Phenyl 4,6-di-O-benzylidene-1-thio-*β*-D-galactopyranoside (9) In dry DMF, 572 mg (2.1 mmol) of **8** were reacted with benzaldehyde dimethyl ketal (648 mg, 4.3 mmol) in the presence of catalytic *p*-toluenesulphonic acid monohydrate (25 mg, 0.1 mmol), under reflux. After 3h no further progress was observed, and the reaction was quenched with sat. $NaHCO_3$. The aqueous phase was extracted with EtOAc (3 x 10 mL). The combined organic phases were washed with brine and dried over Na_2SO_4. The product (378 mg) was obtained from recrystallization with n-Hexane/EtOAc mixture and was obtained as white needle crystals in 49% yield (over two steps). ^{1}H NMR (400 MHz, $CDCl_3$) δ 7.73 – 7.67 (m, 2H, ArH), 7.42 – 7.35 (m, 5H, ArH), 7.35 – 7.27 (m, 3H, ArH), 5.52 (s, 1H, H-7), 4.52 (d, *J* = 9.3 Hz, 1H, H-1), 4.40 (dd, *J* = 12.5, 1.6 Hz, 1H, H-6a), 4.26 – 4.22 (m, 1H, H-4), 4.05 (dd, *J* = 12.5, 1.8 Hz, 1H, H-6b), 3.75 – 3.68 (m, 2H, H-2, H-3), 3.60 – 3.56 (m, 1H, H-5), 2.52 – 2.46 (m, 2H, OH). NMR data are consistent with the literature [10].

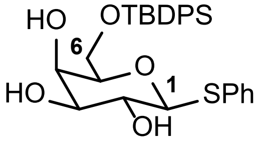

Phenyl 6-*O*-(*tert*-butyl(diphenyl)silyl)-1-thio-*β*-D-galactopyranoside (10) 2.97 g (10.92 mmol) of **8** (10.92 mmol) were dissolved in dry DMF (0.1 M), and imidazole (2.23 g, 32.76 mmol) was added. The solution was cooled in an ice bath and stirred for 15 minutes, after which TBDPSCl (3.57 g, 12.98 mmol, 3.37 mL) was added dropwise over 30 minutes. Progress was monitored by TLC, and after completion, the reaction was quenched with saturated $NaHCO_3$, washed with HCl 1M (2 x 40 mL), saturated $NaHCO_3$ (50 mL), and brine (50 mL) and dried over Na_2SO_4. The mixture was purified by "flash" chromatography and eluted with PE/EtOAc 4:6. The product (3.74 g) was obtained as a colourless oil in 67% yield over two steps. ^{1}H NMR (500 MHz, $CDCl_3$) δ 7.71 (m, 4H, ArH), 7.57 – 7.52 (m, 2H, ArH), 7.47 – 7.35 (m, 6H, ArH), 7.29 – 7.24 (m, 4H, ArH), 4.51 (d, *J* = 9.5 Hz,

1H, H-1), 4.15 – 4.12 (m, 1H, H-4), 4.00 – 3.92 (m, 2H, H-6), 3.68 (t, *J* = 9.3 Hz, 1H, H-2), 3.62 – 3.56 (m, 2H, H-3, H-5), 1.07 (s, 9H, $C(CH_3)_3$). NMR data are consistent with the literature [11].

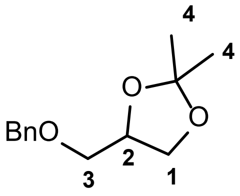

4-(Benzyloxymethyl)-2,2-dimethyl-1,3-dioxolane (11) Commercial 1,2-O-isopropylidene-D-glycerol or (±)-solketal (500 mg, 3.78 mmol) was dissolved in dry DMF 1:3 (0.45 M) and cooled to 0 °C. NaH (196 mg, 4.91 mmol, 60% w/w dispersion in mineral oil) was added in portions under N_2. When gas evolution stopped, BnBr (775 mg, 4.53 mmol) was added dropwise. The reaction was left to proceed and reach rt. After consumption of the starting material (judged by TLC), the solvents were evaporated, and the crude was resuspended in water. EtOAc was used to extract (3 x 15 mL), and the combined organic phases were washed with brine and dried over Na_2SO_4. The mixture was purified by "flash" chromatography and eluted with PE/EtOAc 9:1. The product (832 mg) was obtained as a colourless oil in 99% yield. ^{1}H NMR (400 MHz, $CDCl_3$) δ 7.38 – 7.27 (m, 5H, ArH), 4.63 – 4.53 (m, 2H, OC$\underline{H_2}$Ar), 4.31 (p, *J* = 6.0 Hz, 1H, H-2), 4.06 (dd, *J* = 8.2, 6.5 Hz, 1H, H-1a), 3.75 (dd, *J* = 8.2, 6.4 Hz, 1H, H-1b), 3.56 (dd, *J* = 9.8, 5.7 Hz, 1H, H-3a), 3.48 (dd, *J* = 9.8, 5.5 Hz, 1H, H-3b), 1.42 (s, 3H, H-4), 1.37 (s, 3H, H-4). NMR data are consistent with the literature [12].

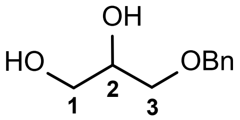

3-(Benzyloxy)propane-1,2-diol (12) 832 mg (3.74 mmol) of 4-(Benzyloxymethyl)-2,2-dimethyl-1,3-dioxolane **(11)** were dissolved in AcOH/H_2O 4:1 (0.4 M) and left to react overnight. The solvent was evaporated, and the product (682 mg) was obtained as a clear yellow oil in 100% yield. ^{1}H NMR (400 MHz, $CDCl_3$) δ 7.39 – 7.28 (m, 5H, ArH), 4.55 (s, 2H, OC$\underline{H_2}$Ar), 3.93 – 3.87 (m, 1H, H-2), 3.70 (dd, *J* = 11.4, 3.8 Hz, 1H, H-1a), 3.63 (dd, *J* = 11.5, 5.5 Hz, 1H, H-1b), 3.60 – 3.51 (m, 2H, H-3). NMR data are consistent with the literature [13].

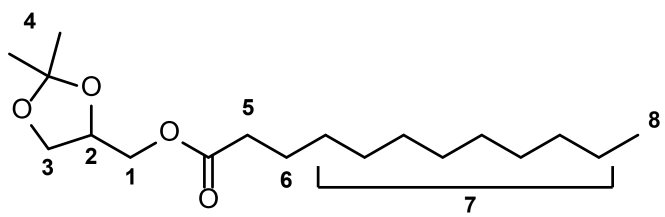

(2,2-Dimethyl-1,3-dioxolan-4-yl)methyl dodecanoate (14) Commercial 1,2-O-isopropylidene-D-glycerol (2.5 g, 18.92 mmol) and dodecanoic acid (4.55 g, 22.7 mmol) were dissolved in dry DCM (0.14 M) and stirred under inert atmosphere at room temperature. Dicyclohexyl carbodiimide (4.68 g, 22.7 mmol) and DMAP (231 mg, 1.89

mmol) were sequentially added, and the reaction stayed overnight. The mixture was filtered, and the filtrate was washed with water (2x 50 mL) and then brine (50 mL) and dried over Na_2SO_4. The mixture was purified by "flash" chromatography and eluted with a PE/EtOAc mixture of 9:1. The product was obtained as a colourless oil with a small contamination of dodecanoic acid and was used without further purification. ^1H NMR (400 MHz, $CDCl_3$) δ 4.30 (p, *J* = 5.9 Hz, 1H, H-2), 4.19 – 4.04 (m, 3H, H-3, H-1a), 3.73 (dd, *J* = 8.2, 6.5 Hz, 1H, H-1b), 2.33 (t, *J* = 7.6 Hz, 2H, H-5), 1.62 (p, *J* = 7.1 Hz, 2H, H-6), 1.42 (s, 3H, H-4), 1.36 (s, 3H, H-4), 1.34 – 1.18 (m, 16H, H-7), 0.87 (t, *J* = 6.6 Hz, 3H, H-8). NMR data are consistent with the literature [14].

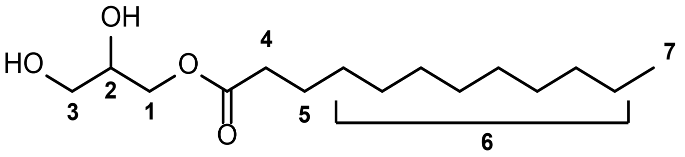

2,3-Dihydroxypropyl dodecanoate (15) Glycerol dodecanoate **14** was dissolved in AcOH/H_2O 4:1 (0.4 M) and left to react overnight. The solvent was evaporated, and the product was purified by "flash" chromatography and eluted with a PE/EtOAc mixture of 6:4. The product was obtained as a colourless oil (1.29 g). ^1H NMR (400 MHz, $CDCl_3$) δ 4.23 – 4.11 (m, 2H, H-1), 3.96 – 3.89 (m, 1H, H-2), 3.73 – 3.65 (m, 1H, H-3), 3.63 – 3.56 (m, 1H, H-3), 2.34 (t, *J* = 7.6 Hz, 2H, H-4), 1.62 (p, *J* = 7.2 Hz, 2H, H-5), 1.37 – 1.20 (m, 16H, H-6), 0.87 (t, *J* = 6.7 Hz, 3H, H-7). NMR data are consistent with the literature [15].

General procedure for the synthesis of 4. In a Schlenk flask, dried overnight at 100 °C and under an N_2 atmosphere with previously activated 3Å MS, were charged dry DCM, the galactose derivative (0.025 M), and the glycerol derivative (1.05 eq). The mixture was stirred for 30 min. at rt, then cooled to -78 °C, and stirred for another 10 min, after which NIS (1.5 eq) and TfOH (0.1 eq) were added sequentially. The reaction was left to slowly reach rt. After full consumption of the galactose derivative, Et_3N was added dropwise to quench (until the reaction mixture turned orange). The sieves were filtered off, and 10% aqueous $Na_2S_2O_3$ was added. The layers were separated, and the organic phase was washed three times with water and then brine. The mixture was purified by "flash" chromatography and eluted with an appropriate mixture of PE/EtOAc.

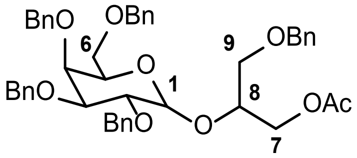

1-*O*-Acetyl-3-*O*-benzyl-2-*O*-(2',3',4',6'-tetra-*O*-benzyl-*β*-D-galactopyranosyl)-sn-glycerol (4a) Eluted with PE/EtOAc 7:3. Obtained as a yellow-orange viscous oil in 84% yield (*α*/*β* 1:2). ***α*-anomer (2 diastereomers):** ^1H NMR (400 MHz, $CDCl_3$) δ 7.42 – 7.21 (m, 25H), 5.16 – 5.12 (m, 1H, H-1), 4.97 – 4.91 (m, 1H, OC<u>H</u>$_2$Ar), 4.86 – 4.64 (m, 4H, OC<u>H</u>$_2$Ar), 4.59 – 4.54 (m, 1H, OC<u>H</u>$_2$Ar), 4.53 – 4.51 (m, 1H, OC<u>H</u>$_2$Ar), 4.47 – 4.45 (m, 1H, OC<u>H</u>$_2$Ar), 4.44

– 4.35 (m, 2H, OCH2Ar), 4.34 – 4.27 (m, 1H, H-7a), 4.21 – 4.00 (m, 4H, H-2, H-4, H-5, H-7b), 3.98 – 3.93 (m, 2H, H-3, H-8), 3.66 – 3.46 (m, 4H, H-6, H-9), 1.99 – 1.92 (m, 3H, OAc). ^{13}C NMR (101 MHz, $CDCl_3$) δ 171.04, 170.98, 139.09, 138.91, 138.84, 138.78, 138.28, 138.11, 128.62, 128.59, 128.56, 128.53, 128.51, 128.48, 128.44, 128.41, 128.08, 128.06, 127.96, 127.94, 127.91, 127.87, 127.83, 127.79, 127.75, 127.66, 127.64, 127.60, 97.37 (C-1), 97.27 (C-1), 79.13, 77.56, 77.44, 77.24, 76.92, 76.61, 76.38, 75.27, 75.10, 75.01, 74.97, 74.19, 74.03, 73.74, 73.65, 73.62, 73.39, 73.32, 73.26, 73.19, 70.02, 69.83, 69.71, 69.66, 69.24, 68.90, 64.84, 64.32, 60.61, 21.02, 20.92. ***β*-anomer (2 diastereomers):** ^{1}H NMR (400 MHz, $CDCl_3$) δ 7.38 – 7.22 (m, 25H, ArH), 4.96 – 4.90 (m, 2H, OCH2Ar), 4.78 – 4.68 (m, 3H, OCH2Ar), 4.61 (d, *J* = 11.6 Hz, 1H, OCH2Ar), 4.51 (s, 2H, OCH2Ar), 4.49 (d, *J* = 7.8 Hz, 1H, H-1), 4.44 – 4.39 (m, 2H, OCH2Ar), 4.35 (dd, *J* = 11.7, 3.4 Hz, 1H, H-7a), 4.22 (dd, *J* = 11.8, 6.3 Hz, 1H, H-7b), 4.10 – 4.04 (m, 1H, H-8), 3.90 – 3.87 (m, 1H, H-4), 3.80 (dd, *J* = 9.6, 7.8 Hz, 1H, H-2), 3.74 (dd, *J* = 9.9, 4.4 Hz, 1H, H-9a), 3.60 (dd, *J* = 7.6, 2.4 Hz, 1H, H-9b), 3.58 – 3.48 (m, 4H, H-6, H-5, H-3, 1.88 (s, 3H, OAc). ^{13}C NMR (101 MHz, $CDCl_3$) δ 138.85, 138.62, 138.49, 138.14, 137.91, 128.45, 128.41, 128.37, 128.34, 128.27, 128.24, 128.21, 128.07, 127.97, 127.91, 127.85, 127.82, 127.70, 127.67, 127.60, 127.56, 127.46, 103.99, 82.19, 79.38, 77.37, 77.25, 77.05, 76.73, 76.46, 75.00, 74.57, 73.54, 73.45, 73.33, 73.14, 73.09, 69.68, 68.88, 64.39, 50.84. HRMS-ESI: Calculated for $[C_{46}H_{50}O_9+Na^+]$ 769.3347; found 769.3340.

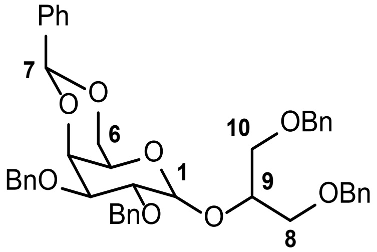

1,3-di-*O*-Benzyl-2-*O*-(2′,3′-di-*O*-benzyl-4′,6′-di-*O*-benzylidene-*α*-D-galactopyranosyl)-sn-glycerol (4b) Eluted with PE/EtOAc 7:3. Both anomers were obtained as yellowish oils in 63% combined yield (*α*/*β* 2:1) ***α*-anomer:** ^{1}H NMR (400 MHz, $CDCl_3$) δ 7.52 – 7.48 (m, 2H, ArH), 7.43 – 7.39 (m, 2H, ArH), 7.39 – 7.22 (m, 21H, ArH), 5.40 (s, 1H, H-7), 5.25 (d, *J* = 3.4 Hz, 1H, H-1), 4.84 – 4.63 (m, 4H, CH2Ar), 4.55 – 4.41 (m, 4H, CH2Ar), 4.15 – 4.09 (m, 2H, H-3, H-5), 4.06 – 3.98 (m, 3H, H-2, H-4, H-8/10), 3.92 - 3.89 (m, 1H, H-9), 3.68 (dd, *J* = 12.5, 1.9 Hz, 1H, H-8/10), 3.65 – 3.62 (m, 2H, H-6), 3.59 (m, 2H, H-8/10). ^{13}C NMR (101 MHz, CDCl3) δ 139.14, 138.88, 138.31, 138.23, 138.09, 128.91, 128.53, 128.51, 128.42, 128.40, 128.36, 128.25, 128.20, 127.93, 127.83, 127.76, 127.70, 127.65, 127.63, 127.60, 126.48, 101.13 (C-7), 97.27 (C-1), 76.21 (C-4), 75.59 (C-2), 74.90 (C-3), 74.79 (C-5), 73.54, 73.29, 73.26,

72.22, 71.01 (C-6), 70.08 (C-9), 69.52 (C-8), 62.60 (C-10). HRMS-ESI: Calculated for [$C_{44}H_{46}O_8$+Na^+]: 727.3085; found: 727.3094.

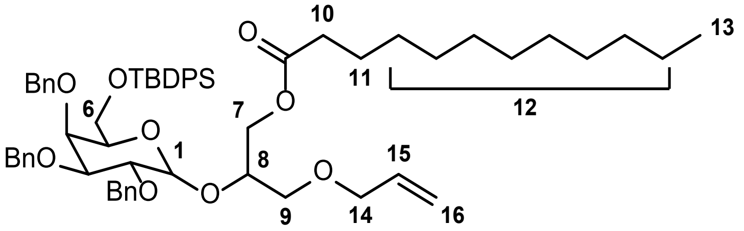

3-(Allyloxy)-2-(((3*R*,4*S*,5*S*,6*R*)-3,4,5-*tris*(benzyloxy)-6-(((*tert*-butyldiphenylsilyl)oxy)methyl)tetrahydro-2*H*-pyran-2-yl)oxy)propyl dodecanoate (4c) Eluted with a mixture of PE/EtOAc 9.5:0.5. The product was obtained as a colourless oil in 63% yield (*α*/*β* 1:1). Since the two anomers were unseparated, the ^{1}H NMR nuclide count is doubled. ^{1}H NMR (400 MHz, $CDCl_3$) δ 7.73 – 7.56 (m, 8H, ArH), 7.50 – 7.18 (m, 42H, ArH), 5.93 – 5.73 (m, 2H, H-15), 5.30 – 5.06 (m, 5H, H-1*α*, H-16), 5.02 – 4.56 (m, 12H, C$\underline{H_2}$Ar), 4.53 – 4.43 (m, 1H, H-1*β*), 4.38 – 4.32 (m, 1H, H-7), 4.28 – 4.12 (m, 3H, H-7), 4.10 – 3.87 (m, 10H, H-2*α*, H-4, H-5*α*, H-8, H-14), 3.84 – 3.63 (m, 6H, H-2*β*, H-6, H-9_{1H}), 3.61 – 3.47 (m, 5H, H-3, H-9_{3H}), 3.43 – 3.37 (m, 1H, H-5*β*), 2.28 – 2.14 (m, 4H, H-10), 1.75 – 1.51 (m, 4H, H-11), 1.41 – 1.19 (m, 32H, H-12), 1.13 – 1.01 (m, 18H, C(C$\underline{H_3}$)$_3$), 0.95 – 0.85 (m, 6H, H-13). ^{13}C NMR (101 MHz, $CDCl_3$) δ 173.75, 173.58, 173.49, 173.43, 139.01, 138.95, 138.82, 138.78, 138.74, 138.71, 138.69, 138.64, 135.64, 135.60, 135.57, 135.55, 135.33, 134.82, 134.58, 134.53, 133.37, 133.32, 133.27, 129.82, 129.78, 129.76, 129.74, 128.37, 128.35, 128.32, 128.28, 128.21, 128.16, 128.14, 128.11, 128.08, 128.01, 127.96, 127.94, 127.86, 127.78, 127.74, 127.68, 127.60, 127.57, 127.54, 127.47, 127.44, 127.40, 127.35, 117.04, 116.99, 116.91, 109.22, 103.98, 103.43, 96.87, 96.77, 82.19, 82.14, 79.50, 79.46, 78.91, 77.37, 77.25, 77.05, 76.73, 76.47, 76.38, 76.32, 76.10, 75.72, 75.23, 74.99, 74.93, 74.88, 74.83, 74.66, 73.89, 73.85, 73.53, 73.27, 73.24, 73.20, 73.13, 72.92, 72.81, 72.32, 72.24, 72.21, 71.22, 71.09, 69.75, 69.63, 69.54, 66.85, 64.59, 64.05, 63.91, 62.59, 62.41, 62.32, 34.19, 34.12, 31.93, 29.73, 29.64, 29.52, 29.49, 29.36, 29.29, 29.17. HRMS-ESI: Calculated for [$C_{61}H_{80}O_9Si$+Na^+] 1007.5459; found 1007.5446.

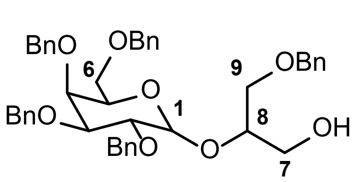

3-*O*-benzyl-2-*O*-(2',3',4',6'-tetra-*O*-benzyl-*β*-D-galactopyranosyl)-sn-glycerol (16) The starting material **4a** was dissolved in MeOH and 0.5 eq of NaOMe were added. The reaction proceeded at rt. After full consumption of the starting material, a few drops of HCl 1M were added to neutralize the reaction. The solvent was evaporated and the crude was dried in a vacuum. The mixture was purified by "flash" chromatography and eluted with petroleum ether/EtOAc 6:4. ***α*-anomer:** ^{1}H NMR (400 MHz, $CDCl_3$) δ 7.41 – 7.24 (m, 25H, ArH), 5.01 (d, *J* = 3.7 Hz, 1H, H-1), 4.93 (d, *J* = 11.4 Hz, 1H, C$\underline{H_2}$Ar), 4.87 (d, *J* = 11.5 Hz, 1H, C$\underline{H_2}$Ar), 4.79 – 4.75 (m, 2H, C$\underline{H_2}$Ar), 4.70

(d, *J* = 11.5 Hz, 1H, C*H₂*Ar), 4.57 (d, *J* = 11.5 Hz, 1H, C*H₂*Ar), 4.50 – 4.42 (m, 2H, C*H₂*Ar), 4.42 – 4.32 (m, 2H, C*H₂*Ar), 4.15 – 4.10 (m, 1H, H-5), 4.10 – 4.04 (m, 1H, H-2), 4.01 – 3.95 (m, 2H, H-3, H-4), 3.89 – 3.81 (m, 1H, H-8), 3.75 – 3.66 (m, 1H, H-6a), 3.65 – 3.42 (m, 5H, H-6b, H-7, H-9). NMR data are consistent with the literature [80]. ***β*-anomer:** ^{1}H NMR (400 MHz, $CDCl_3$) δ 7.45 – 7.20 (m, 25H, ArH), 4.94 (d, *J* = 11.4 Hz, 1H, C*H₂*Ar), 4.90 – 4.81 (m, 1H, C*H₂*Ar), 4.79 – 4.67 (m, 3H, C*H₂*Ar), 4.64 – 4.37 (m, 6H, C*H₂*Ar, H-1), 3.97 – 3.81 (m, 4H), 3.81 – 3.42 (m, 7H).

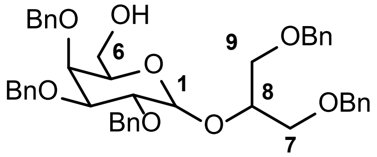

((2*R*,3*S*,4*S*,5*R*)-3,4,5-*Tris*(benzyloxy)-6-((1,3-*bis*(benzyloxy)propan-2-yl)oxy)tetrahydro-2*H*-pyran-2-yl)methanol (17) In a dried round-bottom flask under an N_2 atmosphere, **4b** in THF was added, followed by a catalytic amount (0.15 eq) of $Cu(OTf)_2$. Next, 3 eq of $BH_3.SMe_2$ (2M) or $BH_3.THF$ (1M) were quickly added. After completion, water was added dropwise to quench. After gas evolution stopped, EtOAc was added, and the organic phase was washed with sat. $NaHCO_3$ and brine and dried over Na_2SO_4. The mixture was purified by "flash" chromatography and eluted with a mixture of PE/EtOAc 75:25. The product was obtained in 49% yield as a colourless oil (*α*/*β* 1:1). ***α*-anomer:** ^{1}H NMR (400 MHz, $CDCl_3$) δ 7.43 – 7.22 (m, 25H, ArH), 5.24 (d, *J* = 3.8 Hz, 1H, H-1), 4.95 (d, *J* = 11.6 Hz, 1H, CH₂Ar), 4.89 (d, *J* = 11.6 Hz, 1H, CH₂Ar), 4.74 (d, *J* = 11.6 Hz, 1H, CH₂Ar), 4.72 – 4.69 (m, 2H, CH₂Ar), 4.62 (d, *J* = 11.6 Hz, 1H, CH₂Ar), 4.54 – 4.44 (m, 4H, CH₂Ar), 4.17 – 4.10 (m, 1H, H-5), 4.08 – 3.99 (m, 2H, H-2, H-8), 3.93 (dd, *J* = 10.1, 2.9 Hz, 1H, H-3), 3.86 – 3.83 (m, 1H, H-4), 3.63 – 3.55 (m, 5H, H-6, H-7, H-9a), 3.43 – 3.31 (m, 1H, H-9b). ^{13}C NMR (101 MHz, $CDCl_3$) δ 138.91, 138.55, 138.30, 138.08, 138.04, 128.50, 128.45, 128.42, 128.40, 128.27, 127.87, 127.80, 127.65, 127.55, 127.49, 96.69 (C-1), 79.07 (C-3), 77.25 (C-2), 76.33 (C-5), 75.38 (C-4), 74.64, 74.46, 73.45, 73.42, 73.28, 72.77, 70.73 (C-6), 70.37, 70.30, 62.64, 62.48. ***β*-anomer:** ^{1}H NMR (400 MHz, $CDCl_3$) δ 7.39 – 7.23 (m, 25H, ArH), 5.03 – 4.91 (m, 2H, CH₂Ar), 4.81 (d, *J* = 11.8 Hz, 1H, CH₂Ar), 4.77 – 4.71 (m, 2H, CH₂Ar), 4.66 (d, *J* = 11.9 Hz, 1H, CH₂Ar), 4.58 (d, *J* = 7.7 Hz, 1H, H-1), 4.54 (d, *J* = 2.6 Hz, 2H, CH₂Ar), 4.51 – 4.46 (m, 2H, CH₂Ar), 4.07 (p, *J* = 5.2 Hz, 1H, H-8), 3.84 (dd, *J* = 9.7, 7.6 Hz, 1H, H-3), 3.76 – 3.68 (m, 4H, H-4, H-5, H-7a, H-9a), 3.67 – 3.60 (m, 2H, H-7b, H-9b), 3.51 (dd, *J* = 9.7, 3.0 Hz, 1H, H-2), 3.47 – 3.38 (m, 1H, H-6a), 3.35 – 3.30 (m, 1H, H-6b). ^{13}C NMR (101 MHz, $CDCl_3$) δ 138.92, 138.49, 138.30, 138.24, 128.66, 128.45, 128.44, 128.33, 128.21, 128.07, 127.96, 127.67, 127.63, 127.57, 127.53, 127.41, 103.72 (C-1), 82.32, 79.68, 77.85, 77.23,

75.03, 74.66, 74.13, 73.50, 73.37, 73.31, 73.01, 70.45. HRMS-ESI: Calculated for [$C_{44}H_{48}O_8+Na^+$]: 727.3241; found: 727.3232. NMR data are consistent with the literature [81].

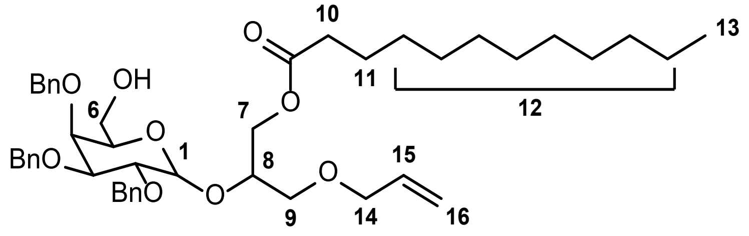

3-(Allyloxy)-2-(((3*R*,4*S*,5*S*,6*R*)-3,4,5-*tris*(benzyloxy)-6-(hydroxymethyl)tetrahydro-2*H*-pyran-2-yl)oxy)propyl dodecanoate (18) To the starting material, **4c** (500 mg, 0.51 mmol) was added 3 equivalents of tetrabutylammonium fluoride (1 M in THF). After 2h and completion of the reaction, the volatiles were evaporated, and the crude mixture was purified by "flash" chromatography and eluted with PE/EtOAc 7:3. The product was obtained as colourless oil in 54% yield (*α*/*β* 1:1). ***α*-anomer:** ^{1}H NMR (400 MHz, $CDCl_3$) δ 7.43 – 7.24 (m, 15H, ArH), 5.85 (ddt, *J* = 17.3, 10.3, 5.6 Hz, 1H, H-15), 5.24 (dq, *J* = 17.3, 1.7 Hz, 1H, H-16a), 5.19 – 5.13 (m, 2H, H-1, H-16b), 4.97 (d, *J* = 11.6 Hz, 1H, C<u>H</u>$_2$Ar), 4.88 (d, *J* = 11.7 Hz, 1H, C<u>H</u>$_2$Ar), 4.80 – 4.75 (m, 2H, C<u>H</u>$_2$Ar), 4.72 (d, *J* = 11.8 Hz, 1H, C<u>H</u>$_2$Ar), 4.64 (d, *J* = 11.7 Hz, 1H, C<u>H</u>$_2$Ar), 4.25 (dd, *J* = 11.7, 4.8 Hz, 1H, H-7a), 4.15 (dd, *J* = 11.7, 6.0 Hz, 1H, H-7b), 4.10 – 3.90 (m, 7H, H-2, H-3, H-4, H-5, H-8, H-14), 3.70 (dd, *J* = 11.3, 6.5 Hz, 1H, H-6a), 3.60 – 3.52 (m, 2H, H-9), 3.52 – 3.46 (m, 1H, H-6b), 2.24 (dd, *J* = 8.1, 7.1 Hz, 2H, H-10), 1.60 – 1.54 (m, 2H, H-11), 1.25 (d, *J* = 5.3 Hz, 16H, H-12), 0.88 (t, *J* = 6.8 Hz, 3H, H-13). ^{13}C NMR (101 MHz, $CDCl_3$) δ 173.64, 138.89, 138.58, 138.34, 134.53 (C-15), 128.69, 128.61, 128.55, 128.51, 128.10, 127.93, 127.81, 127.71, 127.63, 117.50 (C-16), 96.93 (C-1), 79.19, 77.36, 76.92, 76.54, 75.23, 74.55, 74.06, 73.61, 73.26, 72.38 (C-14), 70.73, 70.32, 63.85, 62.79, 34.29, 32.04, 29.74, 29.60, 29.47, 29.41, 29.39, 29.29, 24.99, 22.82, 14.25. ***β*-anomer:** ^{1}H NMR (400 MHz, $CDCl_3$) δ 7.40 – 7.27 (m, 15H), 5.93 – 5.77 (m, 1H), 5.29 – 5.12 (m, 2H), 4.98 – 4.89 (m, 2H), 4.83 – 4.69 (m, 3H), 4.65 (dd, *J* = 11.8, 7.9 Hz, 1H), 4.51 (dd, *J* = 7.7, 2.6 Hz, 1H), 4.34 (ddd, *J* = 26.5, 11.6, 4.1 Hz, 1H), 4.22 (dt, *J* = 12.0, 6.1 Hz, 1H), 4.09 – 3.95 (m, 3H), 3.85 – 3.67 (m, 4H), 3.60 – 3.43 (m, 3H), 3.42 – 3.34 (m, 1H), 2.34 – 2.13 (m, 2H), 1.65 – 1.47 (m, 2H), 1.34 – 1.18 (m, 16H), 0.88 (t, *J* = 6.7 Hz, 3H). ^{13}C NMR (101 MHz, $CDCl_3$) δ 174.06, 173.74, 138.84, 138.77, 138.44, 138.39, 138.24, 138.21, 134.52, 128.69, 128.56, 128.48, 128.44, 128.41, 128.26, 128.09, 128.02, 127.92, 127.71, 127.65, 127.52, 127.50, 117.25, 117.15, 104.19, 103.90, 82.30, 82.26, 79.54, 79.47, 77.36, 77.24, 77.04, 76.78, 76.72, 75.49, 75.07, 75.03, 74.78, 74.21, 74.18, 73.52, 73.47, 73.31, 72.90, 72.40, 72.37, 70.02, 69.98, 64.10, 63.79, 62.48, 61.99, 34.32, 34.10, 31.92,

29.63, 29.48, 29.35, 29.28, 29.15, 29.13, 24.88, 24.84, 22.70, 14.14. HRMS-ESI: Calculated for [$C_{45}H_{62}O_9+Na^+$] 769.4292; found 769.4270.

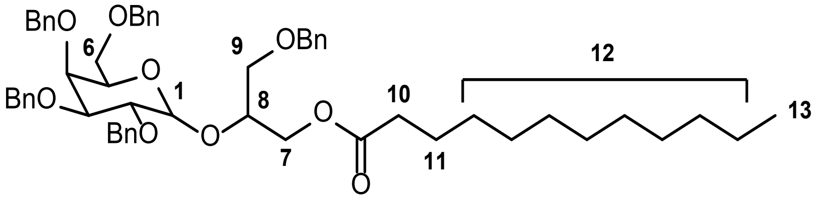

3-(benzyloxy)-2-(((3*R*,4*S*,5*S*,6*R*)-3,4,5-*tris*(benzyloxy)-6-((benzyloxy)methyl)tetrahydro-2*H*-pyran-2-yl)oxy)propyl dodecanoate (19) The starting galactoglycerol **16** (62 mg, 0.088 mmol) was dissolved in dry DCM (0.25 M) under an N_2 atmosphere. Dicyclohexyl carbodiimide (20 mg, 0.097 mmol), DMAP (2.2 mg, 0.018 mmol), and dodecanoic acid (19.4 mg, 0.097 mmol) were sequentially added. The reaction proceeded at room temperature. After completion of the reaction, DCM was added, and the precipitate was filtered. The filtrate was washed with water (3 x 10 mL) and brine (10 mL). The organic phase was dried over Na_2SO_4. The mixture was purified by "flash" chromatography and eluted with PE/EtOAc 7:3. The product was obtained as a white waxy solid in 85% yield (α/β 1:2). ***α*-anomer:** ^{1}H NMR (400 MHz, $CDCl_3$) δ 7.41 – 7.21 (m, 25H, ArH), 5.18 – 5.13 (m, 1H, H-1), 4.97 – 4.91 (m, 1H, C<u>H</u>2Ar), 4.87 – 4.81 (m, 1H, C<u>H</u>2Ar), 4.77 – 4.66 (m, 3H, C<u>H</u>2Ar), 4.65 – 4.44 (m, 3H, C<u>H</u>2Ar), 4.44 – 4.20 (m, 3H, C<u>H</u>2Ar, H-7a), 4.18 – 4.00 (m, 3H, H2, H-5, H-7b), 3.98 – 3.92 (m, 1H, H-3), 3.90 – 3.86 (m, 1H, H-8), 3.65 – 3.45 (m, 5H, H-4, H-6, H-9), 2.39 – 2.10 (m, 2H, H-10), 1.65 – 1.44 (m, 2H, H-11), 1.35 – 1-17 (m, 16H, H-12), 0.88 (t, *J* = 6.8 Hz, 3H, H-13). ***β*-anomer:** ^{1}H NMR (400 MHz, $CDCl_3$) δ 7.41 – 7.21 (m, 25H, ArH), 4.97 – 4.91 (m, 1H, C<u>H</u>2Ar), 4.77 – 4.66 (m, 4H, C<u>H</u>2Ar), 4.65 – 4.44 (m, 4H, C<u>H</u>2Ar, H-1), 4.44 – 4.20 (m, 4H, C<u>H</u>2Ar, H-7a), 4.18 – 4.00 (m, 1H, H-7b, H-8), 3.98 – 3.92 (m, 1H, H-4), 3.90 – 3.86 (m, 1H, H-3), 3.84 – 3.71 8m, 2H, H-2, H-9a) 3.65 – 3.45 (m, 4H, H-5, H-6, H-9b), 2.39 – 2.10 (m, 2H, H-10), 1.65 – 1.44 (m, 2H, H-11), 1.35 – 1-17 (m, 16H, H-12), 0.88 (t, *J* = 6.8 Hz, 3H, H-13). ***αβ*-anomers:** ^{13}C NMR (101 MHz, $CDCl_3$) δ 173.86, 173.75, 139.03, 138.85, 138.72, 138.30, 138.22, 138.13, 138.05, 128.57, 128.54, 128.48, 128.45, 128.43, 128.39, 128.33, 128.20, 128.06, 128.00, 127.97, 127.93, 127.91, 127.88, 127.86, 127.84, 127.80, 127.76, 127.74, 127.70, 127.67, 127.65, 127.56, 127.51, 104.12, 97.21, 97.15, 82.33, 79.51, 79.07, 76.32, 76.16, 75.20, 75.09, 74.95, 74.68, 74.09, 73.88, 73.66, 73.63, 73.58, 73.53, 73.28, 73.05, 70.02, 69.85, 69.55, 69.13, 68.99, 68.86, 64.47, 60.54, 34.28, 34.21, 34.16, 32.05, 29.76, 29.64, 29.61, 29.50, 29.48, 29.44, 29.41, 29.30, 29.26, 25.05, 24.95, 22.83, 14.34, 14.26. HRMS-ESI: Calculated for [$C_{56}H_{70}O_9+Na^+$]: 909.4912; found 909.4899.

The general procedure for the synthesis of phosphoesters 21-26 $POCl_3$ (5 eq) was added to a 0 °C dry DCM solution of dry pyridine (10 eq) 0.25 M under an N_2 atmosphere. After fuming subsided, the clear solution obtained was added to a 0 °C dry DCM solution of dry pyridine (10 eq) and galactoglycerol (100 mg, 0.25 M) under an N_2 atmosphere. After full consumption of the galactoglycerol, the appropriate alcohol (20 eq) was added, and the reaction stayed overnight. The mixture was diluted with DCM and washed with 0.1 M HCl

three times. The organic phase was then washed with brine and dried over Na_2SO_4. Purification was performed/achieved by "flash" chromatography and eluted with an appropriate mixture of PE/EtOAc.

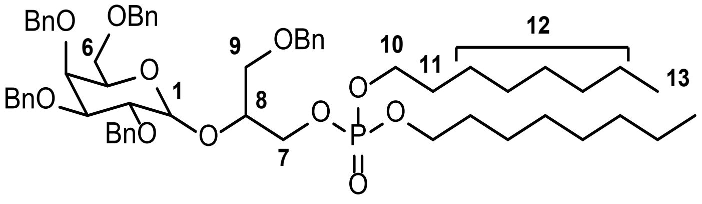

3-*O*-Benzyl-2-*O*-(2',3',4',6'-tetra-*O*-benzyl-D-galactopyranosyl)propyl dioctyl phosphate (21): Starting with **16,** the alcohol used was *n*-octanol. The crude mixture was eluted with PE/EtOAc 8:2. The product was obtained as colourless oil in 52% yield (α/β 1:1). **α-anomer:** ^{1}H NMR (400 MHz, CDCl3) δ 7.39 – 7.19 (m, 25H, ArH), 5.17 (d, J = 3.8 Hz, 1H, H-1), 4.92 (d, J = 11.4 Hz, 1H, C$\underline{H}_2$Ar), 4.82 (d, J = 11.7 Hz, 1H, C$\underline{H}_2$Ar), 4.73 – 4.63 (m, 3H, OC$\underline{H}_2$Ar), 4.57 – 4.48 (m, 4H, C$\underline{H}_2$Ar), 4.41 (d, J = 11.7 Hz, 1H, C$\underline{H}_2$Ar), 4.23 – 4.11 (m, 3H, H-6, H-8), 4.11 – 3.95 (m, 8H, H-2, H-3, H-4, H-5, H-10), 3.66 – 3.60 (m, 2H, H-9), 3.55 (dd, J = 6.7, 1.6 Hz, 2H, H-7), 1.63 – 1.55 (m, 4H, H-11), 1.30 – 1.19 (m, 20H, H-12), 0.87 (m, 6H, H-13). ^{13}C NMR (101 MHz, $CDCl_3$) δ 138.73 - 137.95, 128.39 - 127.36, 97.08 (C-1), 78.91 (C-2), 76.24 (C-3), 75.03 (C-4), 74.81, 74.47 (C-5), 73.47, 73.40 (CH_2, CH_2Ar), 73.11, 72.86, 69.42 (C-8), 69.22 (C-9), 68.74 (C-7), 67.91 (C-10), 67.10 (C-6), 31.79 (C-10), 30.35 (C-11), 30.28 (C-11), 29.20 - 22.64 (C-12), 14.09 (C-13). LCMS: 1009.5 $[M+H]^+$; 1026.5 $[M+NH_4]^+$.

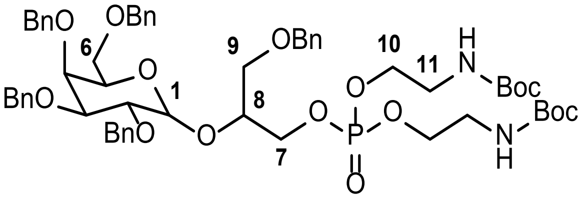

Di-*tert*-butyl (2-(((3-(benzyloxy)-2-(((3*R*,4*S*,5*S*,6*R*)-3,4,5-*tris*(benzyloxy)-6-((benzyloxy)methyl)tetrahydro-2*H*-pyran-2-yl)oxy)propoxy)(2-(methylamino)ethoxy)phosphoryl)*bis*(oxy)*bis*(ethane-2,1-diyl)dicarbamate (22) Starting with **16,** the alcohol used was *N*-Boc ethanolamine. The crude mixture was eluted with PE/EtOAc 6:4. The product was confirmed to be present by ^{1}H, albeit heavily contaminated with *tris*(*N*-Boc ethanolamine)

phosphate, which co-elutes with the intended product. The mixture was used in the next reaction without further purification. LCMS: 1088.5 $[M+NH_4]^+$; 1093.4 $[M+Na]^+$.

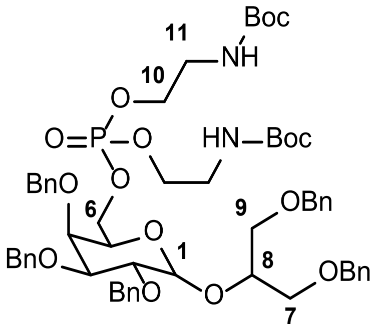

Di-*tert*-butyl (2-(((2*R*,3*S*,4*S*,5*R*)-3,4,5-*tris*(benzyloxy)-6-((1,3-*bis*(benzyloxy)propan-2-yl)oxy)tetrahydro-2*H*-pyran-2-yl)methoxy)phosphoryl) bis(oxy)bis(ethane-2,1-diyl)dicarbamate (23) Starting with **17,** the alcohol used was *N*-Boc ethanolamine. The mixture was purified by flash chromatography and eluted with PE/EtOAc 6:4. The product was confirmed to be present by ^{1}H, albeit heavily contaminated with *N*-Boc ethanolamine, which co-elutes with the intended product. The mixture was used in the next reaction without further purification.

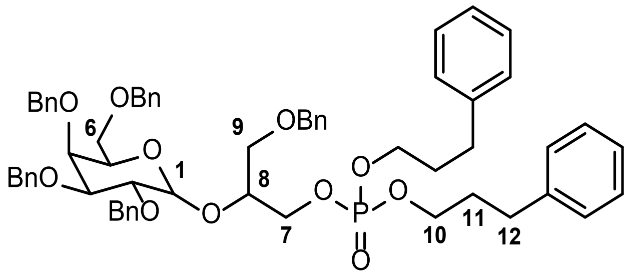

3-(Benzyloxy)-2-(((3*R*,4*S*,5*S*,6*R*)-3,4,5-*tris*(benzyloxy)-6-((benzyloxy)methyl)tetrahydro-2*H*-pyran-2-yl)oxy)propyl *bis*(3-phenylpropyl) phosphate (24) Starting with **16,** the alcohol used was 3-phenylpropan-1-ol. The crude mixture was eluted with PE/EtOAc 7:3. The product was obtained as a colourless viscous oil (α/β 1:10). ***β*-anomer:** ^{1}H NMR (400 MHz, $CDCl_3$) δ 7.40 – 7.05 (m, 35H, ArH), 5.15 (d, J = 3.7 Hz, 1H, H-1α), 5.00 – 4.89 (m, 2H, C<u>H</u>2Ar), 4.76 – 4.65 (m, 3H, C<u>H</u>2Ar), 4.64 – 4.44 (m, 4H, C<u>H</u>2Ar, H-1β), 4.43 – 4.31 (m, 2H, H-7), 4.23 – 3.93 (m, 7H, H-8, H-9, H-10), 3.92 – 3.85 (m, 1H, H-4), 3.84 – 3.72 (m, 2H, H-2, H-6a), 3.71 – 3.42 (m, 5H, H-3, H-5, H-6b, H-9), 2.77 – 2.54 (m, 4H, H-12), 2.08 – 1.81 (m, 4H, H-11). ^{13}C NMR (101 MHz, $CDCl_3$) δ 141.04, 140.99, 140.97, 140.91, 140.71, 138.87, 138.82, 138.71, 138.65, 138.59, 138.53, 138.49, 138.16, 138.08, 137.99, 137.93, 137.90, 137.88, 128.54, 128.49, 128.46, 128.38, 128.35, 128.29, 128.22, 128.17, 128.07, 127.91, 127.88, 127.85, 127.82, 127.78, 127.70, 127.65, 127.63, 127.60, 127.56, 127.45, 127.40, 126.17, 126.12, 126.08, 126.04, 125.89, 103.94, 103.54, 97.55 (C-1α), 82.17, 82.13, 79.46, 79.42, 78.87, 76.51, 76.43, 76.37, 75.11, 75.06, 74.80, 74.68, 74.60, 73.57, 73.52, 73.48, 73.43, 73.38, 73.33, 73.23, 73.16, 73.10, 72.89, 69.58, 69.37, 69.05, 68.81, 68.59, 68.46, 68.40, 67.13, 67.08, 67.03, 66.90,

66.84, 34.26, 32.11, 31.88, 31.81, 31.73, 31.68, 31.64, 31.62, 31.51, 29.73. HRMS-ESI: Calculated for [$C_{62}H_{69}O_{11}P+Na^+$] 1043.4470; found 1043.4459.

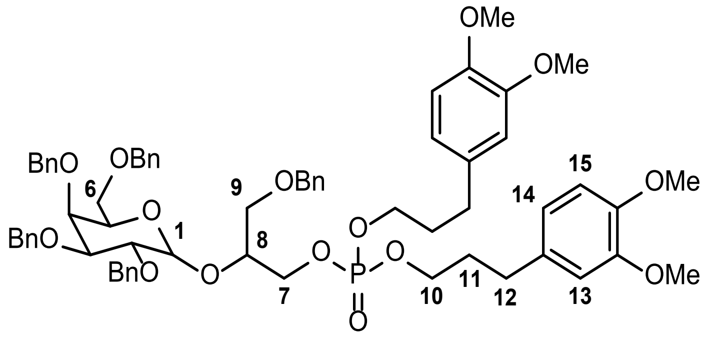

3-(Benzyloxy)-2-(((3*R*,4*S*,5*S*,6*R*)-3,4,5-*tris*(benzyloxy)-6-((benzyloxy)methyl)tetrahydro-2*H*-pyran-2-yl)oxy)propyl *bis*(3-(3,4-dimethoxyphenyl)propyl) phosphate (25) The alcohol used was 3-(3,4-dimethoxyphenyl)propan-1-ol. The crude mixture was eluted with PE/EtOAc 7:3. The product was obtained as colourless viscous oil (α/β 1:1), contaminated with the tri-substituted phosphate of the alcohol used. ^{1}H NMR (400 MHz, $CDCl_3$) δ 7.42 – 7.17 (m, 50H, ArH), 6.84 – 6.62 (m, 12H, H-13, H-14, H-15), 5.20 – 5.12 (m, 1H, H-1α), 4.97 – 4.87 (m, 3H, C<u>H</u>$_2$Ar), 4.82 – 4.64 (m, 7H), 4.61 – 4.29 (m, 11H, H-1β), 4.25 – 3.99 (m, 24H, H-2α, H-10), 3.96 – 3.72 (m, 40H, H-2β, -OMe), 3.71 – 3.42 (m, 12H), 2.79 – 2.54 (m, 8H, H-12), 2.10 – 1.85 (m, 8H, H-11). Nuclide count is doubled to account for both anomers. ^{13}C NMR (101 MHz, $CDCl_3$) δ 170.95, 148.73, 148.69, 147.20, 147.15, 140.70, 138.63, 138.45, 138.38, 138.25, 137.94, 137.81, 137.68, 133.30, 133.28, 133.06, 128.29, 128.23, 128.21, 128.15, 128.13, 128.10, 128.05, 128.03, 128.00, 127.96, 127.83, 127.66, 127.62, 127.60, 127.54, 127.48, 127.45, 127.41, 127.38, 127.35, 127.32, 127.24, 127.16, 127.13, 125.91, 120.07, 120.04, 111.58, 111.54, 111.51, 111.10, 111.08, 97.38, 96.94, 81.98, 79.20, 78.67, 77.55, 76.10, 74.86, 74.77, 74.58, 74.37, 73.28, 73.21, 73.01, 72.92, 72.83, 72.65, 69.38, 68.82, 68.22, 66.86, 66.75, 66.69, 60.19, 55.72, 55.70, 55.64, 55.62, 31.95, 31.88, 31.75, 31.69, 31.47, 31.05, 31.02, 30.90, 20.85, 14.00. HRMS-ESI: Calculated for [$C_{66}H_{77}O_{15}P+Na^+$] 1163.4892; found 1163.4875.

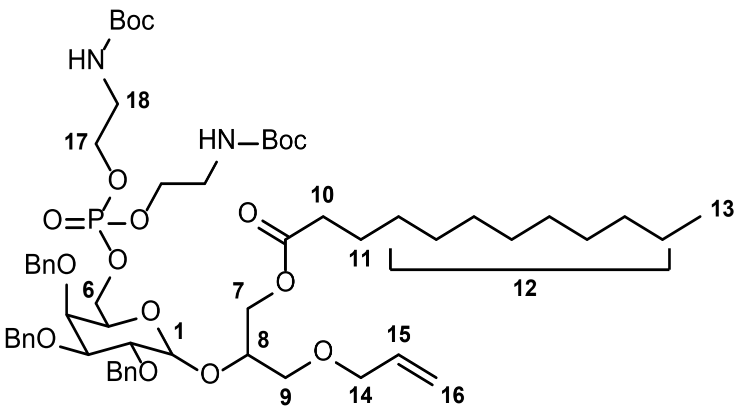

3-(Allyloxy)-2-(((3*R*,4*S*,5*S*,6*R*)-3,4,5-*tris*(benzyloxy)-6-(((*bis*(2-((tert-butoxycarbonyl)amino)ethoxy)phosphoryl)oxy)methyl)tetrahydro-2*H*-pyran-2-yl)oxy)propyl dodecanoate

(26) Starting with **18,** the alcohol used was *N*-Boc ethanolamine. The product was eluted with PE/EtOAc 8:2. The product was obtained contaminated with *N*-Boc ethanolamine, and the mixture appeared as colourless oil. The product was used without further purification (*α*/*β* 1:1). ***β*-anomer:** ^{1}H NMR (400 MHz, $CDCl_3$) δ 7.40 – 7.25 (m, 15H, ArH), 5.93 – 5.76 (m, 1H, H-15), 5.28 – 5.10 (m, 3H, H-16, NH), 5.02 – 4.95 (m, 1H, C<u>H</u>$_2$Ar), 4.93 (d, *J* = 10.7 Hz, 1H, C<u>H</u>$_2$Ar), 4.80 (d, *J* = 11.8 Hz, 1H, C<u>H</u>$_2$Ar), 4.77 – 4.69 (m, 2H, C<u>H</u>$_2$Ar), 4.61 (d, *J* = 11.5 Hz, 1H, C<u>H</u>$_2$Ar), 4.56 – 4.49 (m, 1H, H-1), 4.35 – 4.28 (m, 1H, H-9a), 4.21 (dd, *J* = 12.0, 6.0 Hz, 1H, H-9b), 4.18 – 3.92 (m, 9H, H-6, H-8, H-14, H-17), 3.84 – 3.77 (m, 2H, H-2, H-4), 3.72 – 3.67 (m, 1H, H-7a), 3.62 – 3.49 (m, 3H, H-3, H-5, H-7b), 3.42 – 3.27 (m, 4H, H-18), 2.30 (t, *J* = 7.6 Hz, 1H, H-10a), 2.19 – 2.12 (m, 1H, H-10b), 1.63 – 1.48 (m, 2H, H-11), 1.44 (m, 18H, *tert*-butyl), 1.35 – 1.18 (m, 16H, H-12), 0.88 (t, *J* = 6.7 Hz, 3H, H-13). ^{13}C NMR (101 MHz, $CDCl_3$) δ 173.74, 173.65, 155.84, 138.80, 138.73, 138.36, 138.32, 138.26, 134.55, 134.51, 128.43, 128.34, 128.24, 128.07, 127.89, 127.77, 127.70, 127.62, 127.51, 127.49, 117.17, 117.10, 103.91, 103.65, 81.88, 79.72, 79.19, 79.14, 77.24, 76.56, 76.34, 75.02, 74.98, 74.48, 73.53, 73.49, 73.24, 72.97, 72.35, 69.66, 69.61, 67.24, 67.18, 66.36, 66.31, 64.02, 63.82, 59.54, 40.90, 38.16, 34.23, 34.08, 31.94, 31.92, 31.26, 29.71, 29.67, 29.63, 29.51, 29.49, 29.38, 29.35, 29.31, 29.30, 29.19, 29.14, 28.40, 24.92, 24.83, 22.70. HRMS-ESI: Calculated for [$C_{59}H_{89}N_2O_{16}P+Na^+$]: 1135.5842; found 1135.5824.

General procedure for hydrogenation. The starting material was dissolved in dry EtOH 0.1 M, and 5% of Pd/C (10% w/w) was added. The reaction was conducted in a Parr Shaker 3900 series hydrogenation apparatus, under a 3 bar H_2 atmosphere and stirring. The system was purged 3 times with H_2 before the start. Completion was followed by TLC and ^{1}H NMR. After completion, the solvent was evaporated. If product **1** is the final product of hydrogenation, then **1** was purified by reverse chromatography and eluted with a gradient of water/MeOH in 10% increments.

General procedure for Boc removal. To this, 5 mL of DCM/TFA 1:1 was added, and the reaction was followed by TLC with ninhydrin staining. After 1h, the reaction was complete. The solvents were evaporated. The final product **1** was purified by reverse chromatography and eluted with a gradient of water/MeOH in 10% increments.

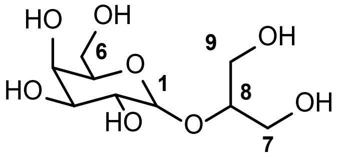

2-*O*-D-(galactopyranosyl)glycerol (1a) Galactoglycerol **16** was subjected to hydrogenation conditions. The product **1a** was obtained as a yellowish, highly hygroscopic oil (*α*/*β* 3:1). ^{1}H NMR (400 MHz, MeOD) δ 5.03 (d,

J = 3.2 Hz, 1H, H-1α), 4.38 (d, J = 7.6 Hz, 1H, H-1β), 4.04 – 3.99 (m, 1H), 3.90 – 3.88 (m, 1H), 3.85 – 3.64 (m, 16H), 3.61 – 3.47 (m, 4H). HRMS-ESI: Calculated for [$C_9H_{18}O_8$+Na^+] 277.0894; found 277.0891.

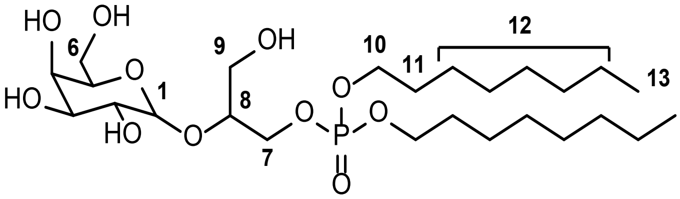

3-Hydroxy-2-galactopyranosylpropyl dioctyl phosphate (1b) Starting material **21** was subjected to hydrogenation conditions. The product **1b** was obtained as a colourless oil (α/β 1:1). ^{1}H NMR (400 MHz, MeOD) δ 5.07 – 5.00 (m, 1H, H-1α), 4.38 (d, J = 7.5 Hz, 1H, H-1β), 4.25 – 4.14 (m, 4H, H-6), 4.13 – 4.04 (m, 9H, H-4, H-10), 4.04 – 3.94 (m, 3H, H-4, H-8), 3.93 – 3.86 (m, 2H, H-5), 3.84 – 3.63 (m, 9H, H-2α, H-7, H-9), 3.57 – 3.50 (m, 1H, H-2β), 3.50 – 3.43 (m, 2H, H-3α), 1.75 – 1.65 (m, 8H, H-11), 1.38 – 1.20 (m, 40H, H-12), 0.93 – 0.86 (m, 12H, H-13). ^{13}C NMR (101 MHz, MeOD) δ 104.99, 100.60, 76.88, 74.81, 72.48, 71.49, 71.08, 70.23, 69.57, 62.82, 62.53, 40.43, 32.97, 31.37, 31.30, 30.75, 30.35, 30.23, 26.61, 23.71, 14.43. HRMS-ESI: Calculated for [$C_{25}H_{51}O_{11}P$+Na^+] 581.3061; found 581.3055.

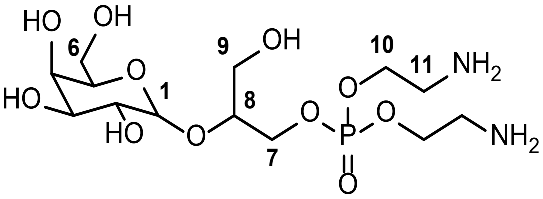

3-Hydroxy-2-galactopyranosylpropyl (*bis*(2-aminoethyl)) phosphate (1c) Starting material **22** was subjected to hydrogenation conditions and then Boc removal conditions. The product **1c** was obtained as an orange viscous oil (α/β 1:1). ^{1}H NMR (400 MHz, D_2O) δ 5.21 – 5.17 (m, 1H, H-1), 4.49 – 4.42 (m, 5H, H-6a, H-10), 4.38 – 4.31 (m, 1H, H-6b), 4.10 – 4.04 (m, 2H, H-4, H-8), 4.03 – 3.99 (m, 1H, H-5), 3.93 – 3.84 (m, 2H, H-2, H-3), 3.84 – 3.73 (m,

4H, H-7, H-9), 3.43 – 3.38 (m, 4H, H-11) ^{13}C NMR (101 MHz, D_2O) δ 98.83, 76.54, 76.47, 71.94, 69.95, 69.84, 68.88, 68.00, 67.94, 65.52, 65.47, 61.81, 61.36, 40.12, 40.04. LCMS: 421 $[M+H]^+$, 443 $[M+Na]^+$.

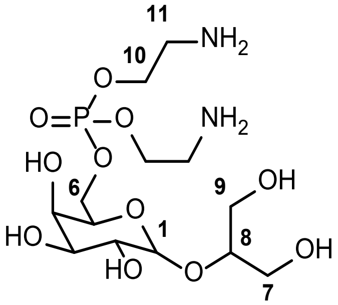

***Bis*(2-Aminoethyl) (galactopyranosyl)glycerol)-6-phosphate (1d)** Starting material **23** was subjected to hydrogenation conditions and then Boc removal conditions. The product **1d** was obtained as an orange viscous oil. Only the α-anomer was recovered. ***α*-anomer:** ^{1}H NMR (400 MHz, MeOD) δ 5.08 (d, J = 3.5 Hz, 1H, H-1), 4.25 – 4.10 (m, 5H, H-5, H-10), 4.07 – 4.00 (m, 2H, H-6), 3.99 – 3.95 (m, 1H, H-4), 3.87 – 3.62 (m, 7H, H-2, H-7, H-8, H-9), 3.28 – 3.17 (m, 4H, H-11). ^{13}C NMR (101 MHz, MeOD) δ 97.91, 78.83, 69.00, 68.92, 64.20, 61.36, 61.07, 60.63, 59.87, 39.28, 39.22. HRMS: Calculated for $[C_{11}H_{24}O_{11}NP - C_2H_6N]^-$: 376.1014; found 376.1009. Calculated for $[C_{11}H_{24}O_{11}NP + Na^+ - C_2H_5N]$: 400.0985; found 400.0974.

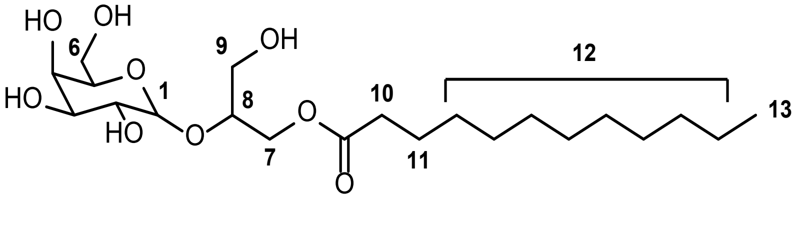

3-Hydroxy-2-(galactopyranosyl)propyl dodecanoate (1e) Starting material **19** was subjected to hydrogenation conditions. The product **1e** was obtained as a colourless viscous oil (α/β 1:1). The ^{1}H NMR signals of the diastereomers were assigned according to the anomers α and β. ^{1}H NMR (400 MHz, MeOD) δ 5.09 – 5.02 (m, 2H, H-1α), 4.43 – 4.38 (m, 2H, H-1β), 4.34 – 4.17 (m, 4H, H-7), 4.05 – 3.89 (m, 4H, H-4, H-8), 3.87 – 3.62 (m, 10H, H-2α, H-3α, H-6, H-9), 3.60 – 3.46 (m, 4H, H-2β, H-3β, H-5), 2.43 – 2.32 (m, 4H, H-10), 1.70 – 1.57 (m, 4H, H-11), 1.41 – 1.18 (m, 32H, H-12), 0.96 – 0.87 (m, 6H, H-13). ^{13}C NMR (101 MHz, MeOD) δ 174.04, 174.01, 103.72, 103.34, 98.96, 98.75, 78.12, 77.74, 76.79, 76.11, 75.43, 75.36, 73.46, 71.34, 71.15, 71.08, 70.03, 69.71, 69.58, 68.88, 68.77, 63.56,

63.34, 63.20, 62.95, 61.93, 61.59, 61.42, 61.08, 60.59, 33.56, 31.67, 29.34, 29.22, 29.07, 29.03, 28.83, 24.62, 24.57, 22.33, 13.04. HRMS-ESI: Calculated for [$C_{21}H_{40}O_9P+Na^+$] 459.2564; found 459.2558.

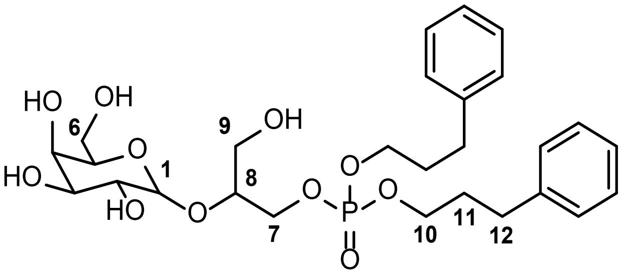

3-Hydroxy-2-(galactopyranosyl)propyl *bis*(3-phenylpropyl) phosphate (1f) Starting material **24** was subjected to hydrogenation conditions. The product **1f** was obtained as a colourless viscous oil (α/β 1:10). ***β*-anomer:** ^{1}H NMR (400 MHz, MeOD) δ 7.32 – 7.13 (m, 10H, ArH), 4.42 – 4.35 (m, 1H, H-1), 4.27 – 4.18 (m, 2H, H-6), 4.14 – 4.05 (m, 4H, H-10), 4.01 – 3.93 (m, 1H, H-5), 3.84 – 3.80 (m, 1H, H-8), 3.78 – 3.66 (m, 4H, H-3, H-4, H-7), 3.59 – 3.43 (m, 3H, H-2, H-9), 2.73 (t, J = 7.6 Hz, 4H, H-12), 2.05 – 1.96 (m, 4H, H-11). ^{13}C NMR (101 MHz, MeOD) δ 140.93, 129.17, 128.14, 128.12, 125.71, 103.74, 77.88, 71.08, 67.29, 31.68, 31.61, 31.20, 29.36. HRMS-ESI: Calculated for [$C_{27}H_{39}O_{11}P+Cl^-$]: 605.1924; found 605.1925.

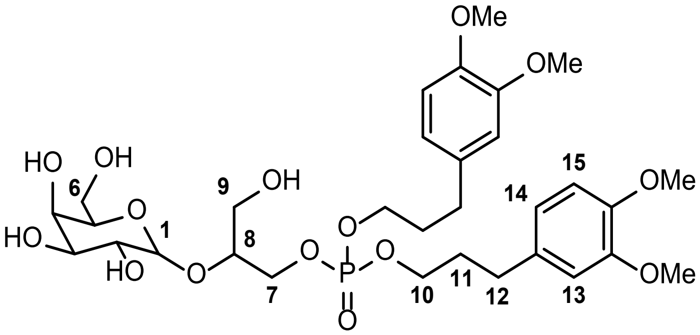

3-Hydroxy-2-(galactopyranosyl)propyl (*bis*(3-(3,4-dimethoxyphenyl)propyl) phosphate (1g) Starting material **25** was subjected to hydrogenation conditions. The product was obtained as a colourless viscous oil and a mixture of 4 diastereomers (α/β 1:0.75). ^{1}H NMR (400 MHz, MeOD) δ 6.86 – 6.70 (m, 12H, ArH), 5.05 – 5.01 (m, 1H, H-1α), 4.39 – 4.34 (m, 1H, H-1β), 4.28 – 4.19 (m, 4H, H-6), 4.13 – 4.03 (m, 8H, H-10), 4.00 – 3.93 (m, 2H), 3.92 – 3.85 (m, 4H), 3.85 – 3.63 (m, 33H, -OCH_3, H-2α, H-7, H-9), 3.58 – 3.42 (m, 4H, H-2β), 2.66 (d, J = 3.8 Hz, 8H, H-11), 2.06 – 1.94 (m, 8H, H-12). ^{13}C NMR (101 MHz, MeOD) δ 135.34, 135.32, 129.60, 121.88, 113.69, 113.32, 105.08,

100.69, 76.93, 74.90, 72.94, 72.56, 71.58, 71.16, 70.43, 68.82, 62.92, 62.75, 62.35, 56.63, 56.55, 33.20, 33.13, 32.20. HRMS-ESI: Calculated for [$C_{31}H_{47}O_{15}P+Na^+$]: 713.2545; found 713.2531.

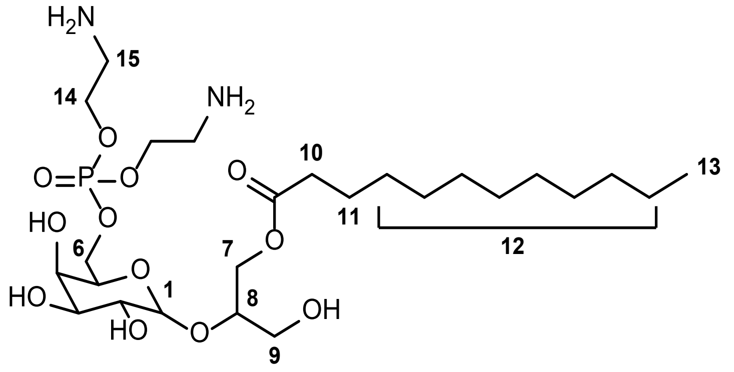

2-(6-(*bis*(2-Aminoethoxy))phosphoryl)galactopyranosyl-3-hydroxypropyl dodecanoate (1h) The starting material **26** (119 mg) was dissolved in 2 mL of DCM/MeOH 1:1. A catalytic amount of $PdCl_2$ (0.2 molar eq) was added, and the reaction proceeded at rt. After completion (followed by TLC), the reaction was quenched with a few drops of Et_3N, and the black precipitate formed was filtered. The orange solution obtained was washed with HCl 0.1M (2 x 10 mL), saturated $NaHCO_3$ (10 mL) and brine (10 mL). The organic phase was dried over Na_2SO_4 and the solvent evaporated. The resulting crude was subjected to hydrogenation and then Boc removal conditions. The product **1h** (23.2 mg, yield) was obtained as an orange viscous oil (α/β 1:2). ***α*-anomer:** ^{1}H NMR (400 MHz, MeOD) δ 5.13 – 5.07 (m, 1H, H-1), 4.49 – 4.15 (m, 10H, H-4, H-5, H-6, H-9, H-14), 4.04 – 3.83 (m, 2H, H-3, H-8), 3.83 – 3.64 (m, 3H, H-2, H-7), 3.39 – 3.27 (m, 4H, H-15), 2.41 – 2.33 (m, 2H, H-10), 1.69 – 1.58 (m, 2H, H-11), 1.41 – 1.25 (m, 16H, H-12), 0.92 (t, *J* = 6.7 Hz, 3H, H-13). ***β*-anomer:** ^{1}H NMR (400 MHz, MeOD) δ 4.49 – 4.15 (m, 10H, H-1, H-5, H-6, H-9, H-14), 4.04 – 3.83 (m, 2H, H-4, H-8), 3.83 – 3.64 (m, 2H, H-7), 3.60 – 3.52 (m, 2H, H-2, H-3), 3.39 – 3.27 (m, 4H, H-15), 2.41 – 2.33 (m, 2H, H-10), 1.69 – 1.58 (m, 2H, H-11), 1.41 – 1.25 (m, 16H, H-12), 0.92 (t, *J* = 6.7 Hz, 3H, H-13). ***αβ*-anomers:** ^{13}C NMR (101 MHz, MeOD) δ 175.48, 105.20, 104.90, 101.05, 100.22, 80.07, 79.85, 78.92, 77.76, 74.66, 74.58, 74.52, 74.44, 74.35, 72.44, 72.18, 70.86, 70.67, 70.07, 69.91, 69.33, 68.82, 65.87, 65.81, 64.66, 64.58, 64.44, 64.36, 63.07, 62.96, 62.42, 62.14, 40.86, 40.78, 35.06, 34.99, 34.95, 34.91, 33.04, 30.75, 30.72, 30.63, 30.60, 30.45, 30.41, 30.25, 30.23, 27.57, 27.56, 26.07, 26.00, 25.93, 23.71, 14.42. HRMS-ESI: Calculated for [$C_{23}H_{46}NO_{12}P+Na^+$]: 582.2650; found 582.2644.

2. NMR and mass spectra of compounds

Compound 6

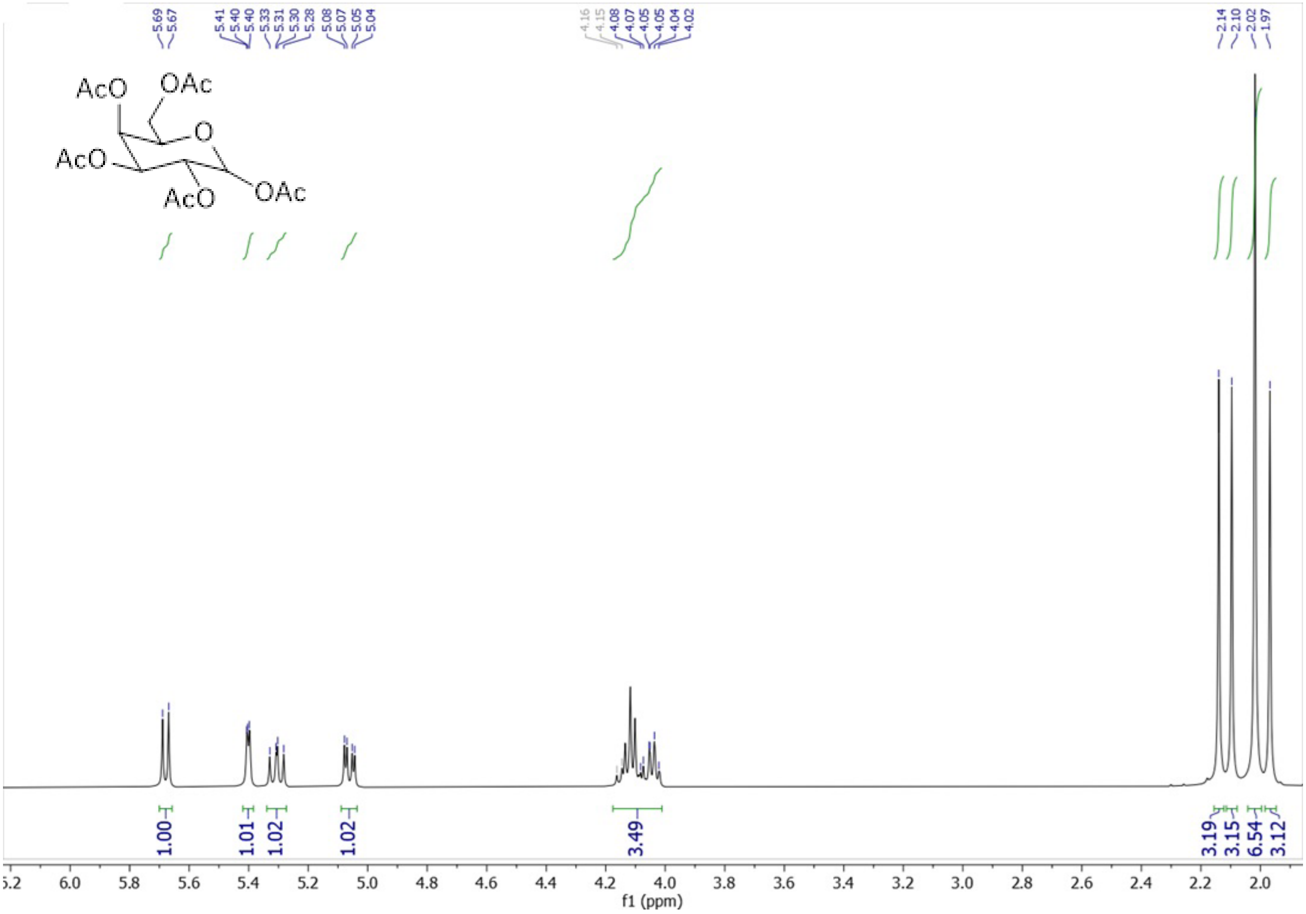

Figure S1 ^{1}H NMR ($CDCl_3$) spectrum of **6.**

Compound 7

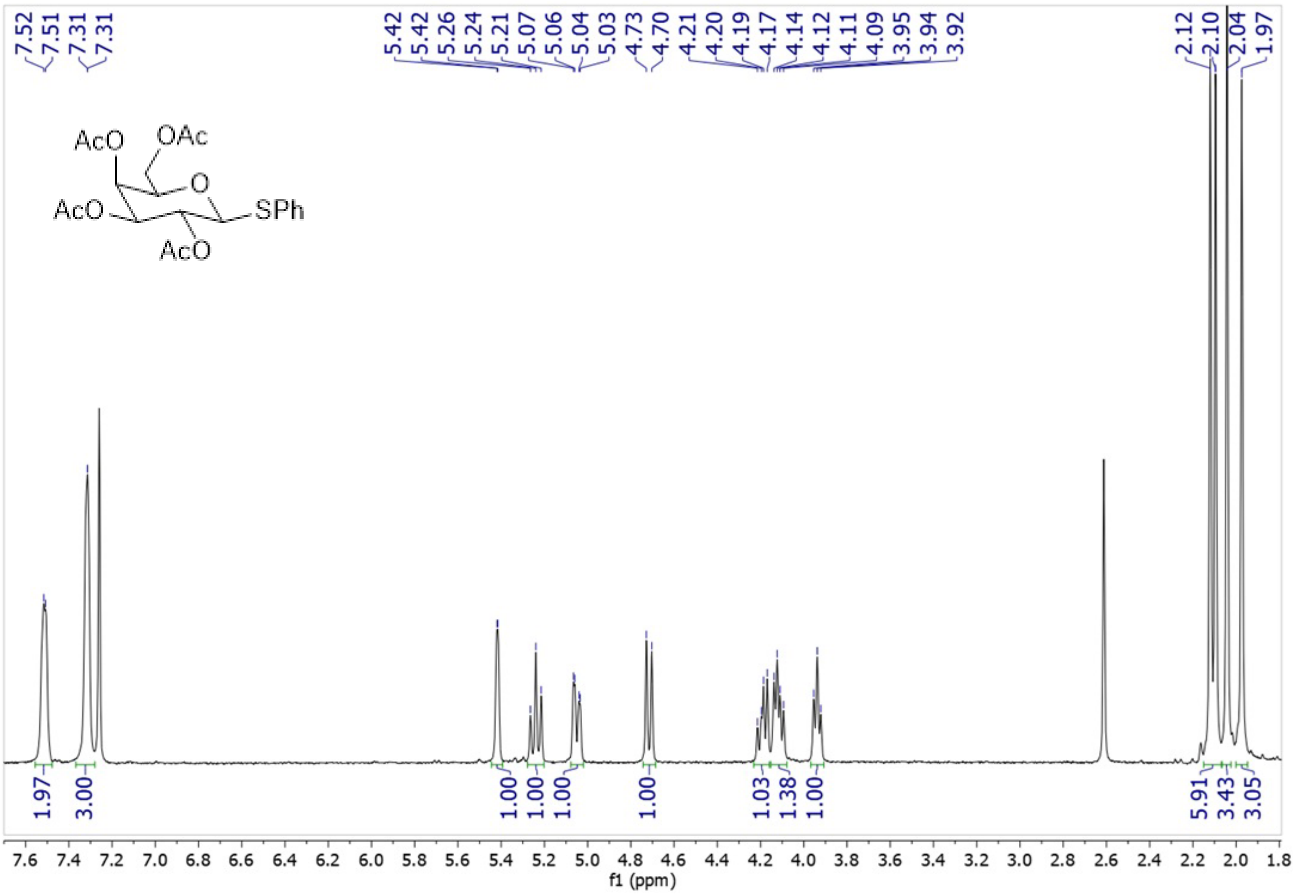

Figure S2 ^{1}H NMR ($CDCl_3$) spectrum of **7.**

Compound 8

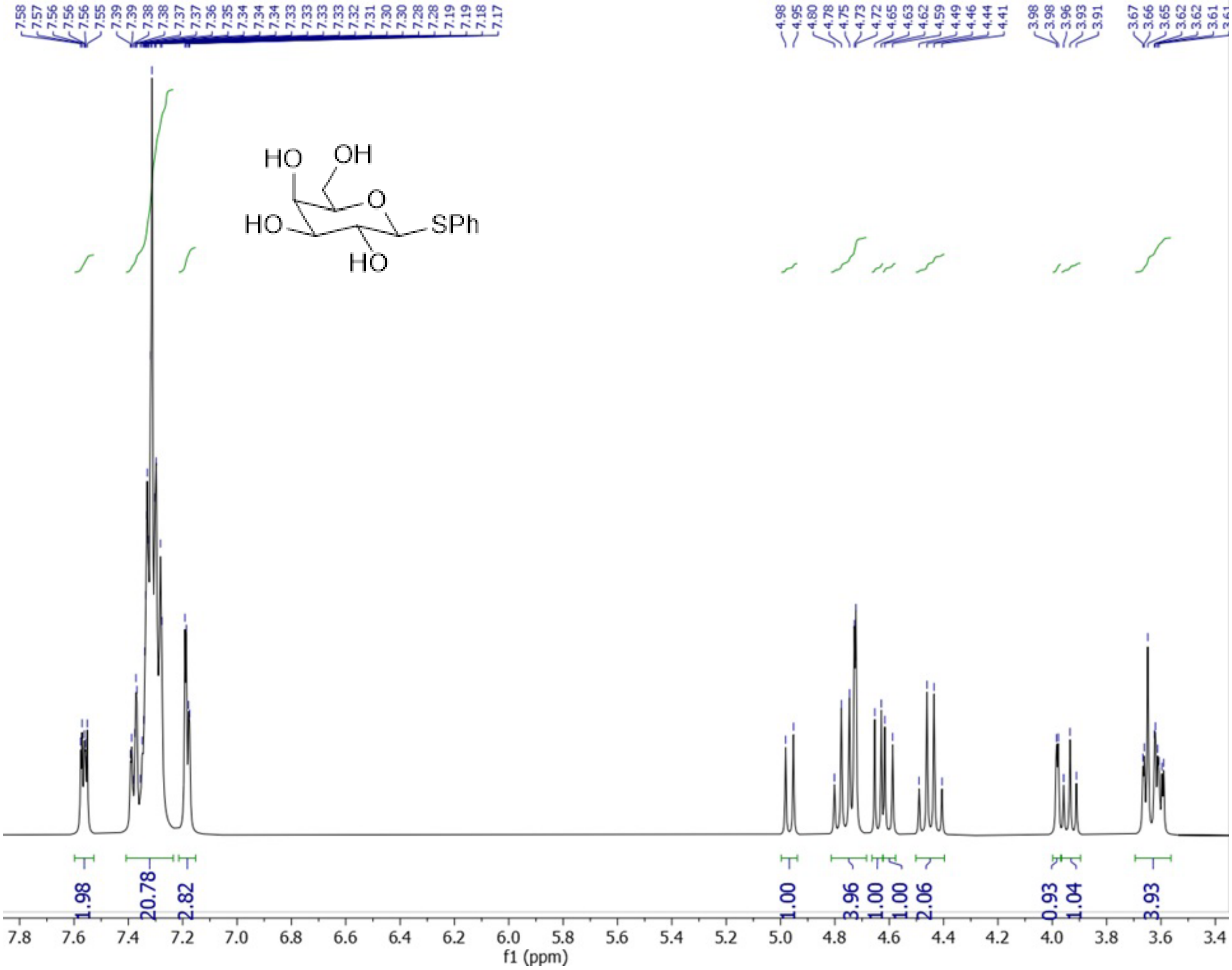

Figure S3 ^{1}H NMR ($CDCl_3$) spectrum of **8.**

Compound 9

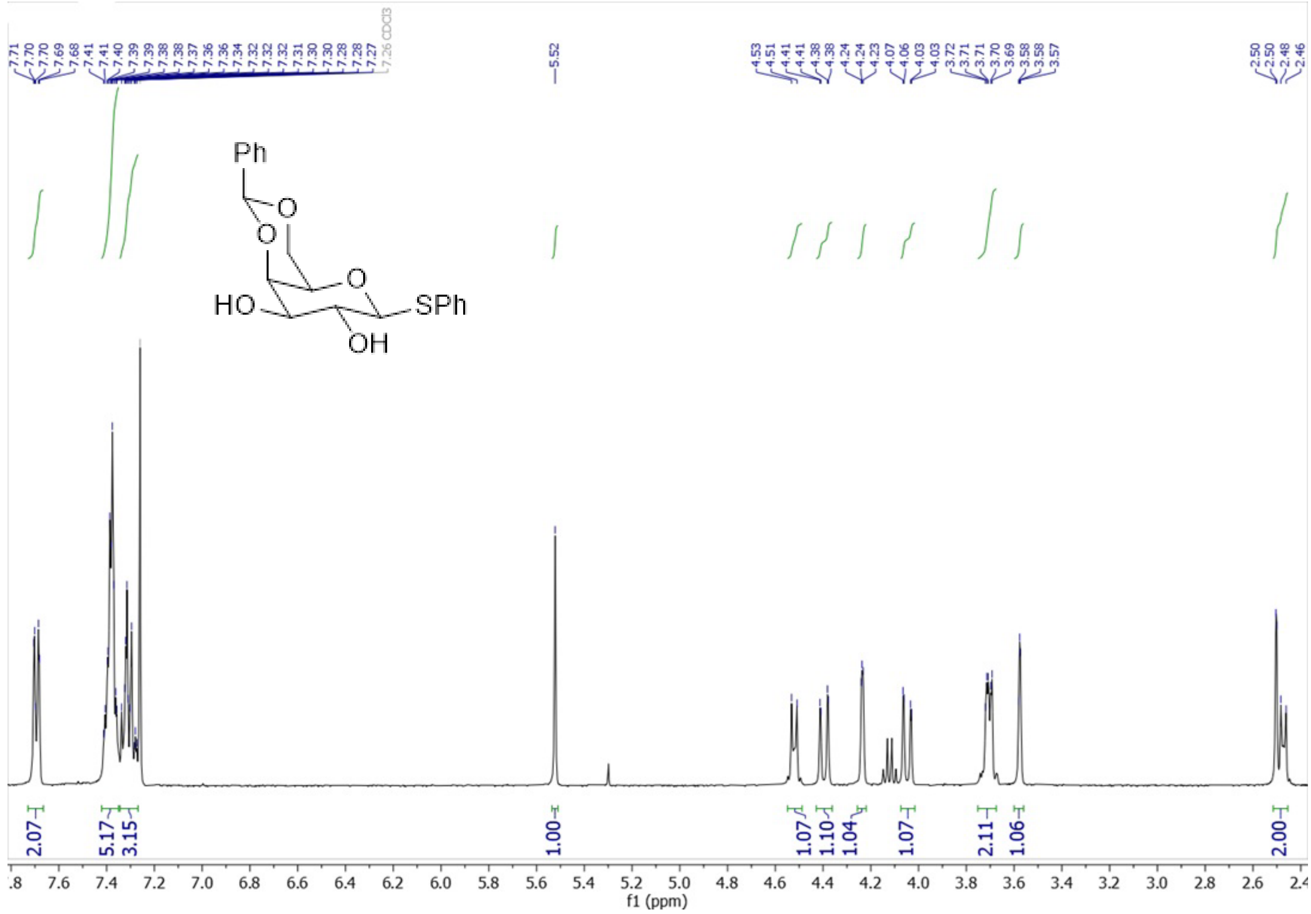

Figure S4 ^{1}H NMR ($CDCl_3$) spectrum of **9.**

Compound 10

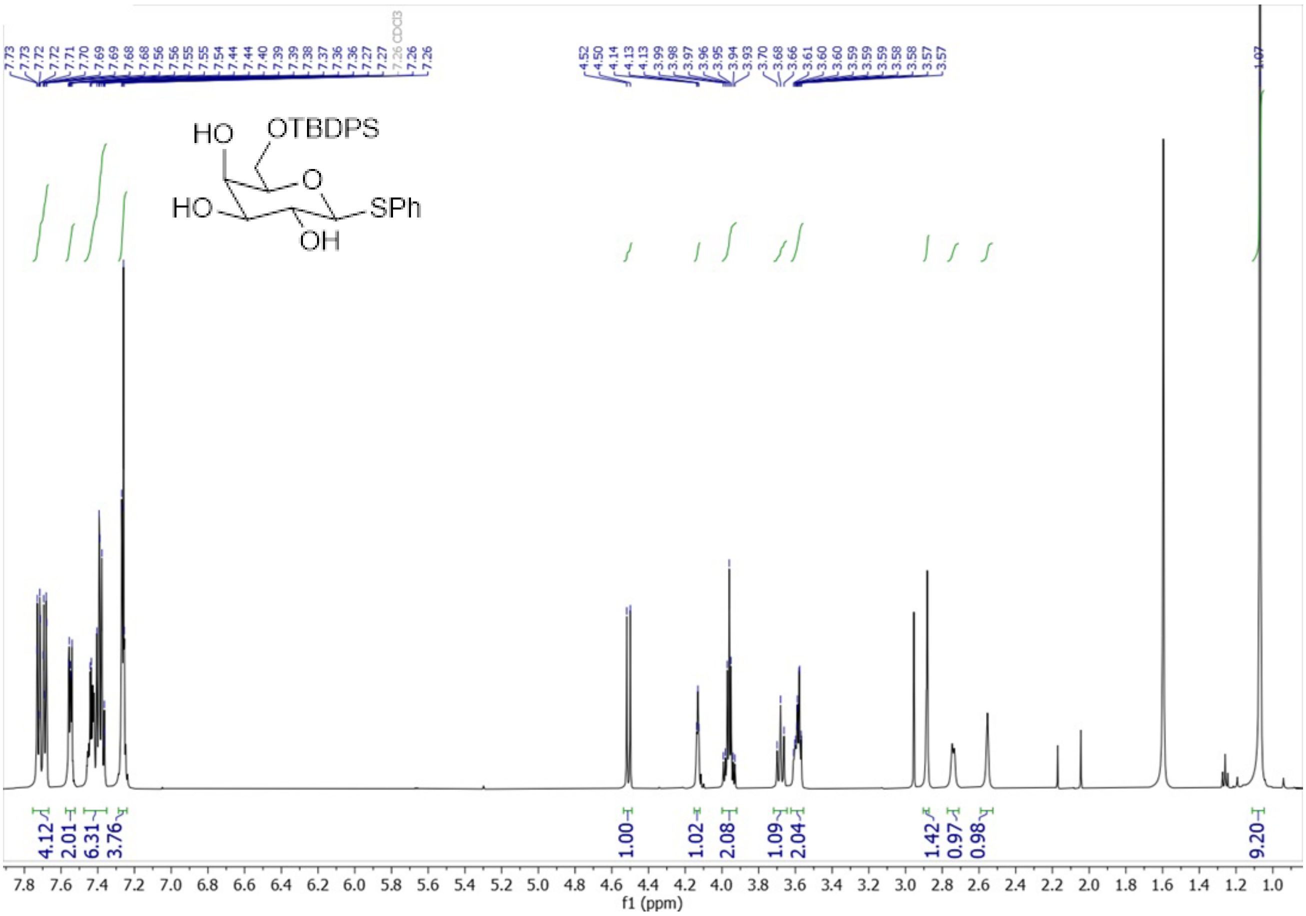

Figure S5 ^{1}H NMR ($CDCl_3$) spectrum of **10.**

Compound 11

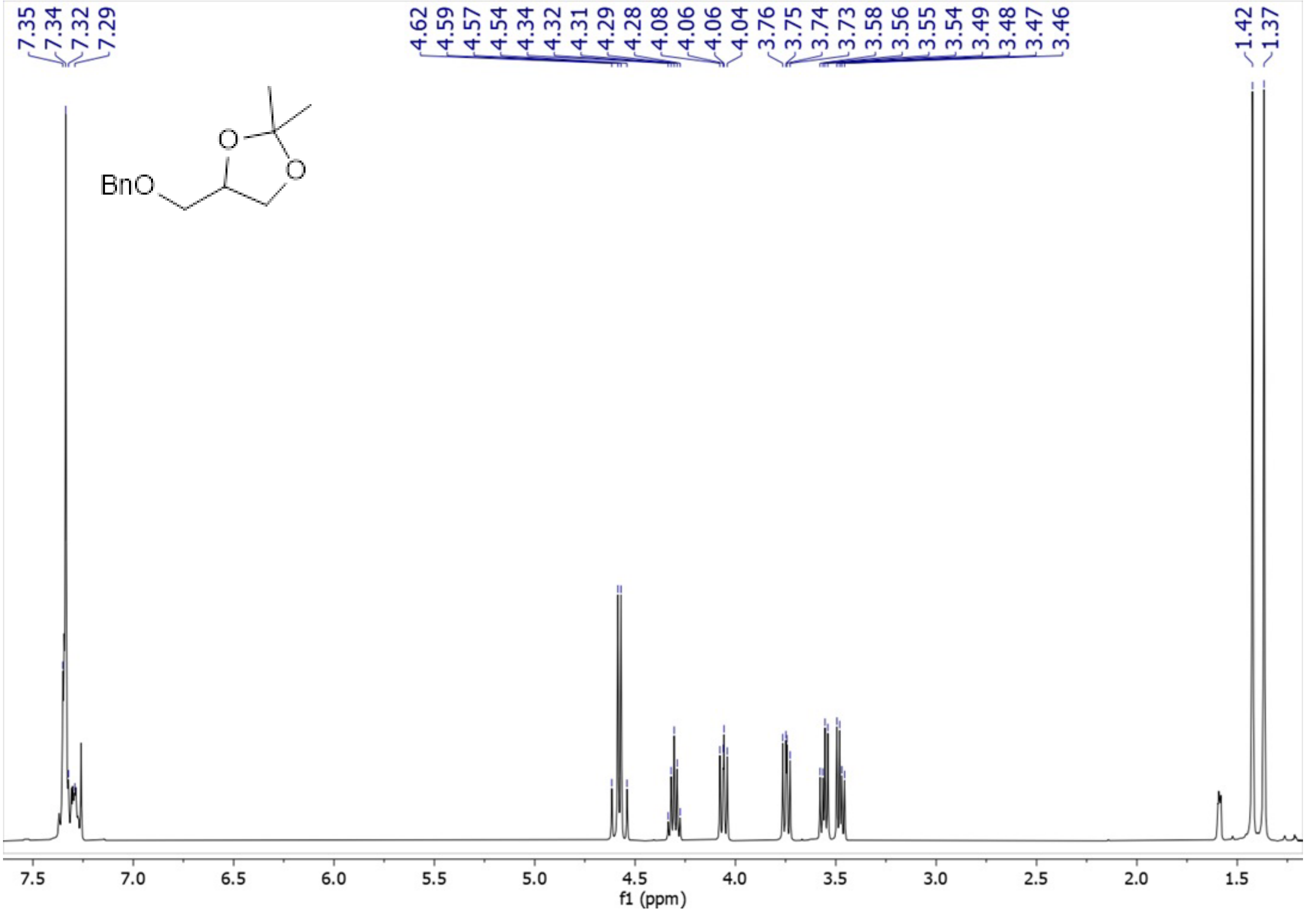

Figure S6 ^{1}H NMR ($CDCl_3$) spectrum of **11.**

Compound 12

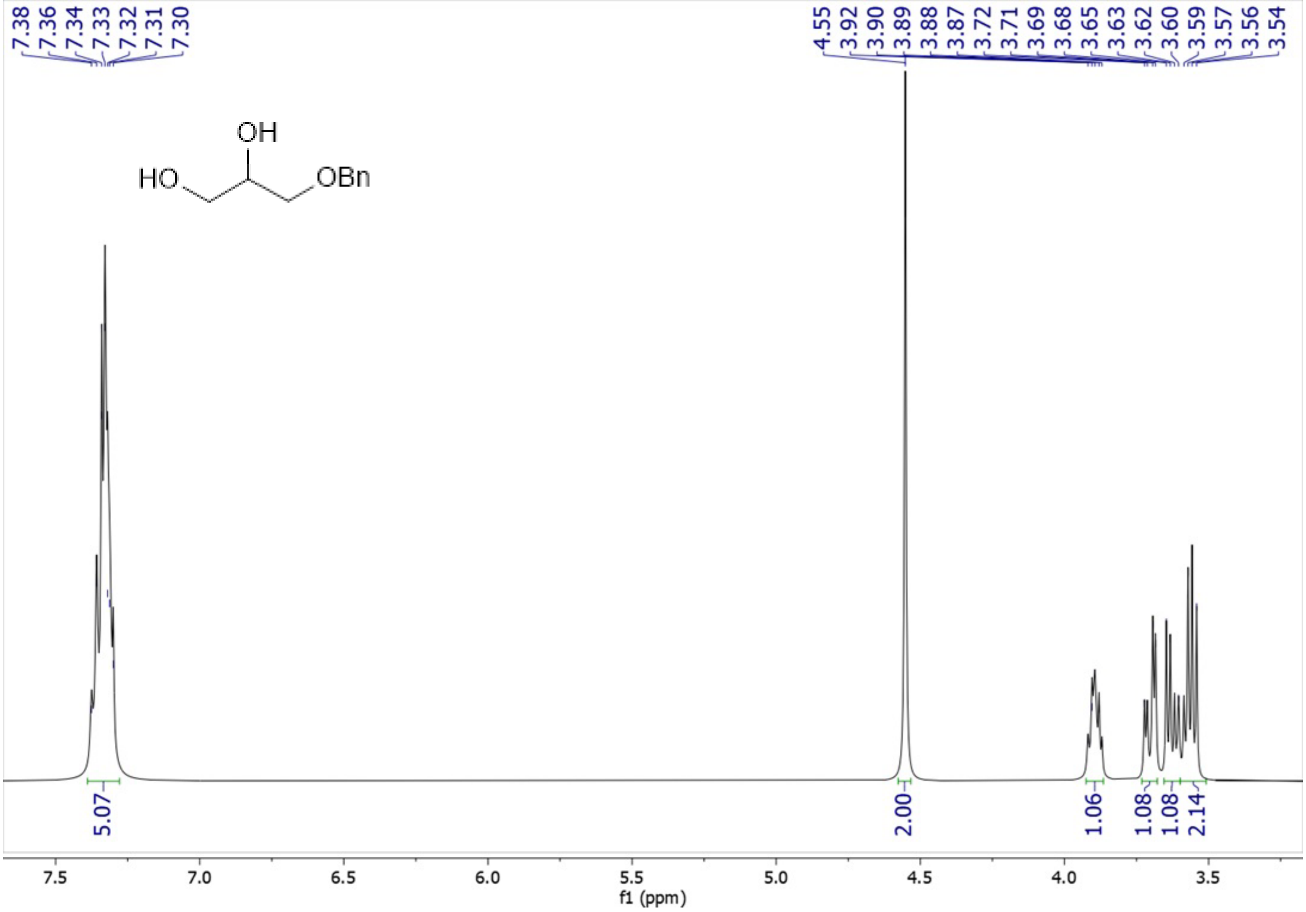

Figure S7 ^{1}H NMR ($CDCl_3$) spectrum of **12.**

Compound 14

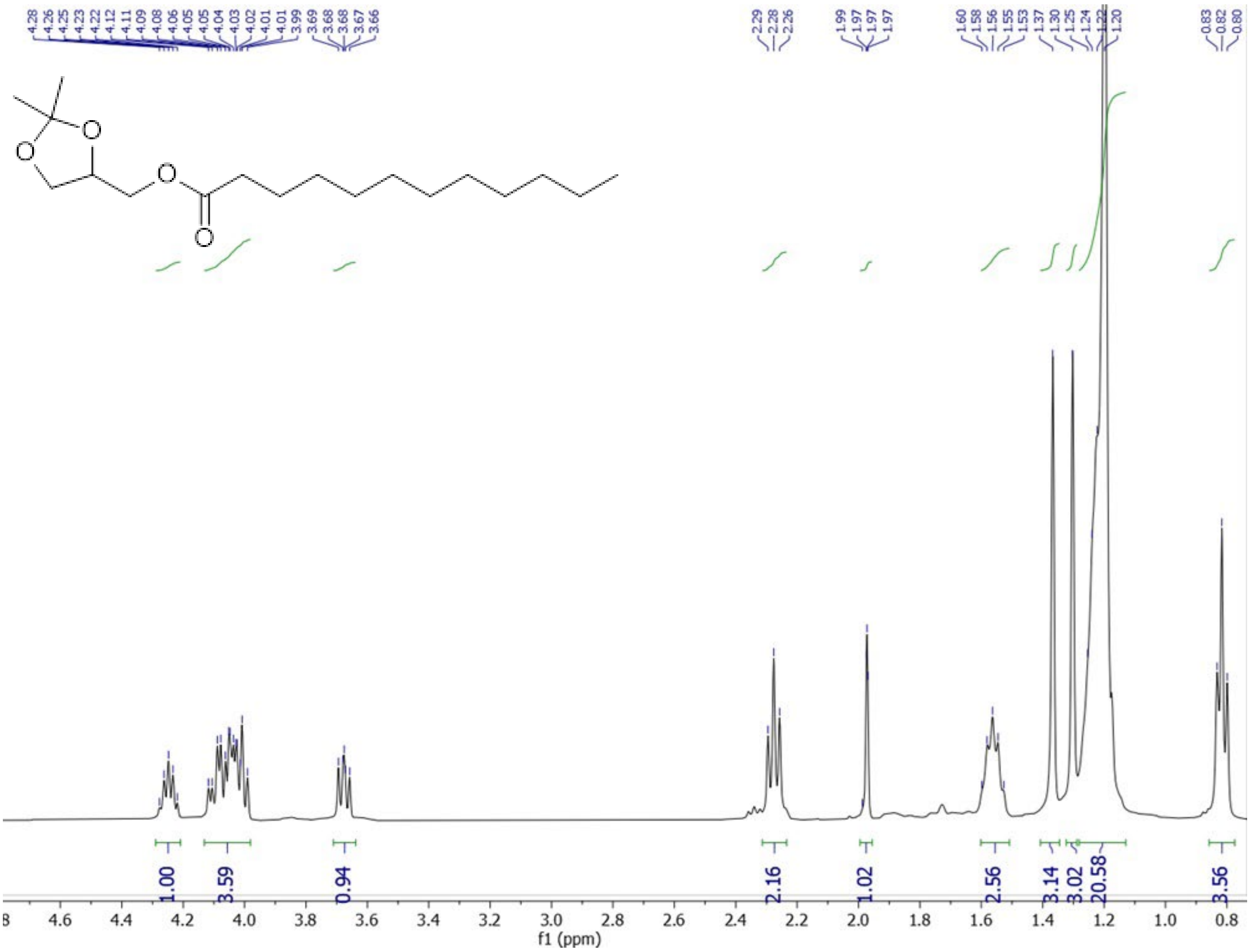

Figure S8 ^{1}H NMR ($CDCl_3$) spectrum of **14.**

Compound 15

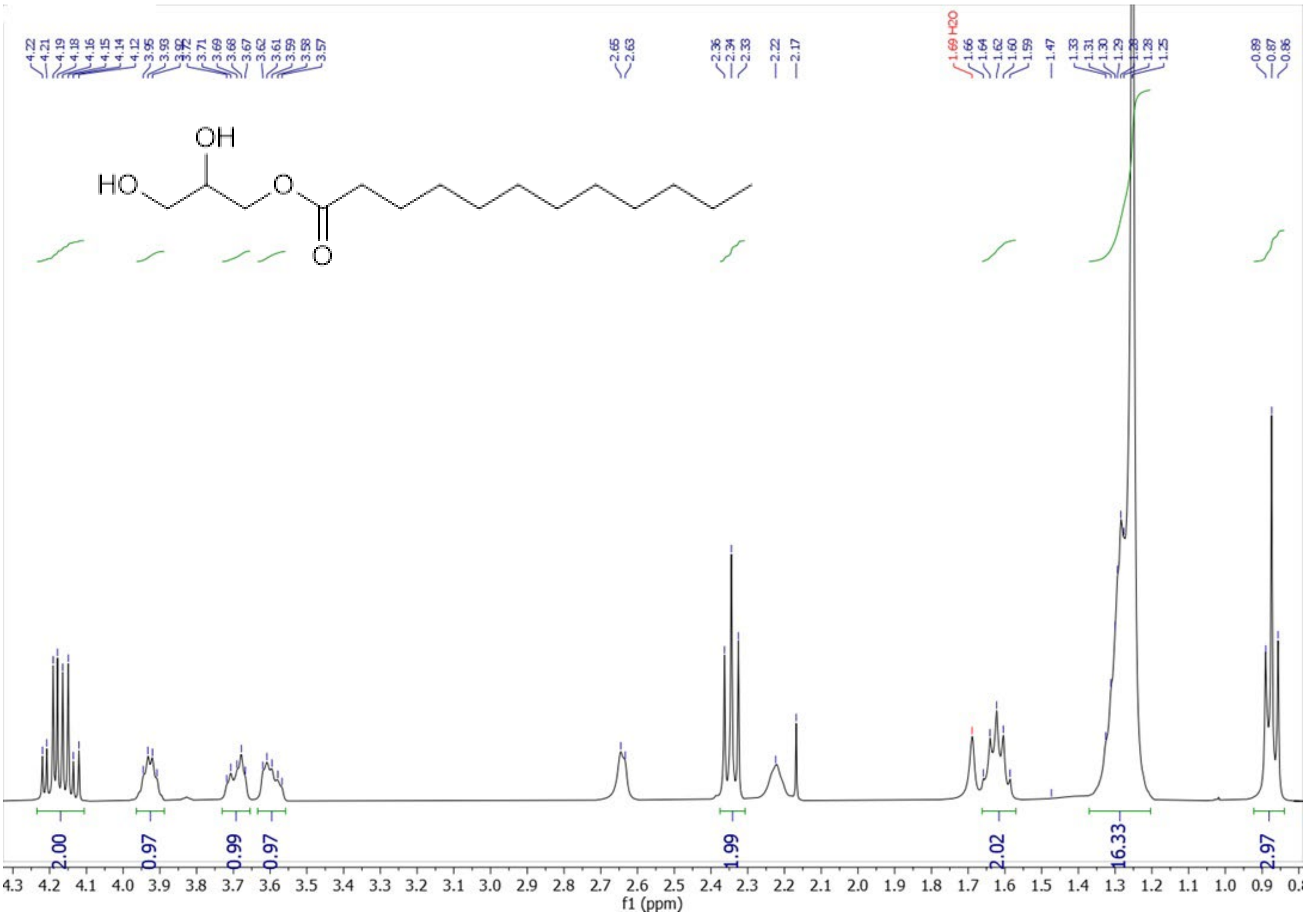

Figure S9 ^{1}H NMR ($CDCl_3$) spectrum of **15.**

Compound 2a

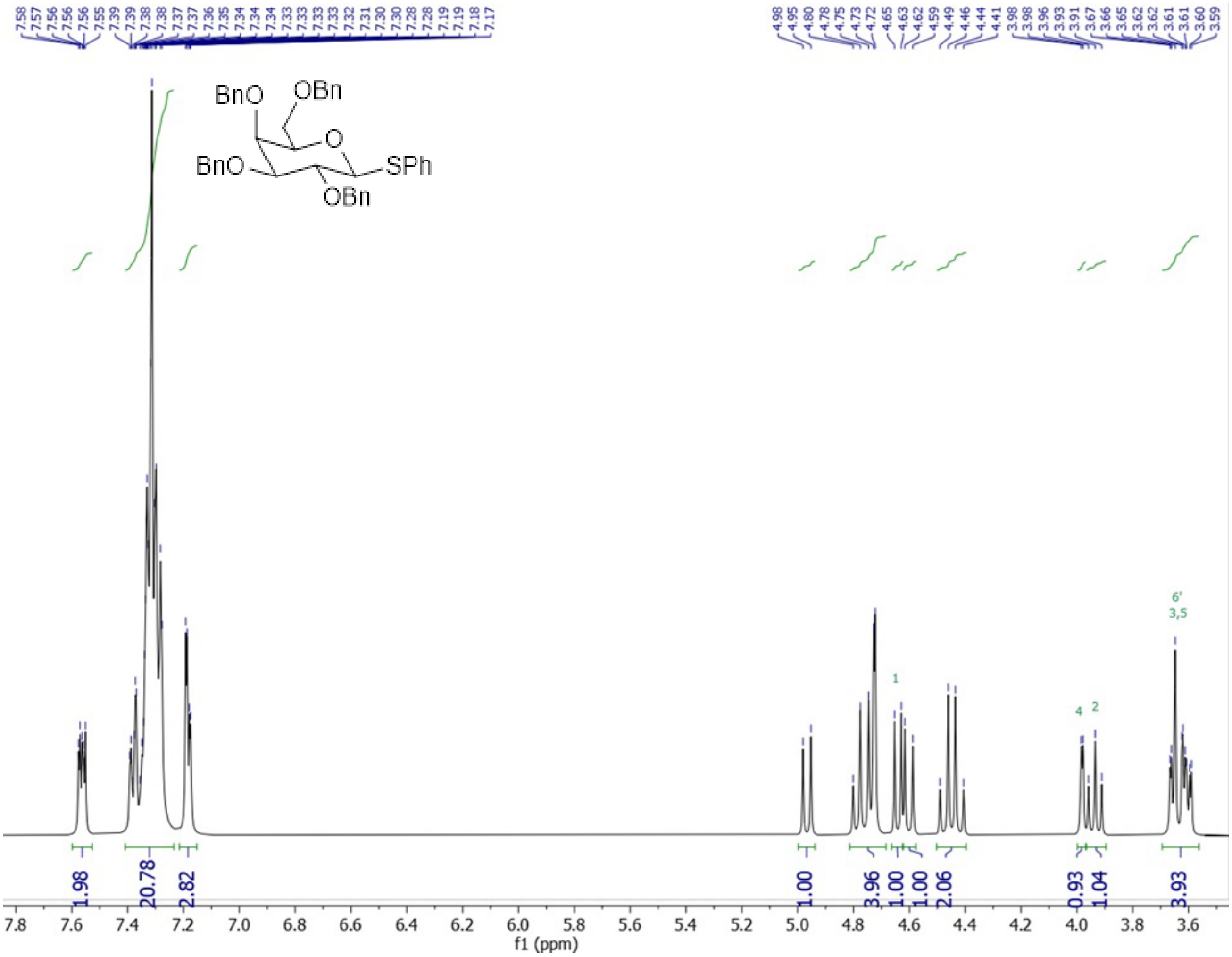

Figure S10 ^{1}H NMR ($CDCl_3$) spectrum of **2a.**

Compound 2b

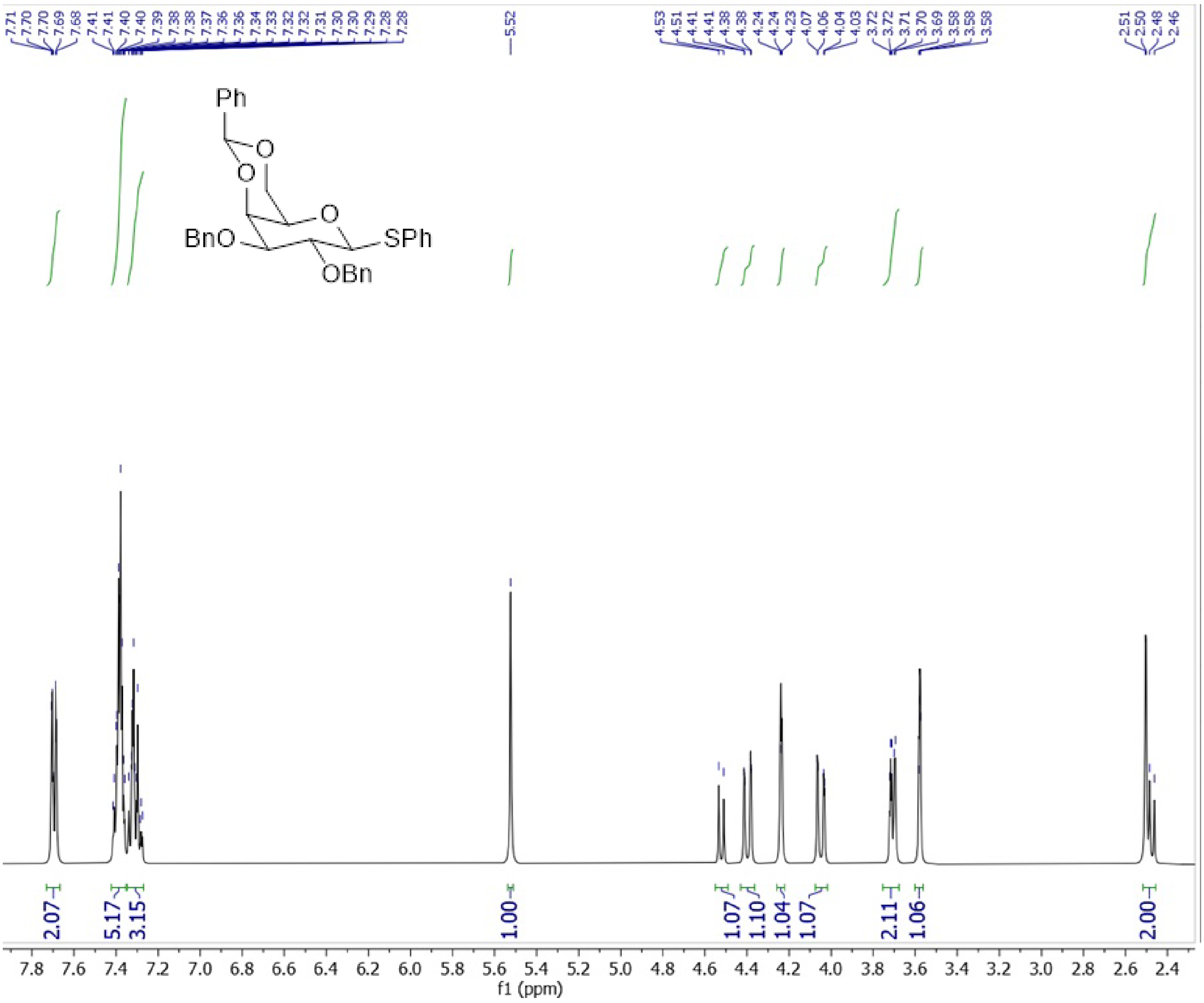

Figure S11 ^{1}H NMR ($CDCl_3$) spectrum of **2b.**

Compound 2c

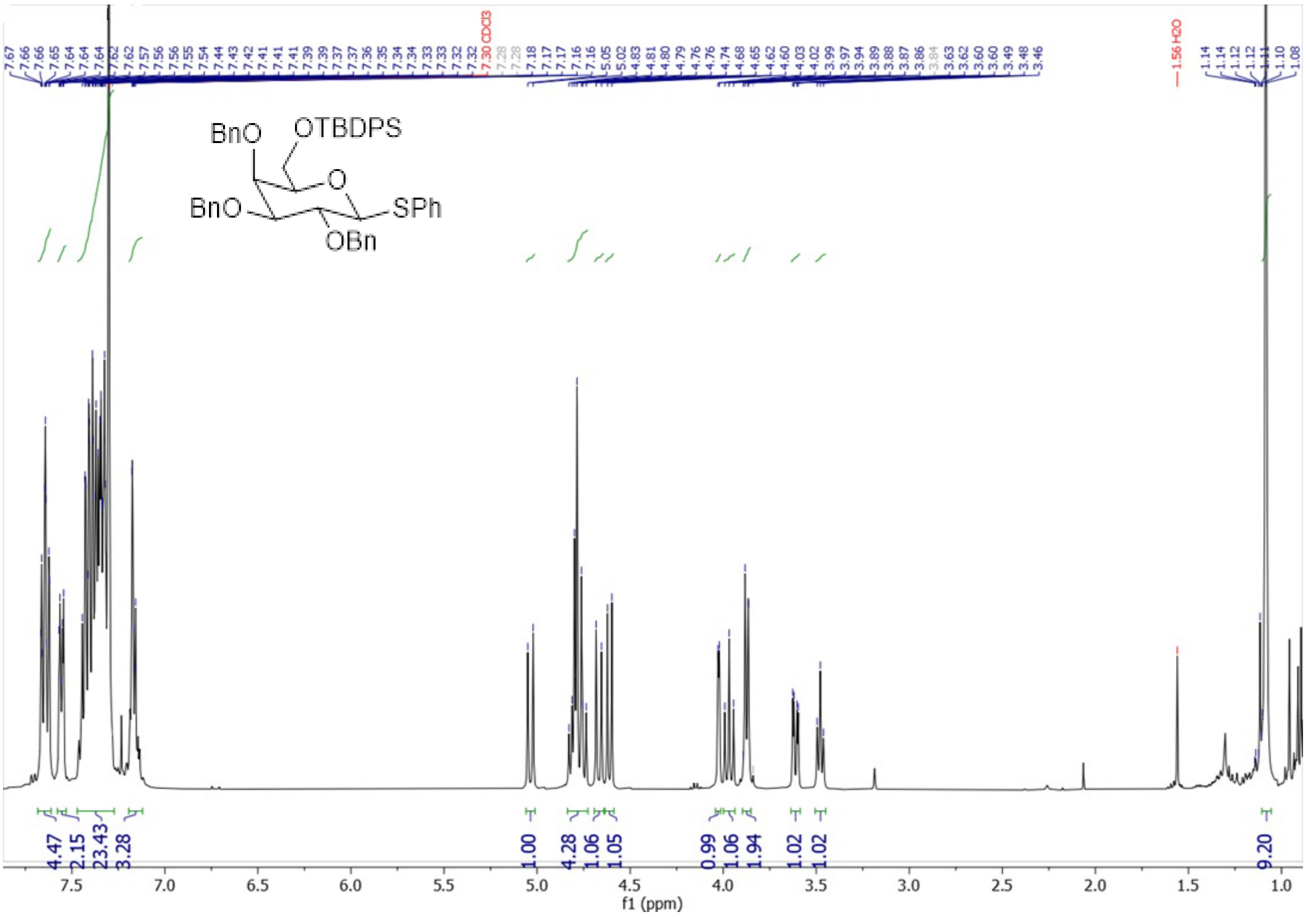

Figure S12 ^{1}H NMR ($CDCl_3$) spectrum of **2c.**

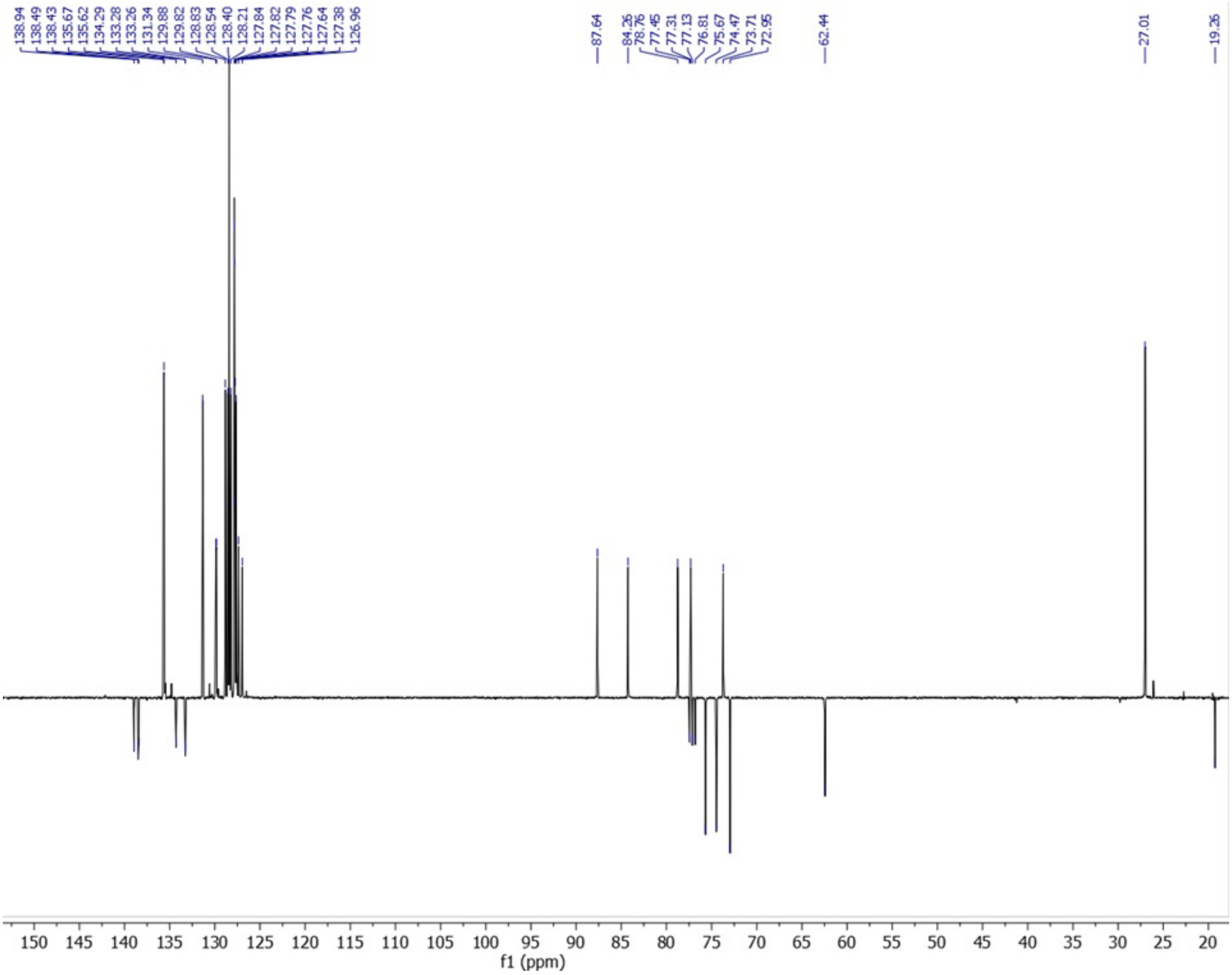

Figure S13 ^{13}C APT-NMR ($CDCl_3$) spectrum of **2c.**

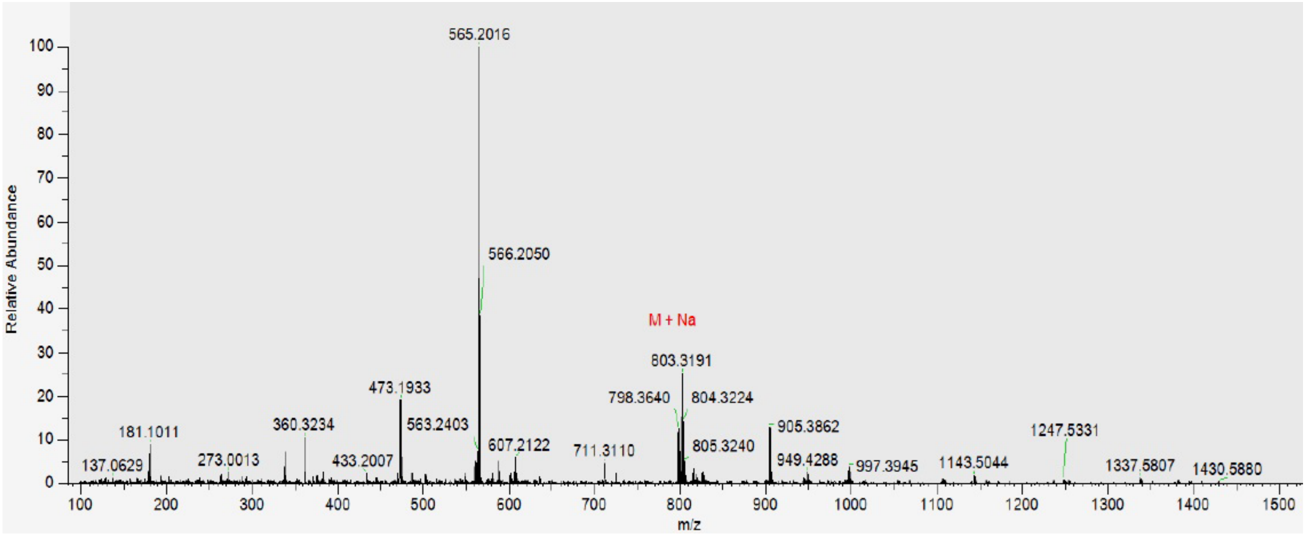

Figure S14 ESI-HRMS spectrum of **2c.**

Compound 3a

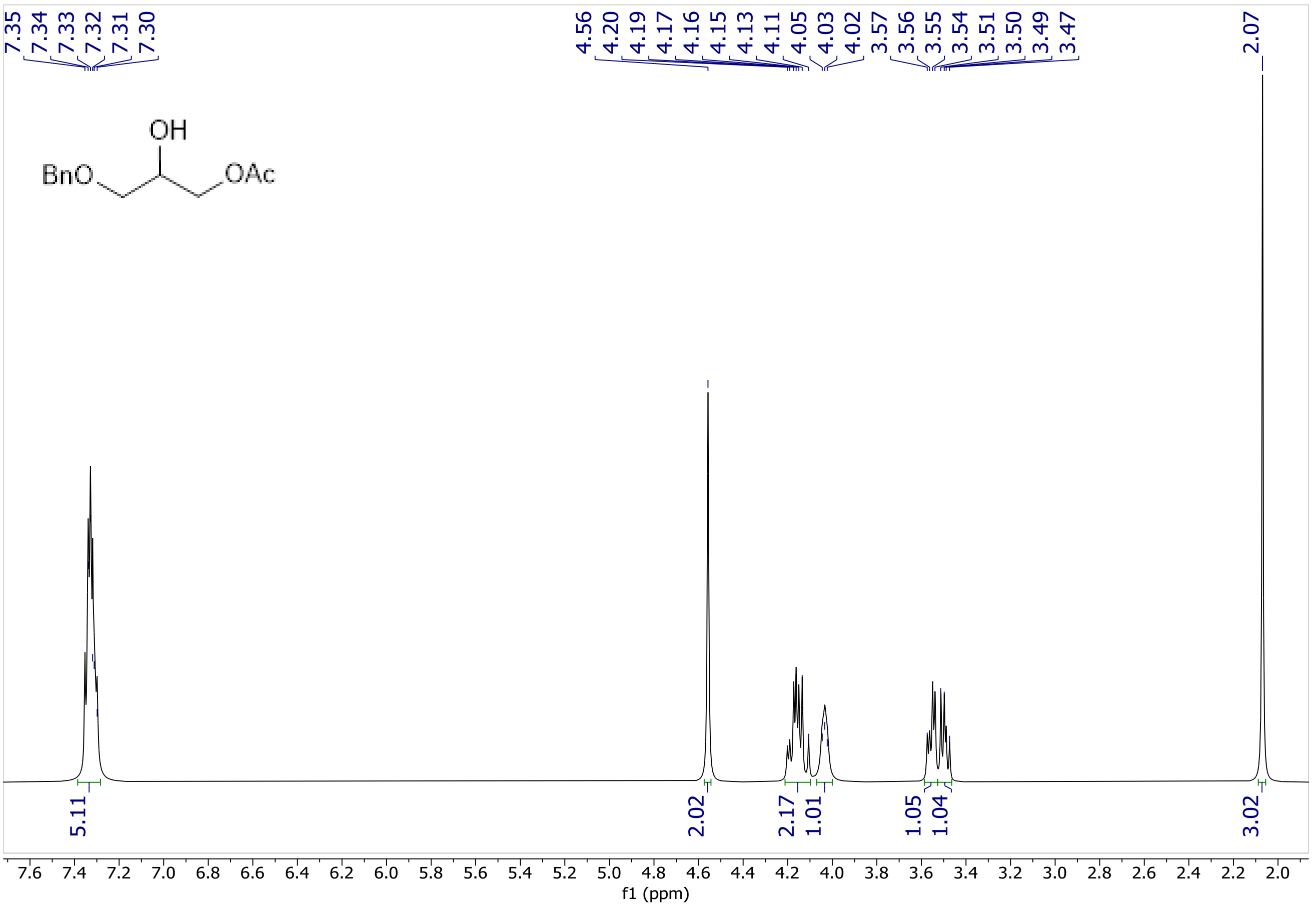

Figure S15 ^{1}H NMR ($CDCl_3$) spectrum of **3a.**

Compound 3b

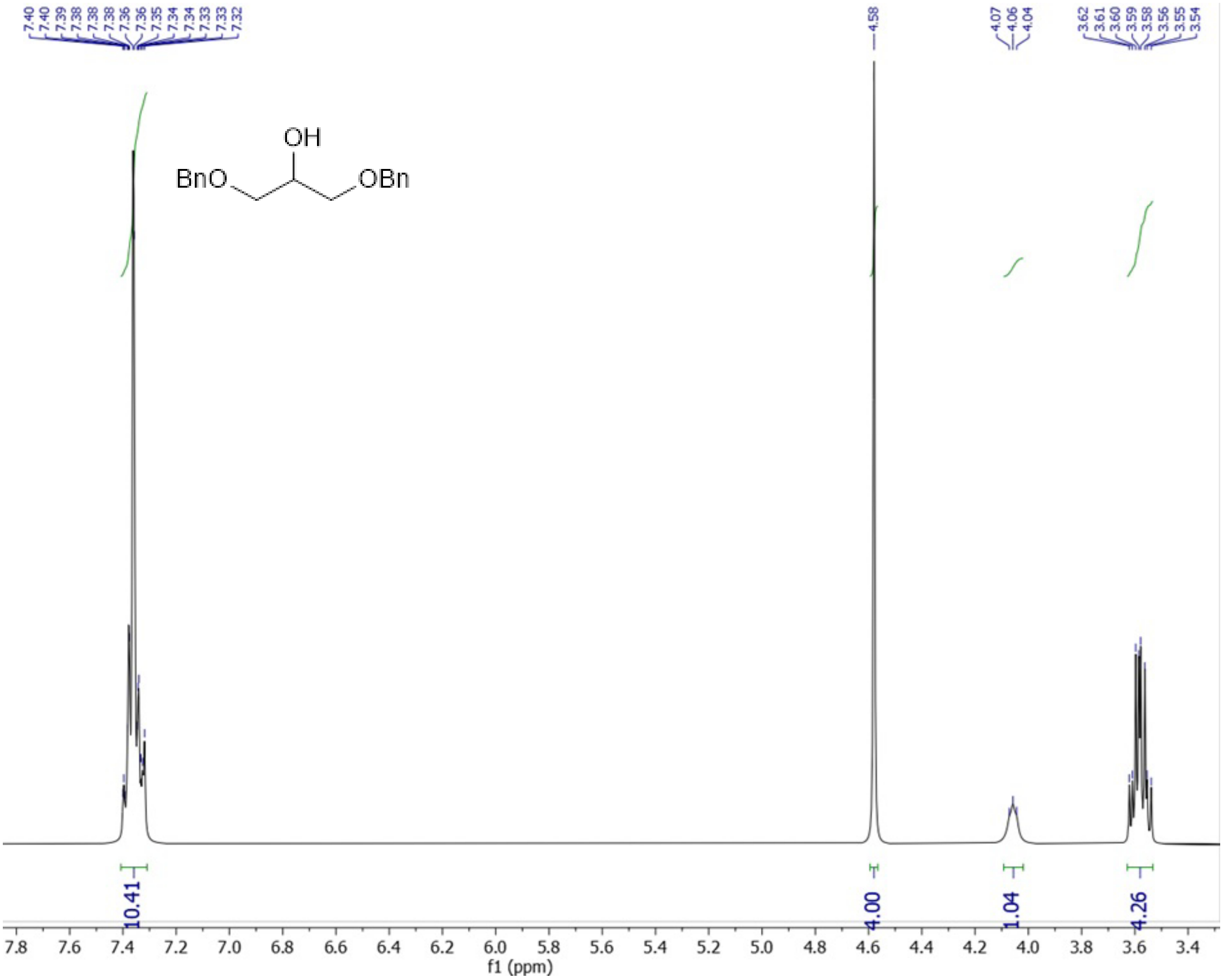

Figure S16 ^{1}H NMR ($CDCl_3$) spectrum of **3b.**

Compound 3c

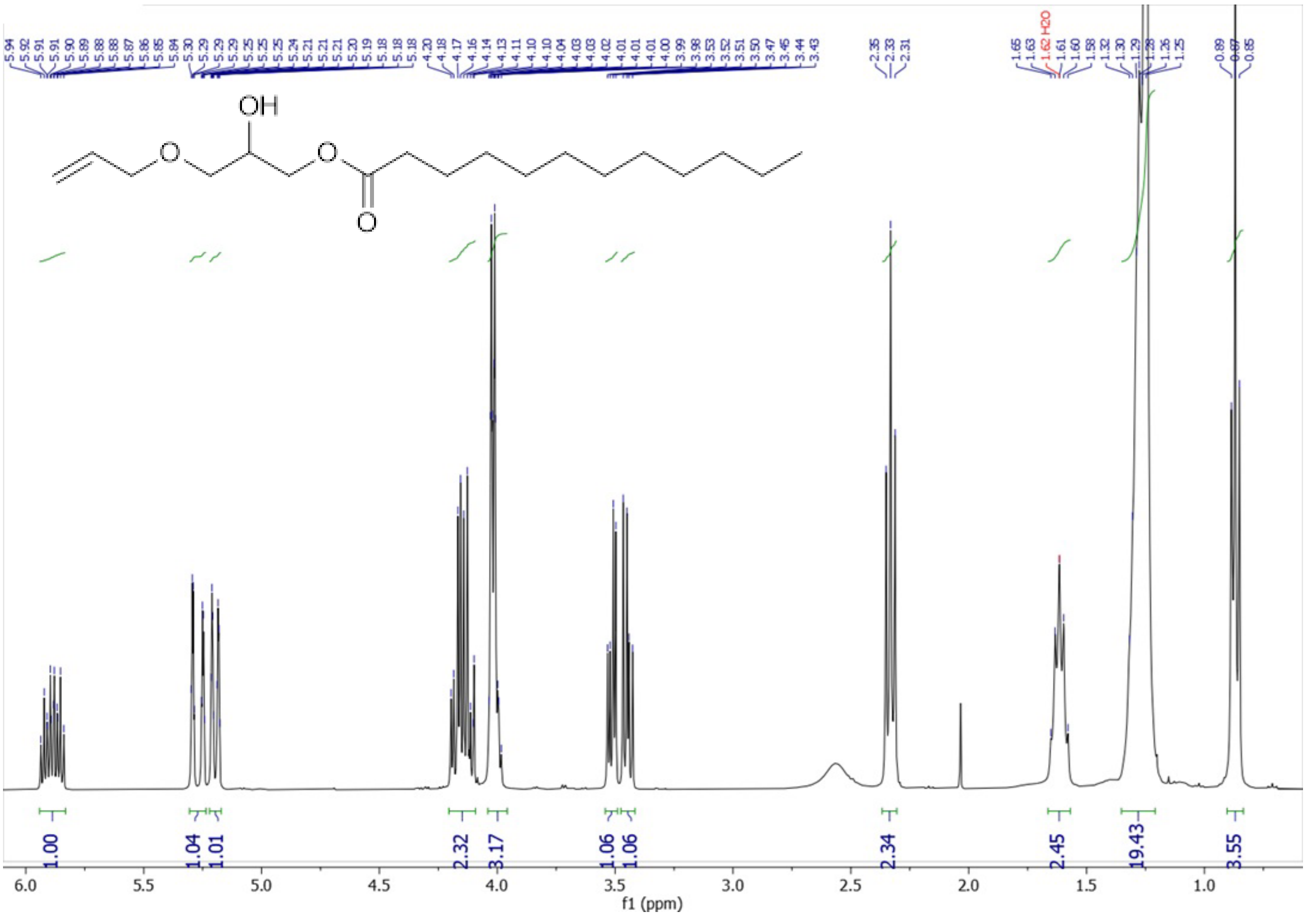

Figure S17 ^{1}H NMR ($CDCl_3$) spectrum of **3c.**

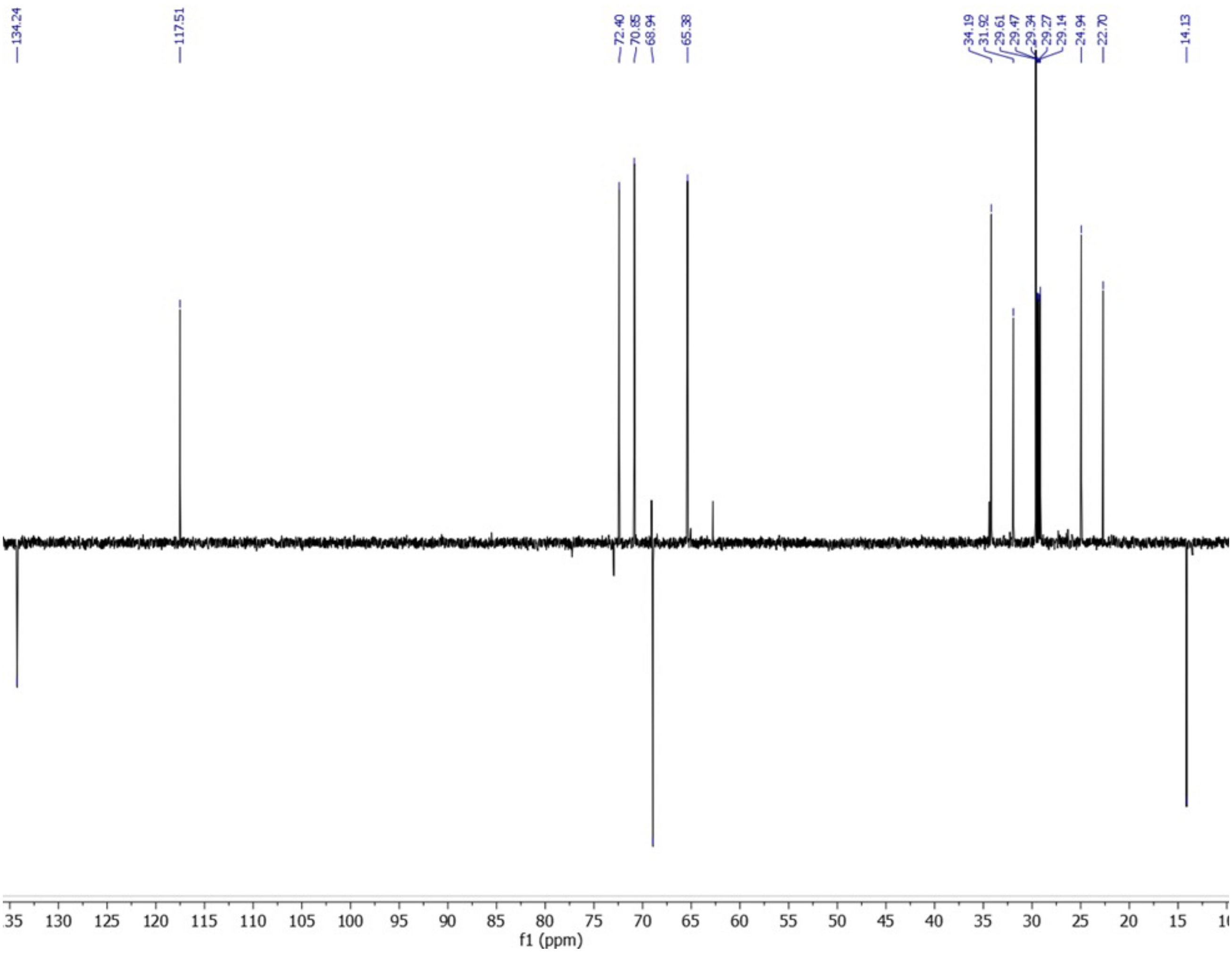

Figure S18 ^{13}C DEPT-135 ($CDCl_3$) NMR spectrum of **3c.**

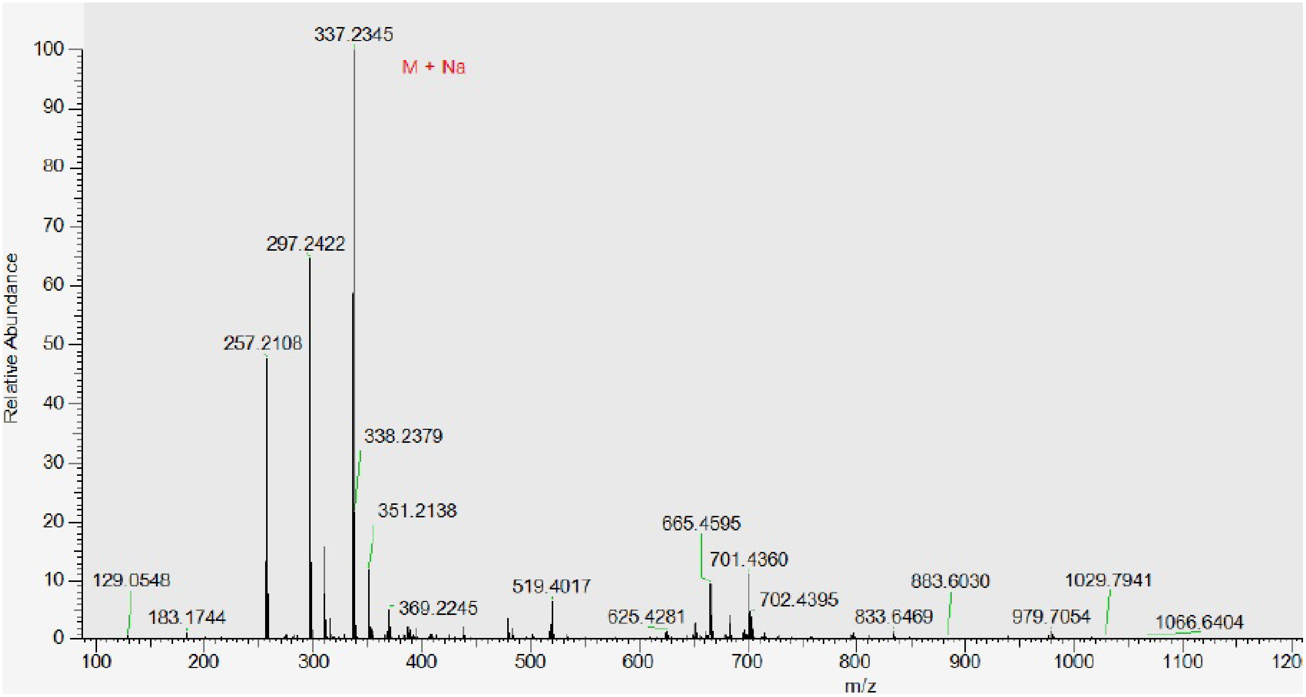

Figure S19 ESI-HRMS spectrum of **2c.**

Compound 4a

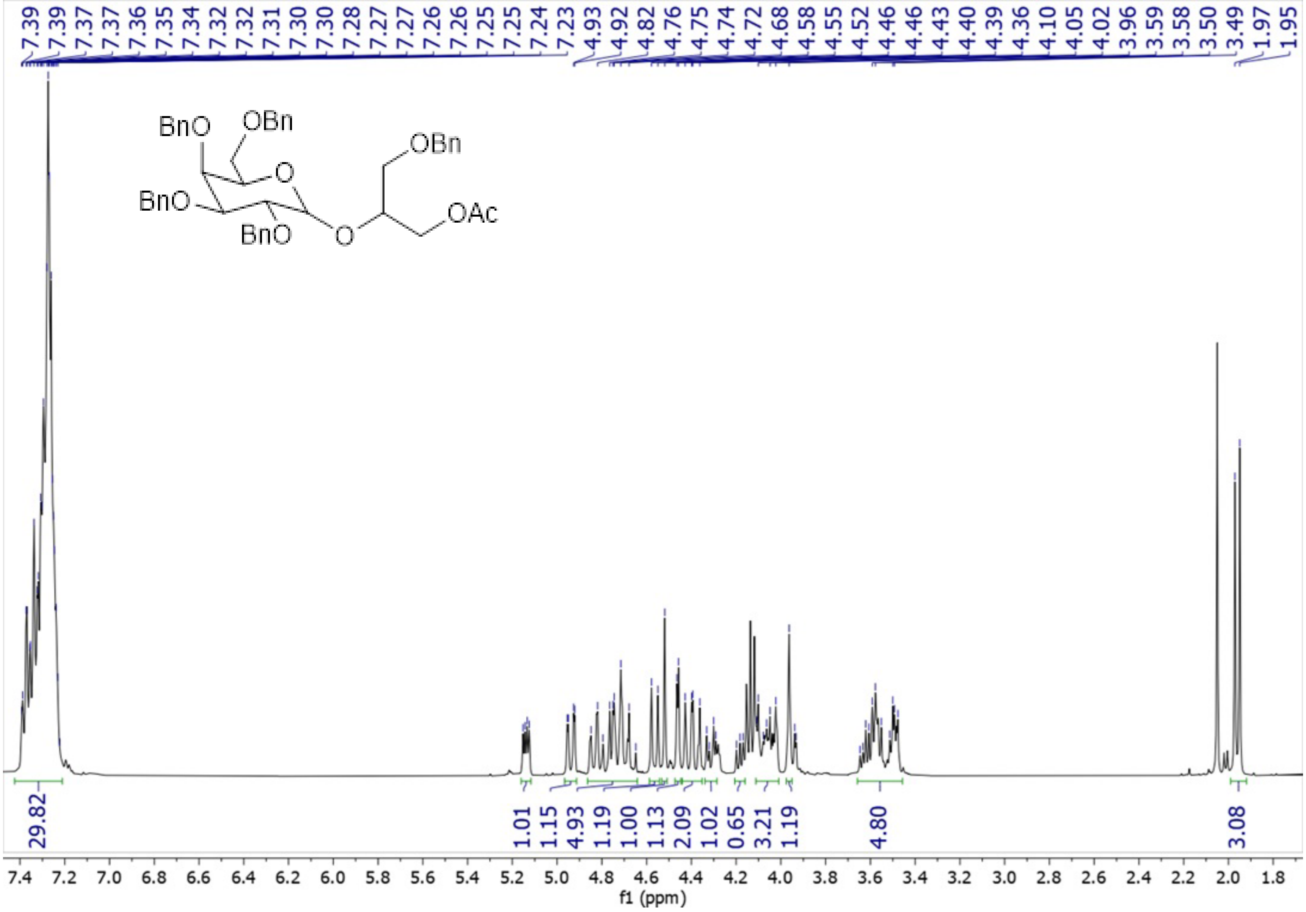

Figure S20 ^{1}H NMR ($CDCl_3$) spectrum of **4a** (α-anomer).

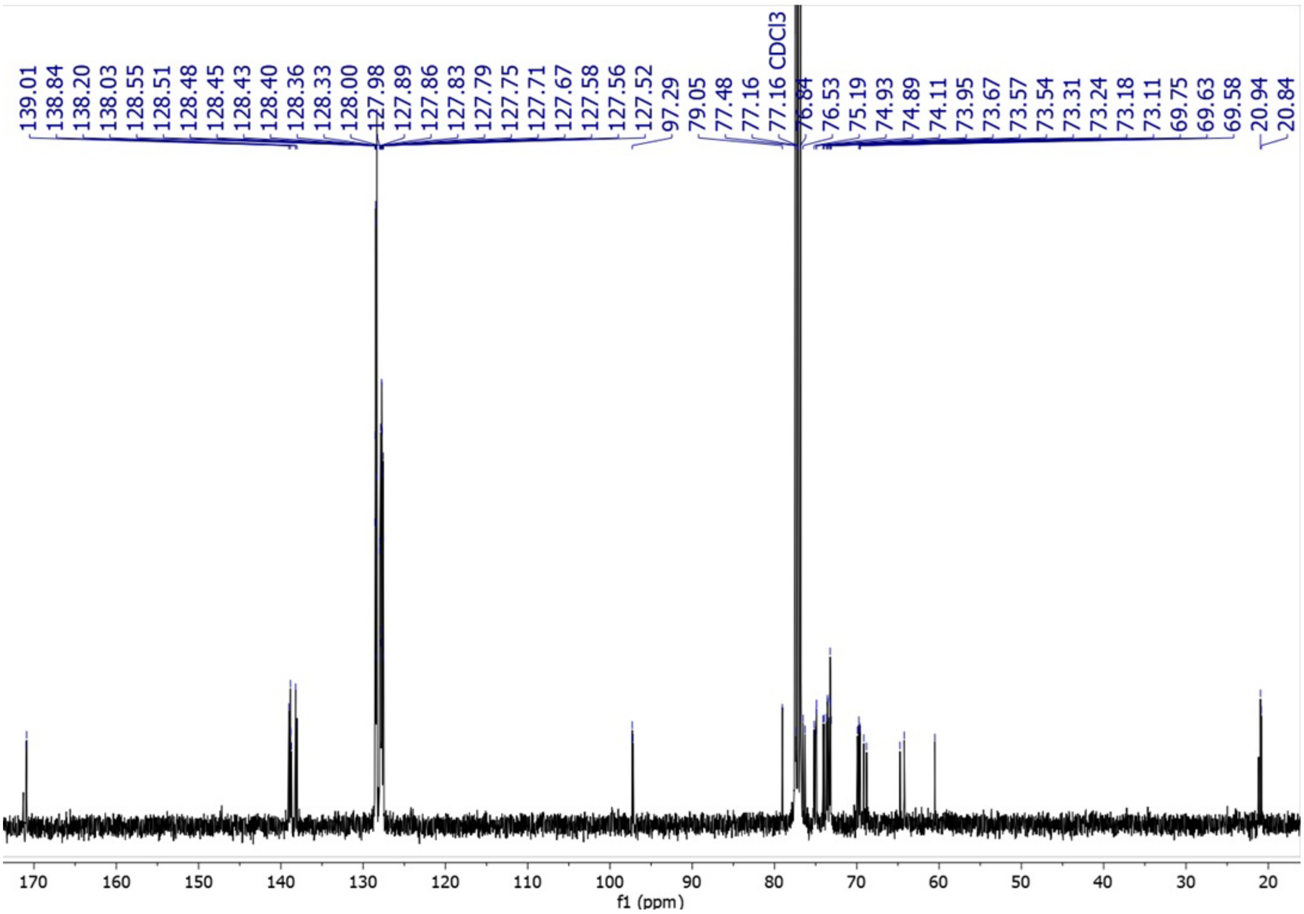

Figure S21 ^{13}C NMR ($CDCl_3$) spectrum of **4a** (α-anomer).

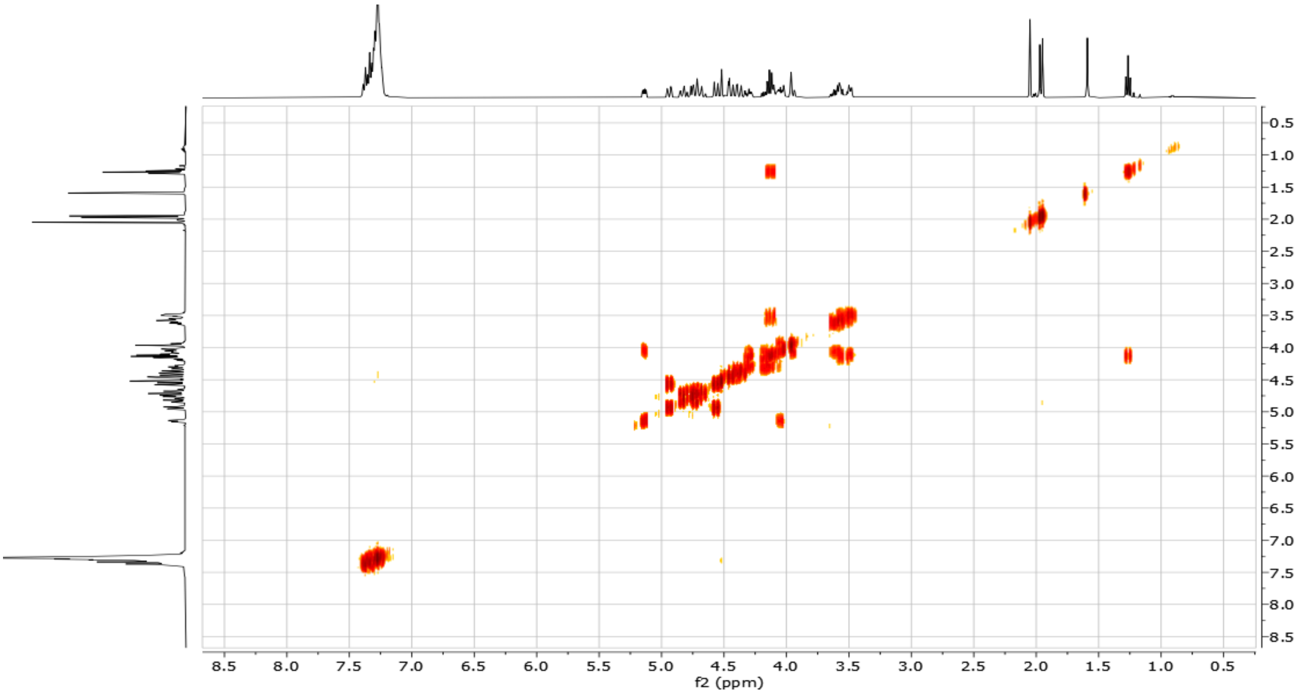

Figure S22 2D NMR COSY ($CDCl_3$) spectrum of **4a** (α-anomer).

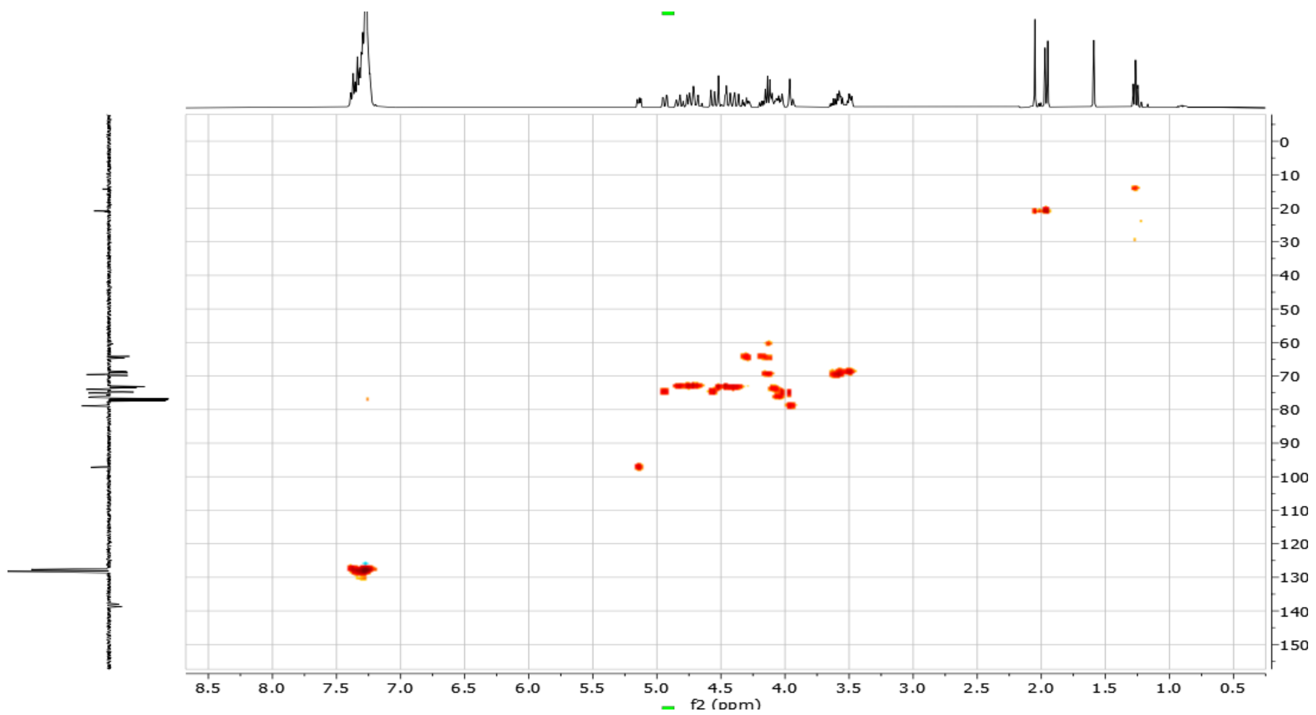

Figure S23 2D NMR HSQC ($CDCl_3$) spectrum of **4a** (*α*-anomer).

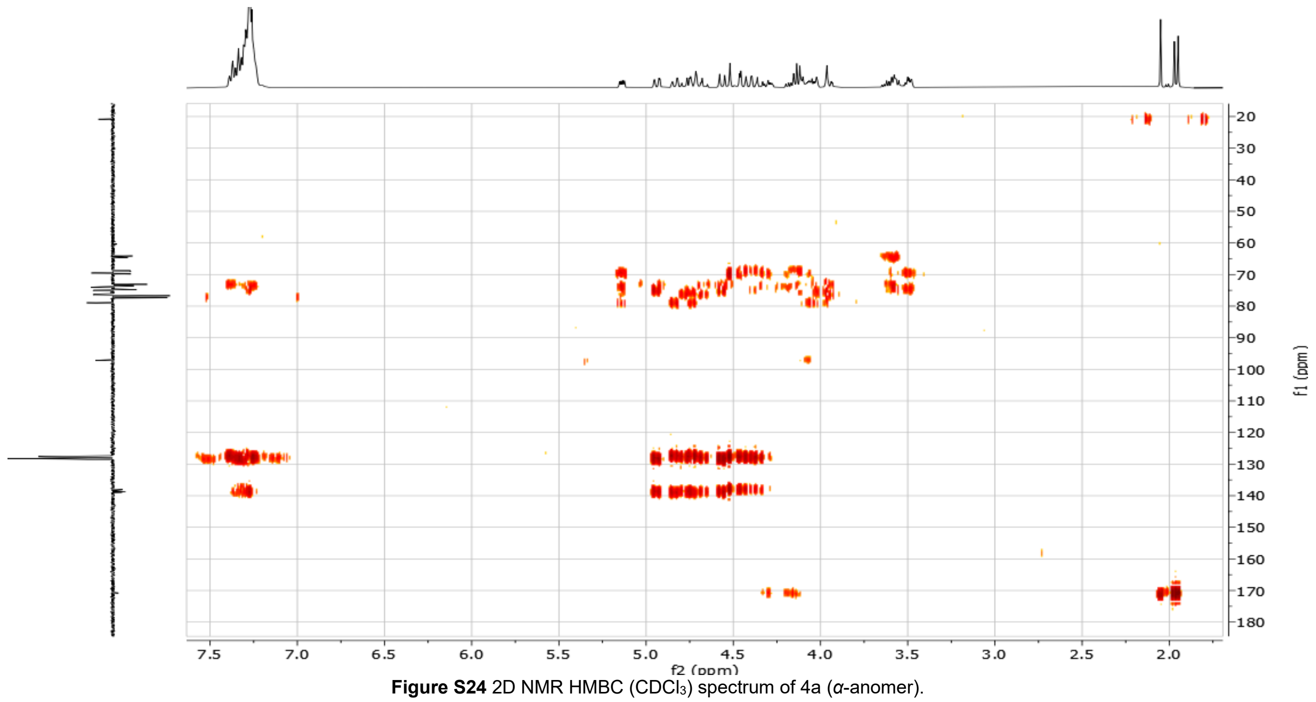

Figure S24 2D NMR HMBC ($CDCl_3$) spectrum of 4a (*α*-anomer).

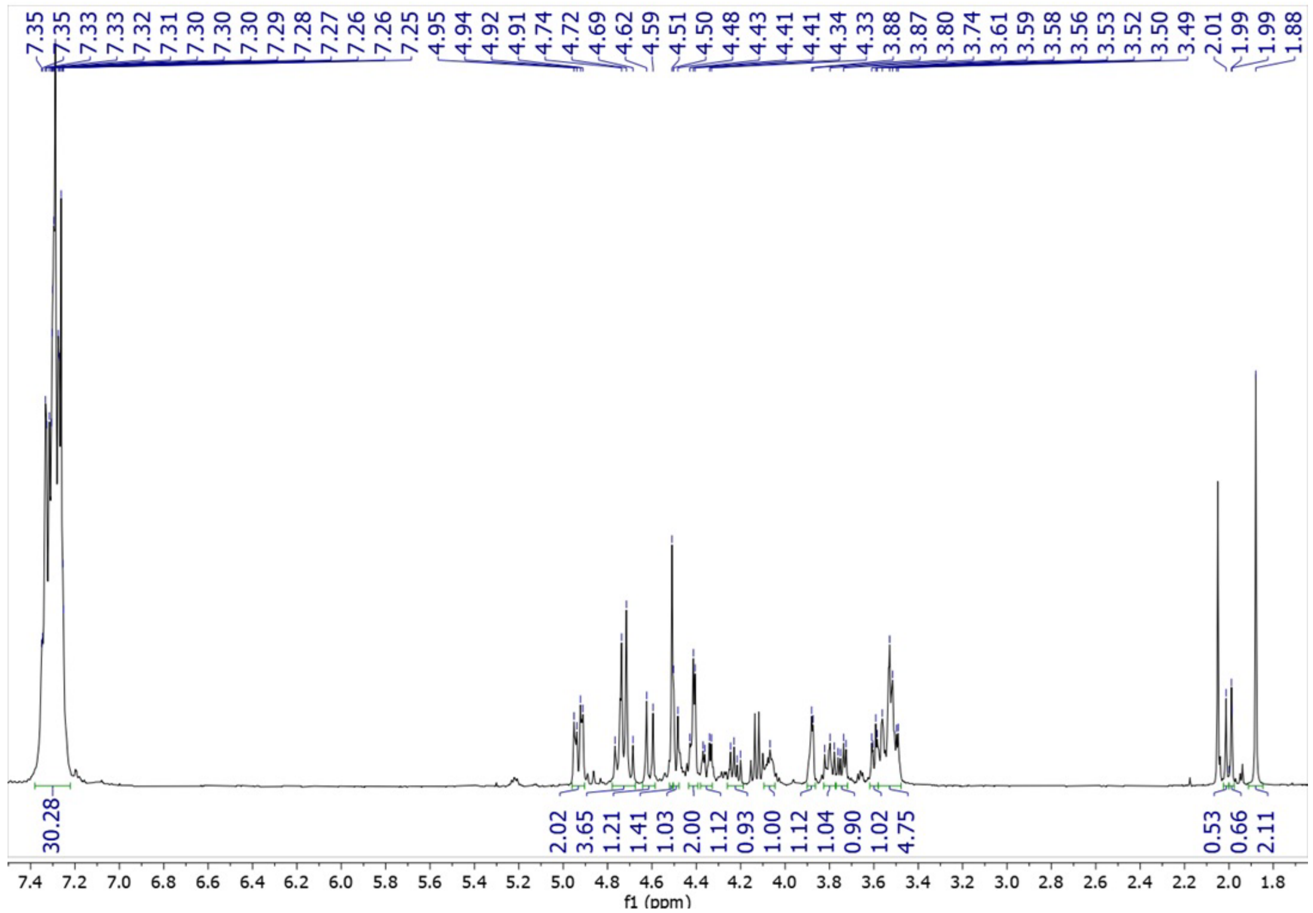

Figure S25 ^{1}H NMR ($CDCl_3$) spectrum of **4a** (*β*-anomer).

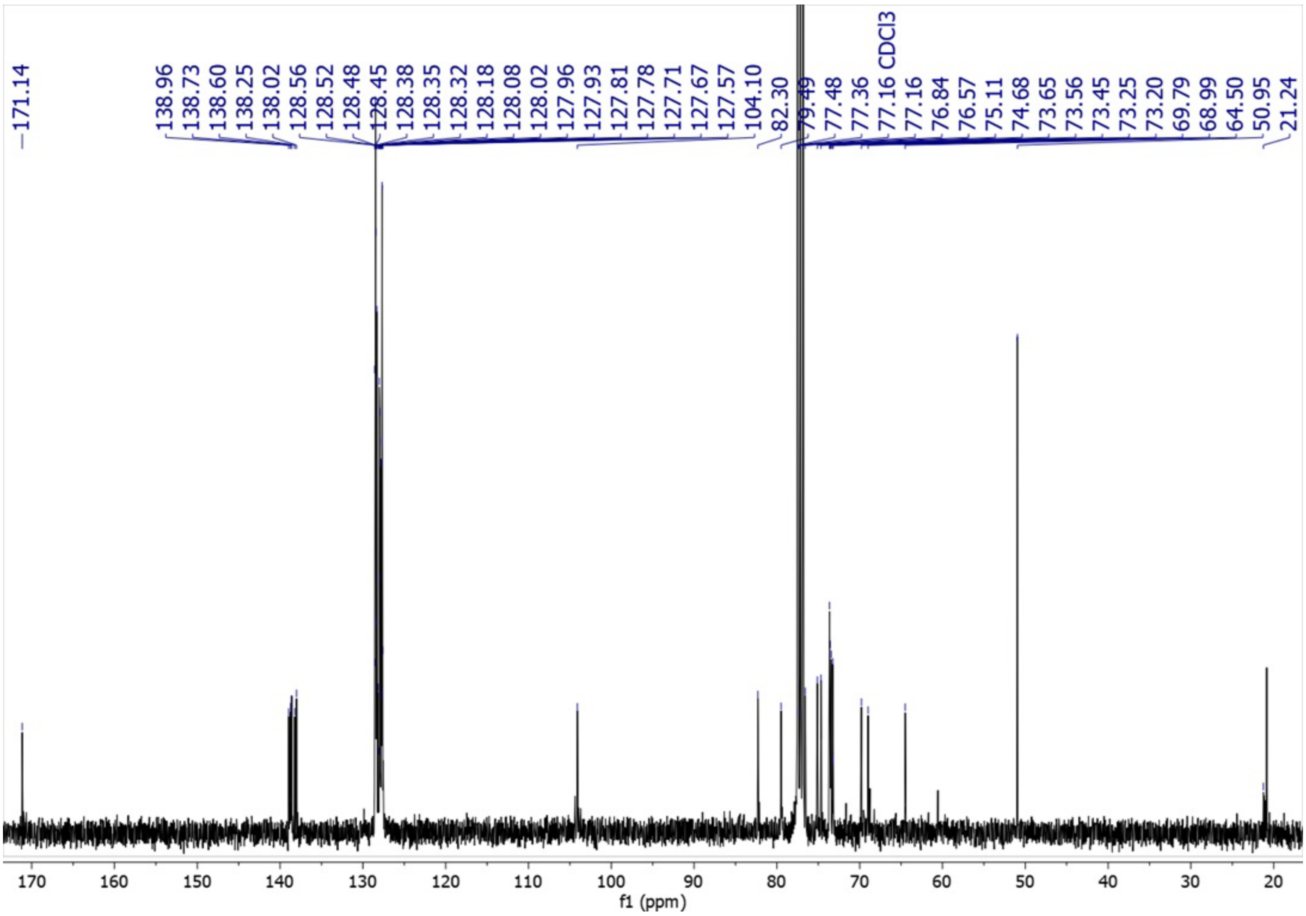

Figure S26 ^{13}C NMR ($CDCl_3$) spectrum of **4a** (*β*-anomer).

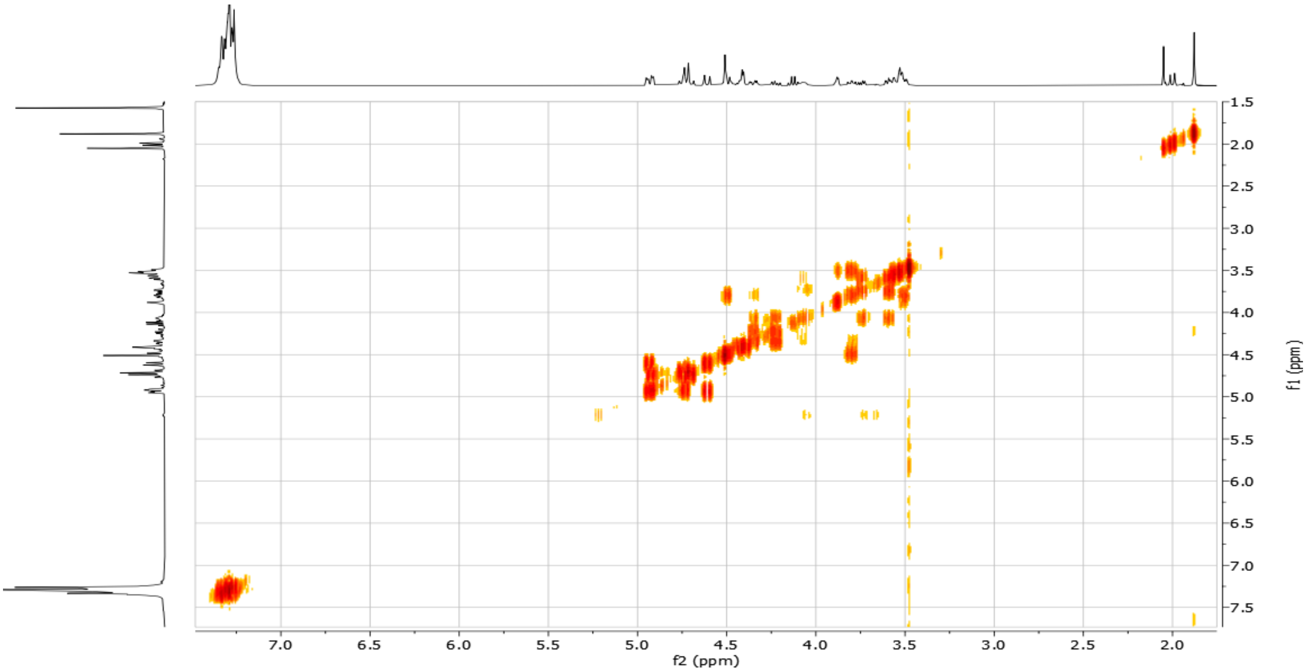

Figure S27 2D NMR COSY ($CDCl_3$) spectrum of **4a** (*β*-anomer).

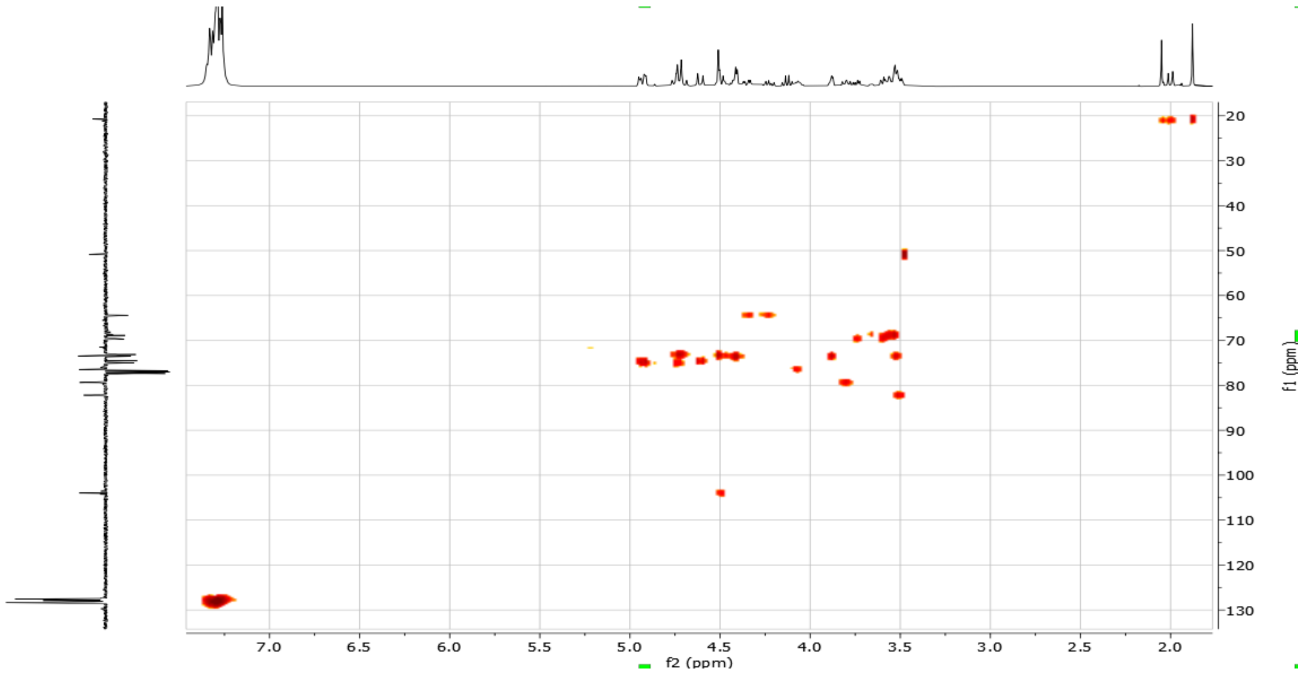

Figure S28 2D NMR HSQC ($CDCl_3$) spectrum of **4a** (β-anomer).

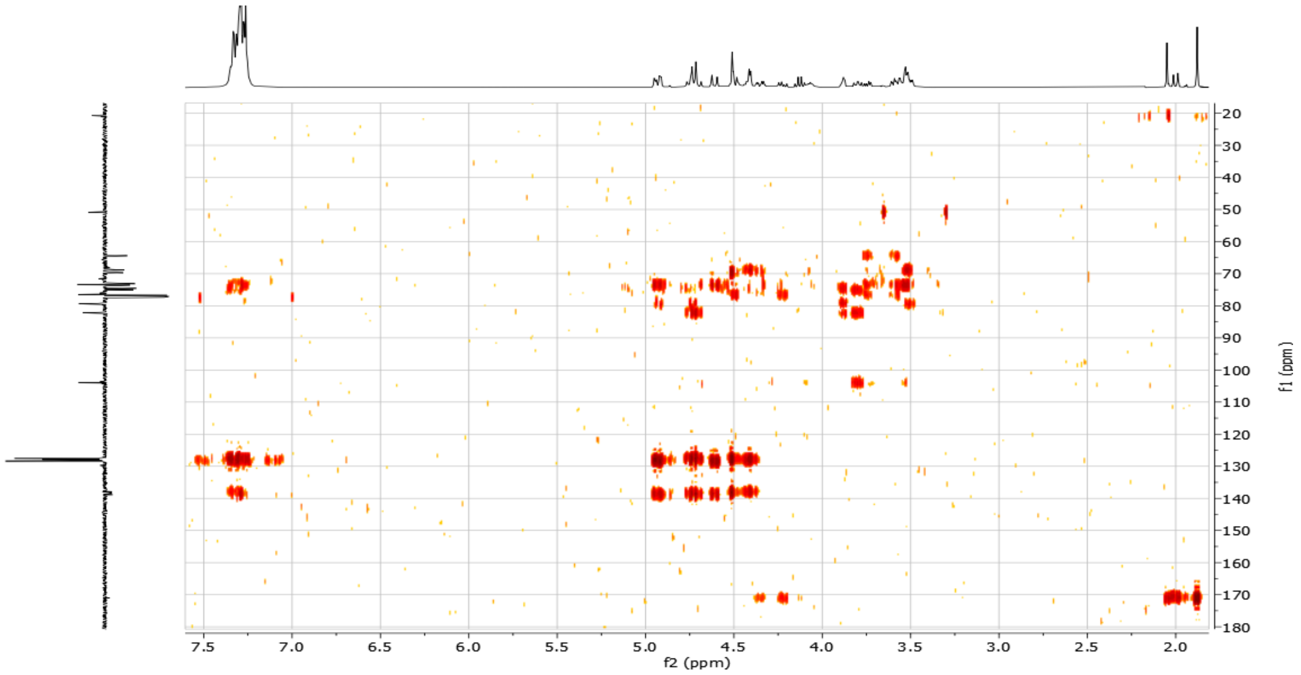

Figure S29 2D NMR HMBC ($CDCl_3$) spectrum of **4a** (β-anomer).

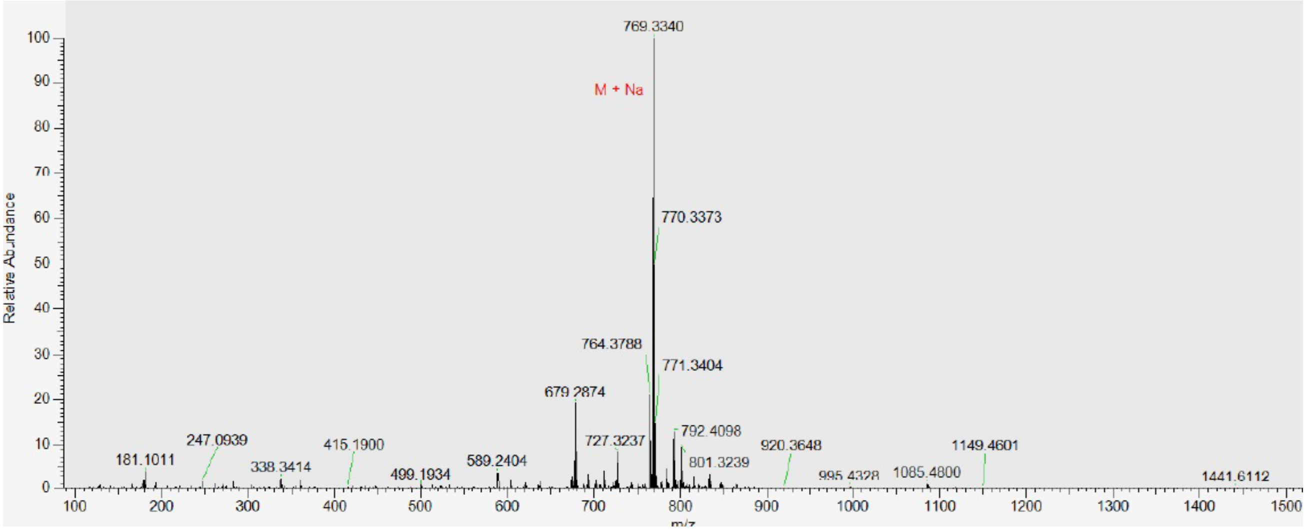

Figure S30 ESI-HRMS spectrum of **4a.**

Compound 4b

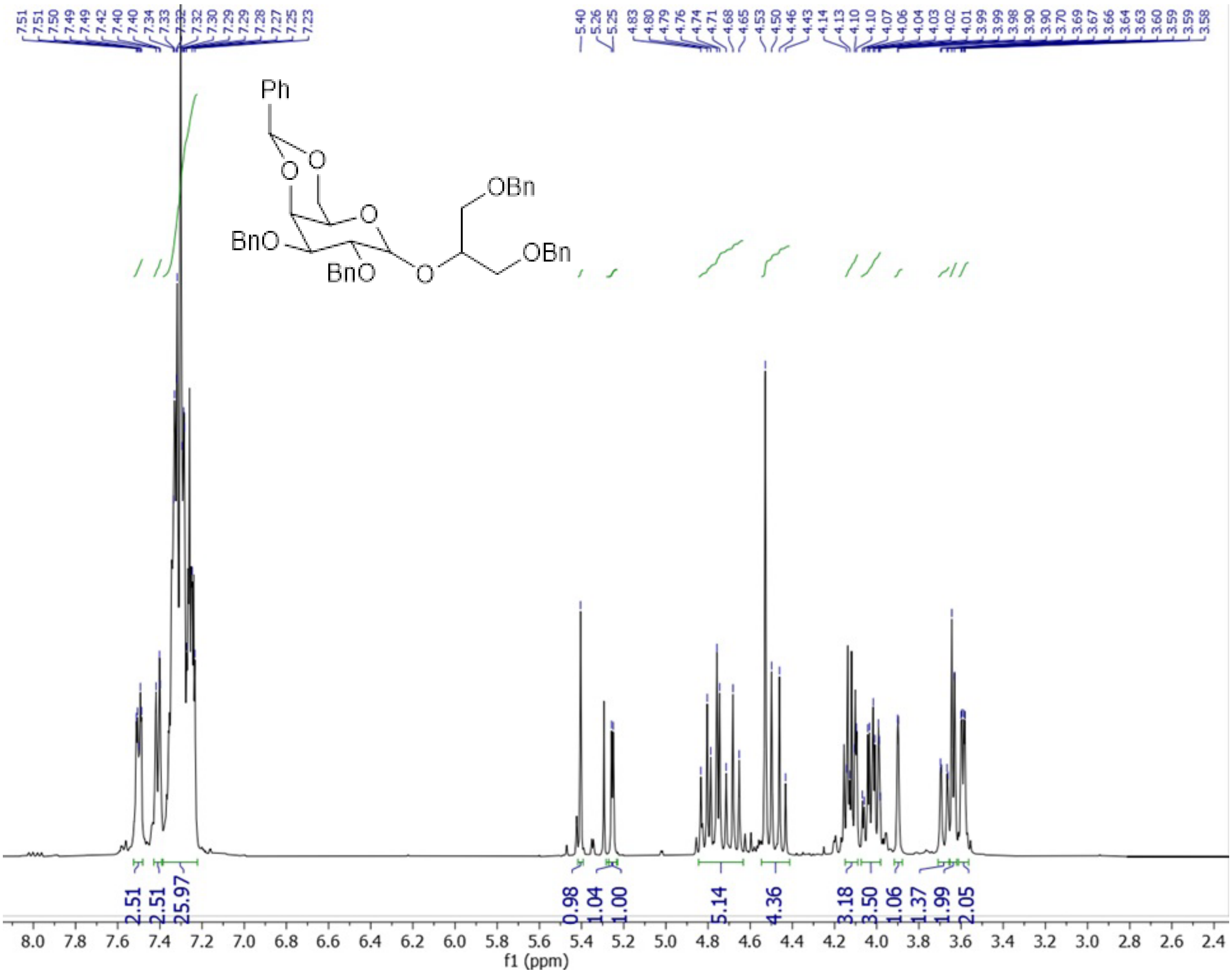

Figure S31 ¹H NMR ($CDCl_3$) spectrum of **4b** (α-anomer).

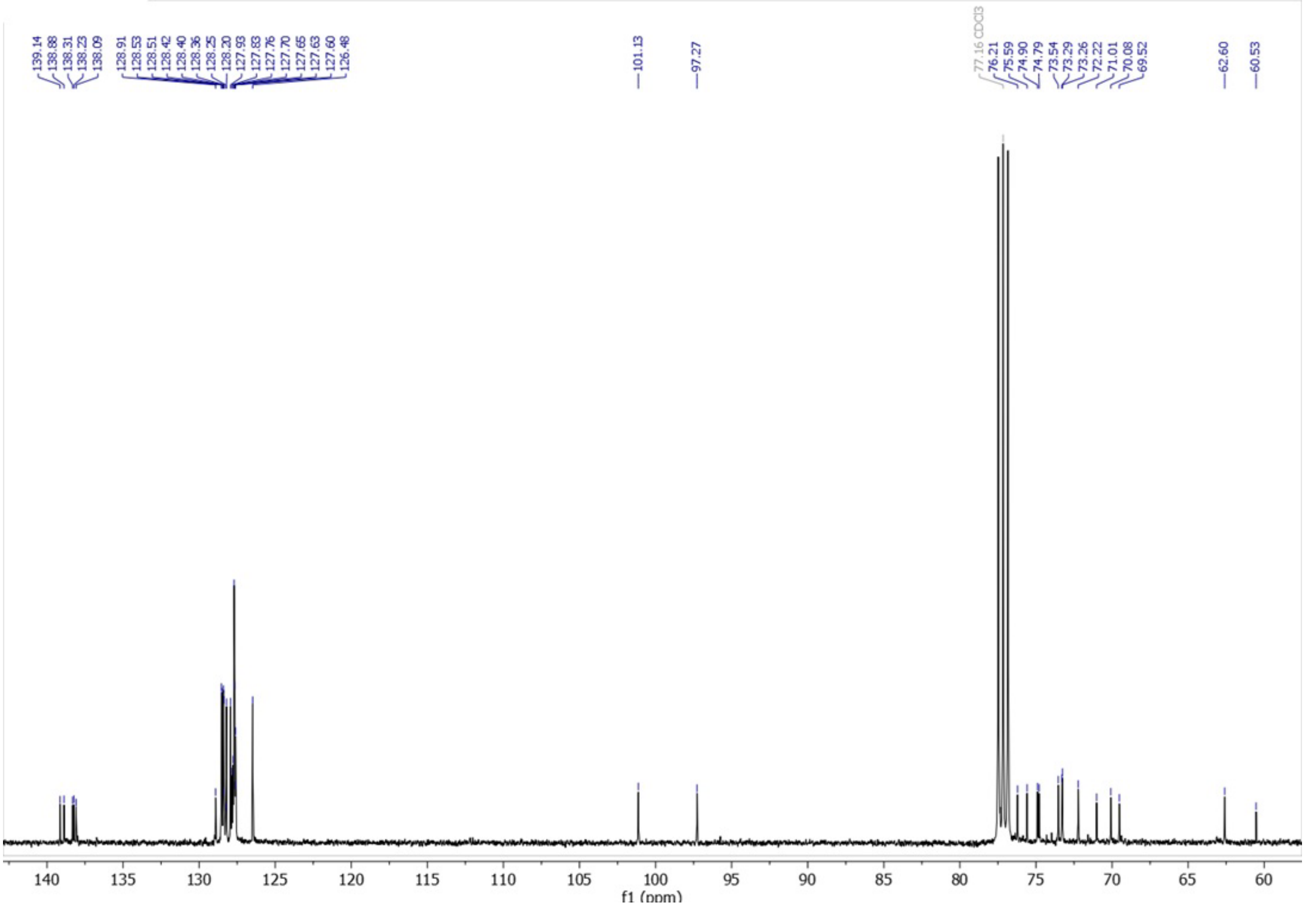

Figure S32 ^{13}C NMR ($CDCl_3$) spectrum of **4b** (α-anomer).

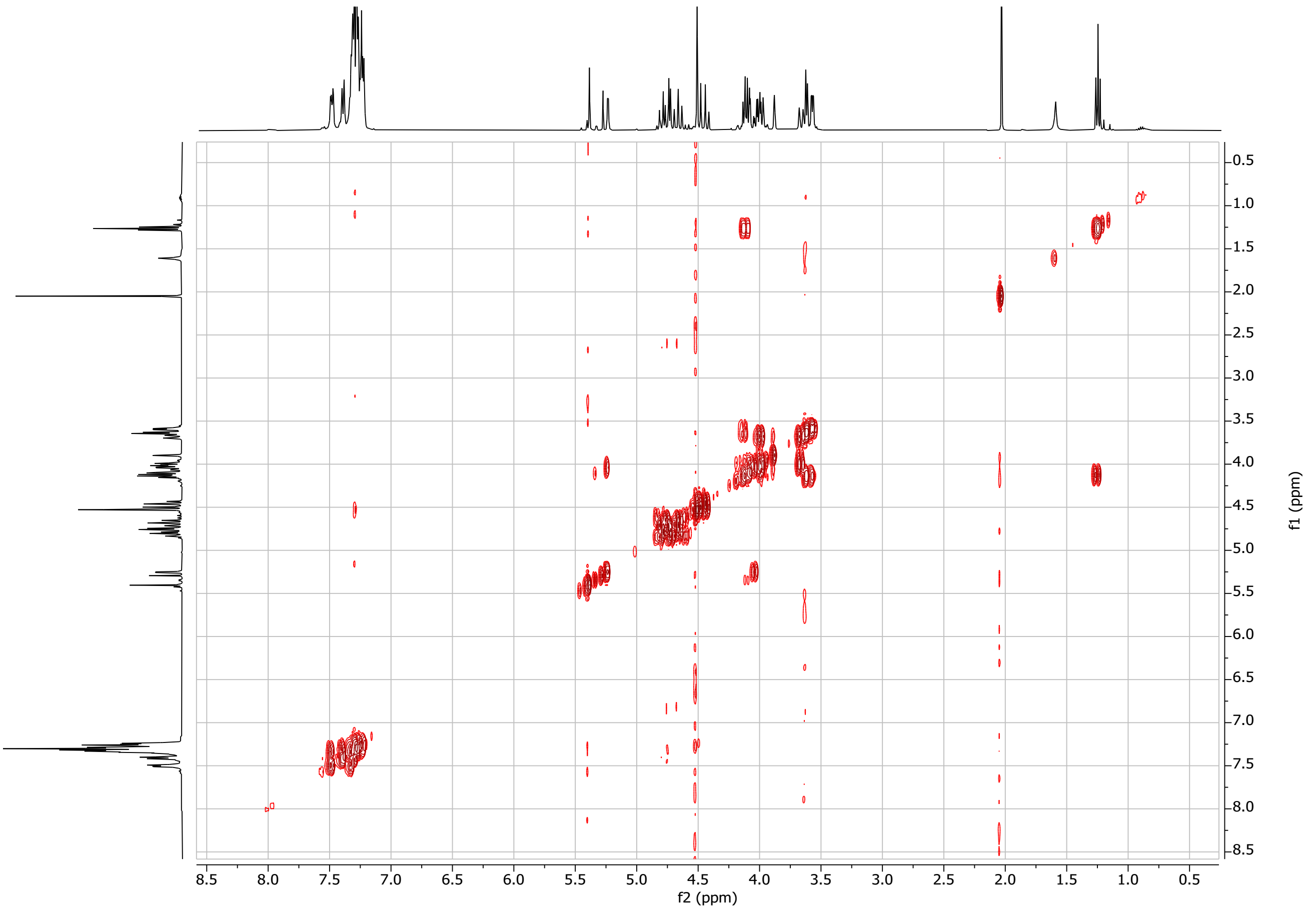

Figure S33 2D NMR COSY ($CDCl_3$) spectrum of **4b** (*α*-anomer).

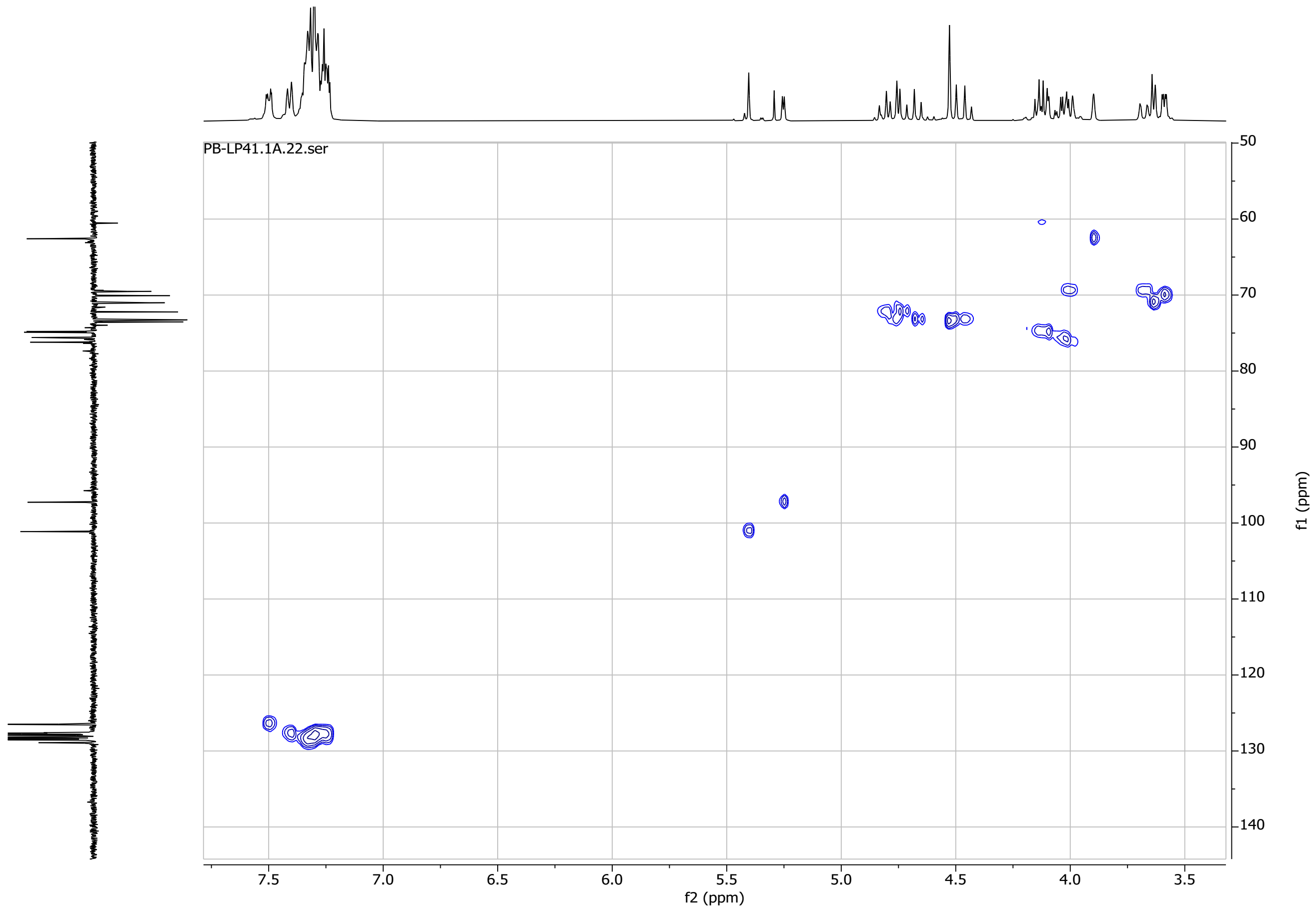

Figure S34 2D NMR HSQC ($CDCl_3$) spectrum of **4b** (*α*-anomer).

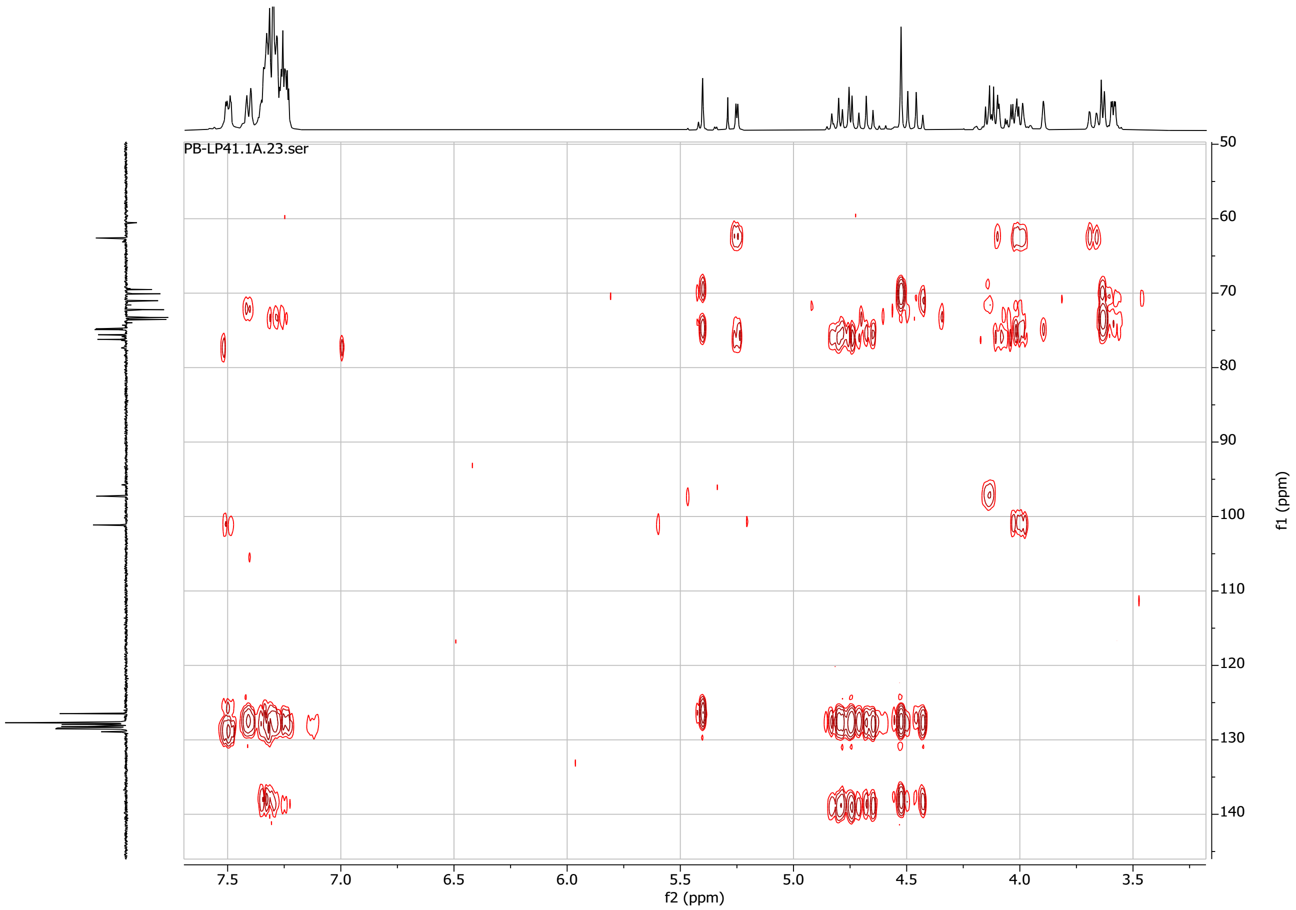

Figure S35 2D NMR HMBC ($CDCl_3$) spectrum of **4b** (*α*-anomer).

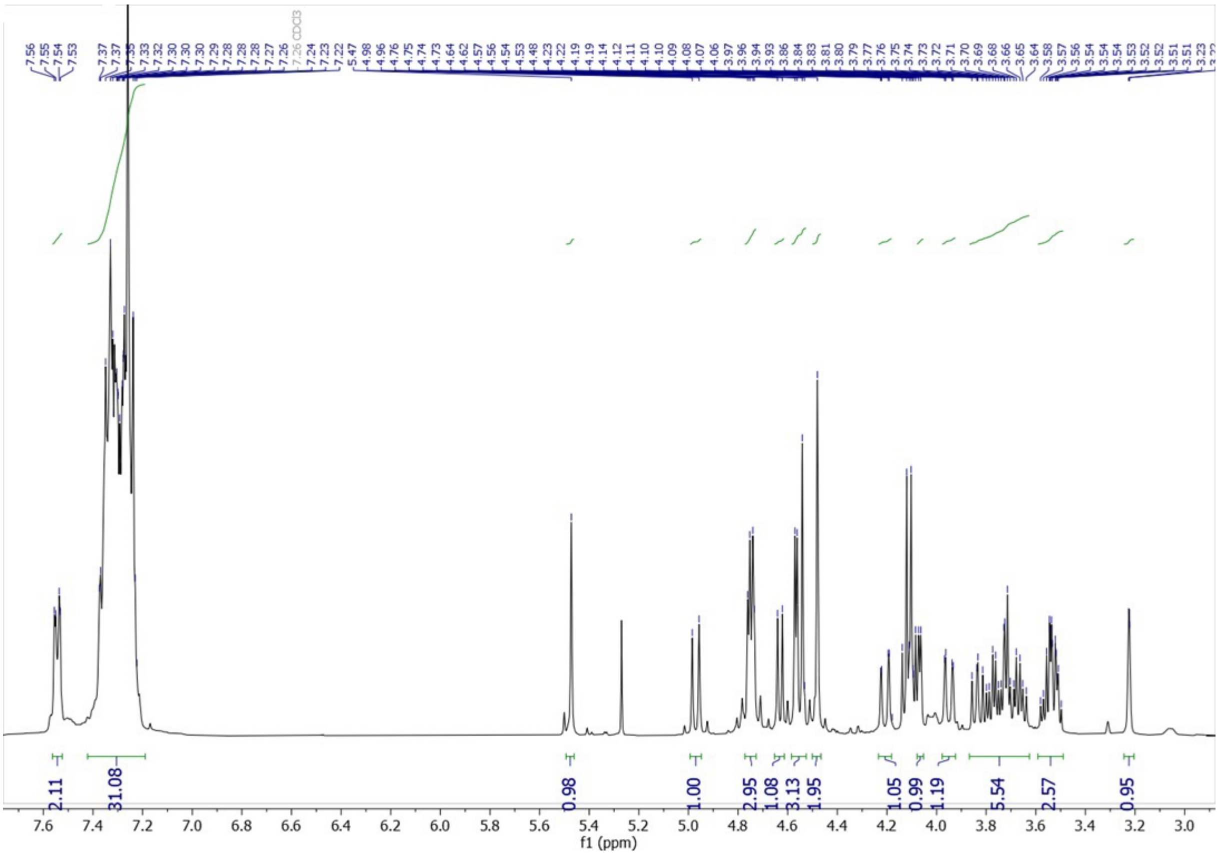

Figure S36 ¹H NMR ($CDCl_3$) spectrum of **4b** (*β*-anomer).

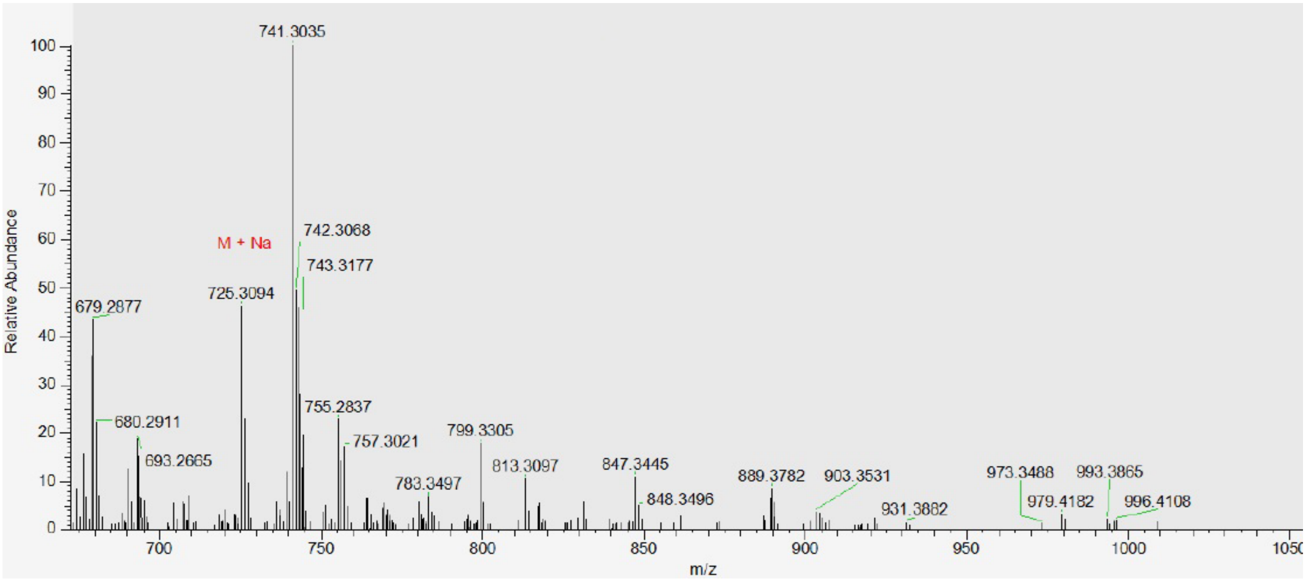

Figure S37 ESI-HRMS spectrum of **4b.**

Compound 4c

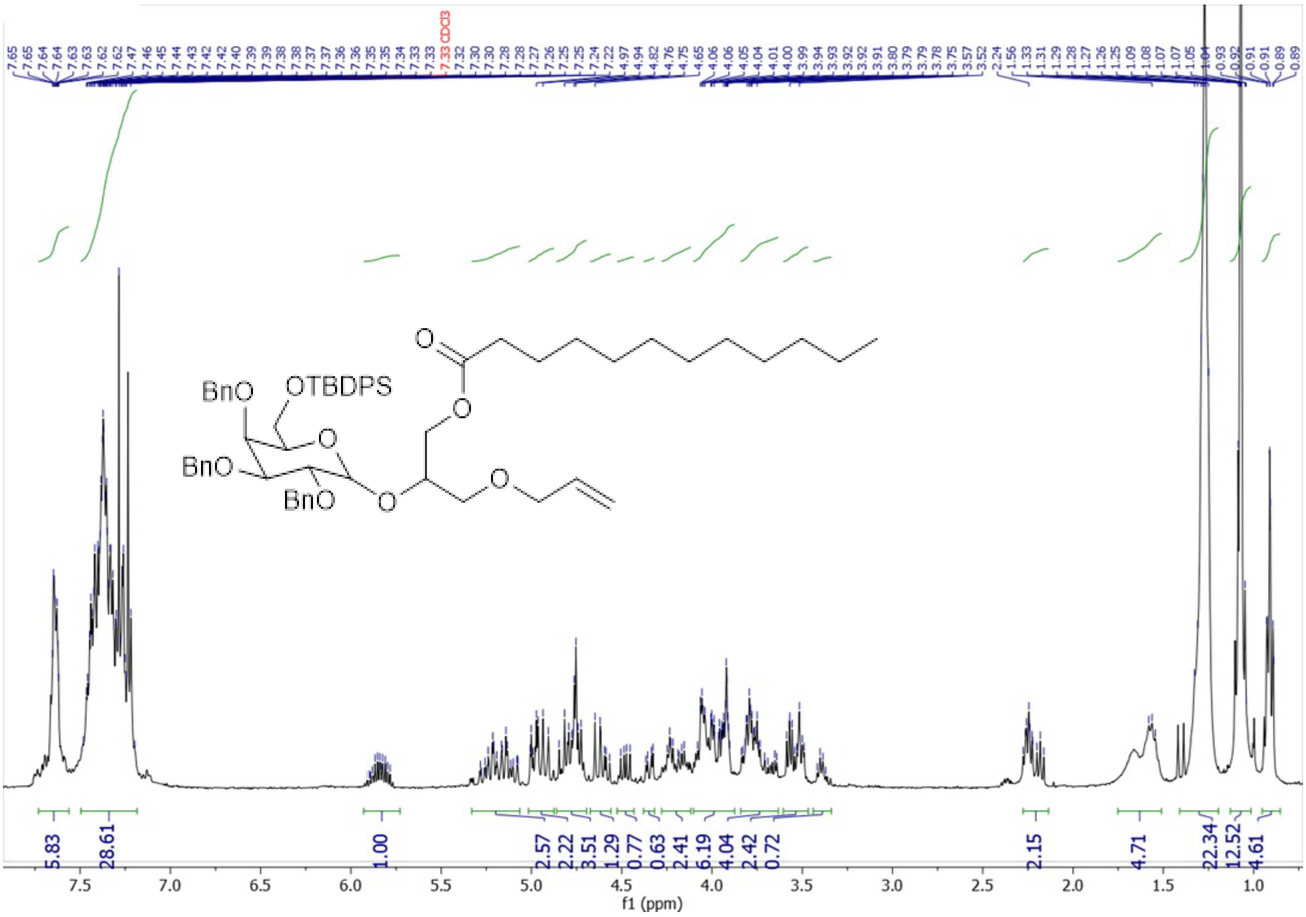

Figure S38 ^{1}H NMR ($CDCl_3$) spectrum of **4c.**

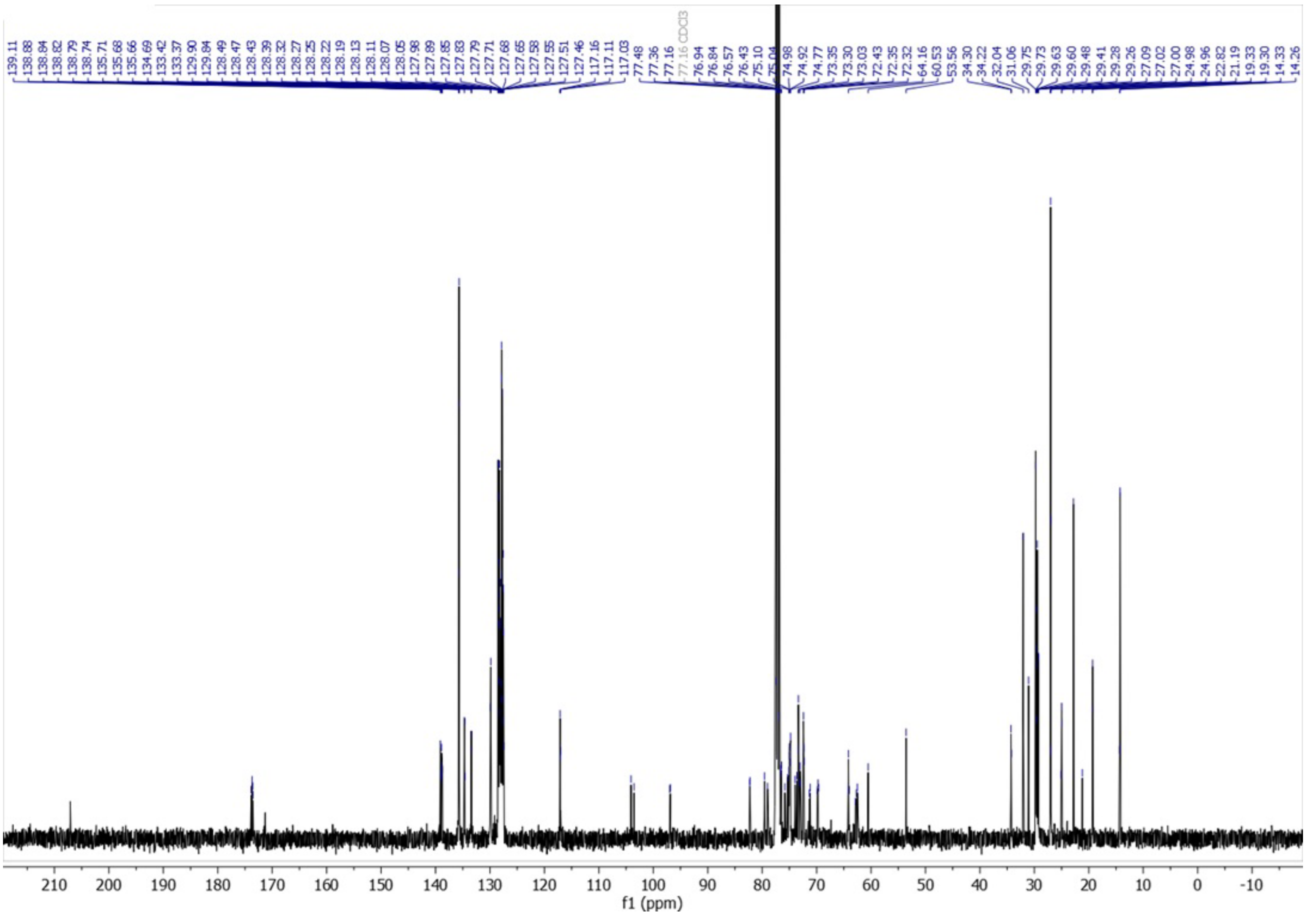

Figure S39 ^{13}C NMR ($CDCl_3$) spectrum of **4c.**

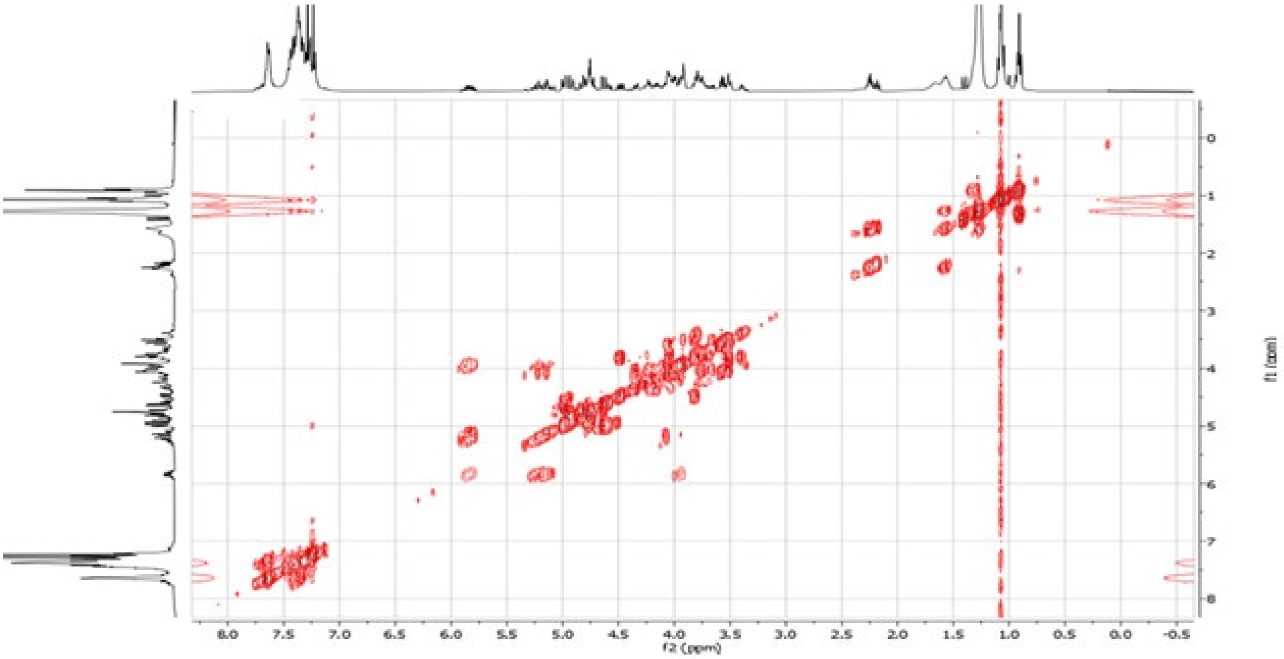

Figure S40 2D NMR COSY ($CDCl_3$) spectrum of **4c.**

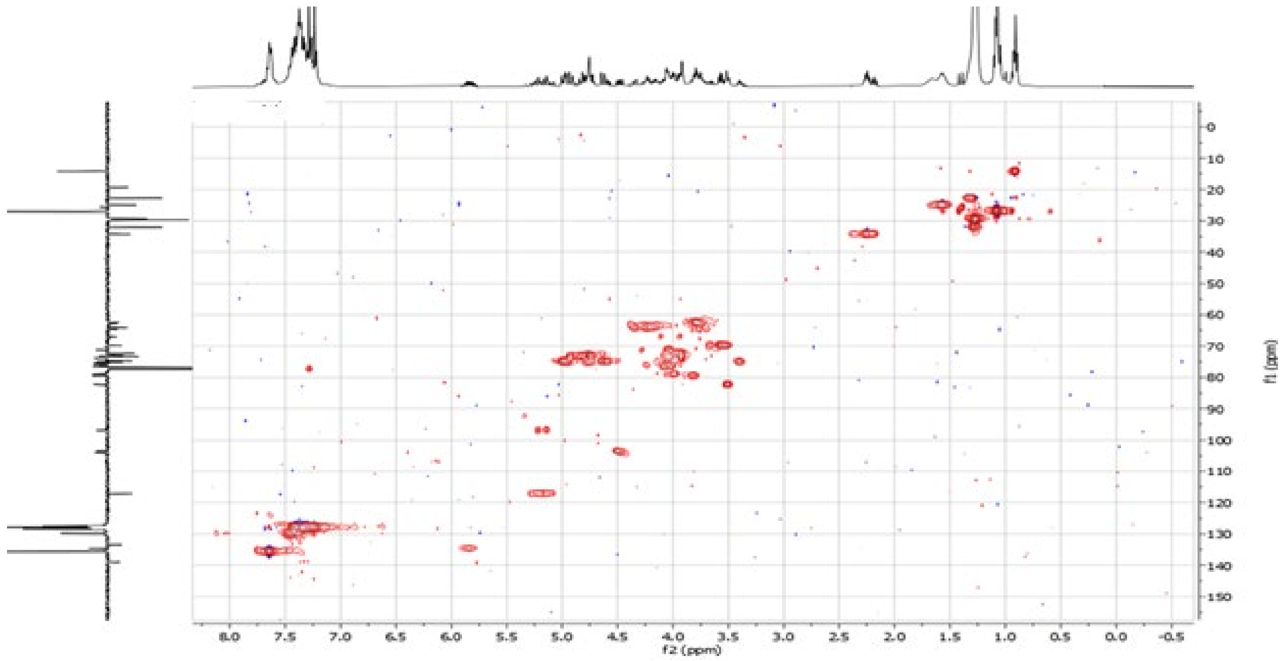

Figure S41 2D NMR HSQC ($CDCl_3$) spectrum of **4c.**

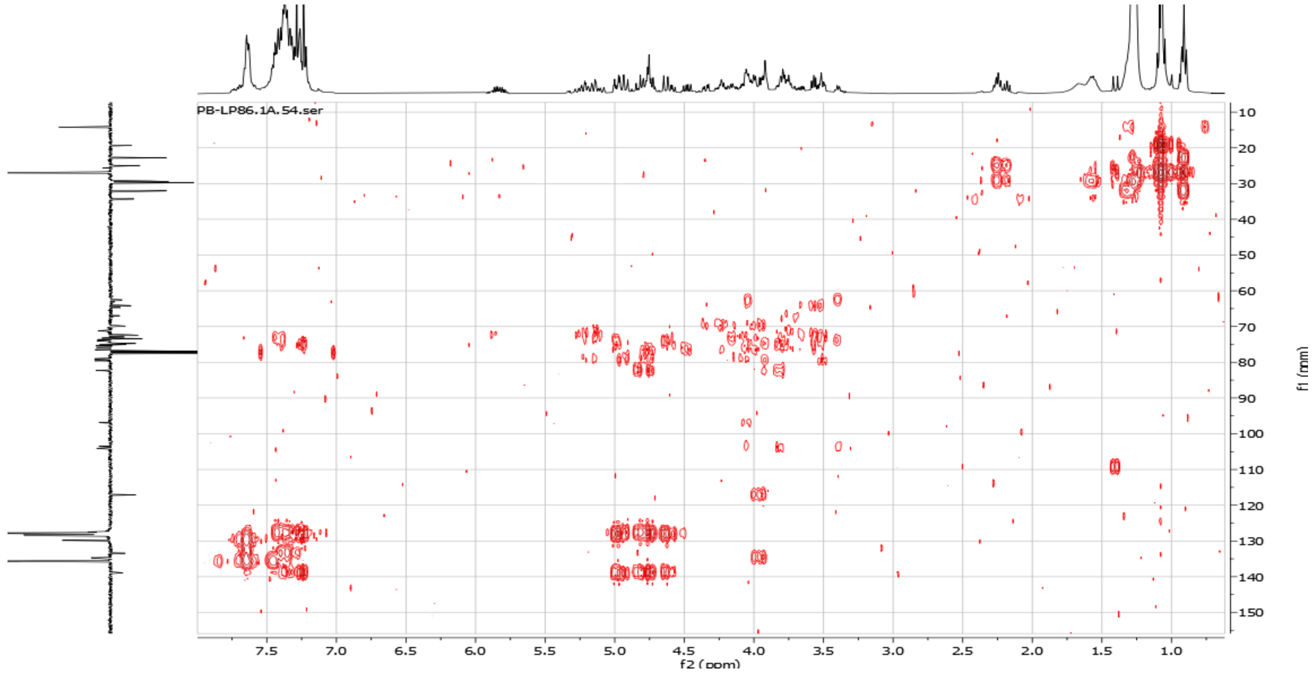

Figure S42 2D NMR HMBC ($CDCl_3$) spectrum of **4c.**

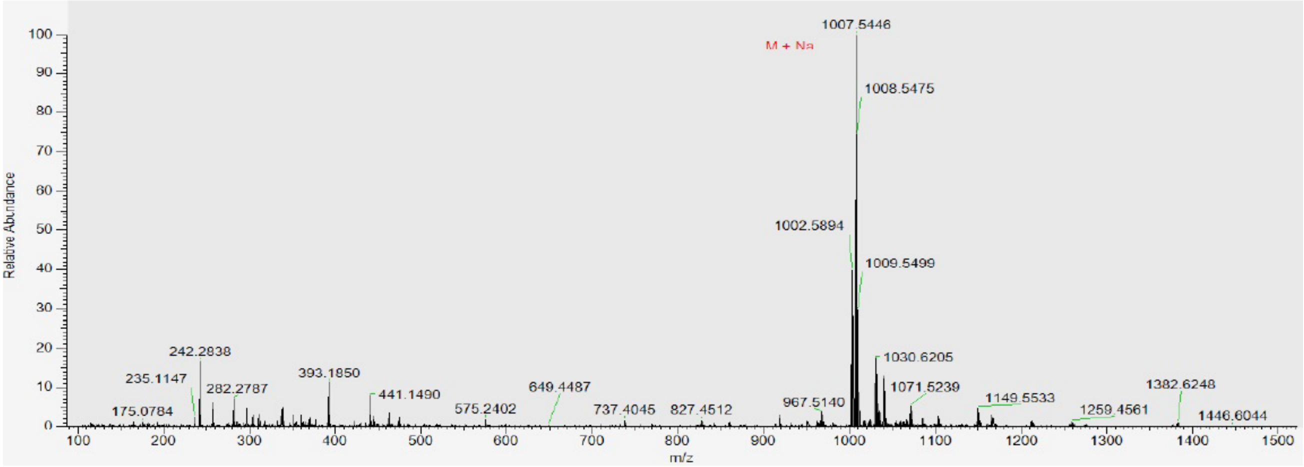

Figure S43 ESI-HRMS spectrum of **4c.**

Compound 16

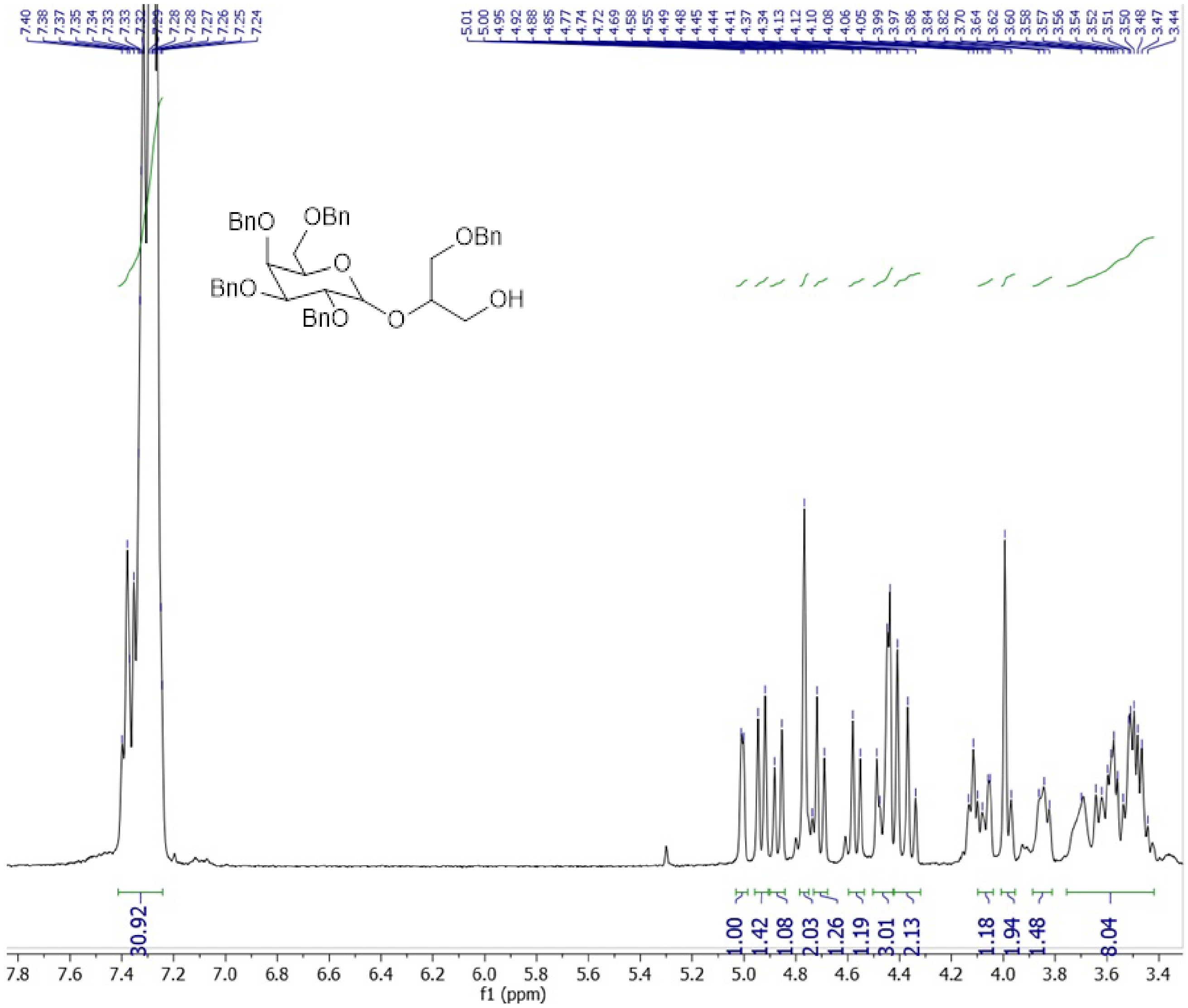

Figure S44 ^{1}H NMR ($CDCl_3$) spectrum of **16** (α-anomer).

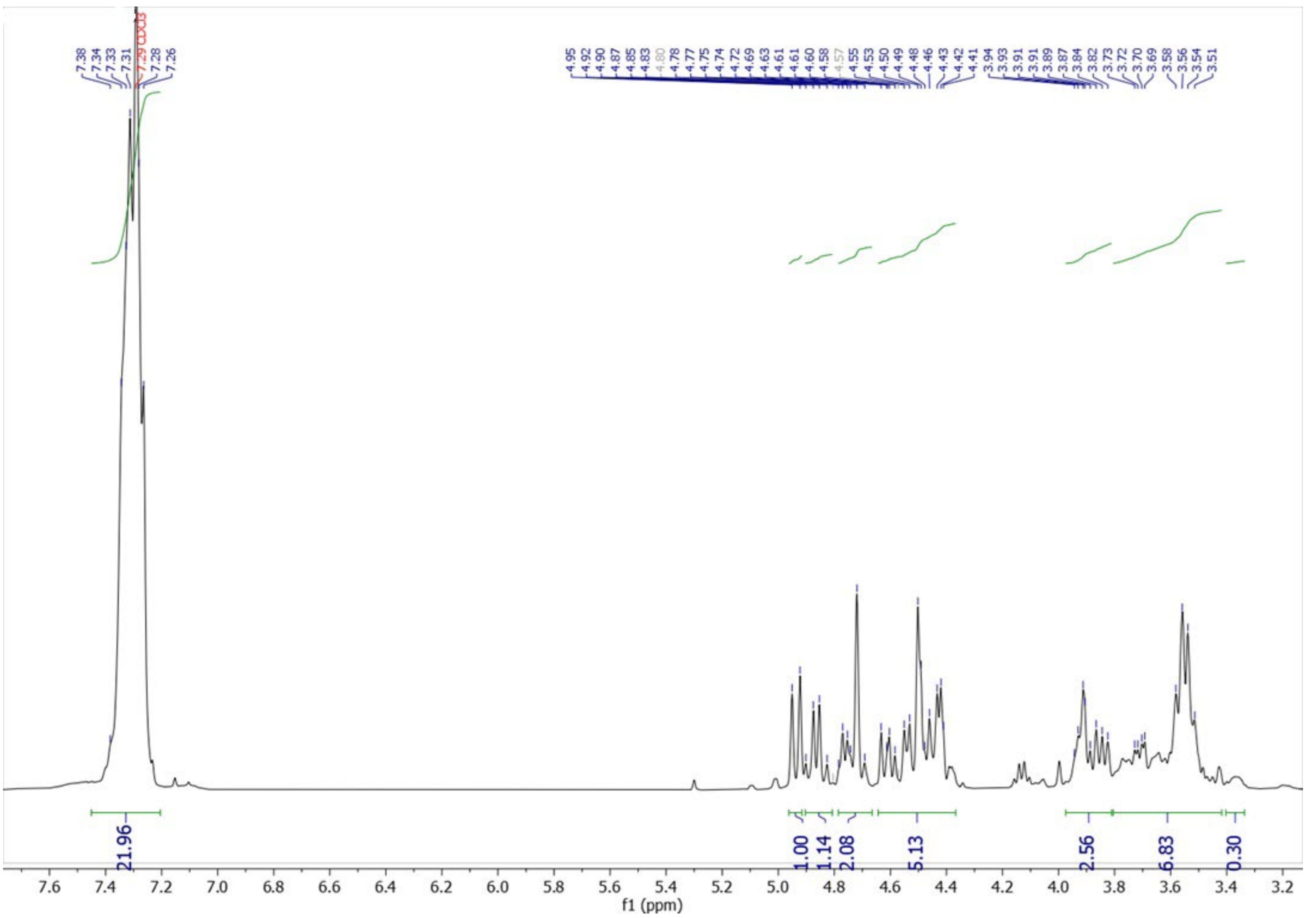

Figure S45 ^{1}H NMR ($CDCl_3$) spectrum of **16** (*β*-anomer).

Compound 17

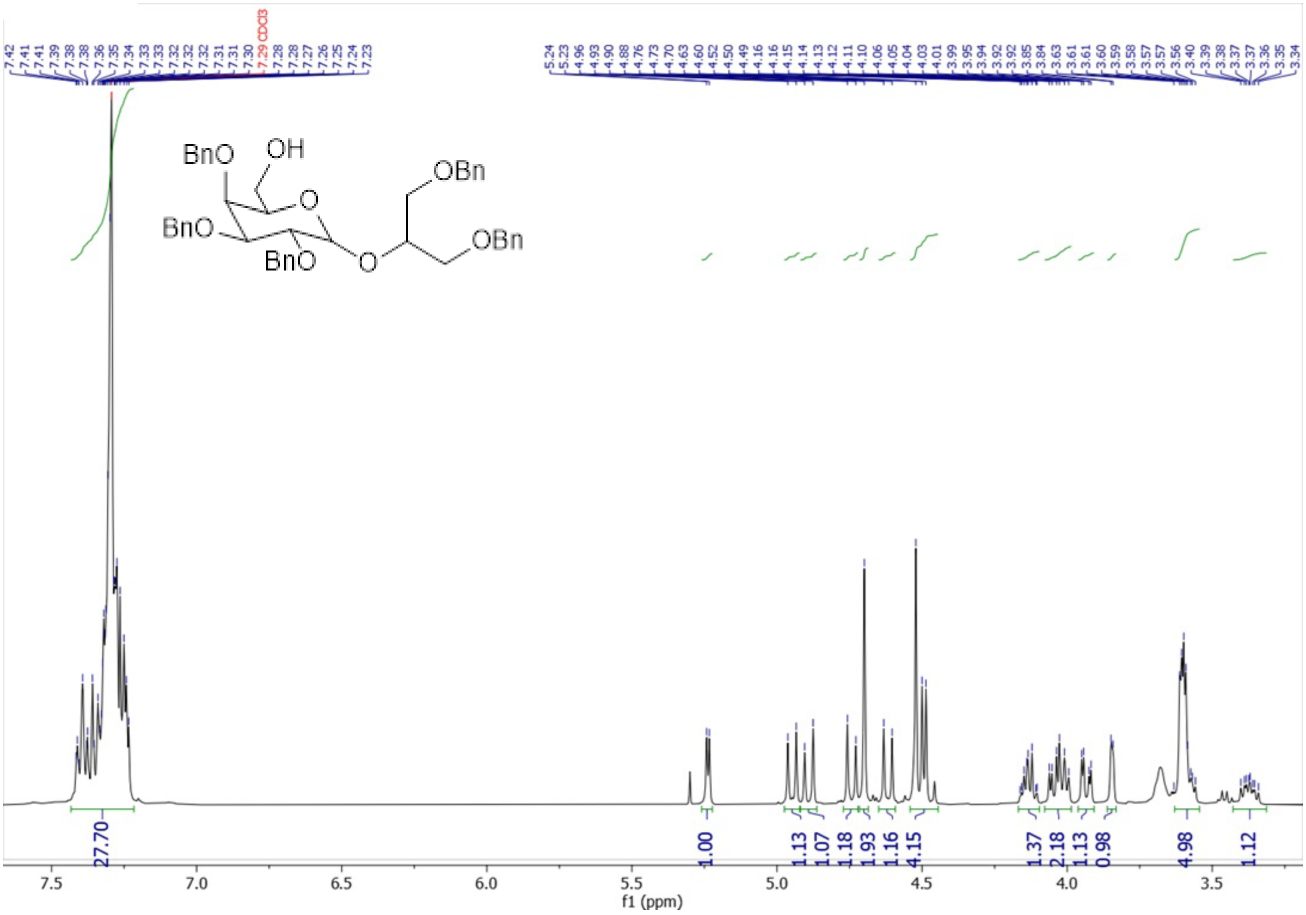

Figure S46 ^{1}H NMR (CDCl$_3$) spectrum of **17** (*α*-anomer).

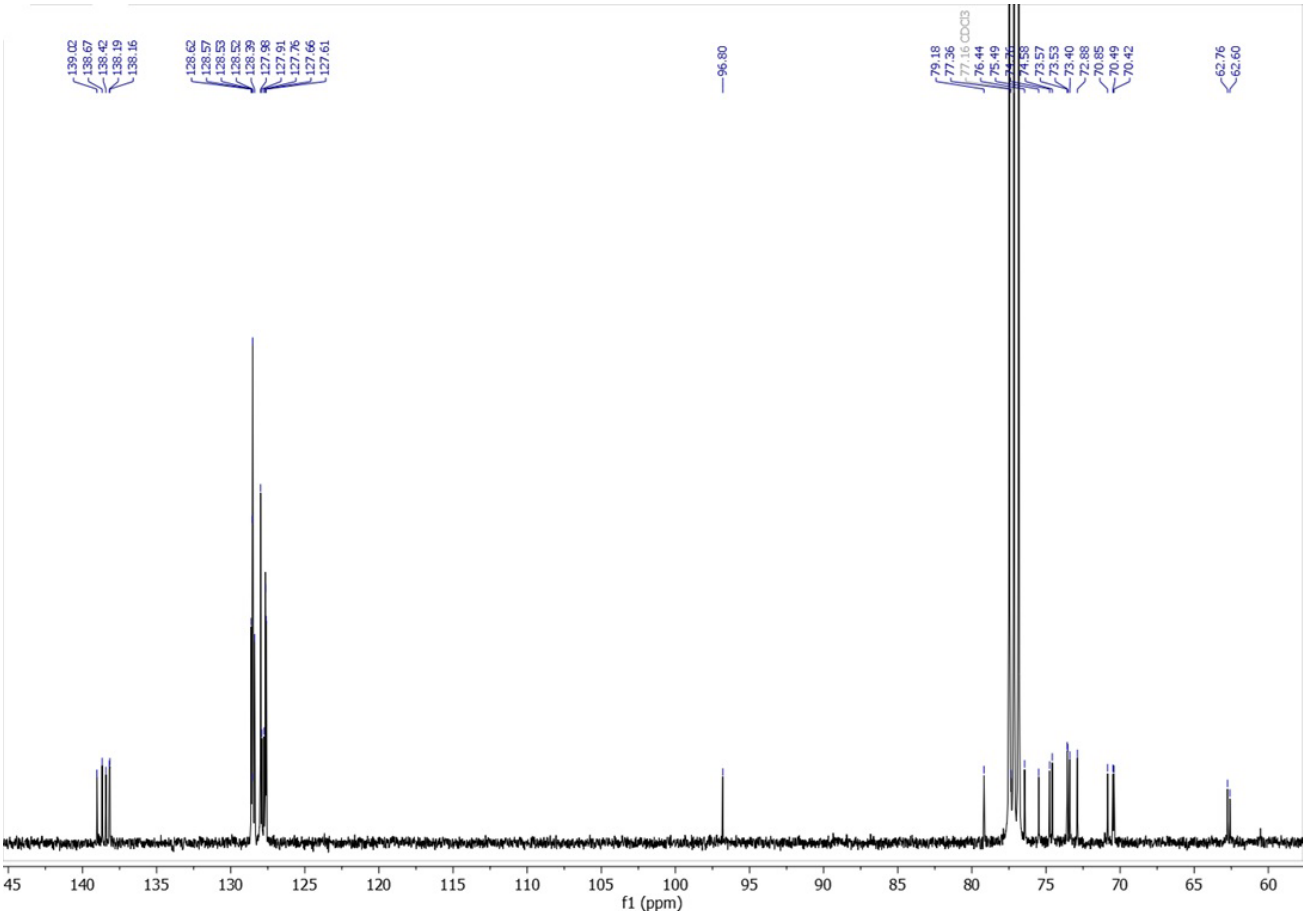

Figure S47 ^{13}C NMR ($CDCl_3$) spectrum of **17** (*α*-anomer).

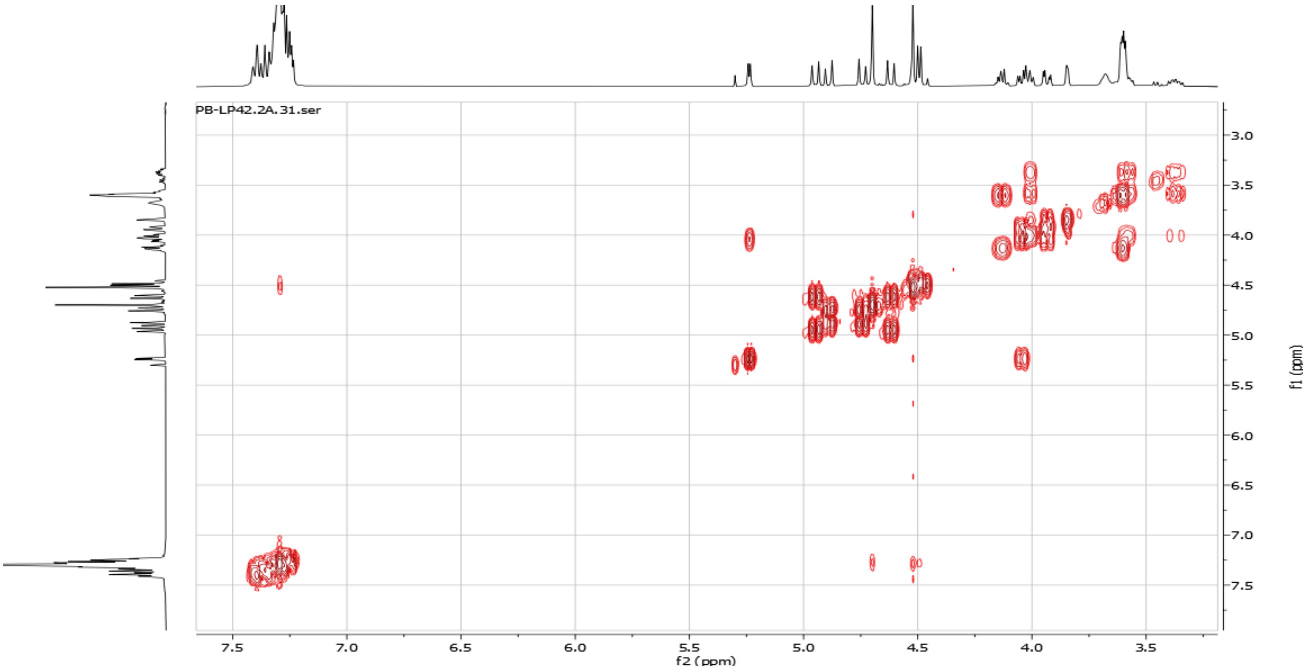

Figure S48 2D NMR COSY ($CDCl_3$) spectrum of **17** (*α*-anomer).

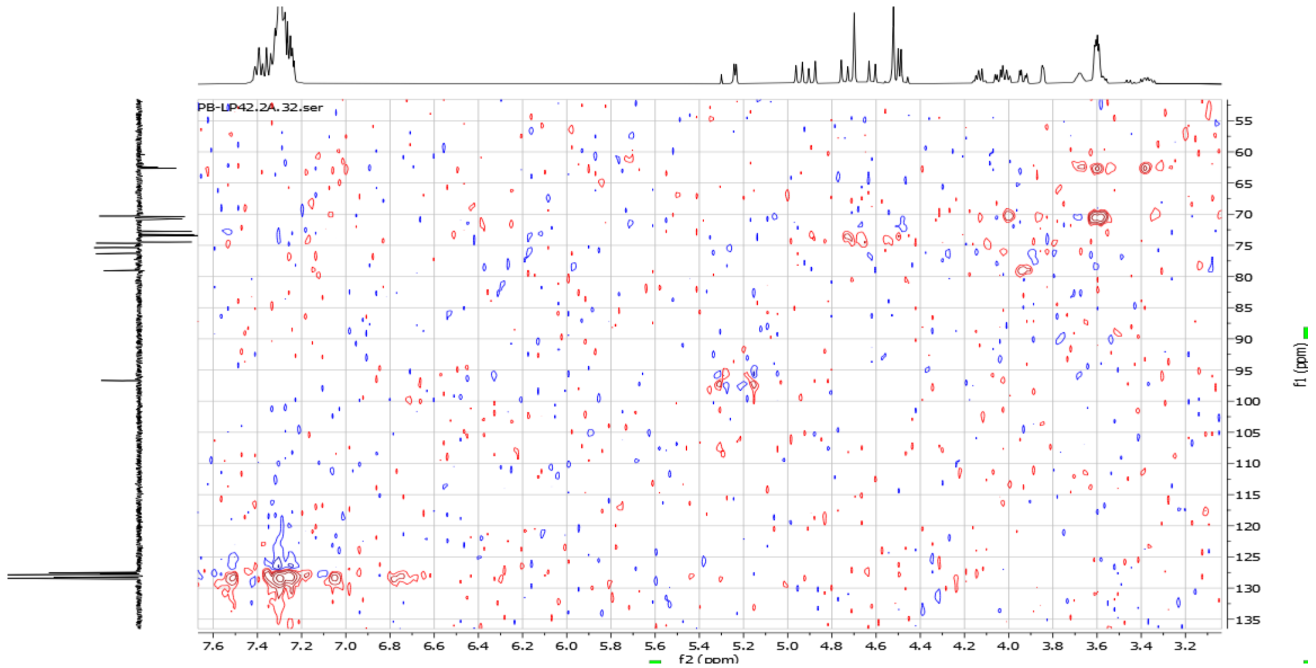

Figure S49 2D NMR HSQC ($CDCl_3$) spectrum of **17** (*α*-anomer).

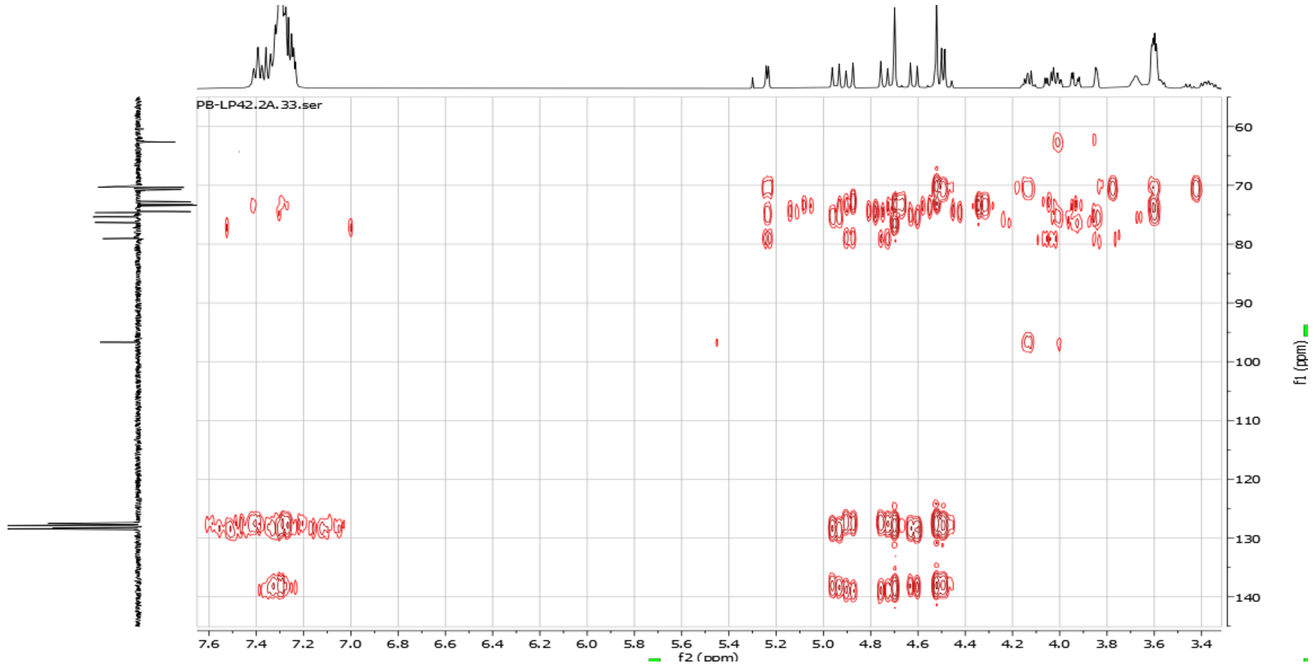

Figure S50 2D NMR HMBC ($CDCl_3$) spectrum of **17** (*α*-anomer).

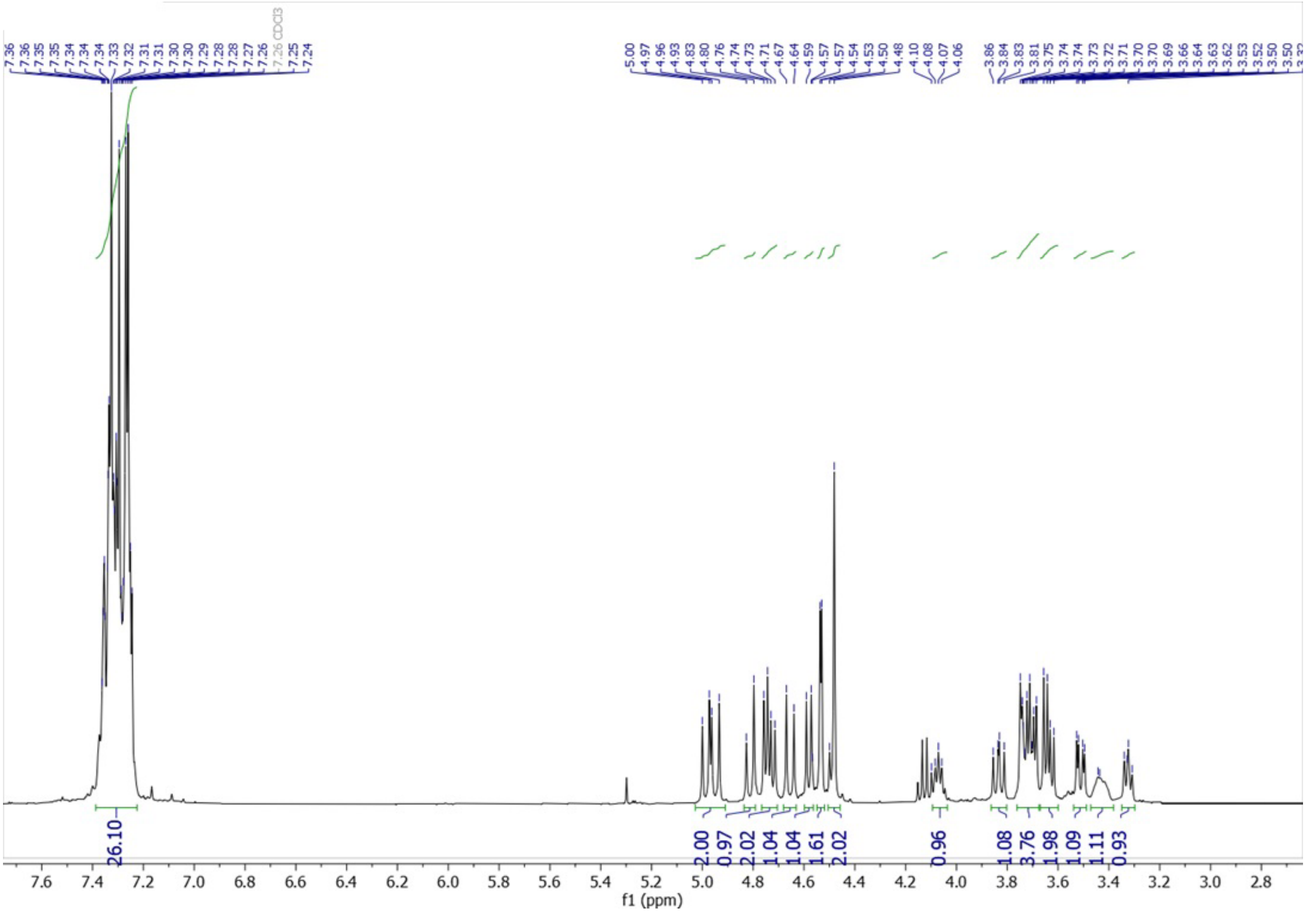

Figure S51 ^{1}H NMR ($CDCl_3$) spectrum of **17** (*β*-anomer).

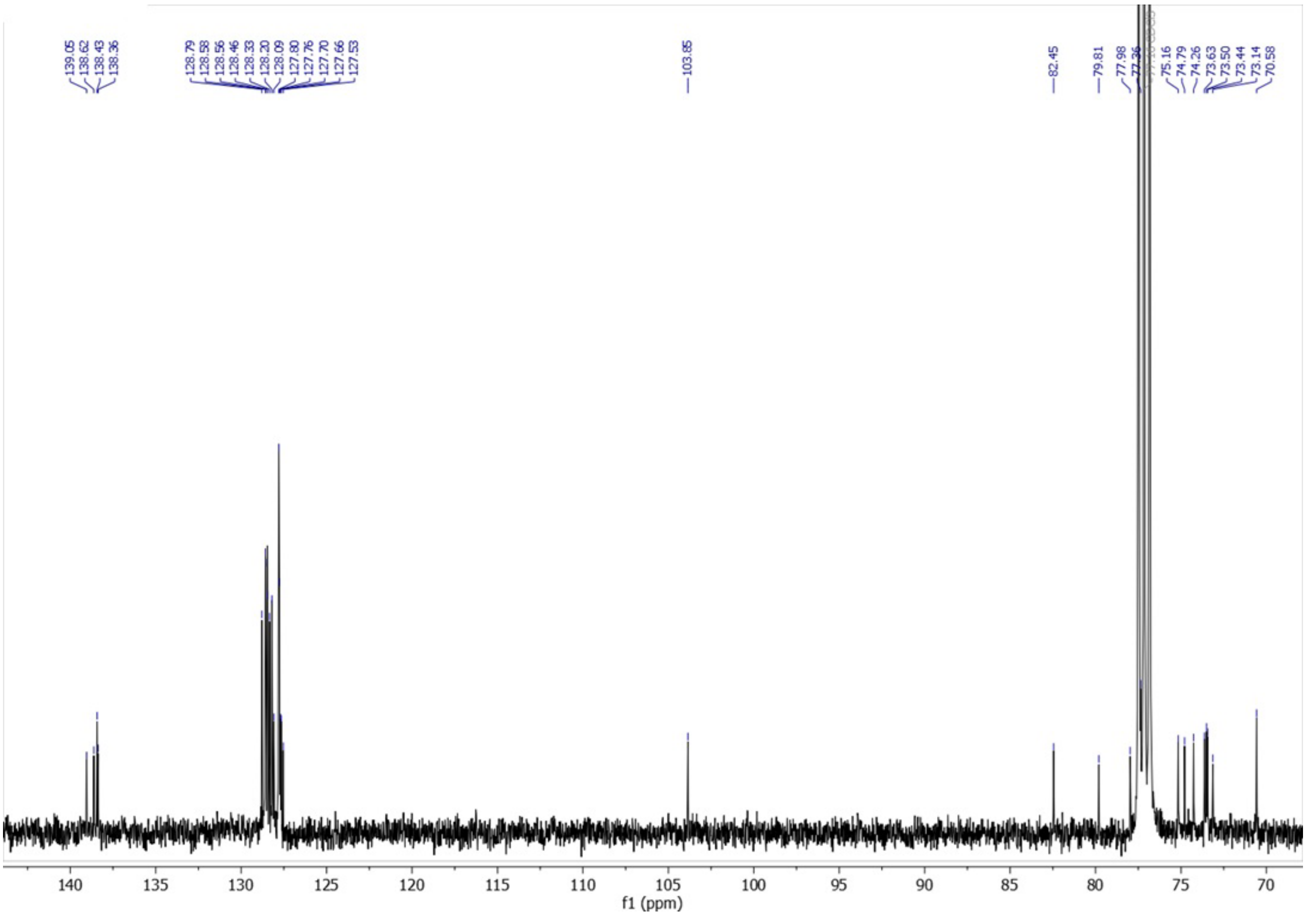

Figure S52 ^{13}C NMR ($CDCl_3$) spectrum of **17** (β-anomer).

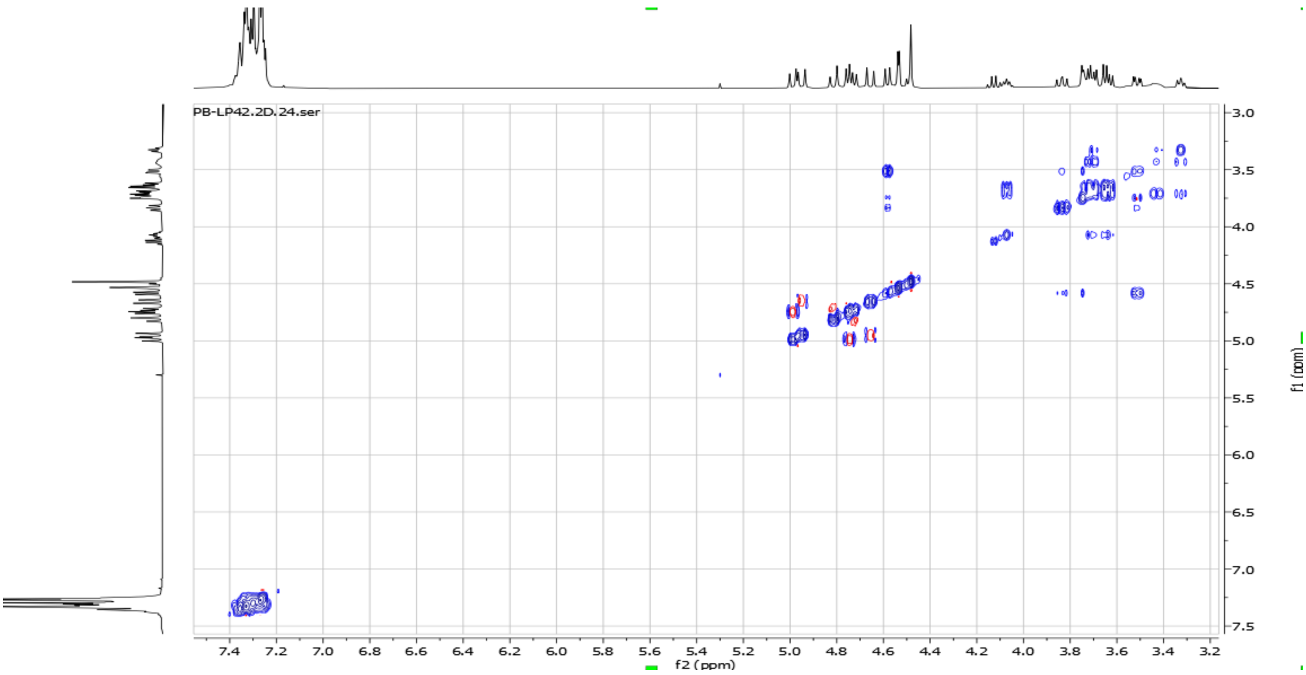

Figure S53 2D NMR COSY ($CDCl_3$) spectrum of **17** (β-anomer).

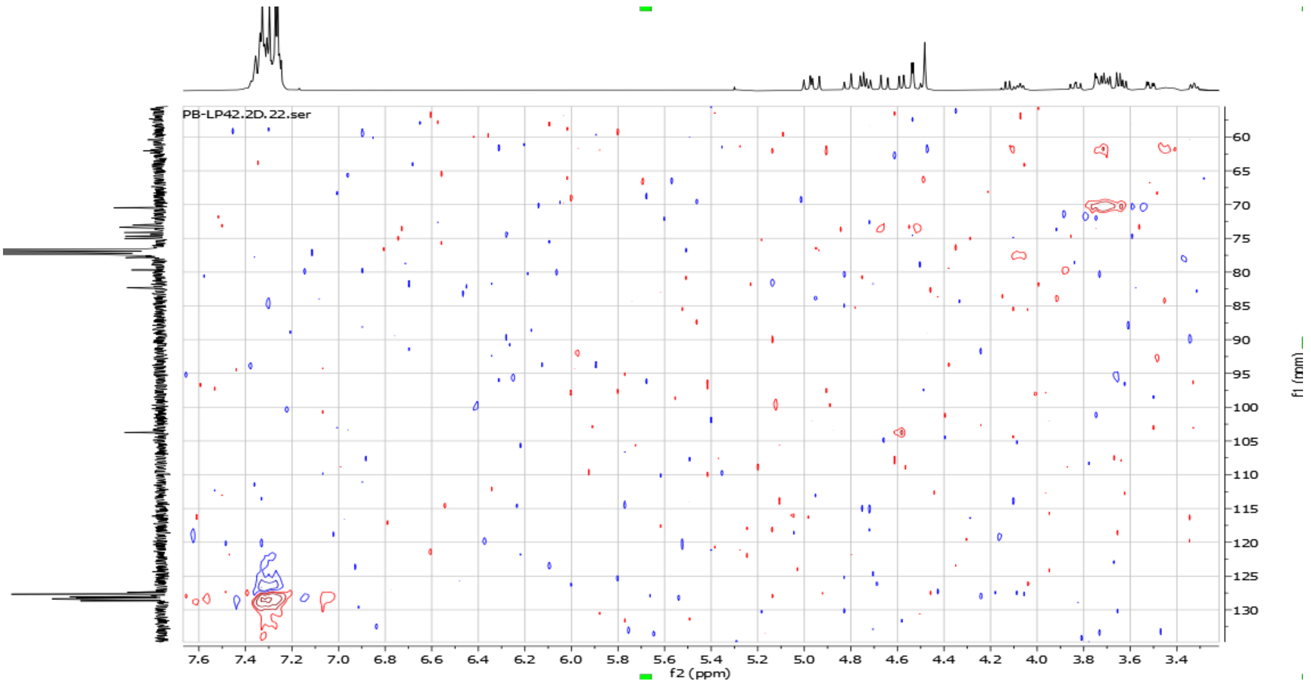

Figure S54 2D NMR HSQC ($CDCl_3$) spectrum of **17** (β-anomer).

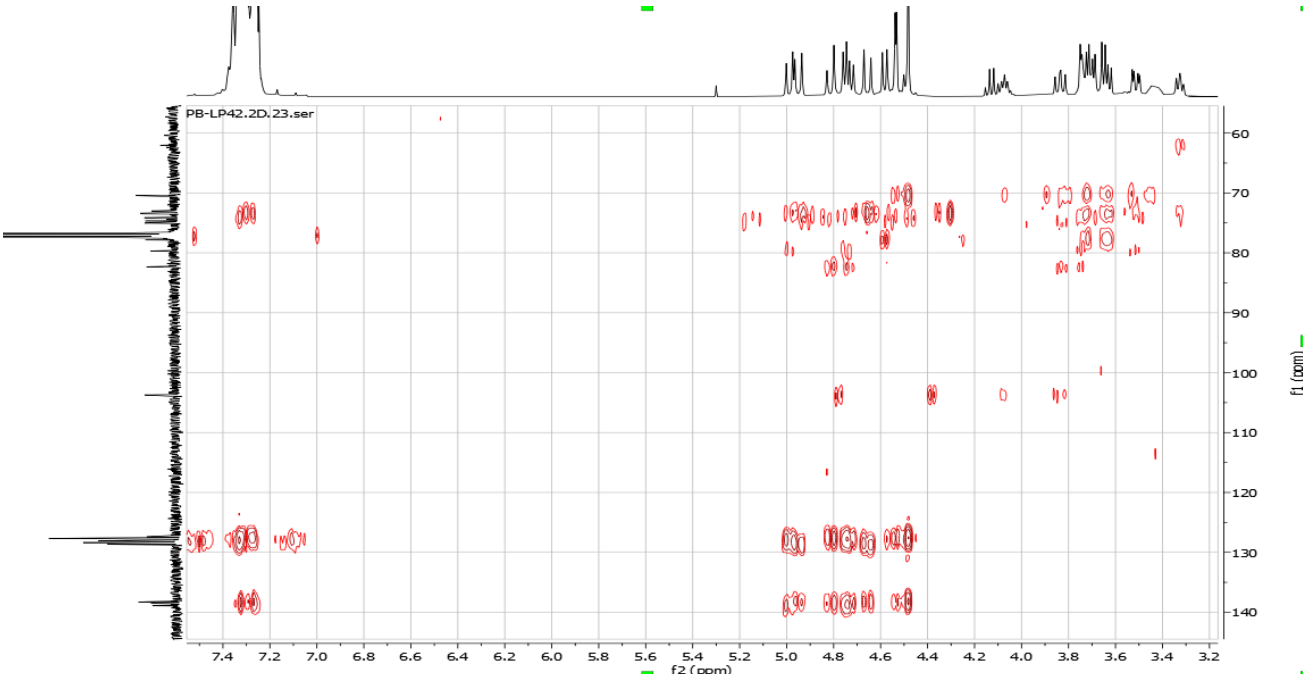

Figure S55 2D NMR HMBC ($CDCl_3$) spectrum of **17** (β-anomer).

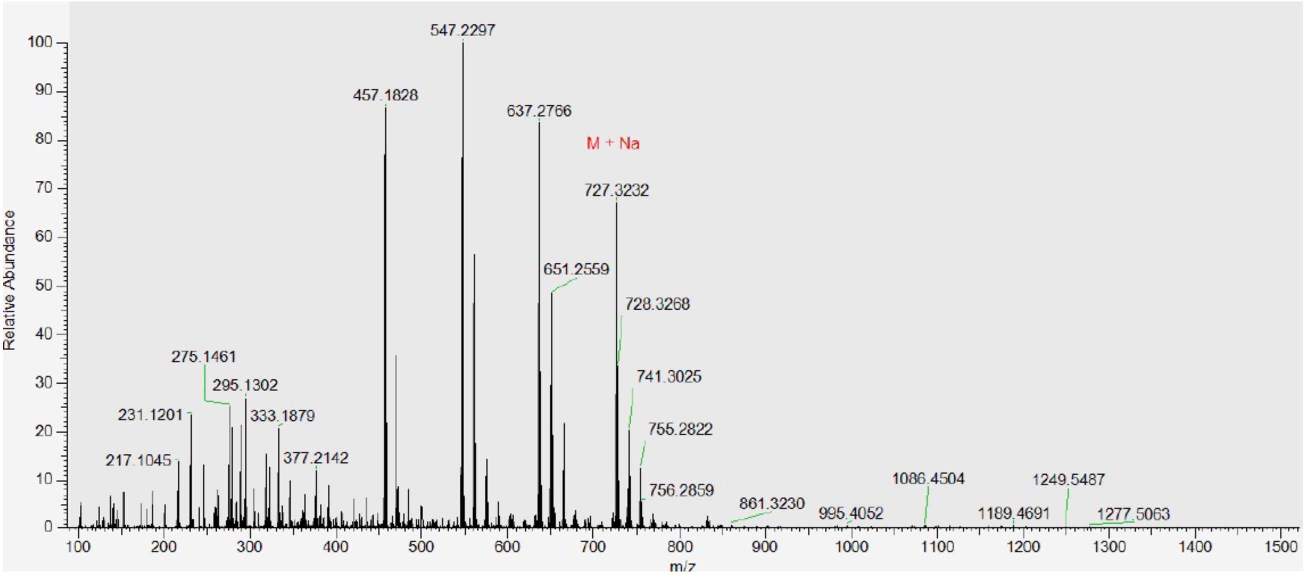

Figure S56 ESI-HRMS spectrum of **17.**

Compound 18

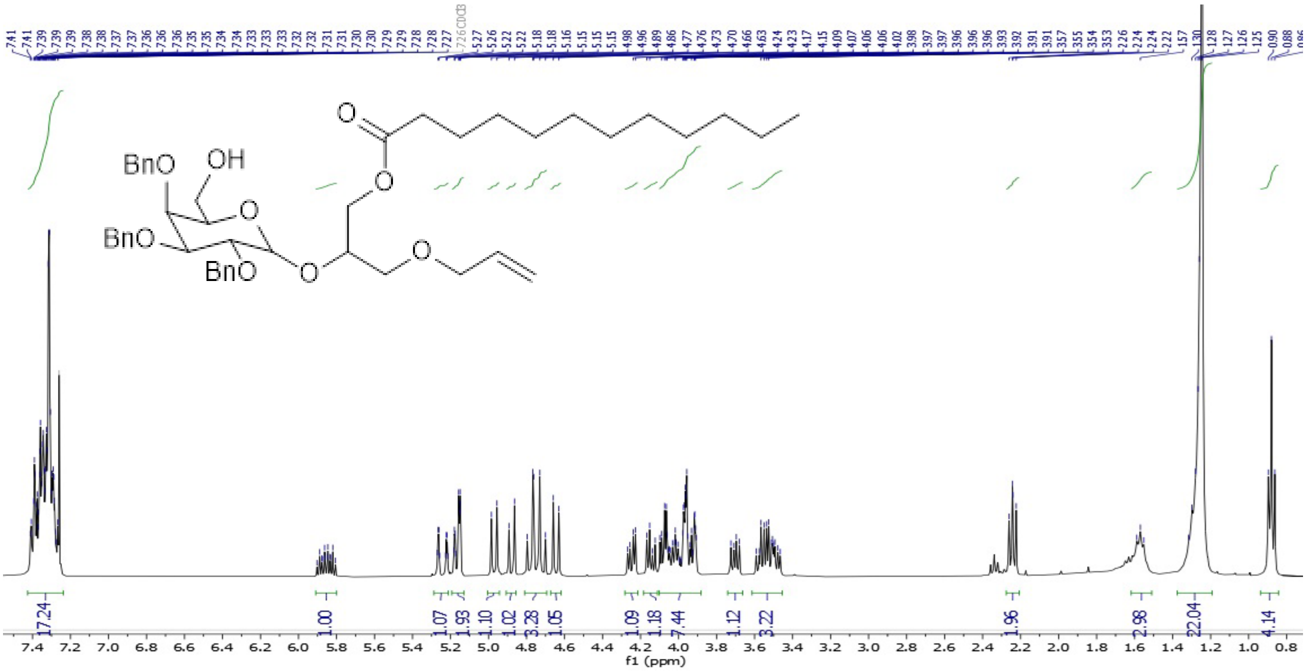

Figure S57 ^{1}H NMR (CDCl$_3$) spectrum of **18** (α-anomer).

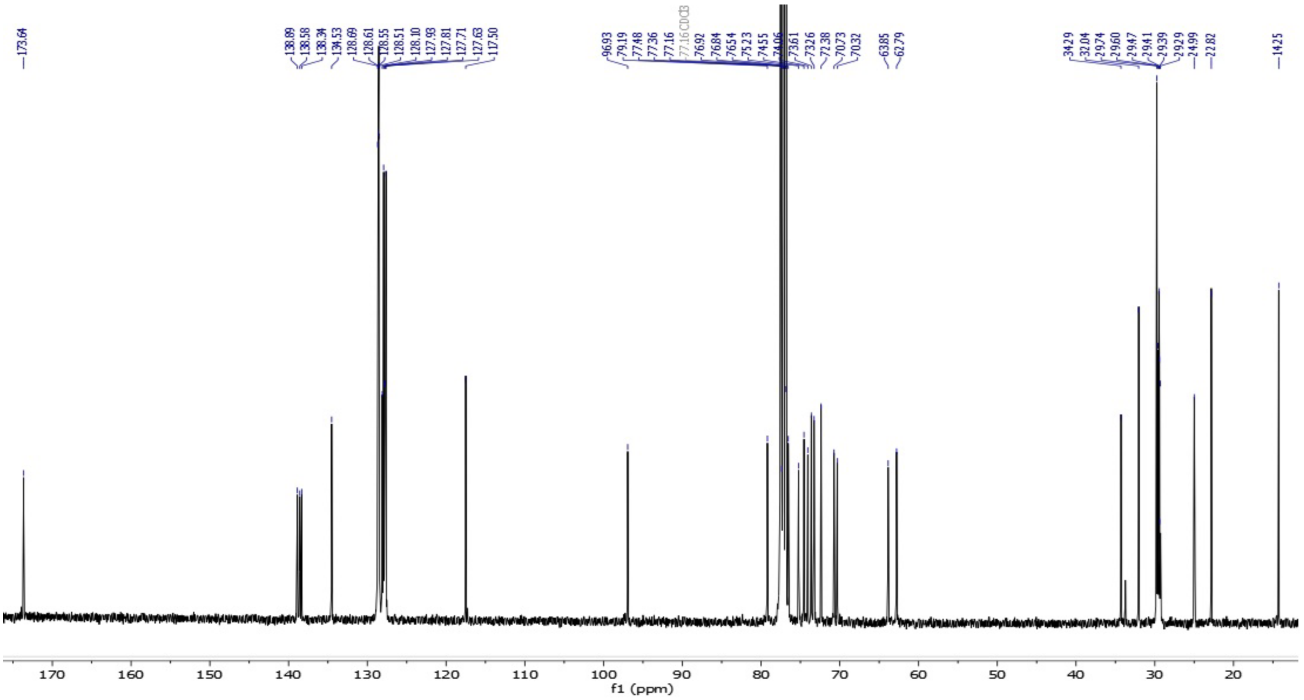

Figure S58 ^{13}C NMR (CDCl$_3$) spectrum of **18** (α-anomer).

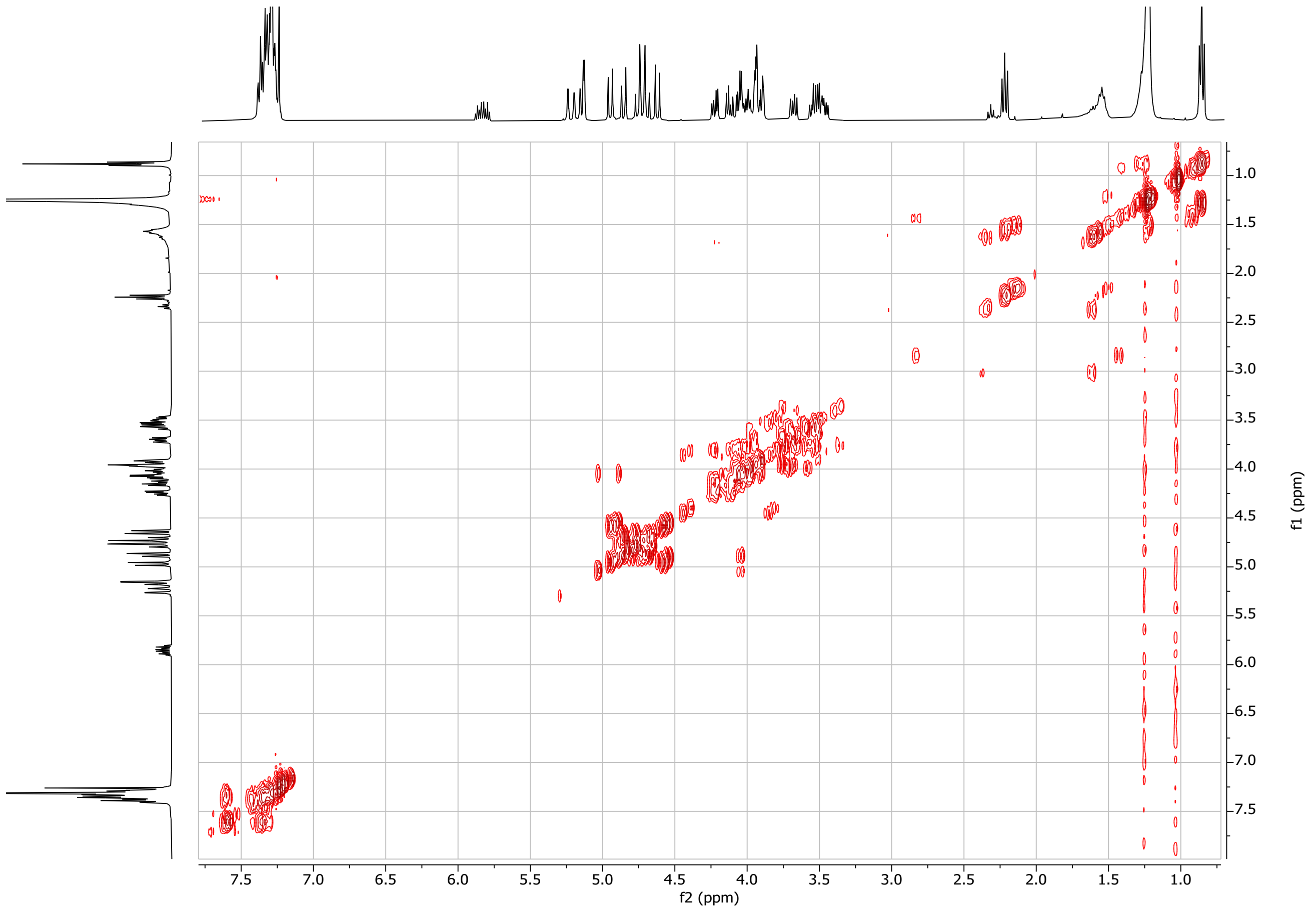

Figure S59 2D NMR COSY ($CDCl_3$) spectrum of **18** (α-anomer).

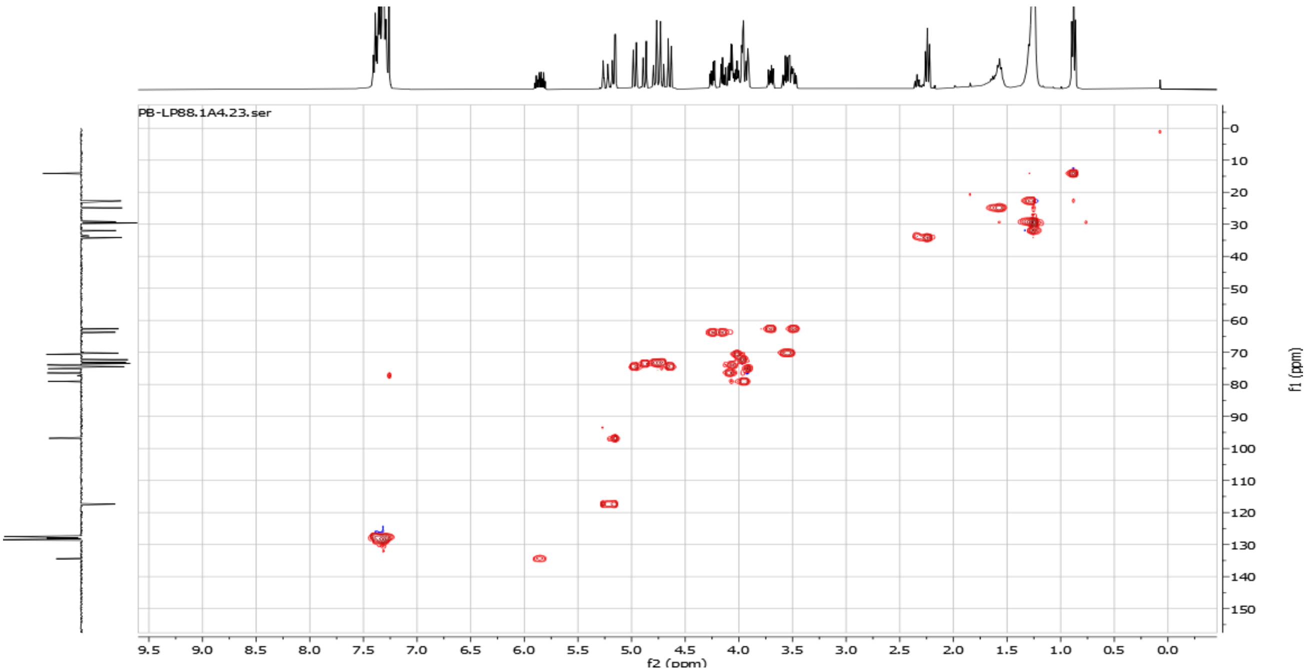

Figure S60 2D NMR HSQC ($CDCl_3$) spectrum of **18** (α-anomer).

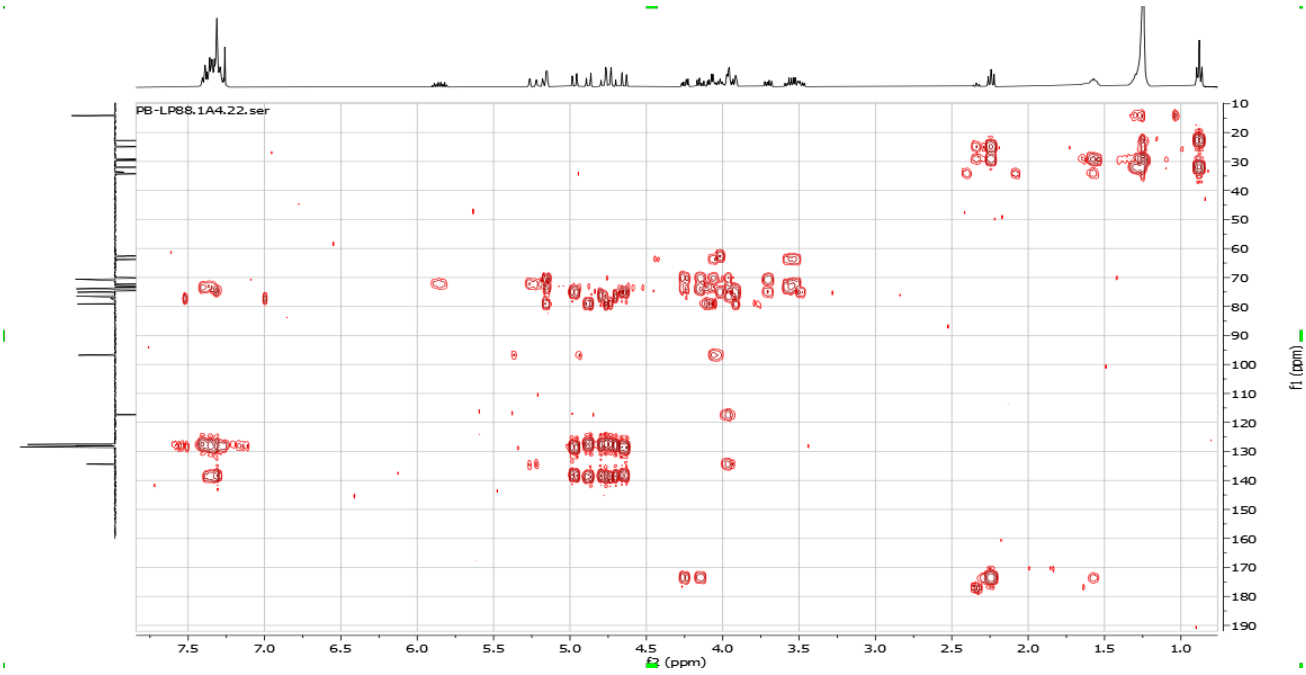

Figure S61 2D NMR HMBC ($CDCl_3$) spectrum of **18** (*α*-anomer).

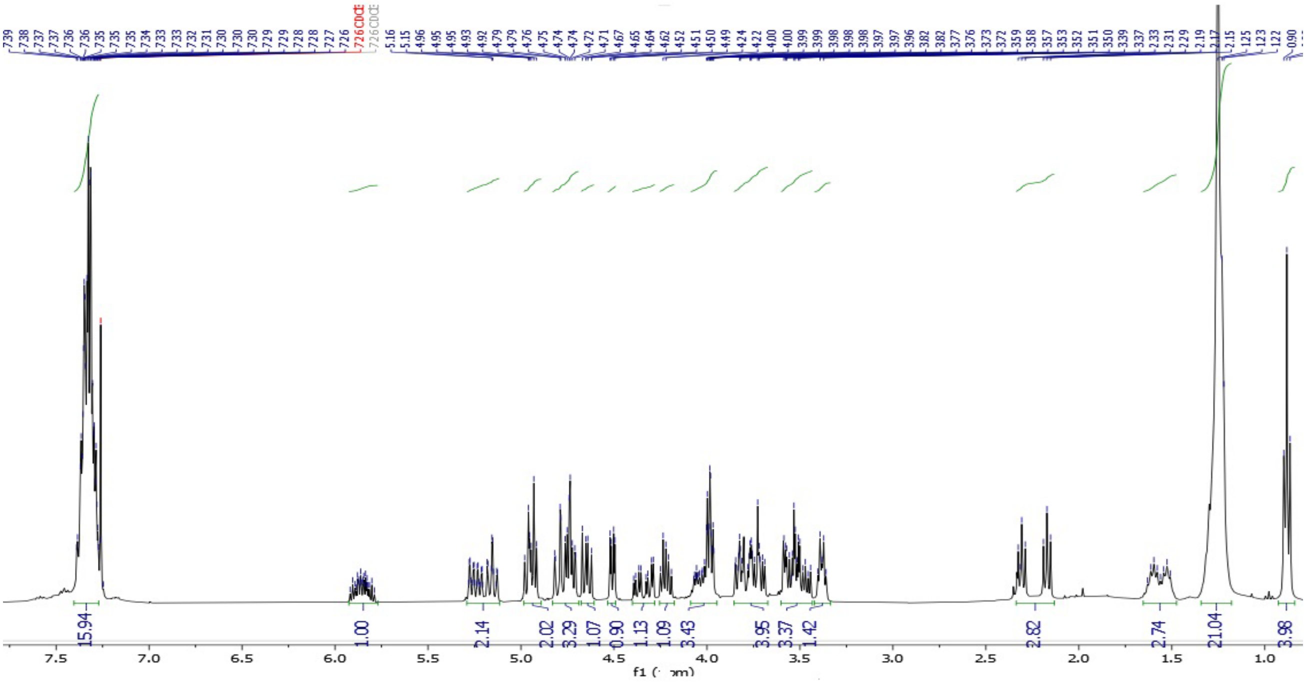

Figure S62 ^1H NMR ($CDCl_3$) spectrum of **18** (*β*-anomer).

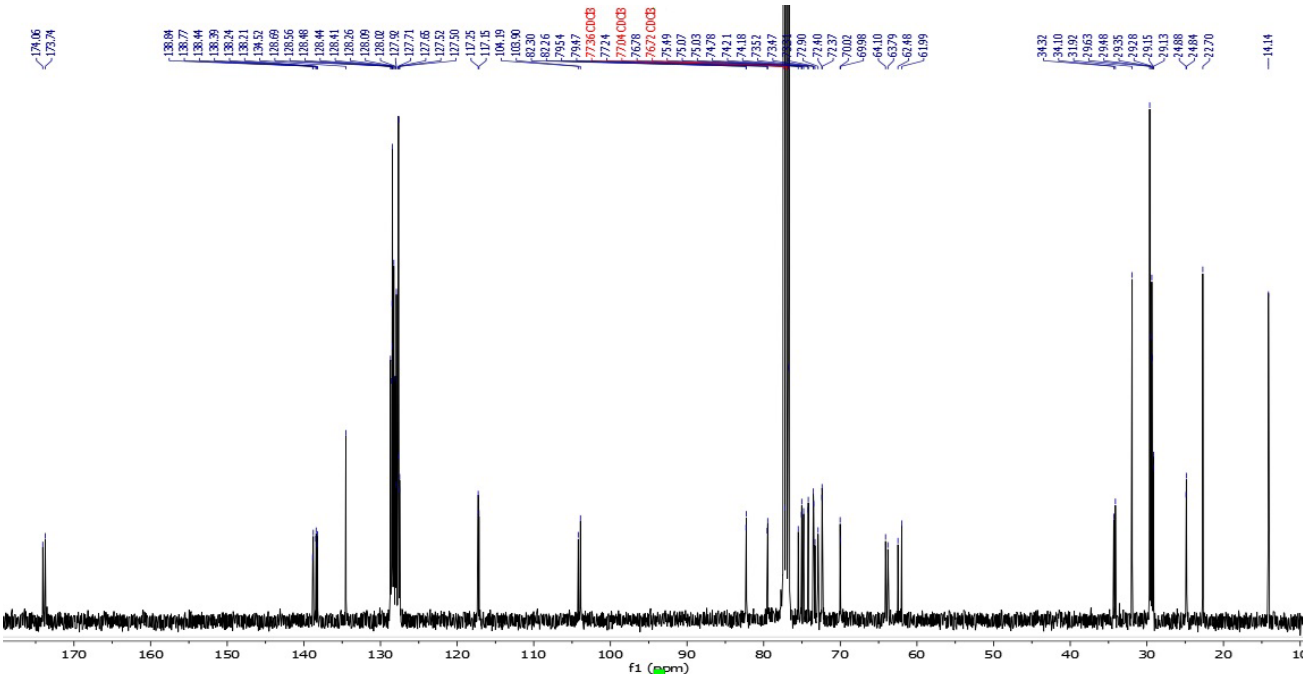

Figure S63 ^{13}C NMR ($CDCl_3$) spectrum of **18** (*β*-anomer).

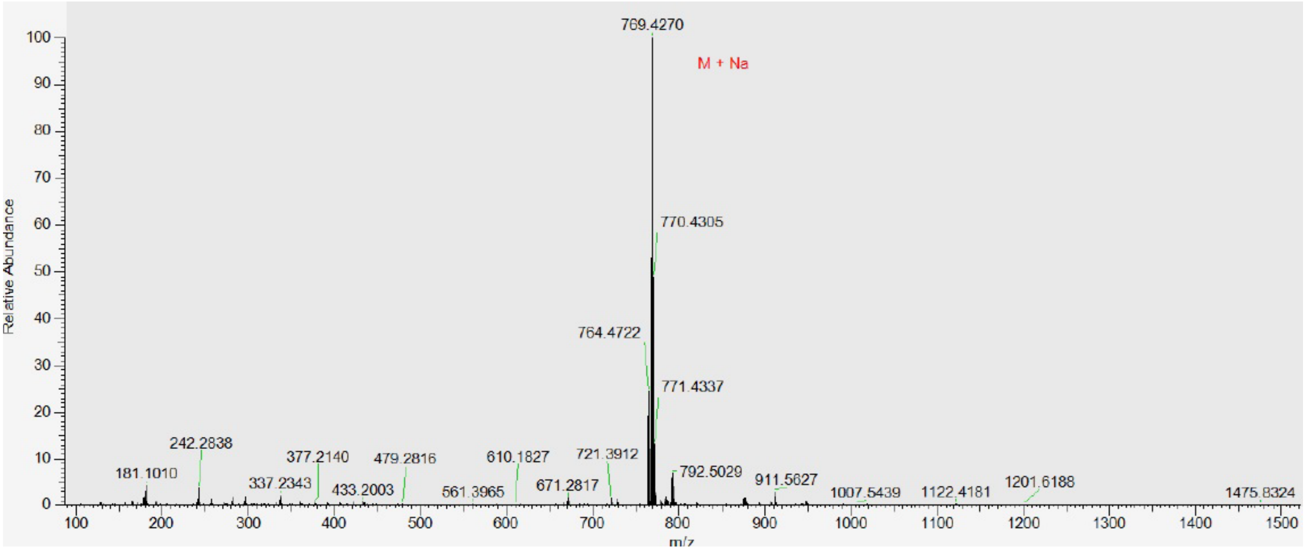

Figure S64 ESI-HRMS spectrum of **18.**

Compound 19

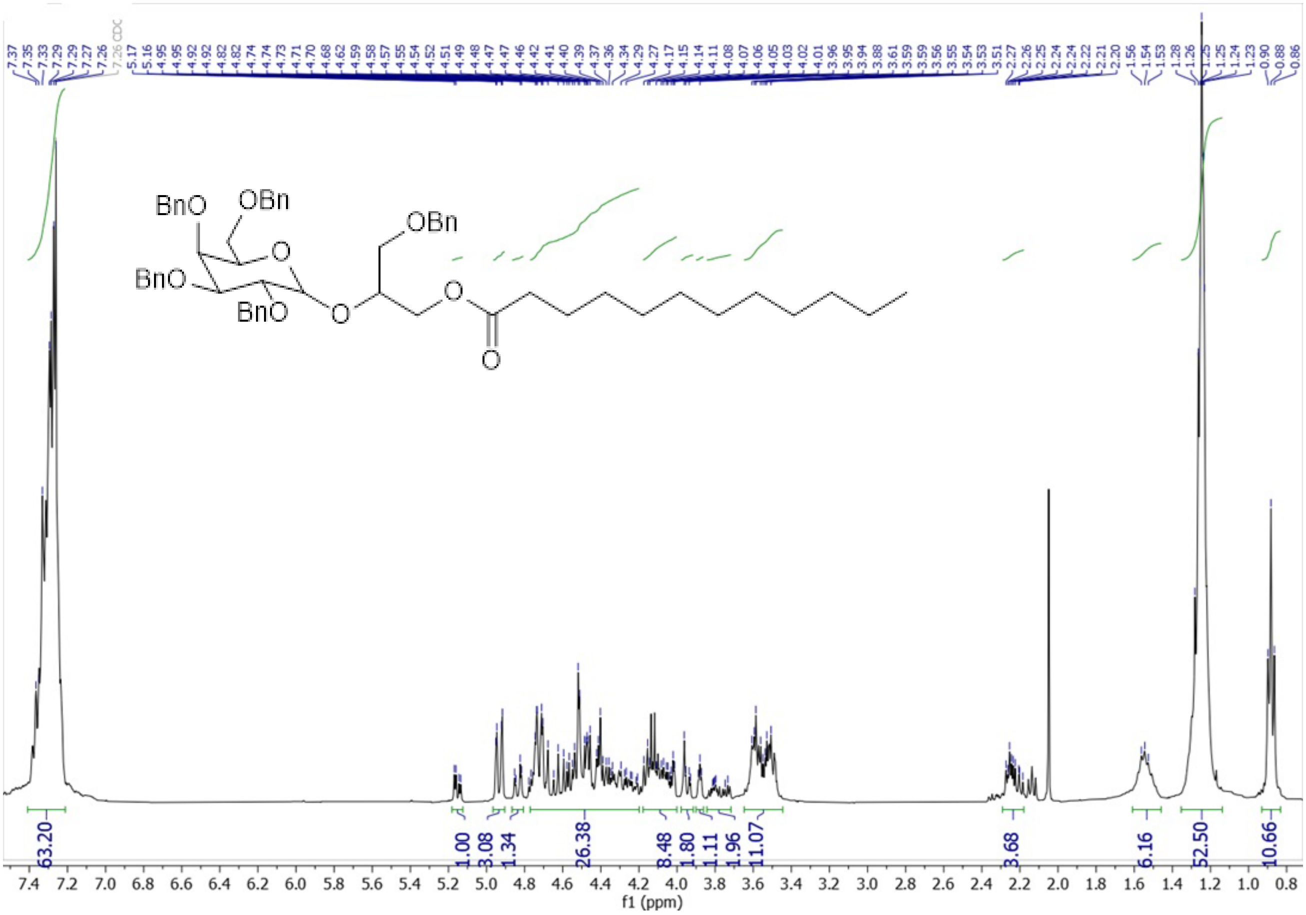

Figure S65 ^{1}H NMR (CDCl$_3$) spectrum of **19.**

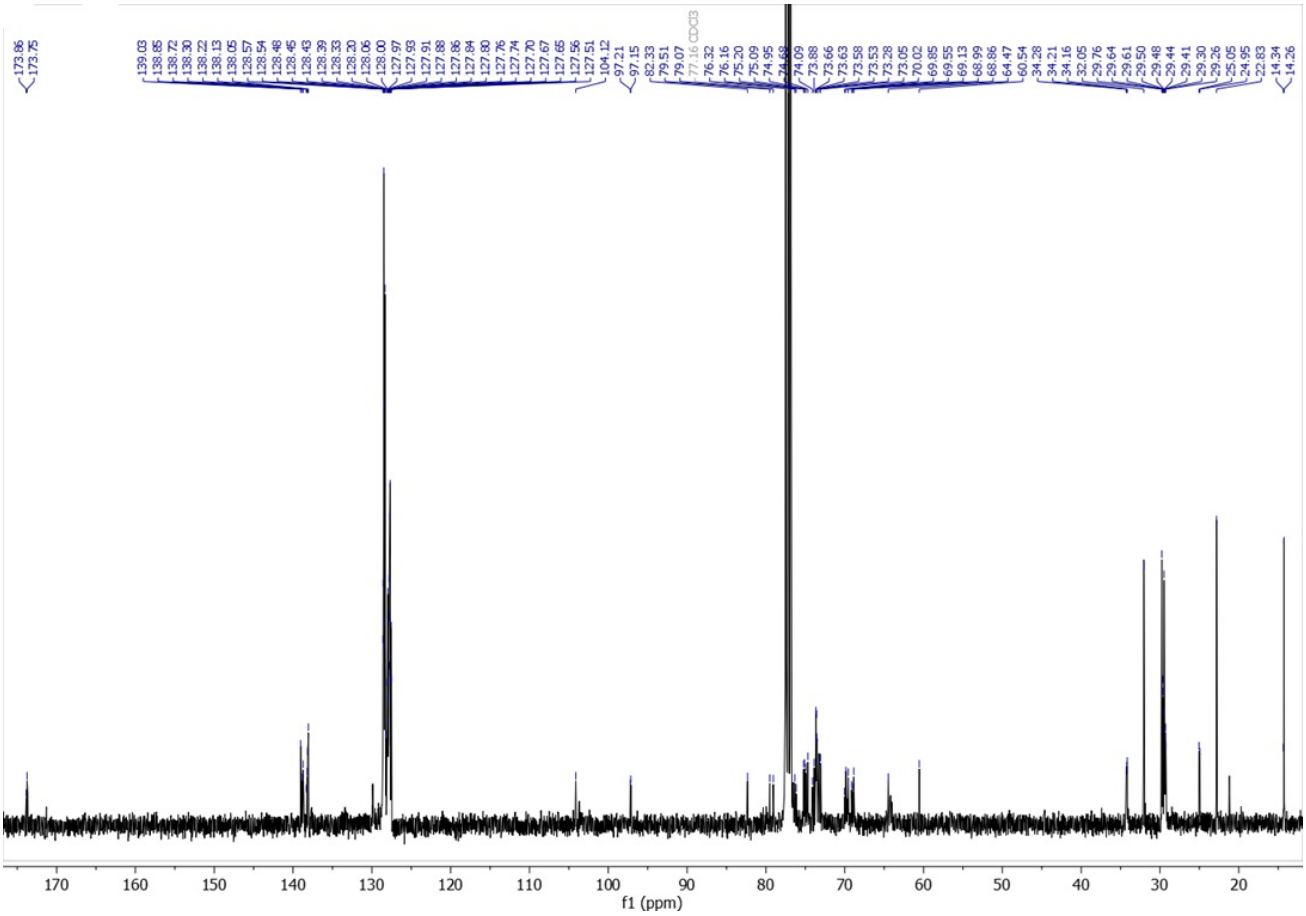

Figure S66 ^{13}C NMR ($CDCl_3$) spectrum of **19.**

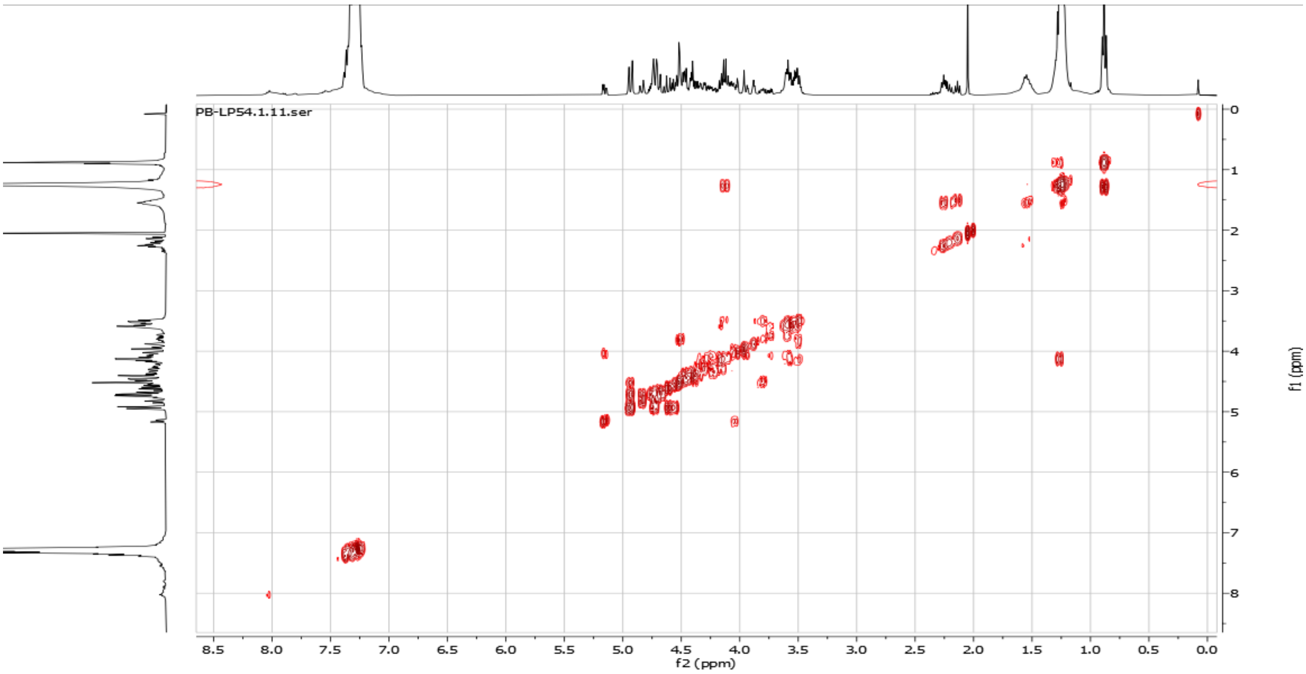

Figure S67 2D NMR COSY ($CDCl_3$) spectrum of **19.**

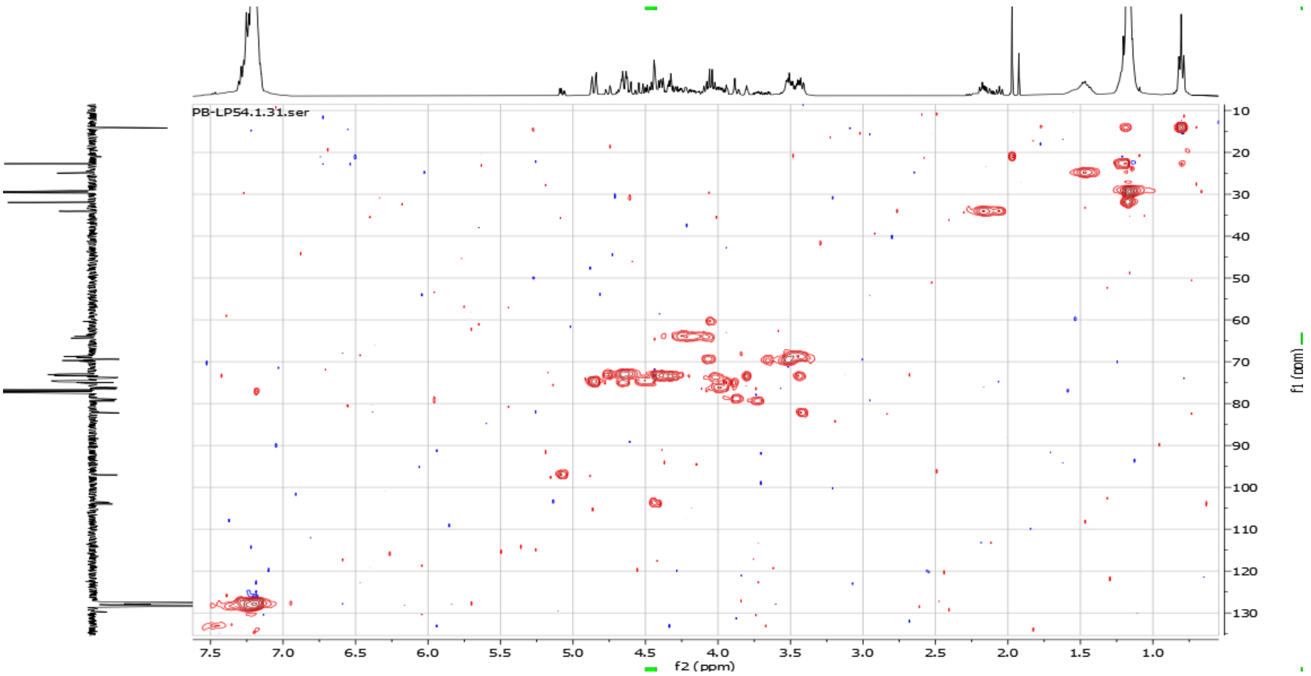

Figure S68 2D NMR HSQC ($CDCl_3$) spectrum of **19.**

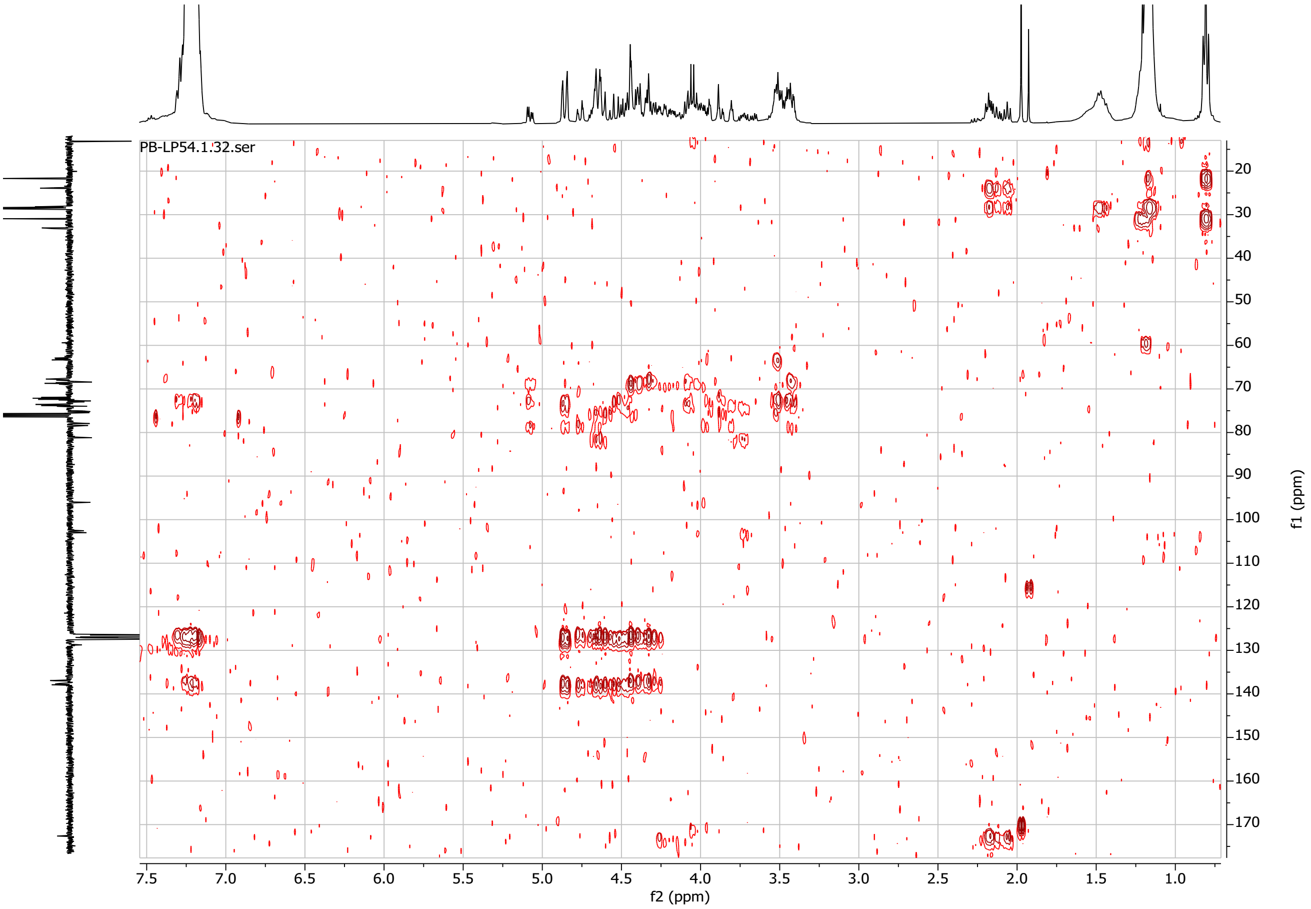

Figure S69 2D NMR HMBC ($CDCl_3$) spectrum of **19.**

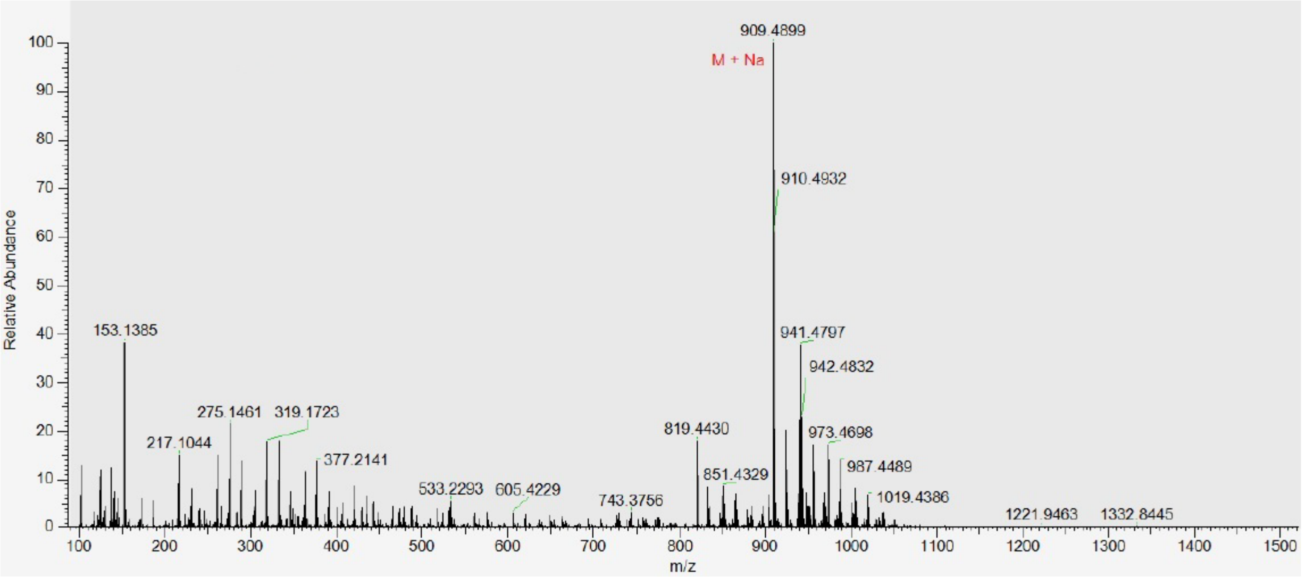

Figure S70 ESI-HRMS spectrum of **19.**

Compound 21

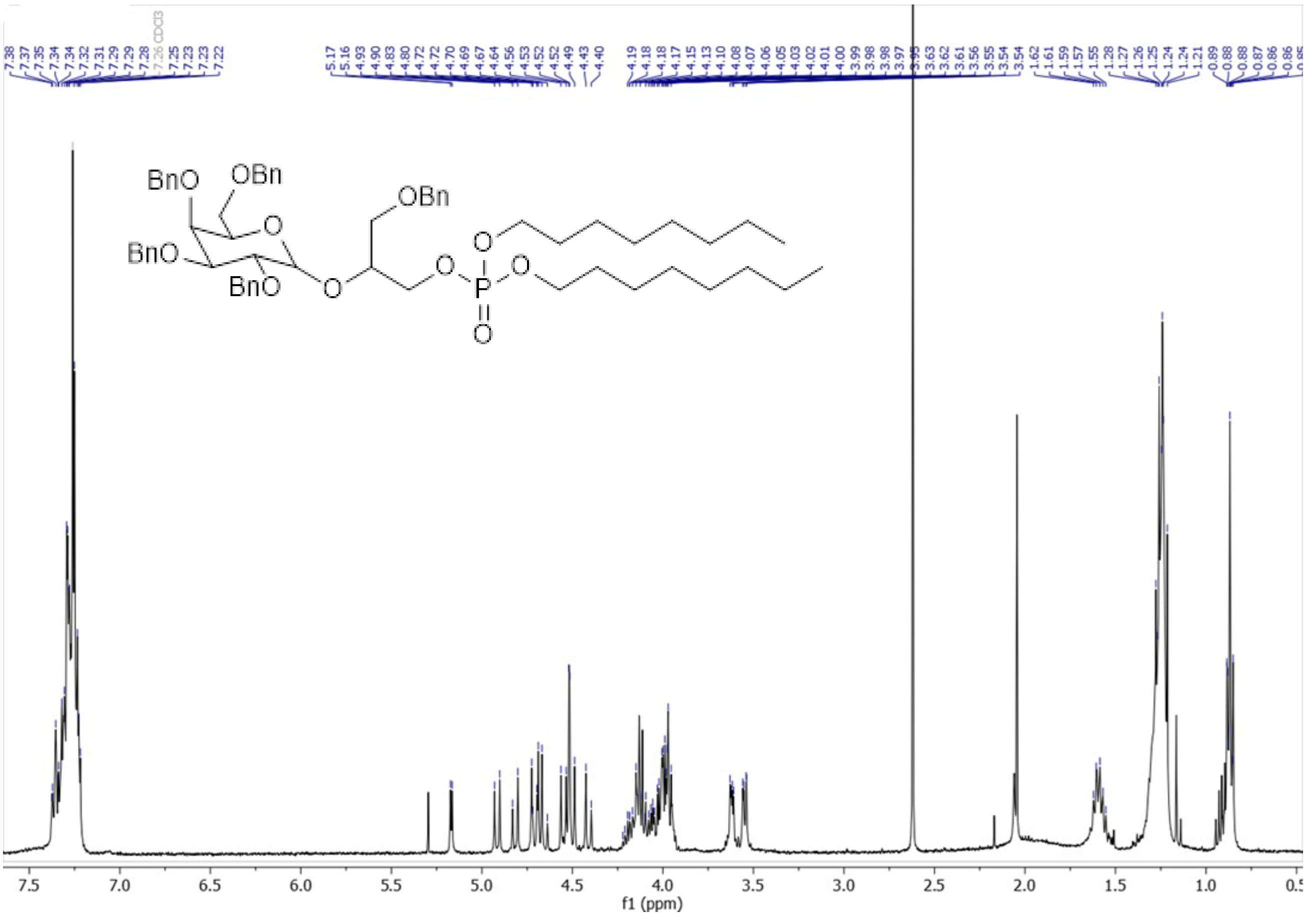

Figure S71 ^{1}H NMR ($CDCl_3$) spectrum of **21.**

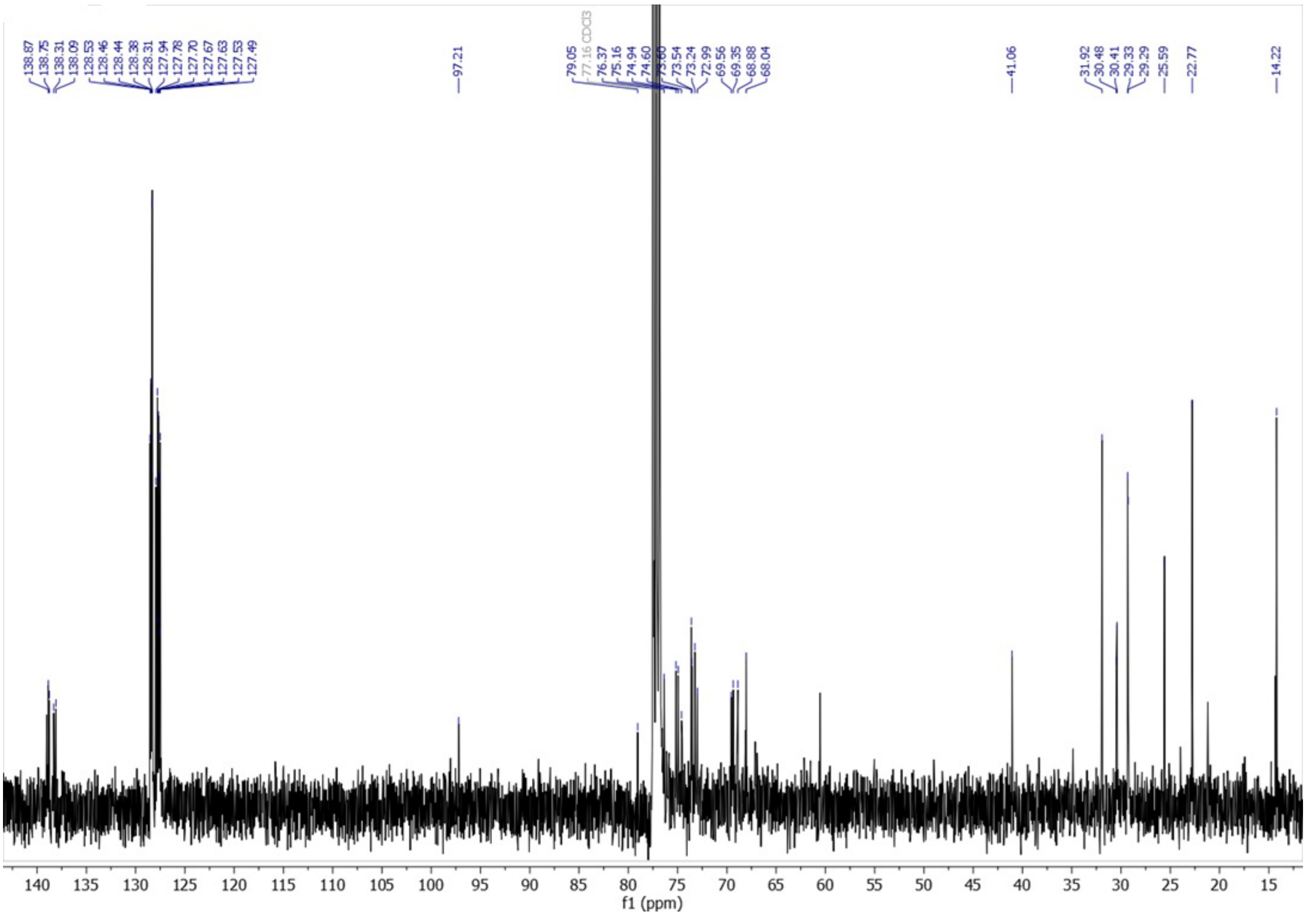

Figure S72 ^{13}C NMR ($CDCl_3$) spectrum of **21.**

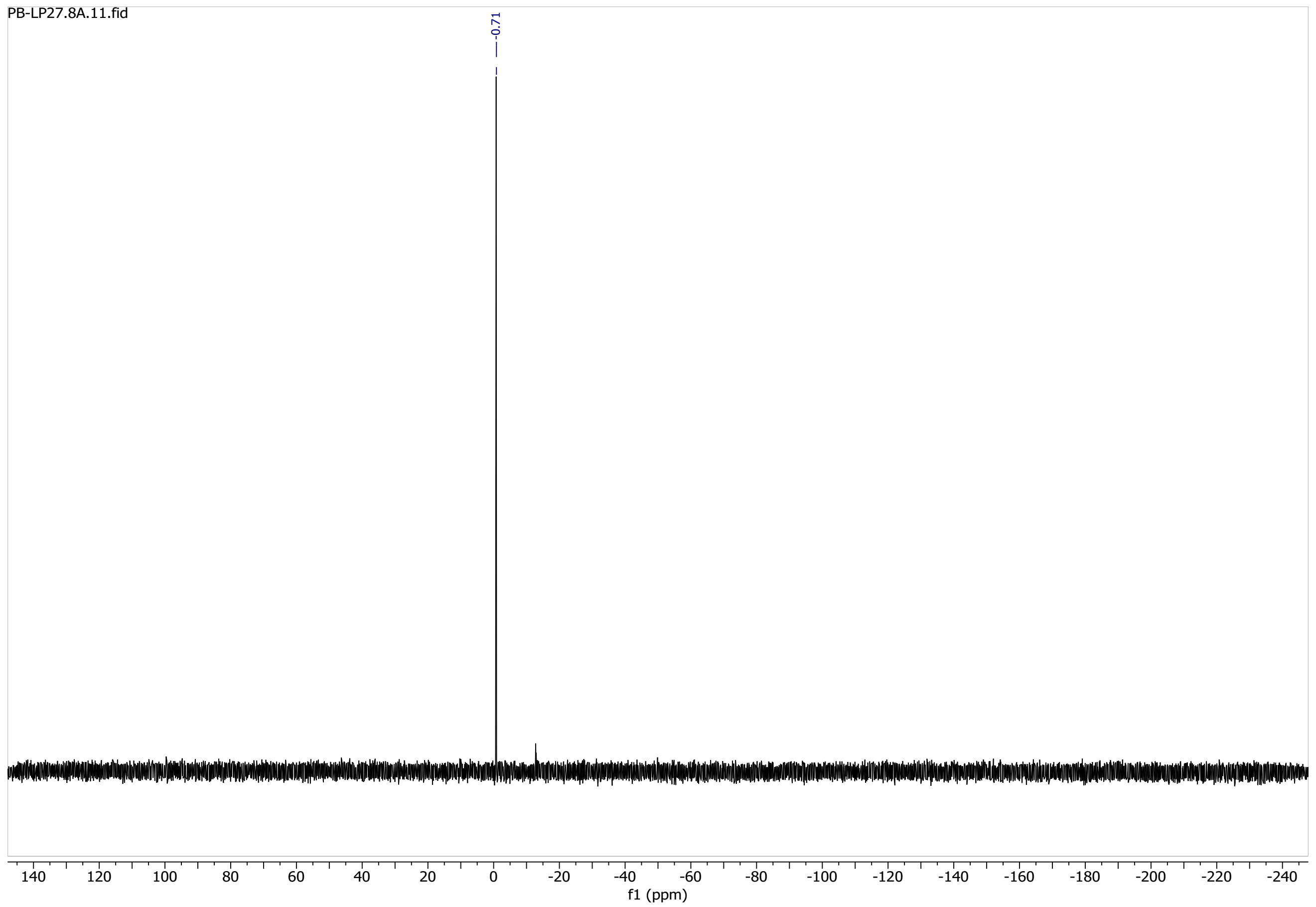

Figure S73 ^{31}P NMR ($CDCl_3$) spectrum of **21.**

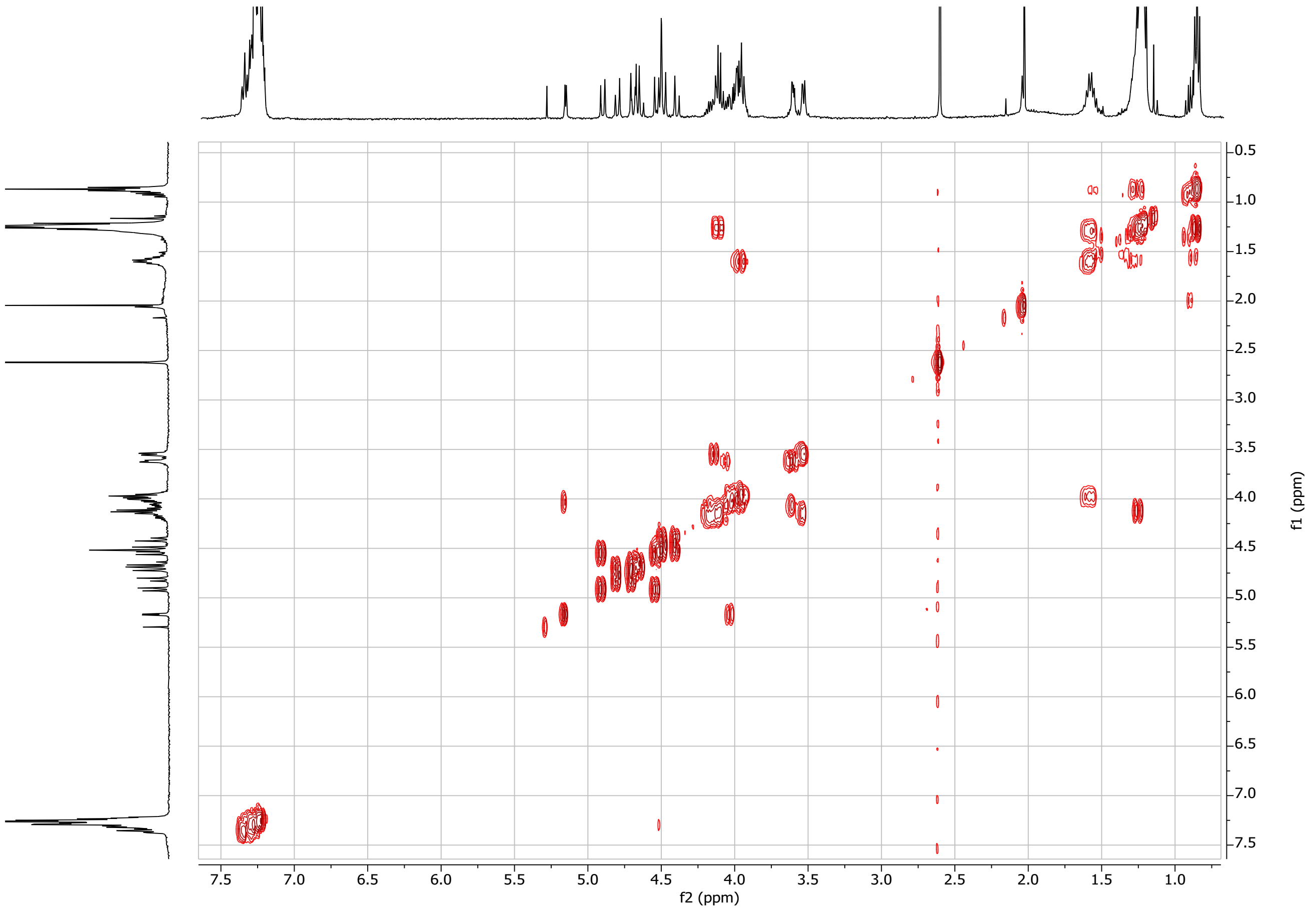

Figure S74 2D NMR COSY ($CDCl_3$) spectrum of **21.**

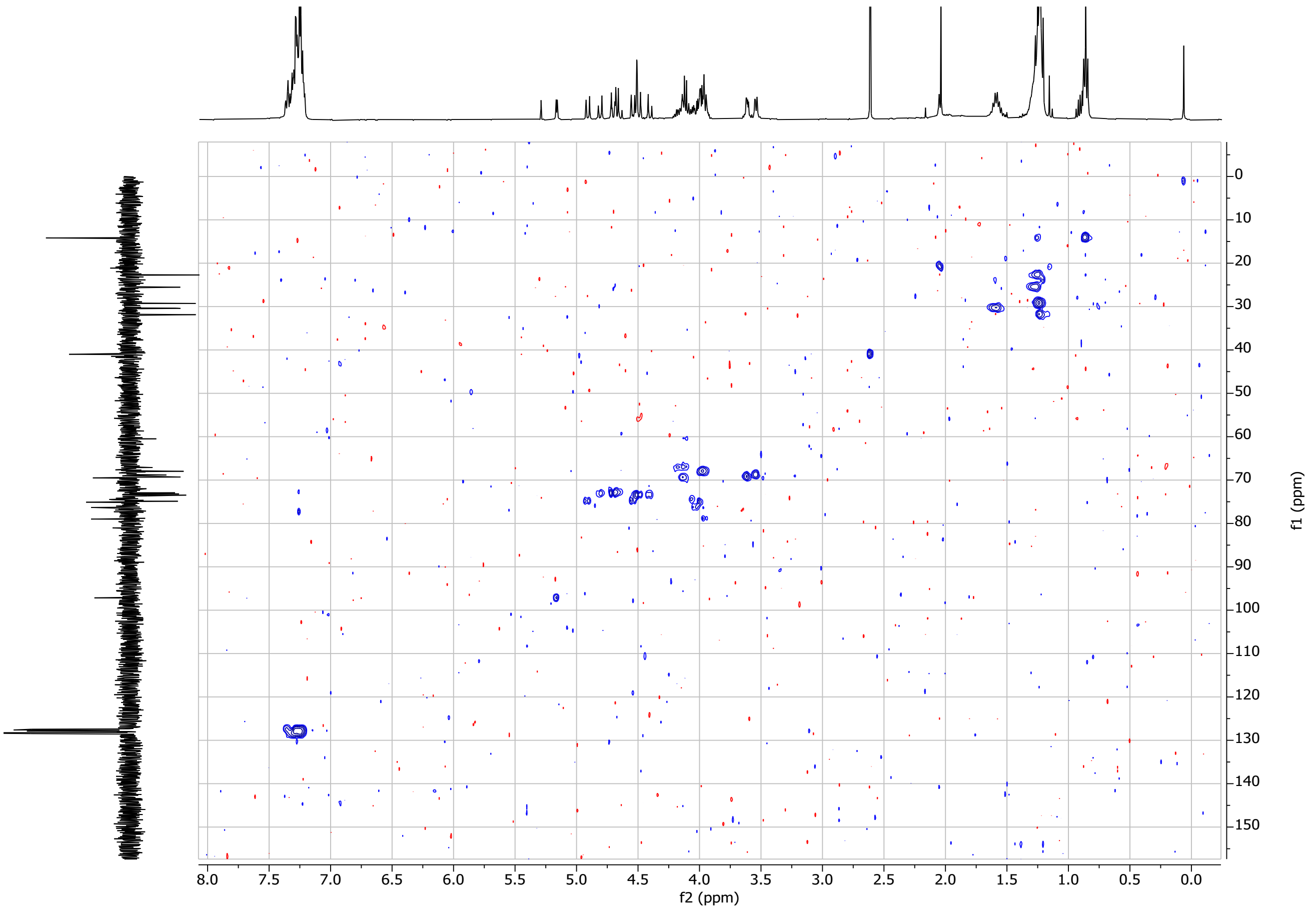

Figure S75 2D NMR HSQC ($CDCl_3$) spectrum of **21.**

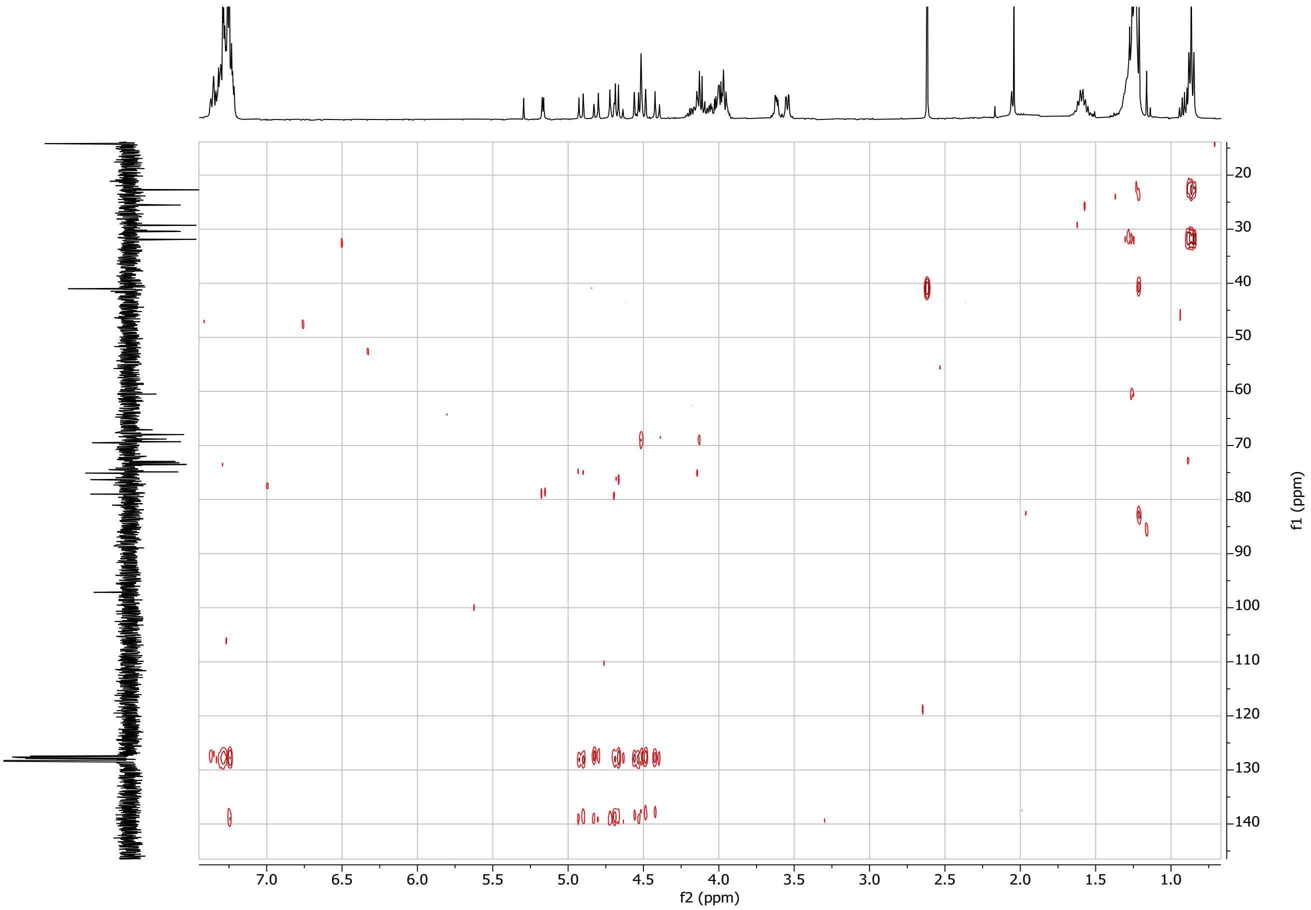

Figure S76 2D NMR HMBC ($CDCl_3$) spectrum of **21.**

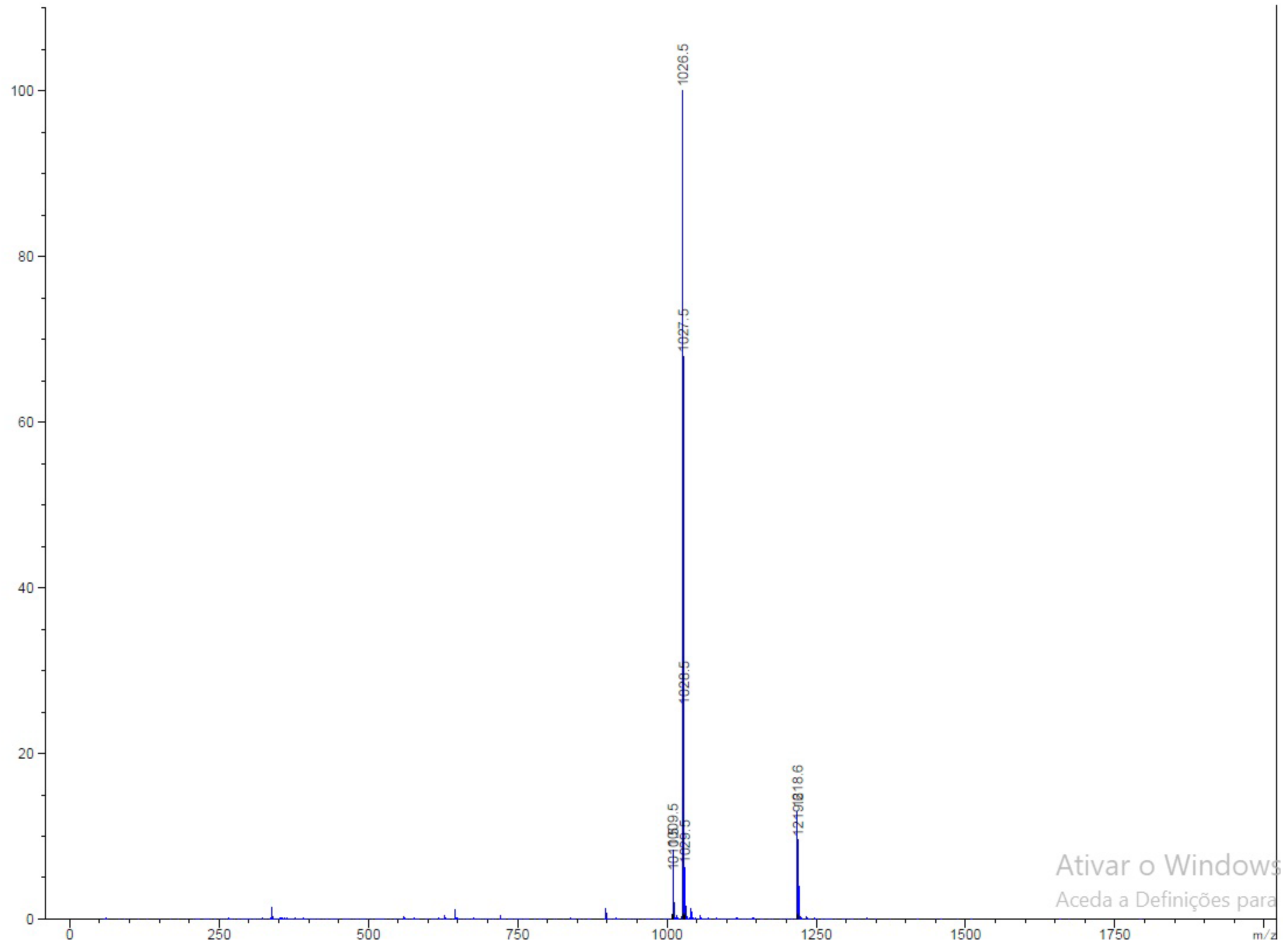

Figure S77 LCMS spectrum of **21.**

Compound 22

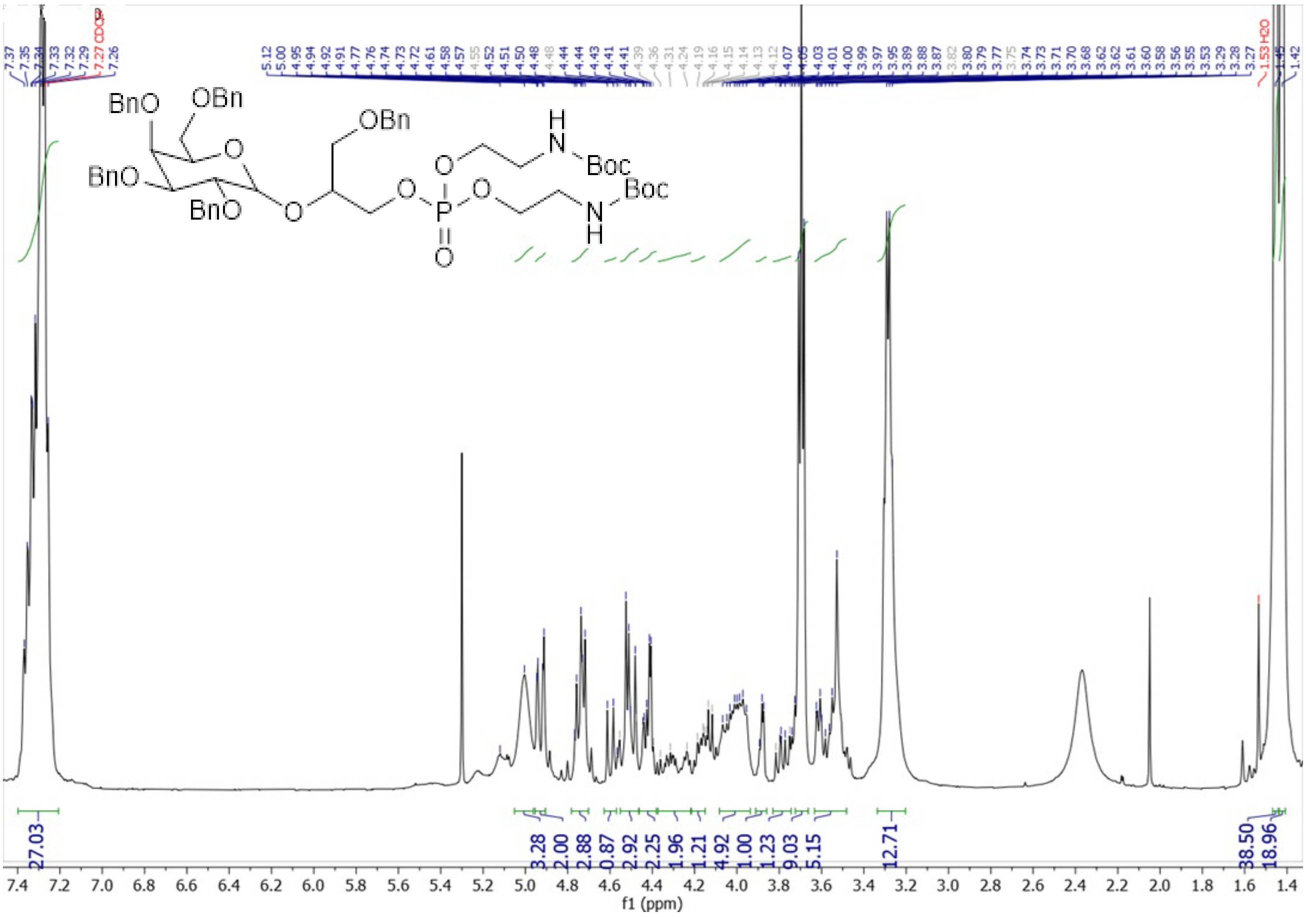

Figure S78 ^{1}H NMR ($CDCl_3$) spectrum of **22** (*β*-anomer).

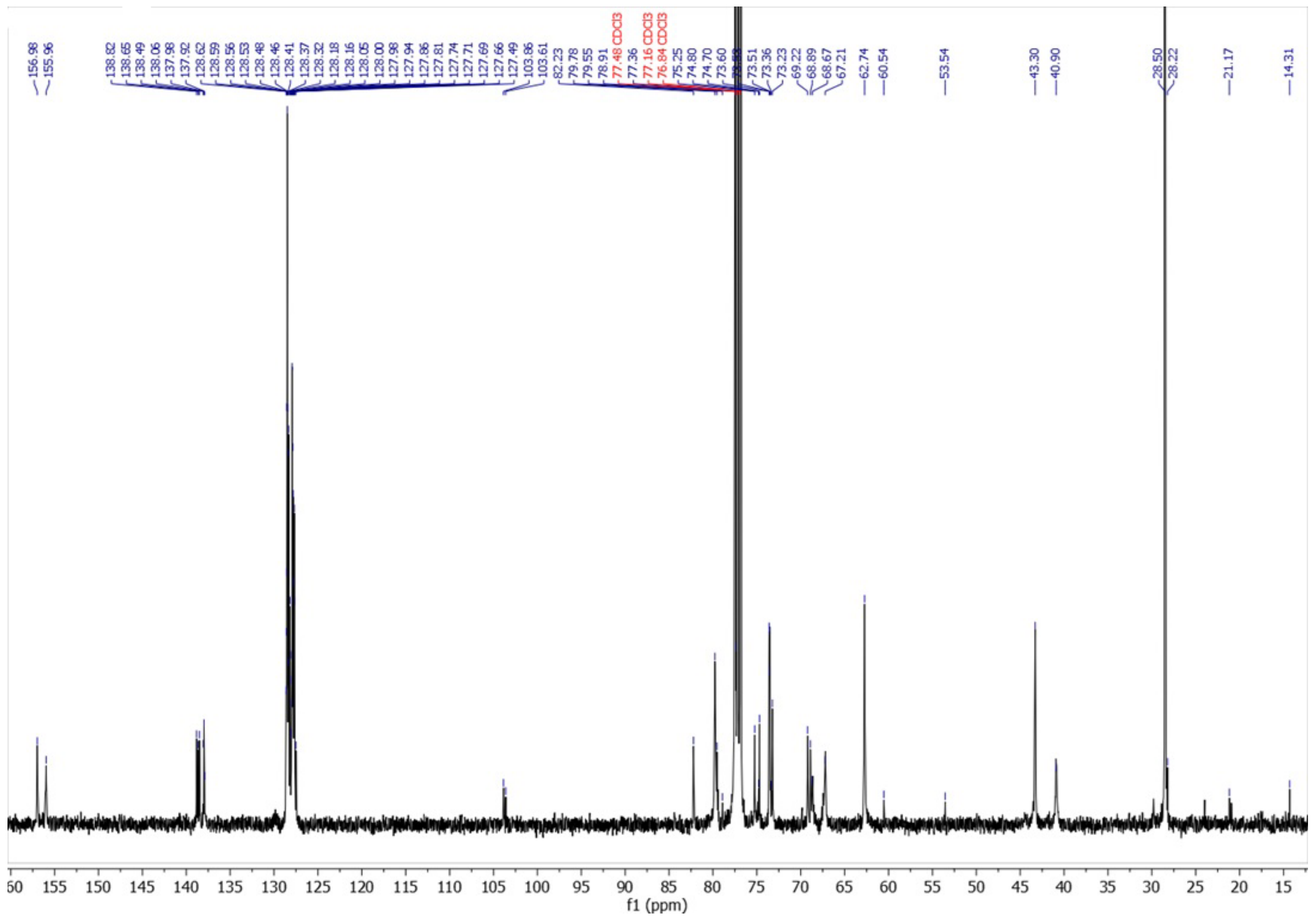

Figure S79 ^{13}C NMR ($CDCl_3$) spectrum of **22** (*β*-anomer).

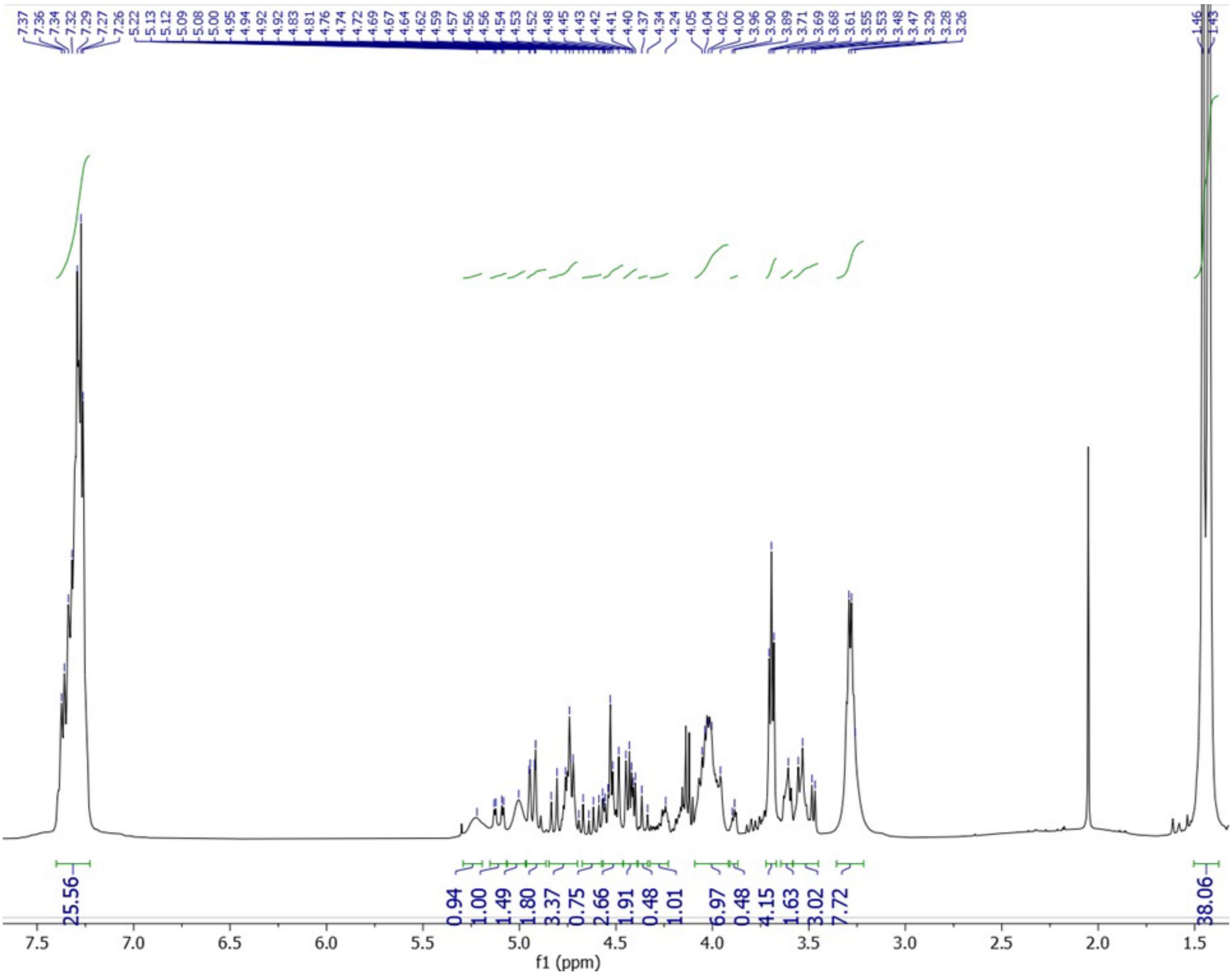

Figure S80 ^{1}H NMR ($CDCl_3$) spectrum of **22** (mixture of *α*- and *β*-anomers).

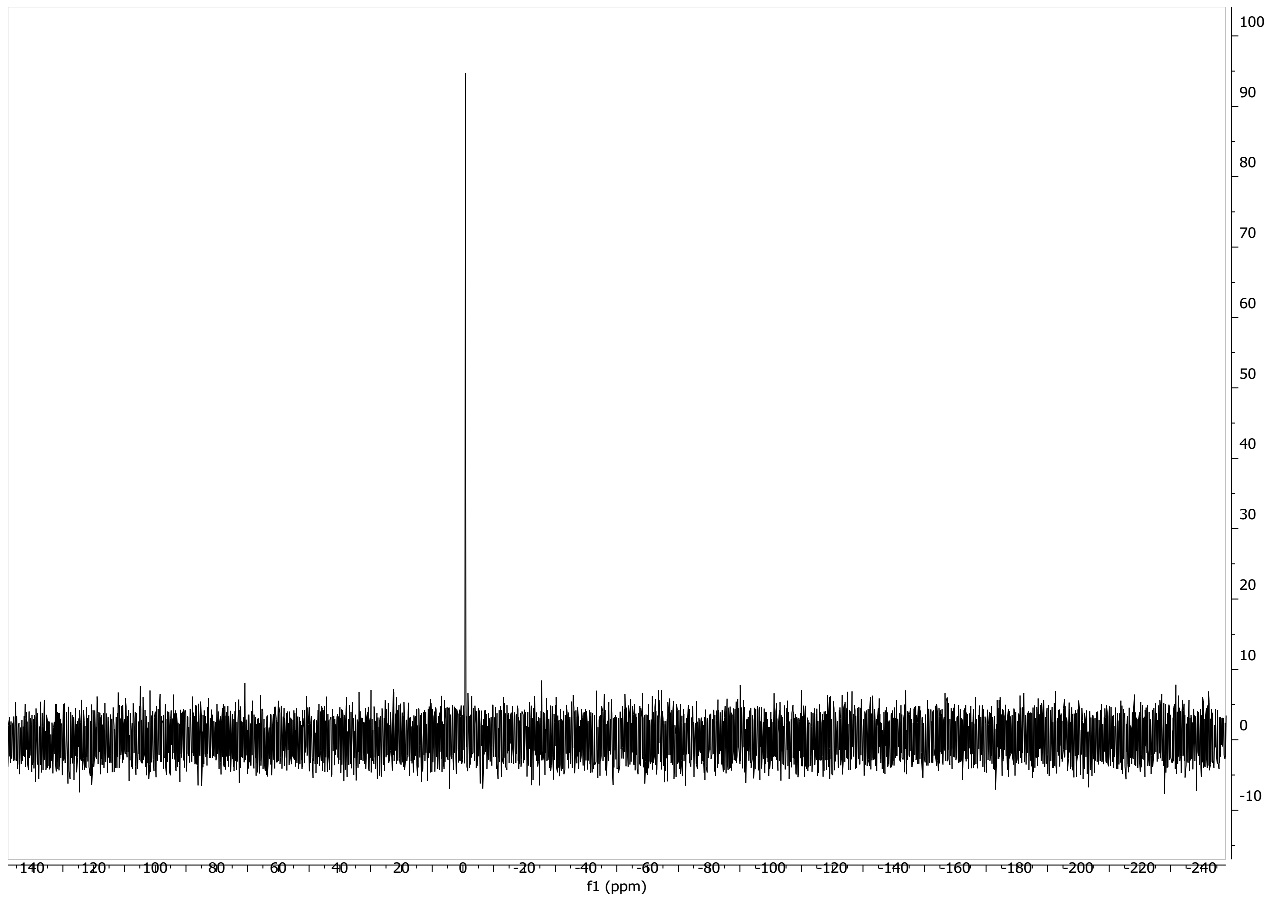

Figure S81 ^{31}P NMR ($CDCl_3$) spectrum of **22** (mixture of *α*- and *β*-anomers).

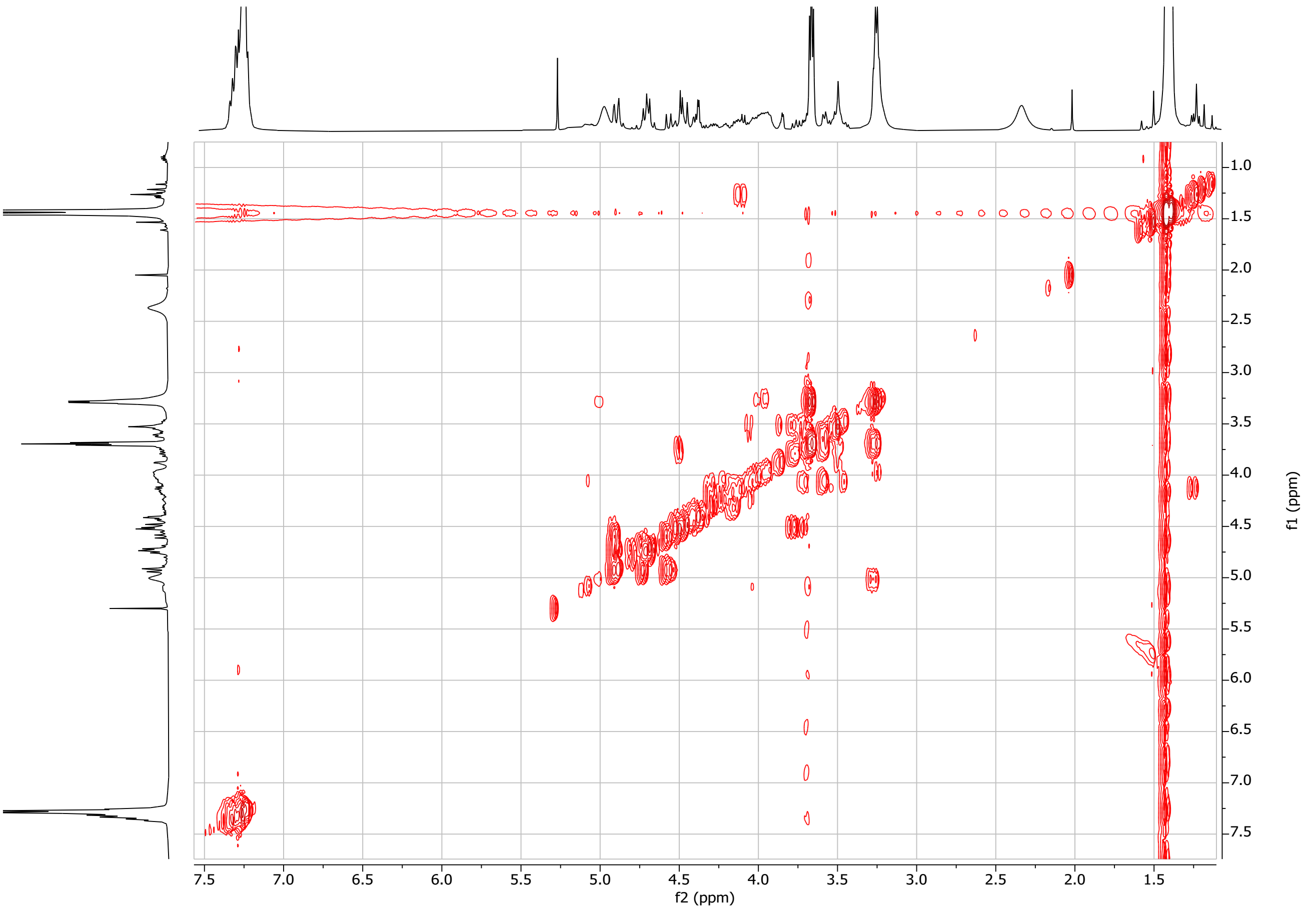

Figure S82 2D NMR COSY ($CDCl_3$) spectrum of **22** (β-anomer).

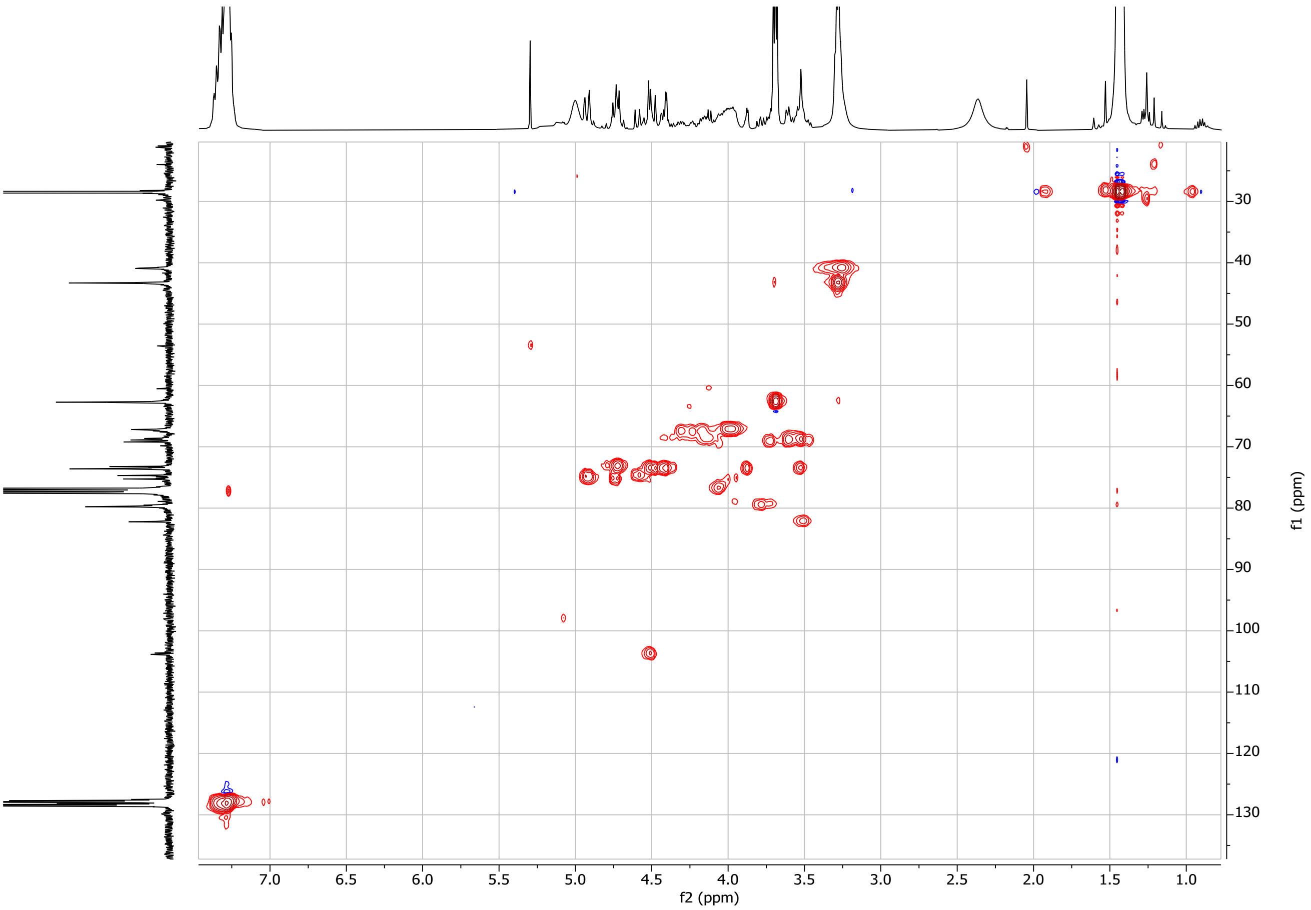

Figure S83 2D NMR HSQC ($CDCl_3$) spectrum of **22** (*β*-anomer).

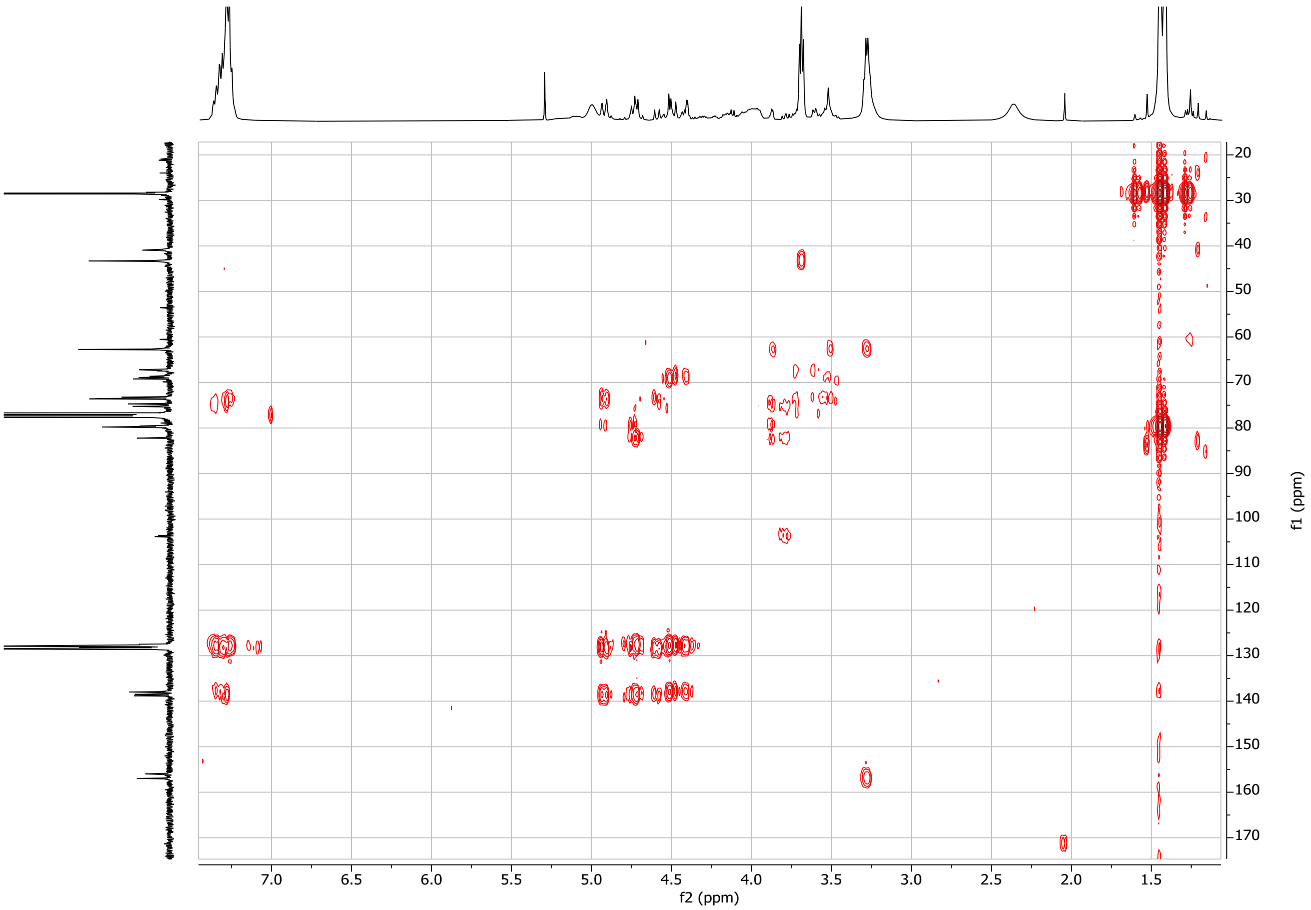

Figure S84 2D NMR HMBC ($CDCl_3$) spectrum of **22** (*β*-anomer).

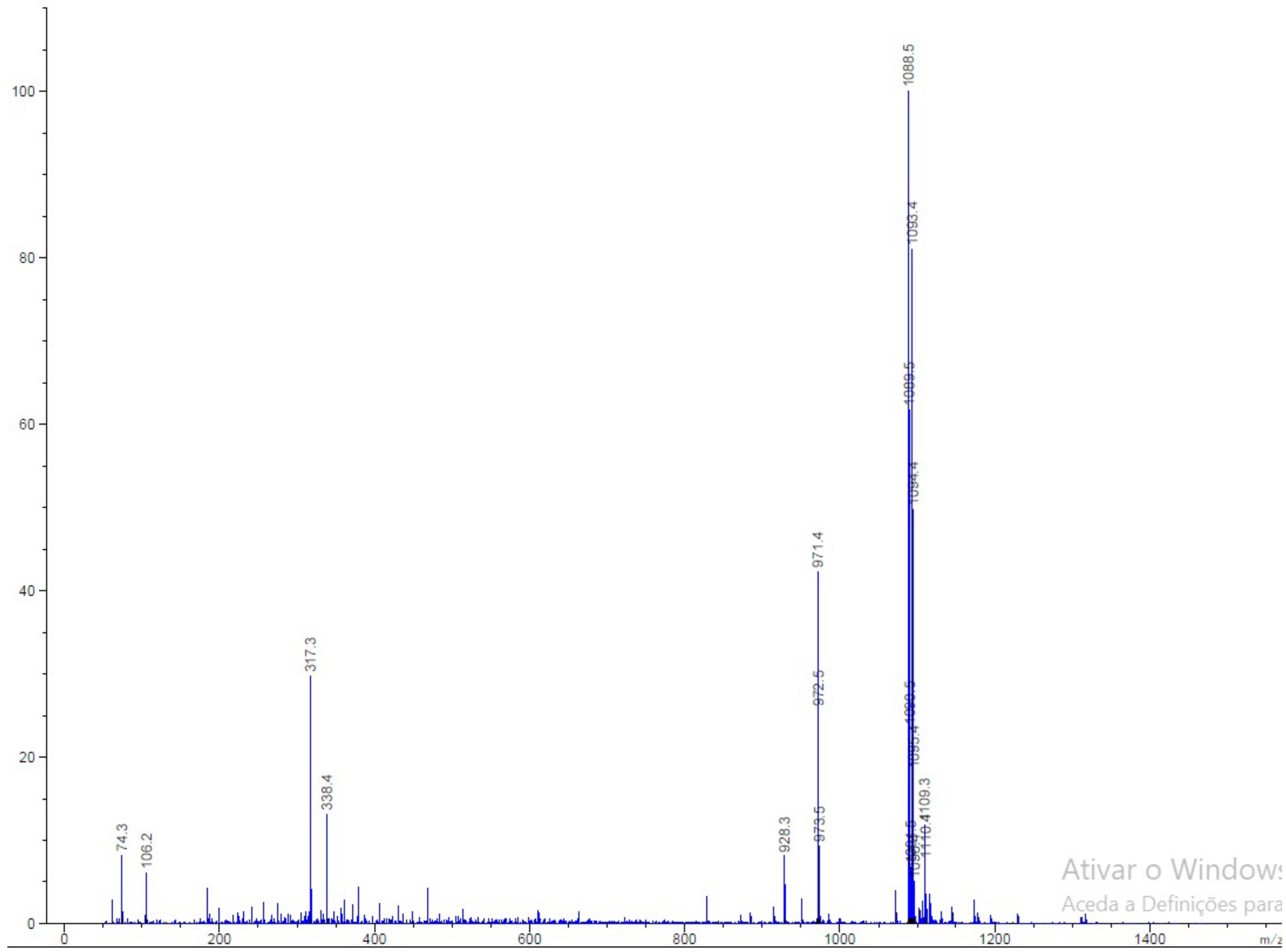

Figure S85 LCMS spectrum of **22.**

Compound 23

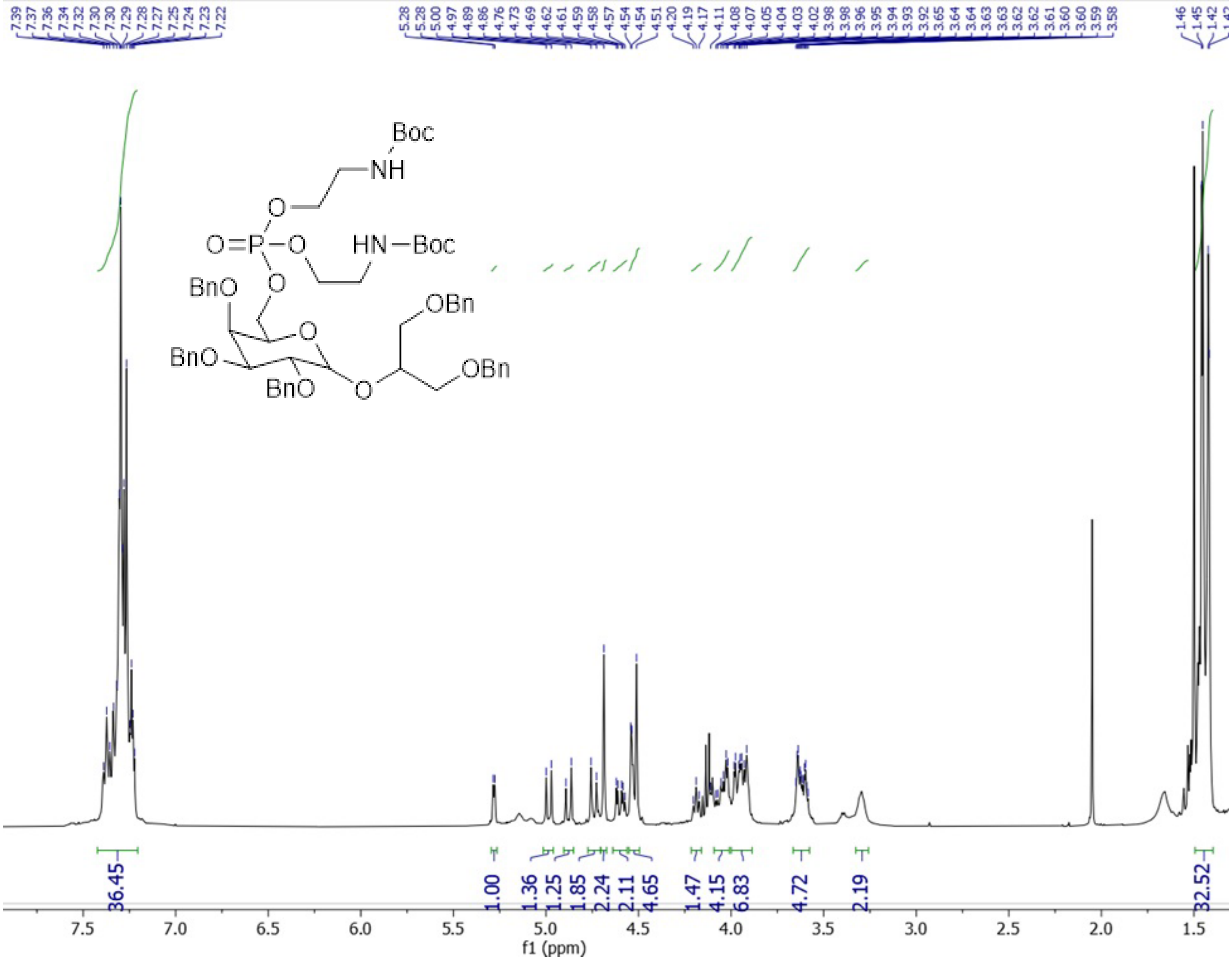

Figure S86 ^{1}H NMR ($CDCl_3$) spectrum of **23.**

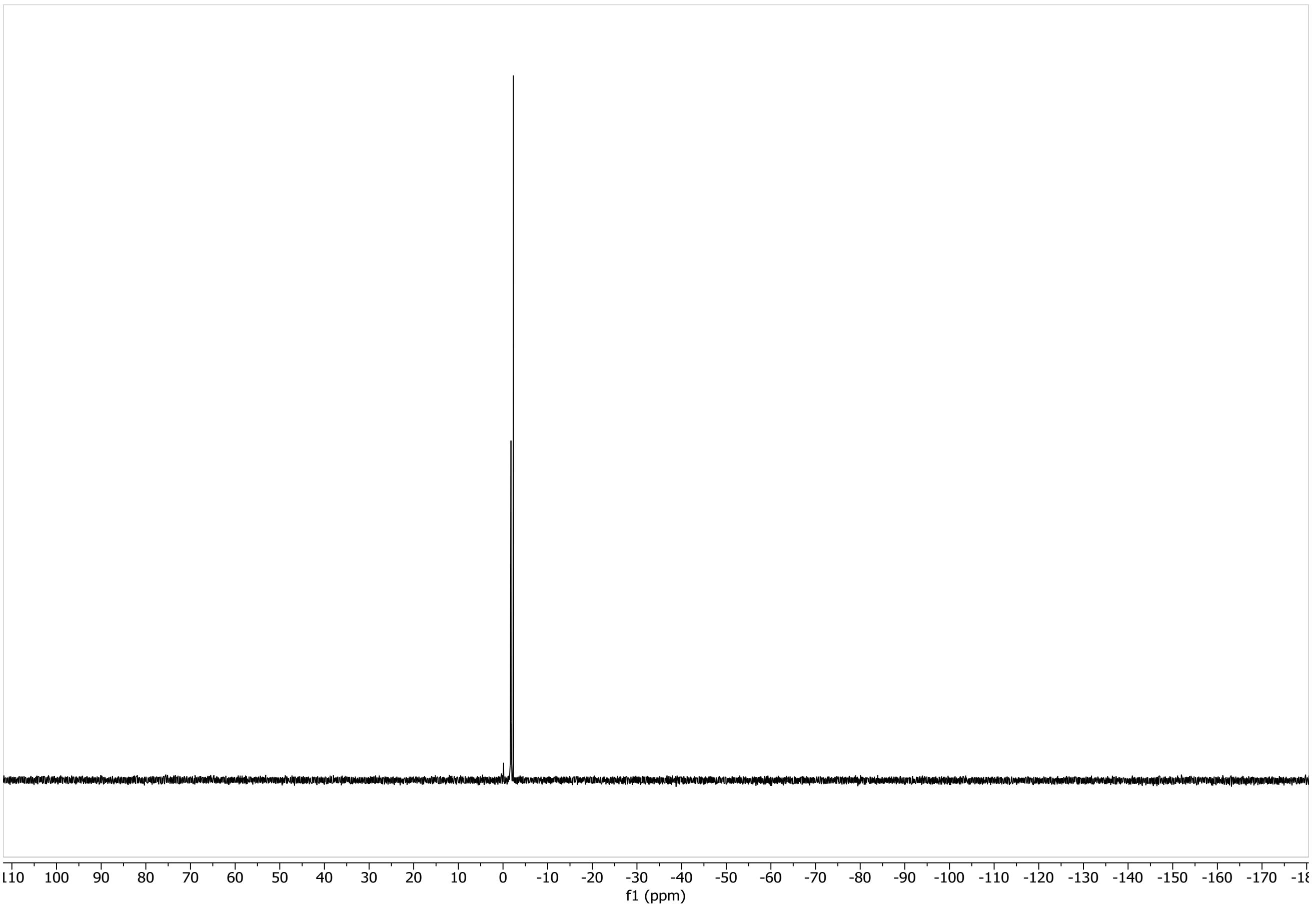

Figure S87 ^{31}P NMR ($CDCl_3$) spectrum of **23.**

Compound 24

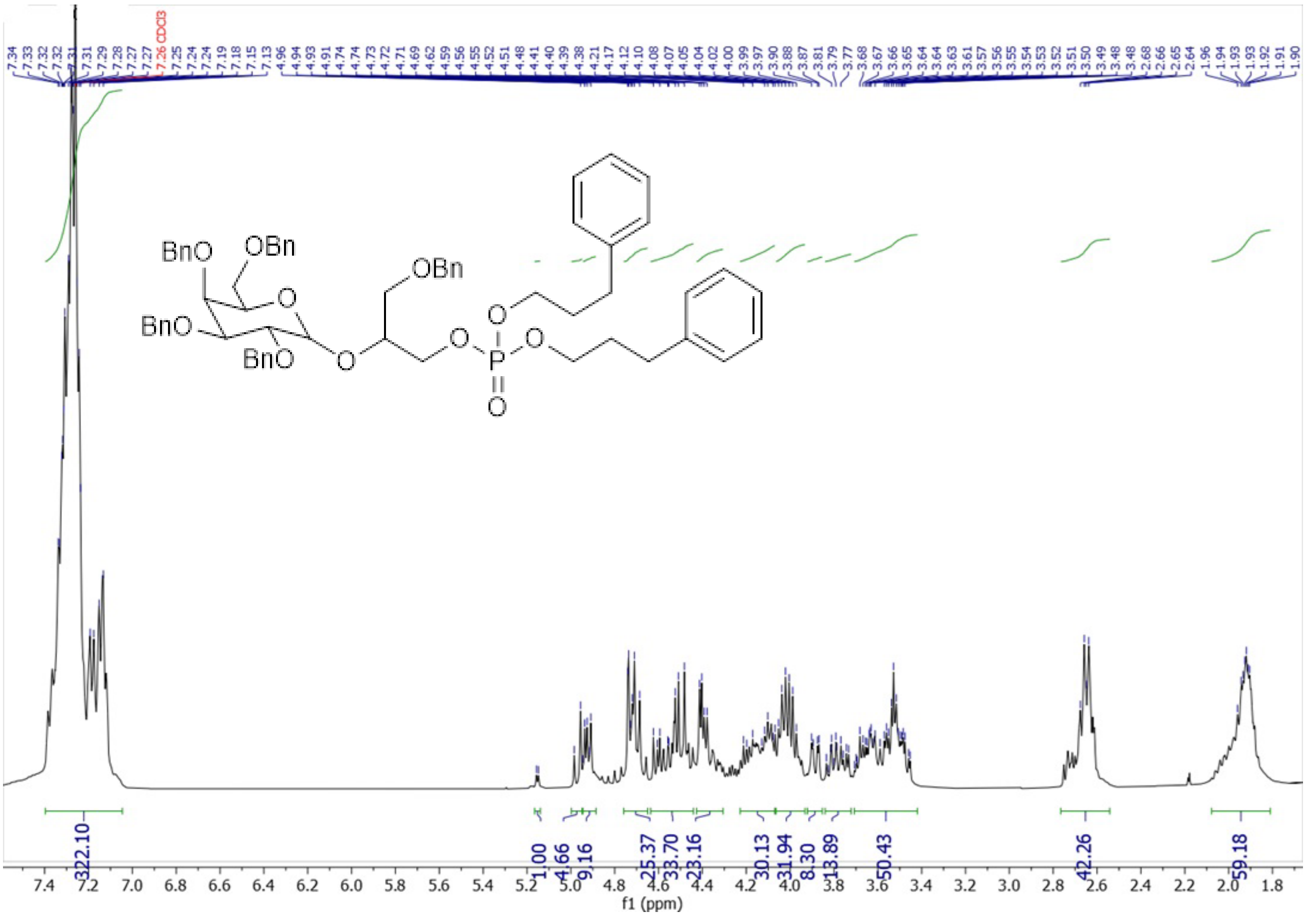

Figure S88 ^{1}H NMR ($CDCl_3$) spectrum of **24.**

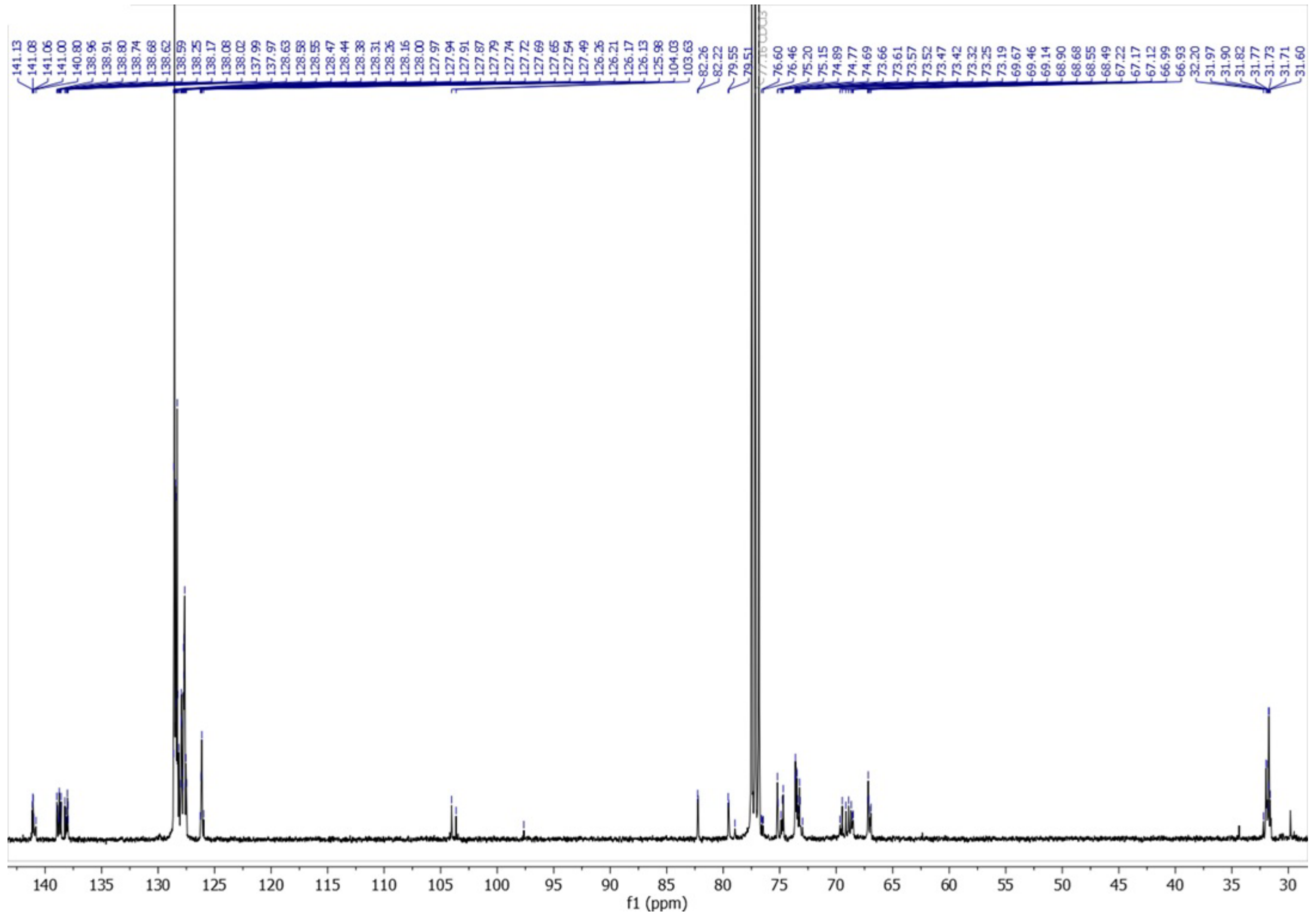

Figure S89 ^{13}C NMR ($CDCl_3$) spectrum of **24.**

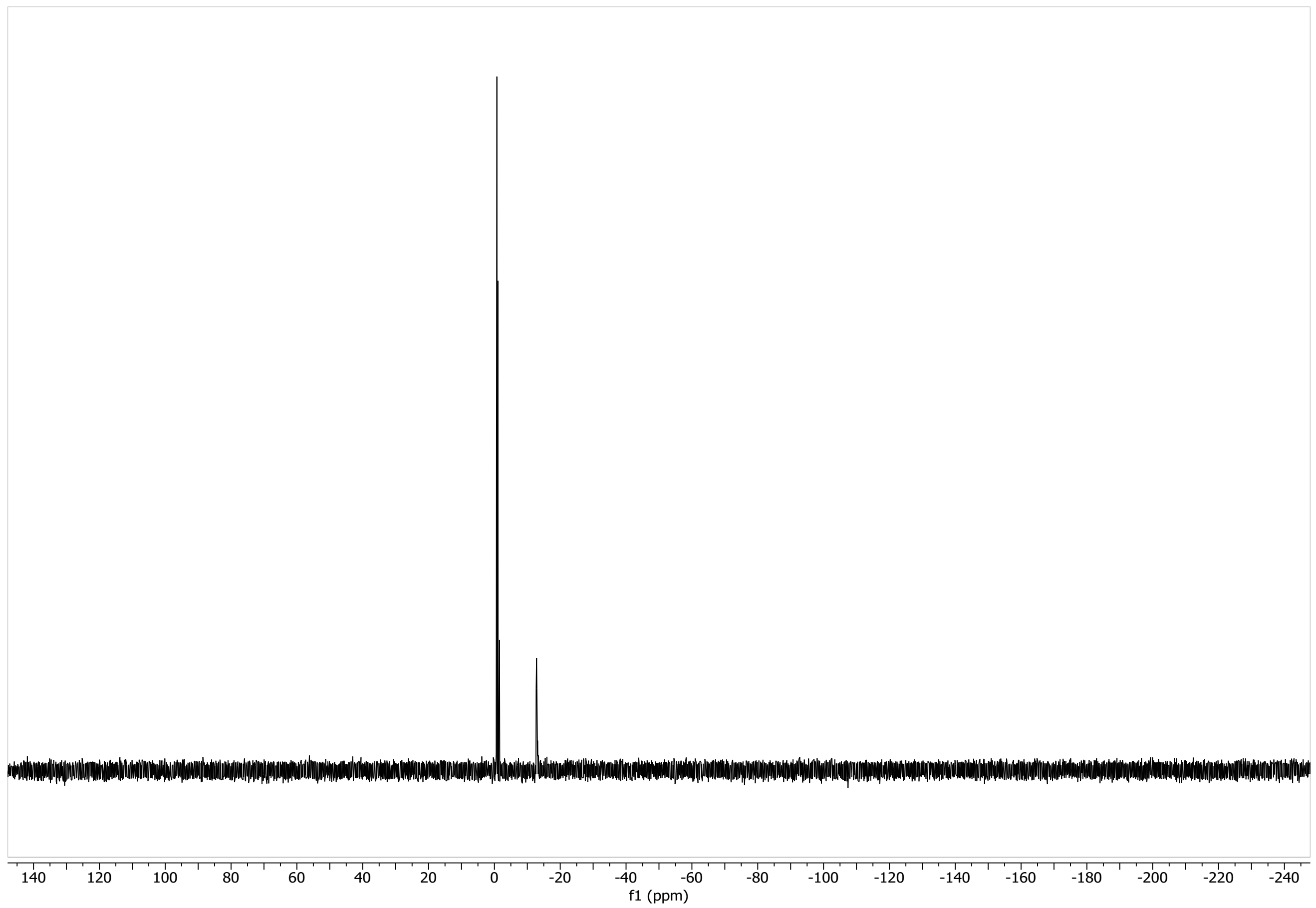

Figure S90 ^{31}P NMR ($CDCl_3$) spectrum of **24.**

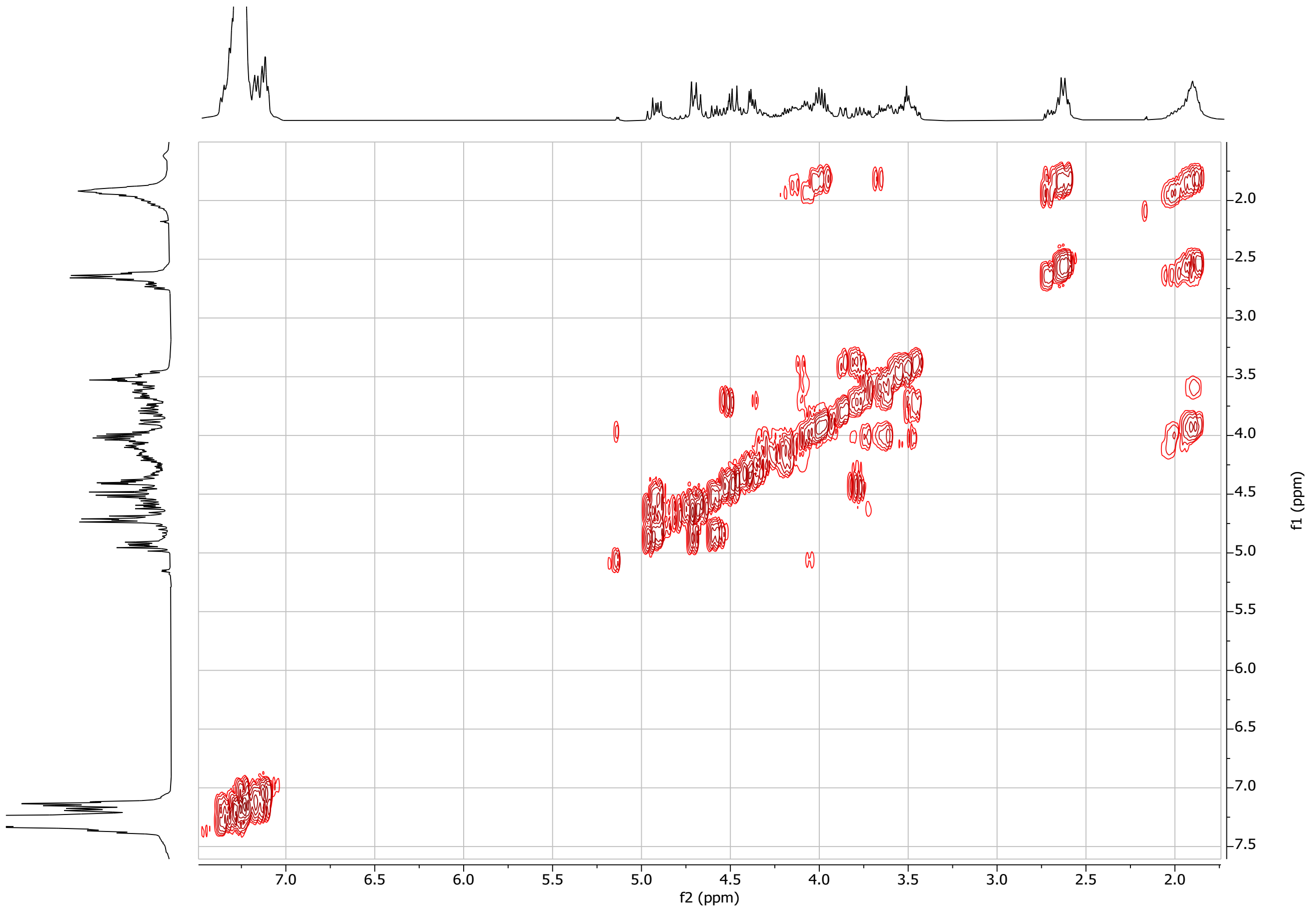

Figure S91 2D COSY NMR ($CDCl_3$) spectrum of **24.**

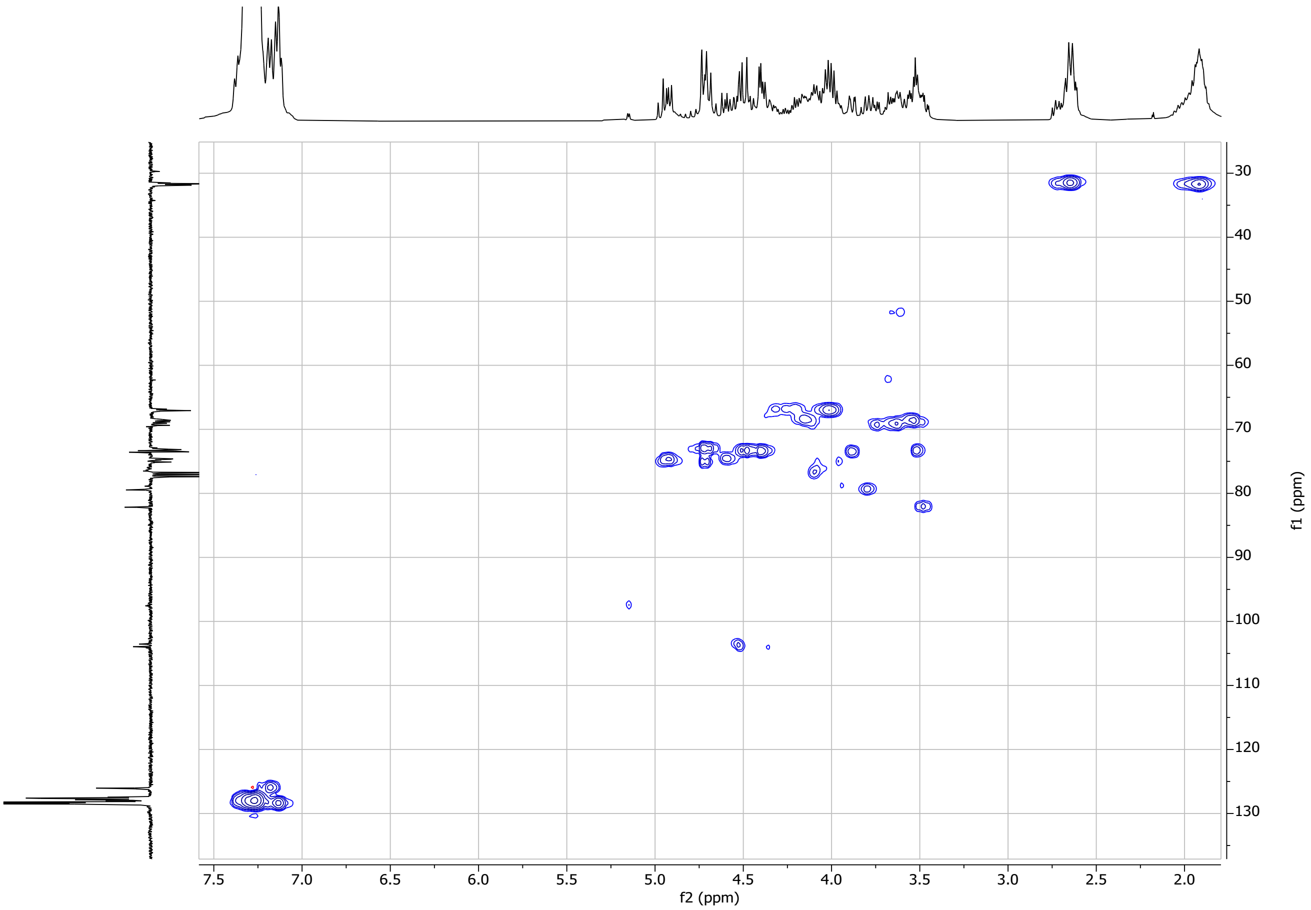

Figure S92 2D HSQC NMR ($CDCl_3$) spectrum of **24.**

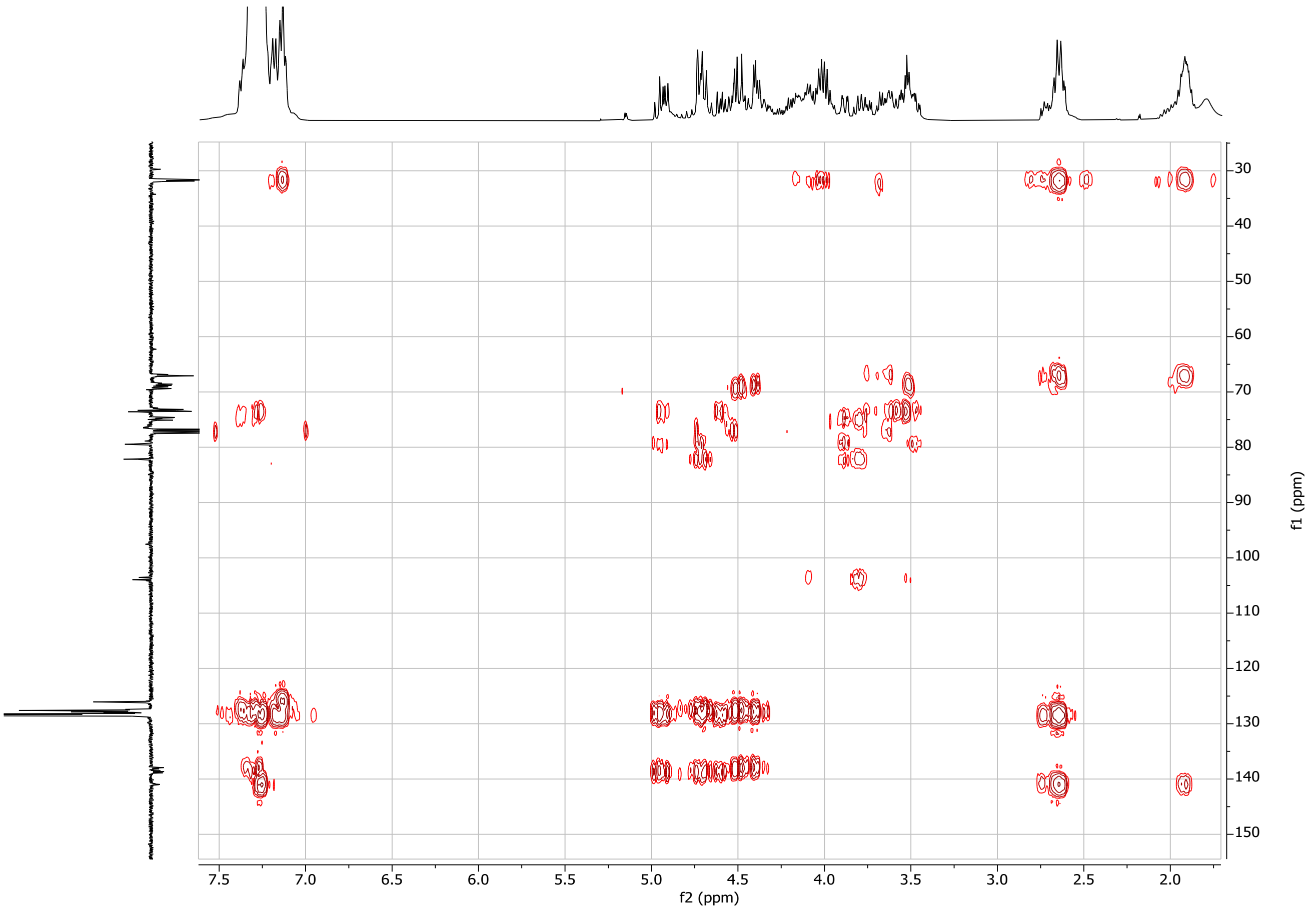

Figure S93 2D HMBC NMR (CDCl₃) spectrum of **24.**

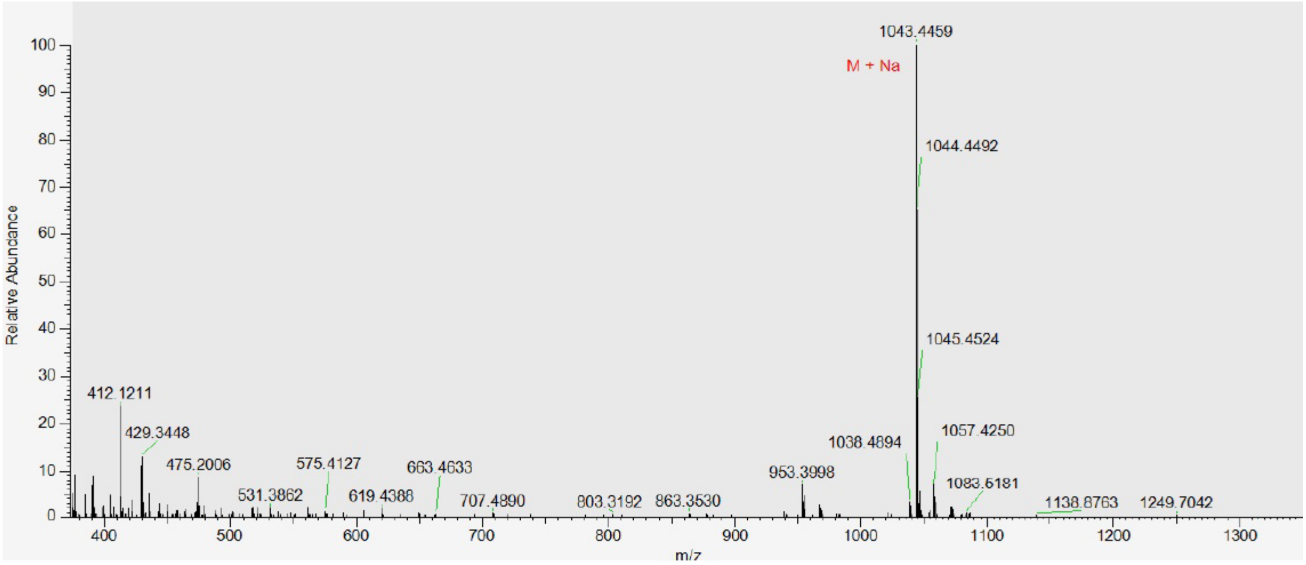

Figure S94 ESI-HRMS spectrum of **24.**

Compound 25

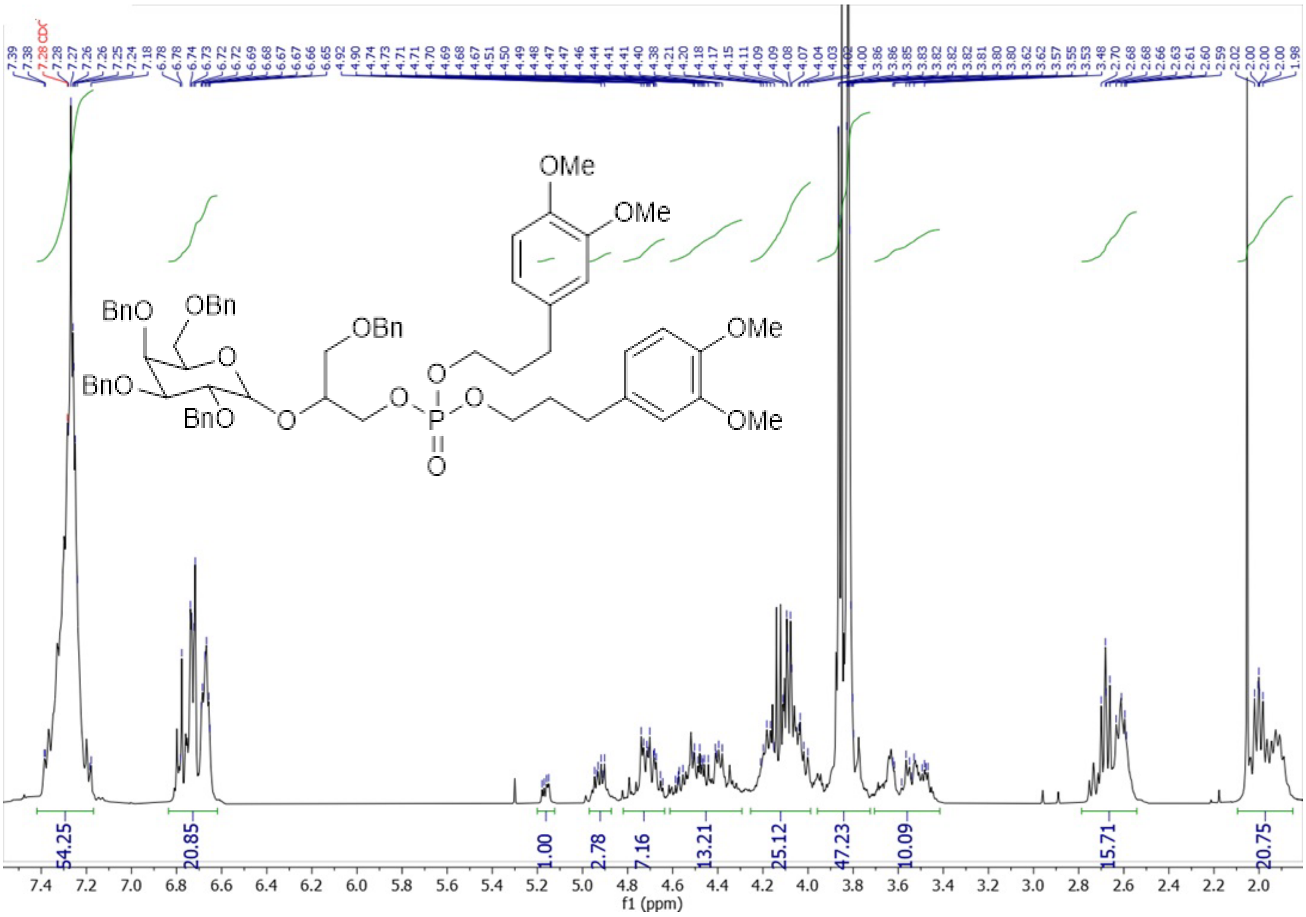

Figure S95 ^{1}H NMR ($CDCl_3$) spectrum of **25**.

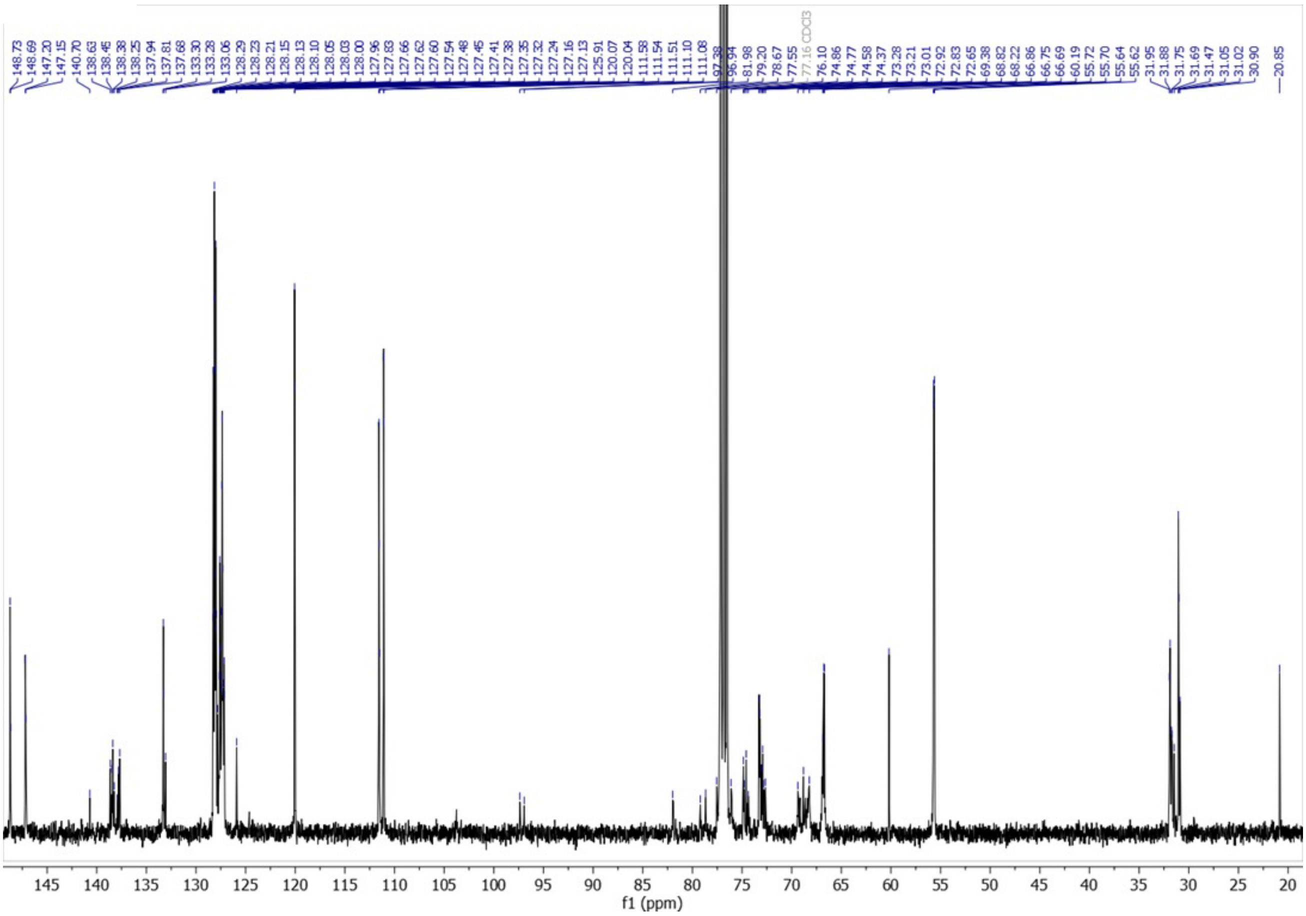

Figure S96 ^{13}C NMR ($CDCl_3$) spectrum of **25.**

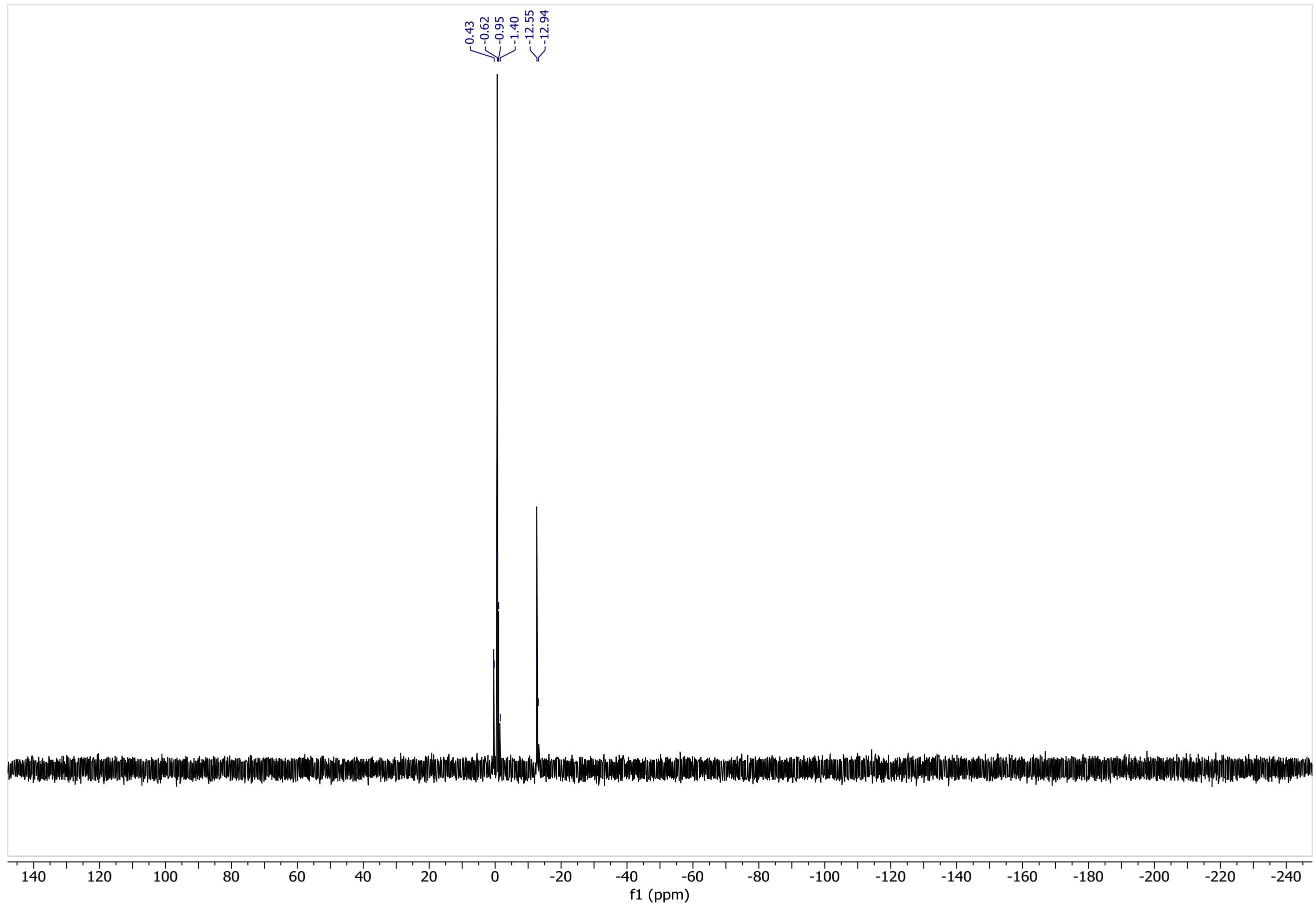

Figure S97 ^{31}P NMR ($CDCl_3$) spectrum of **25.**

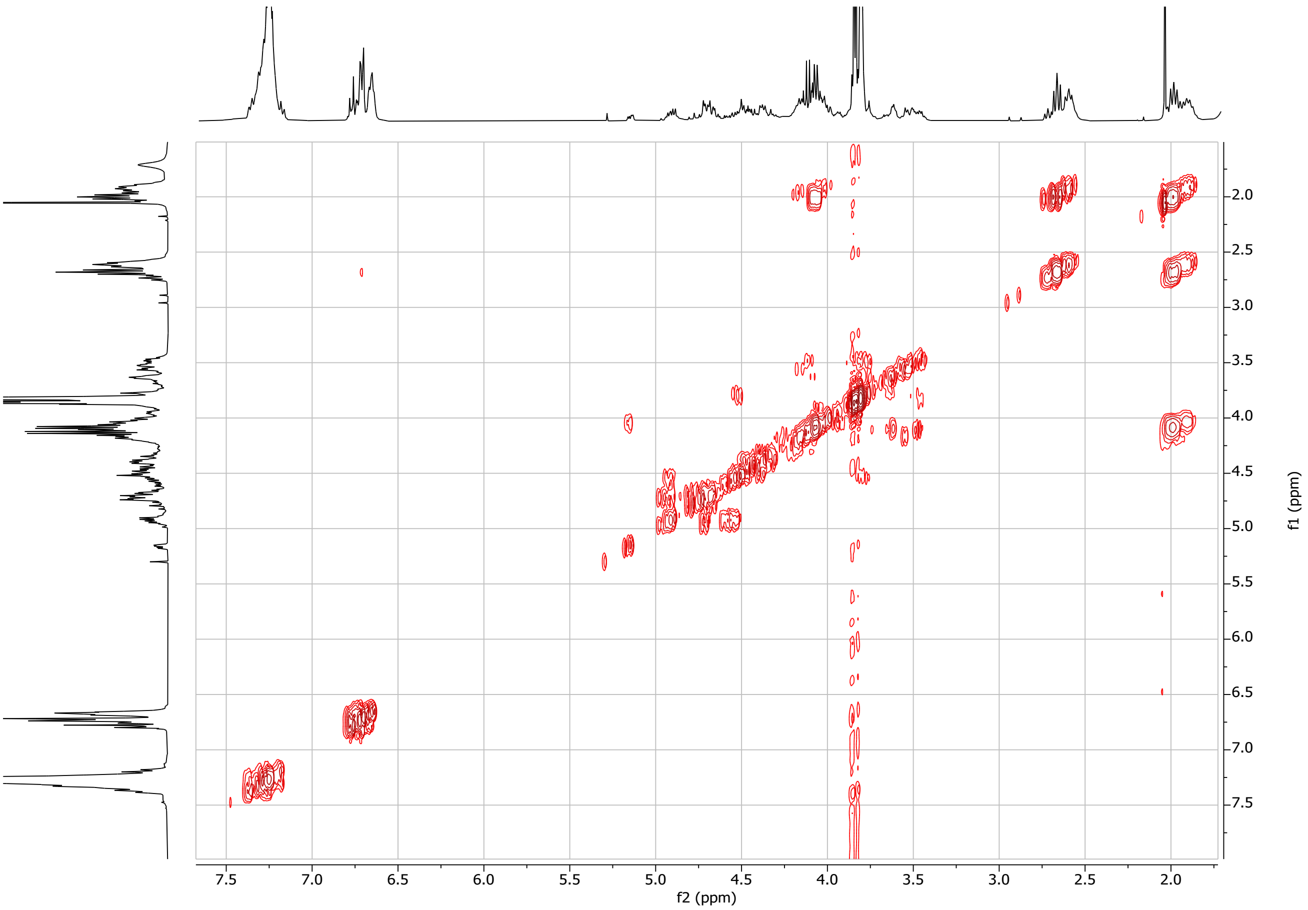

Figure S98 2D NMR COSY ($CDCl_3$) spectrum of **25.**

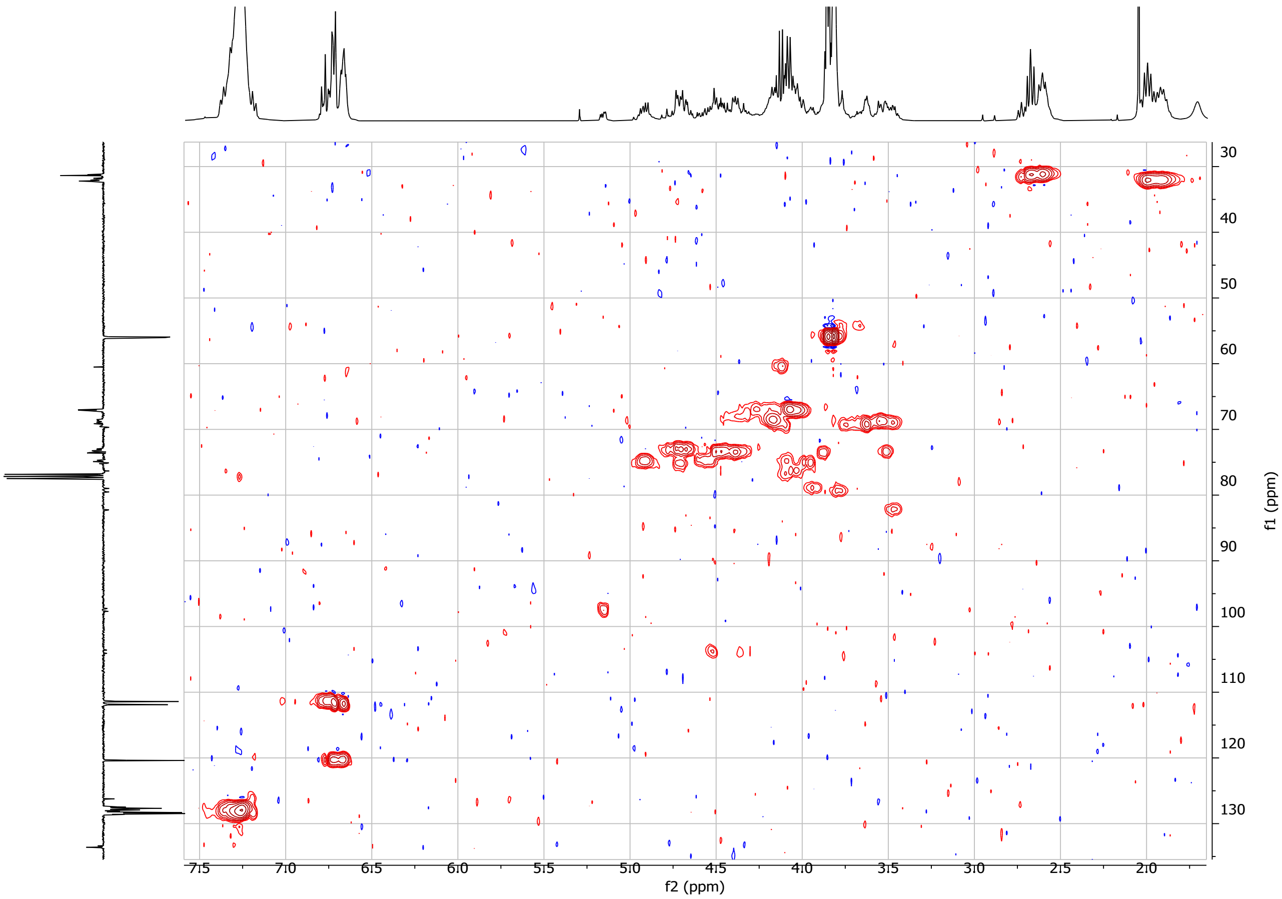

Figure S99 2D NMR HSQC ($CDCl_3$) spectrum of **25.**

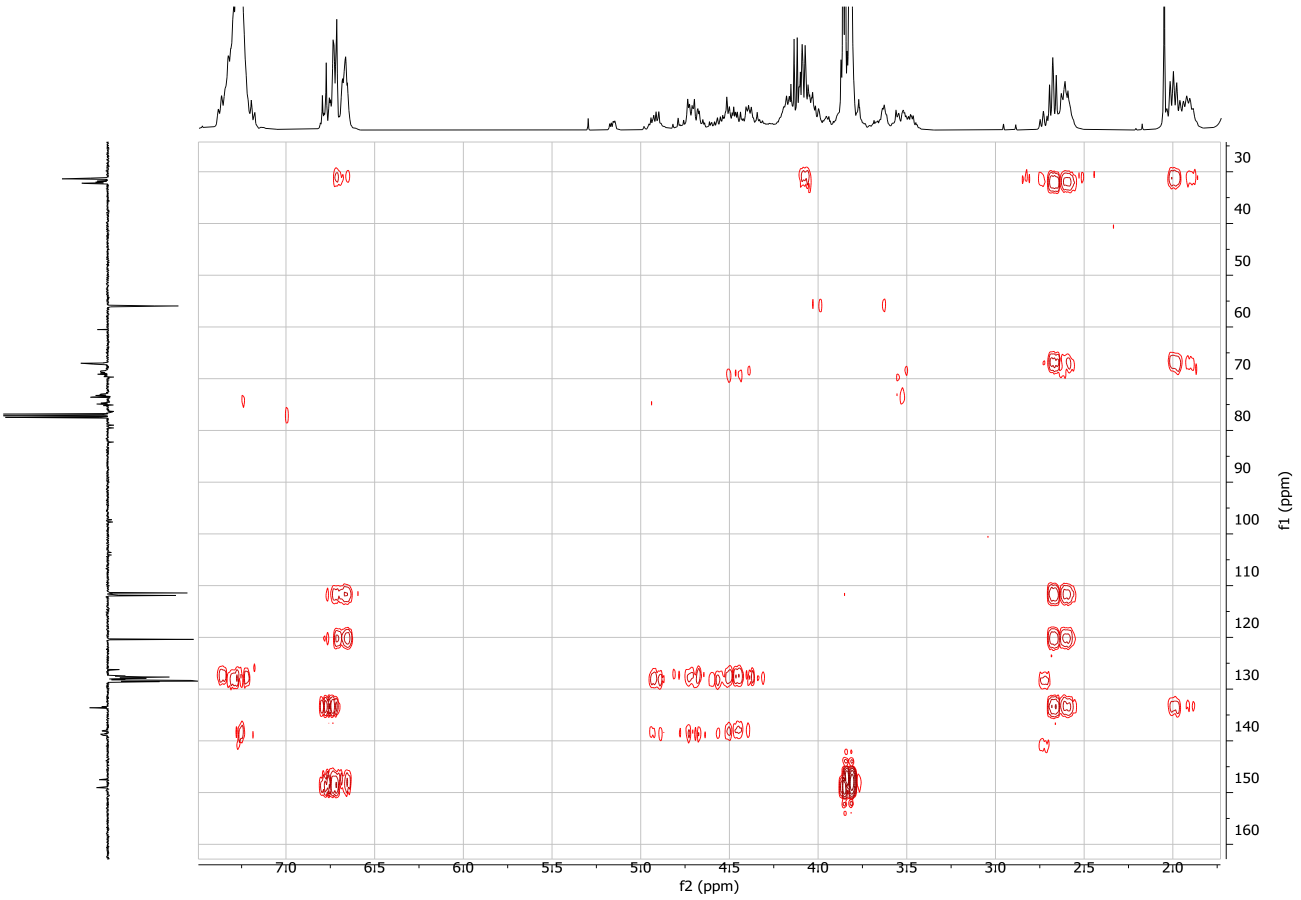

Figure S100 2D NMR HMBC ($CDCl_3$) spectrum of **25.**

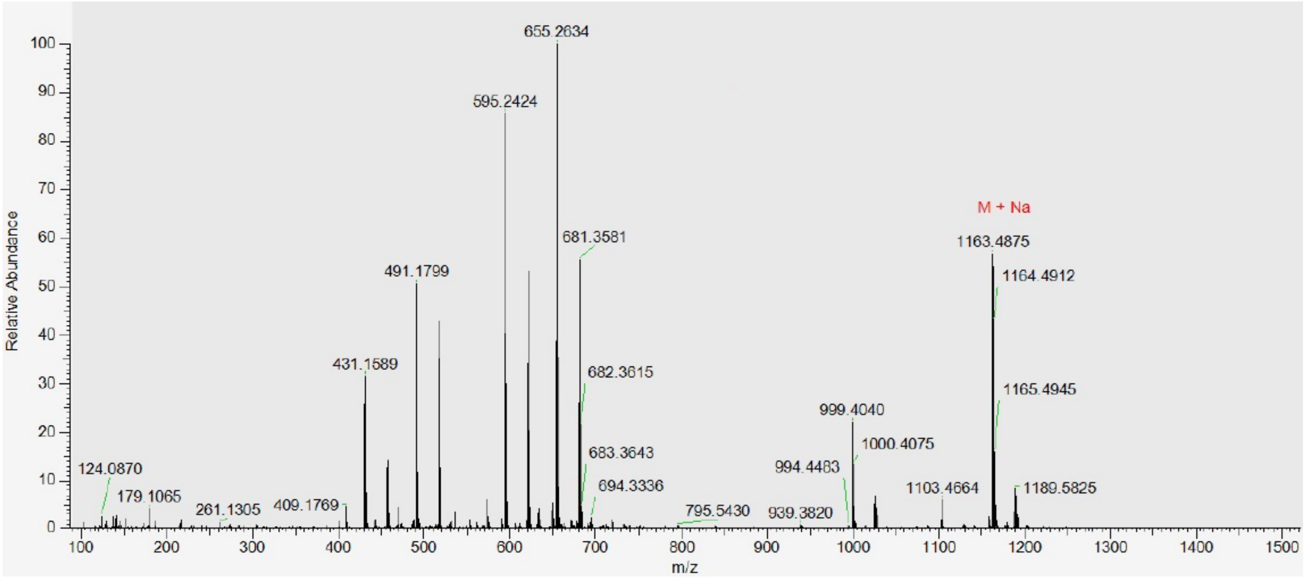

Figure S101 ESI-HRMS spectrum of **25.**

Compound 26

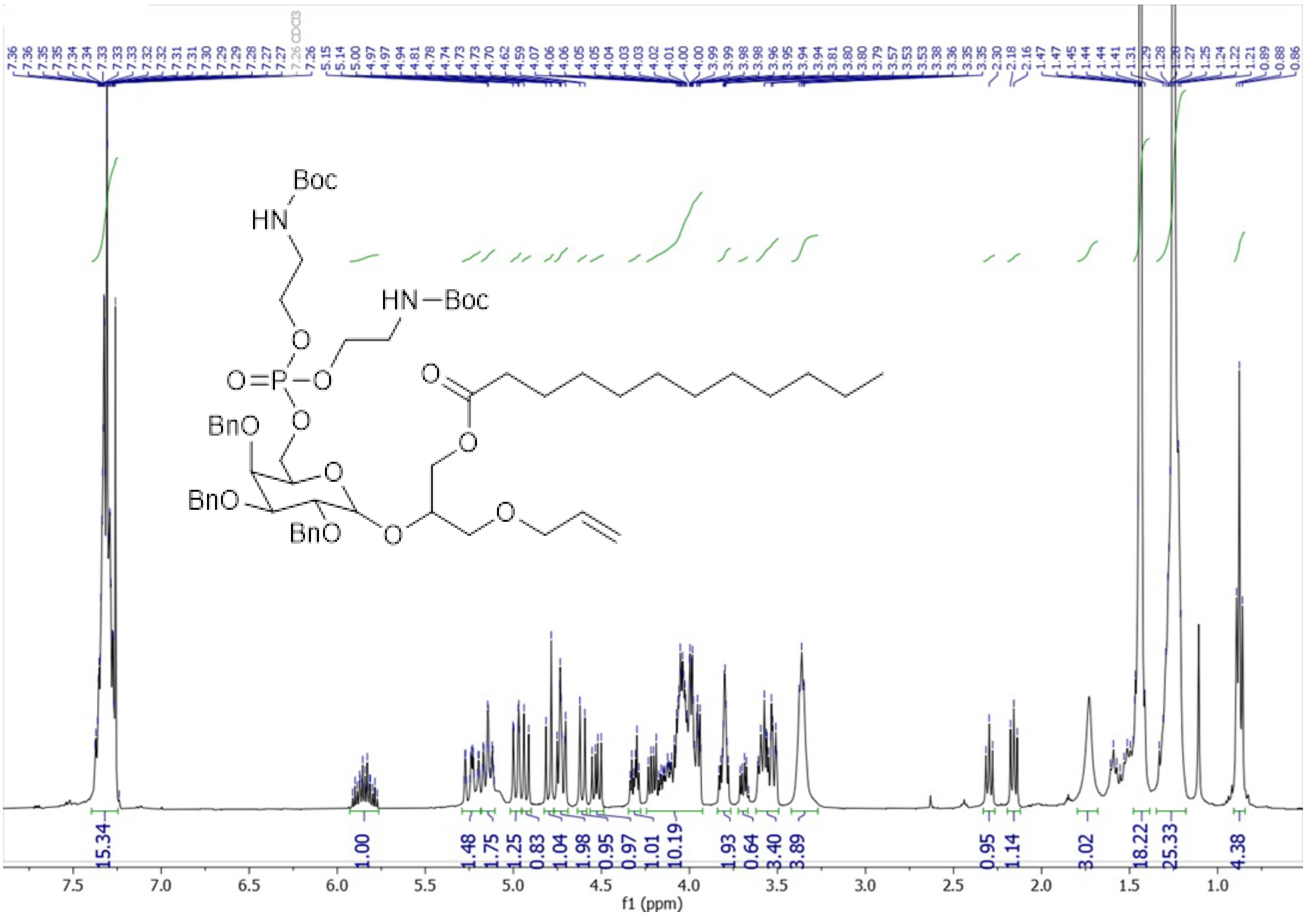

Figure S102 ^{1}H NMR ($CDCl_3$) spectrum of **26.**

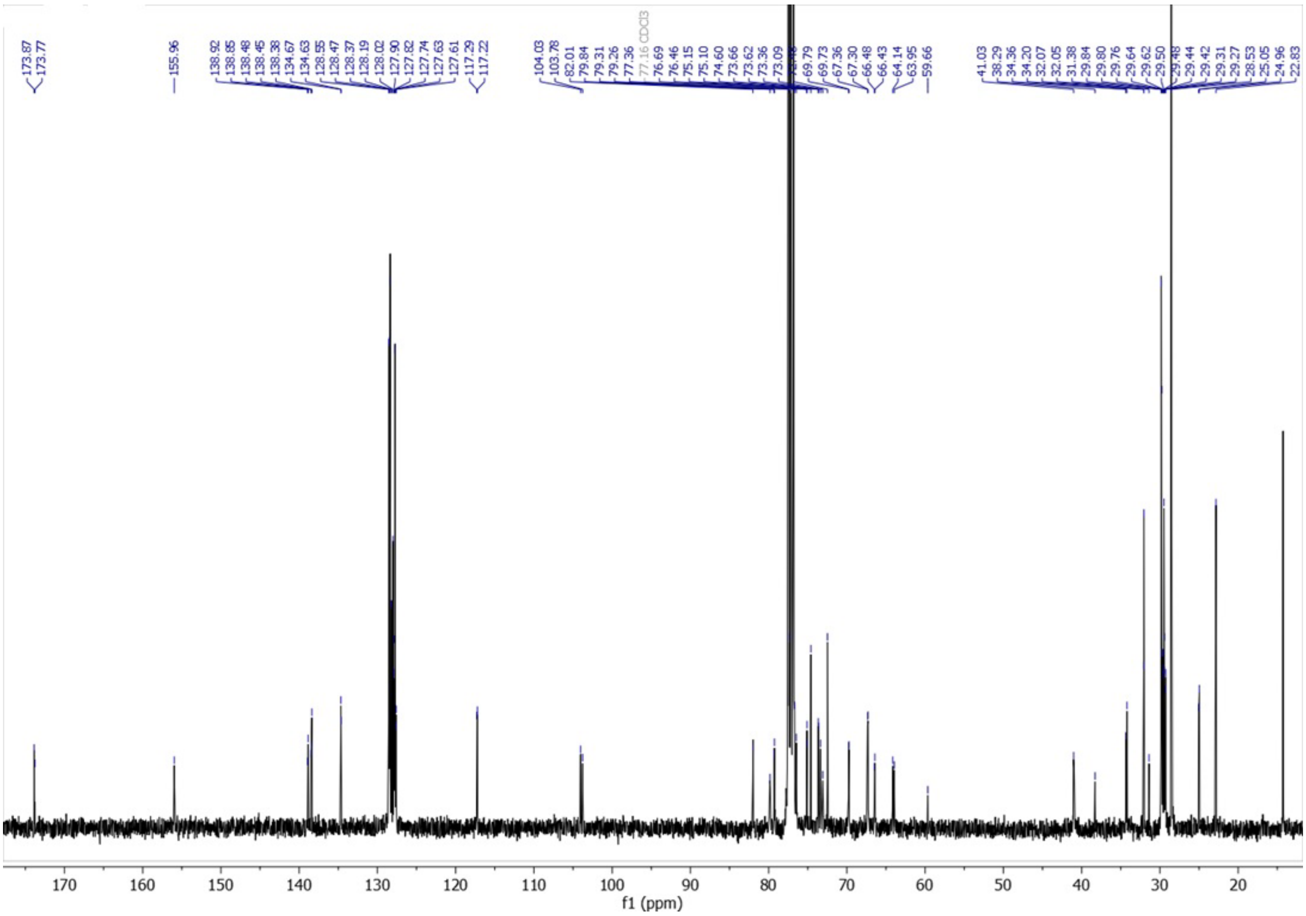

Figure S103 ^{13}C NMR ($CDCl_3$) spectrum of **26.**

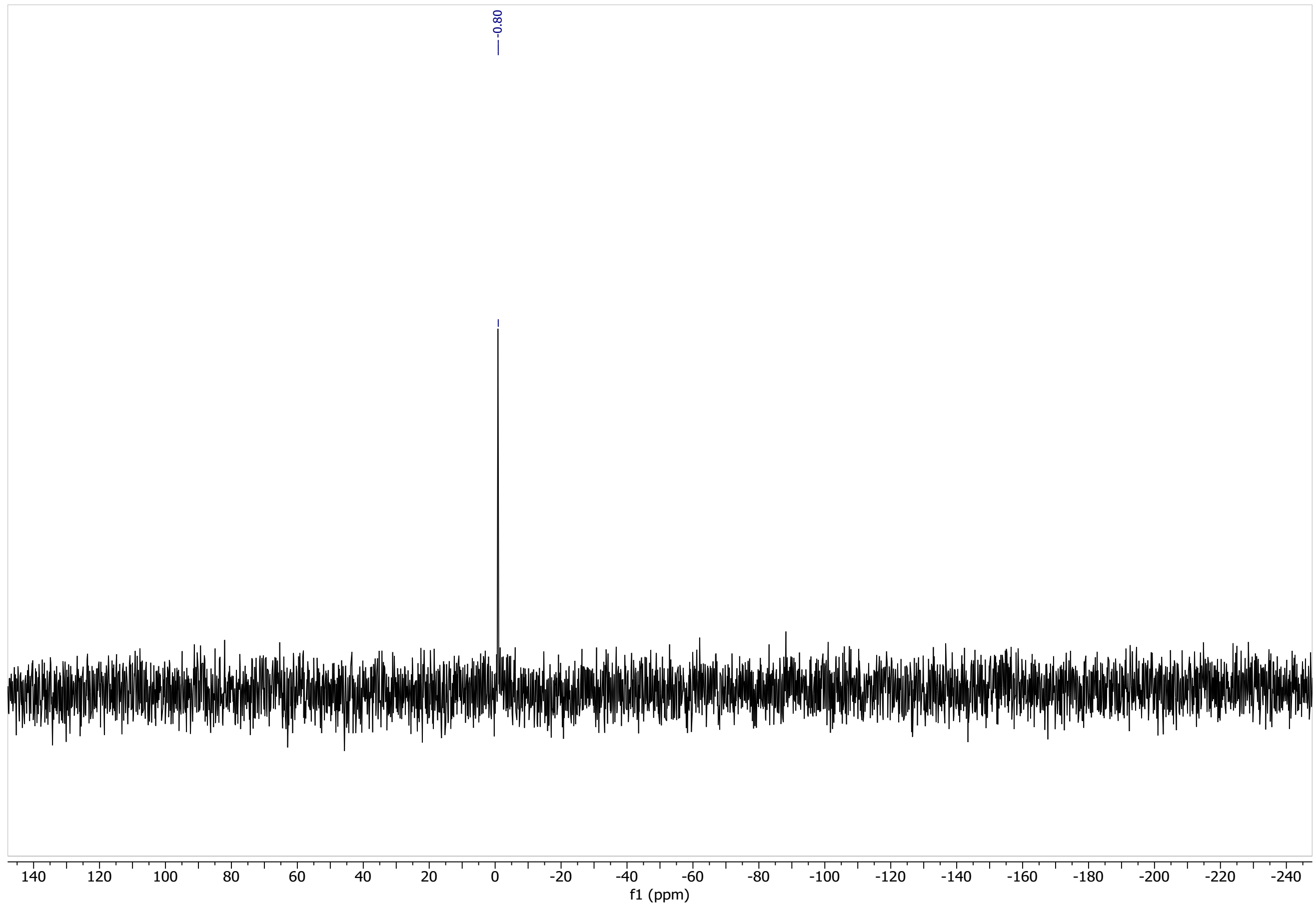

Figure S104 ^{31}P NMR ($CDCl_3$) spectrum of **26.**

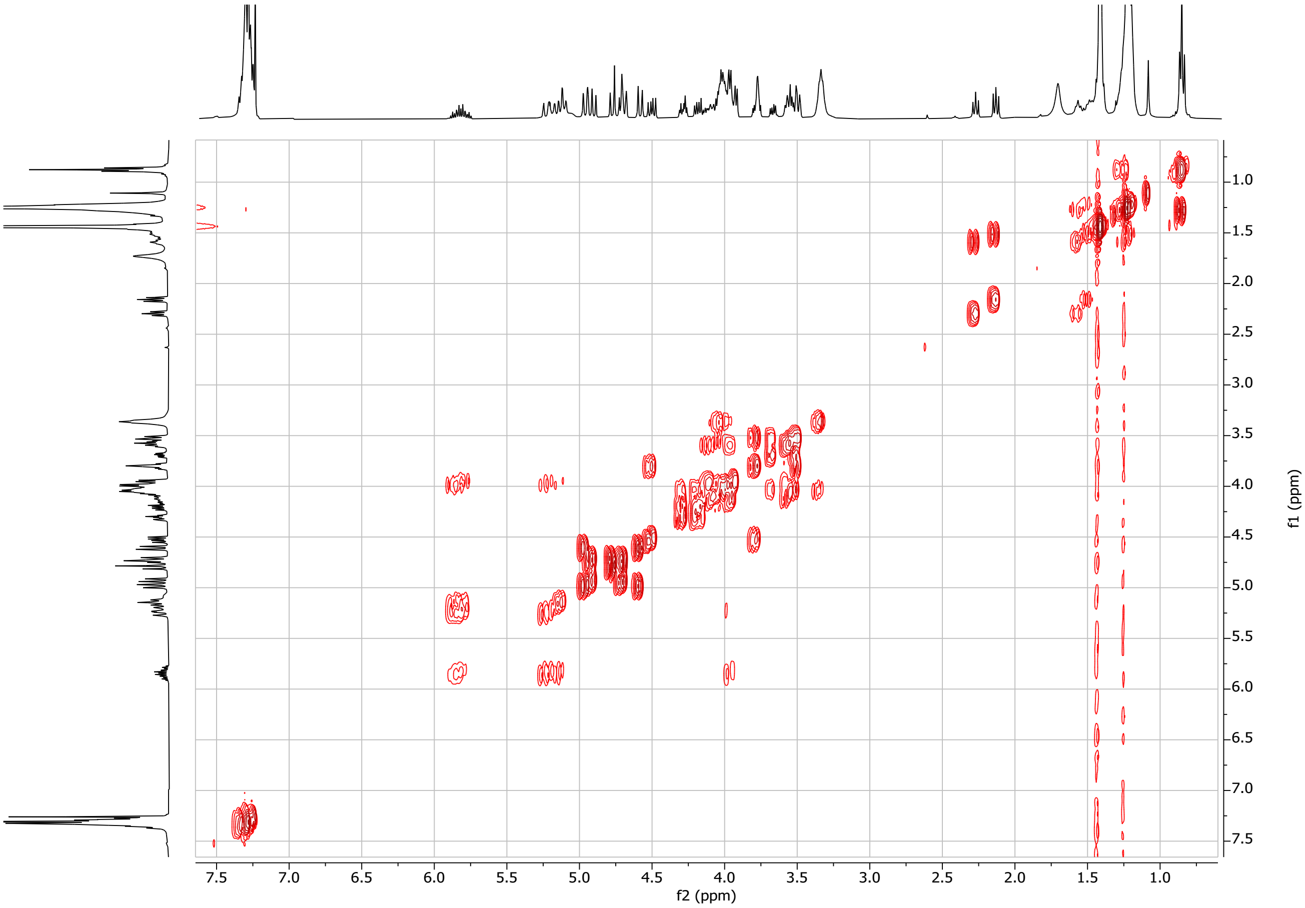

Figure S105 2D NMR COSY ($CDCl_3$) spectrum of **26.**

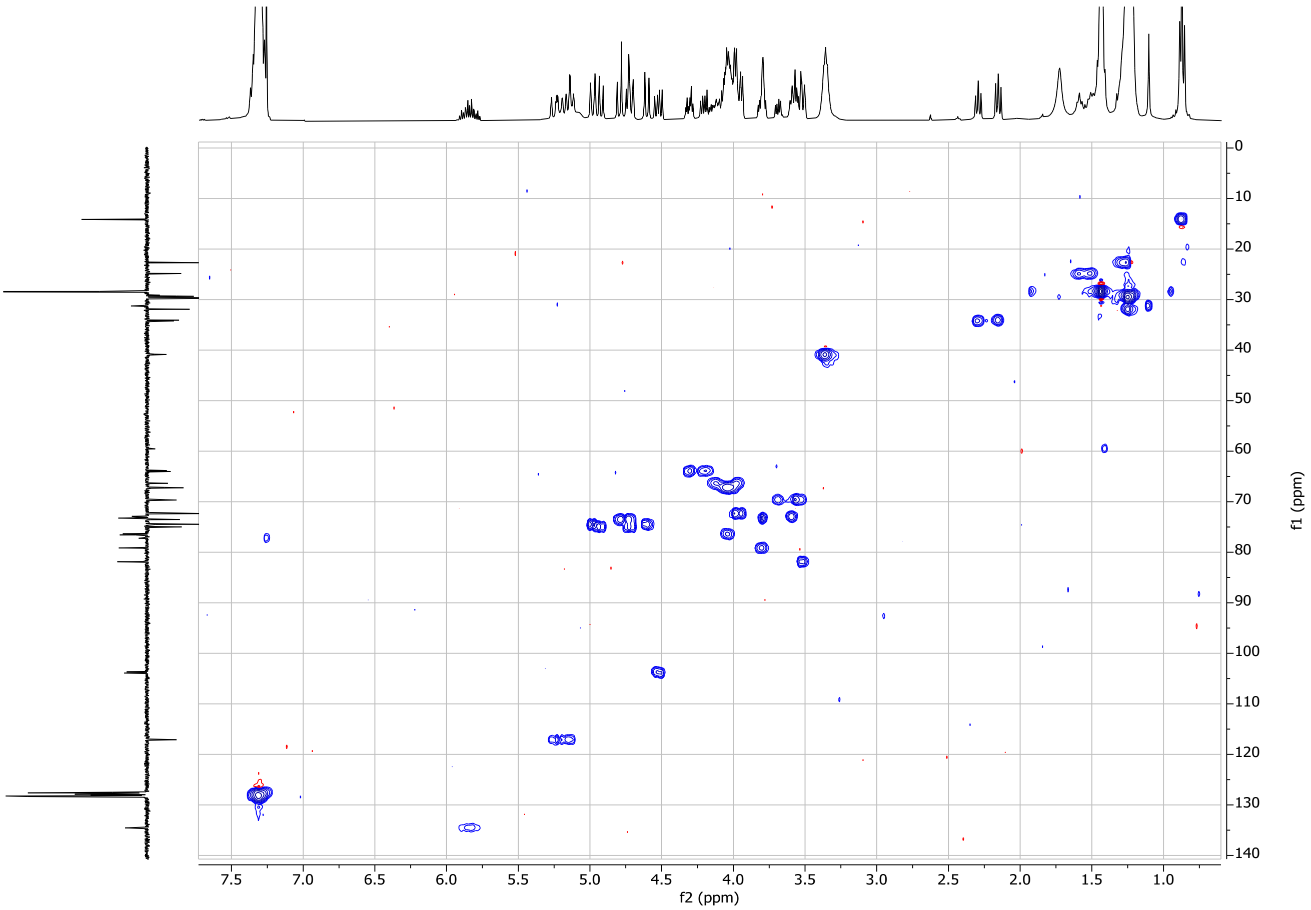

Figure S106 2D NMR HSQC ($CDCl_3$) spectrum of **26.**

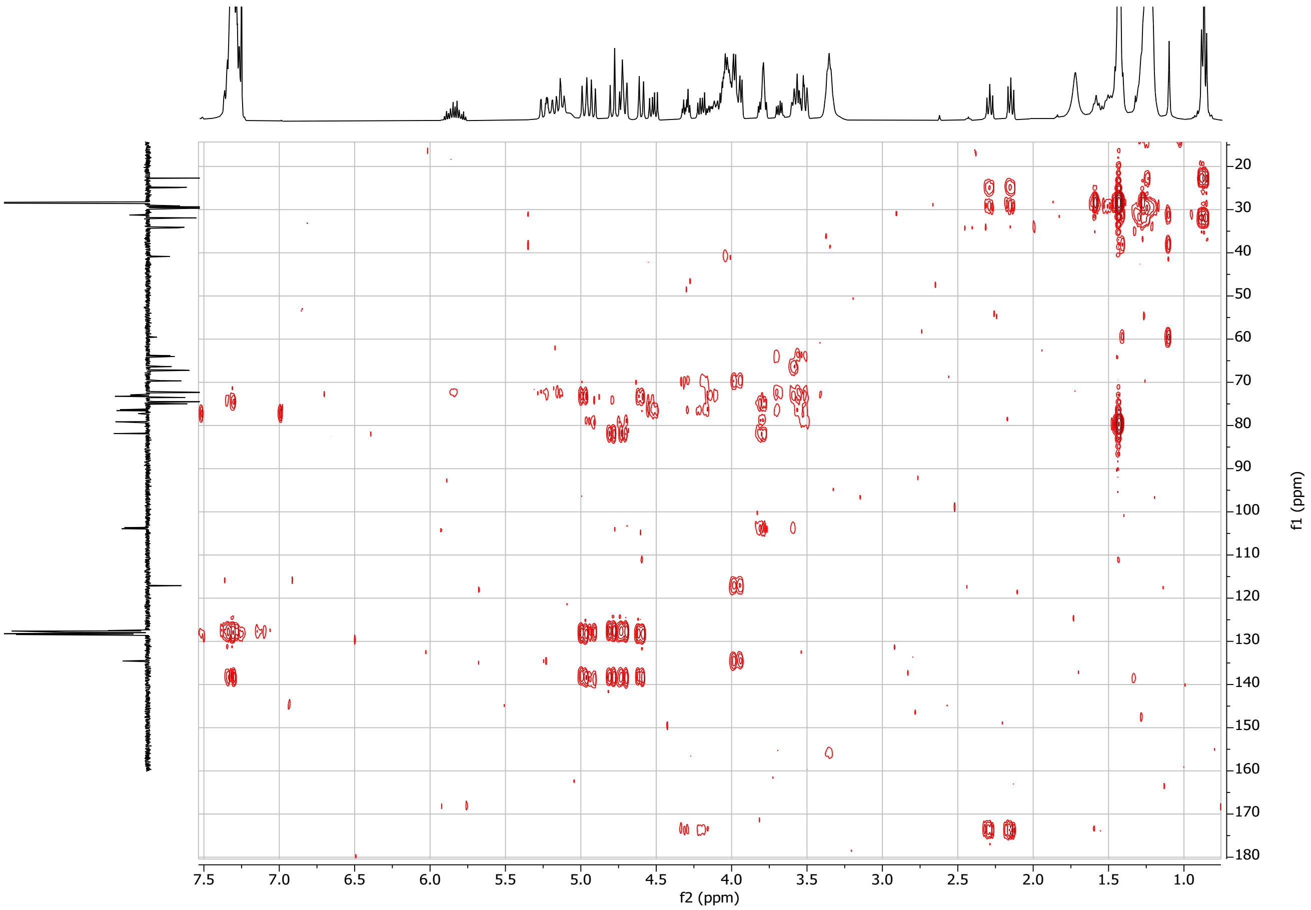

Figure S107 2D NMR HMBC ($CDCl_3$) spectrum of **26.**

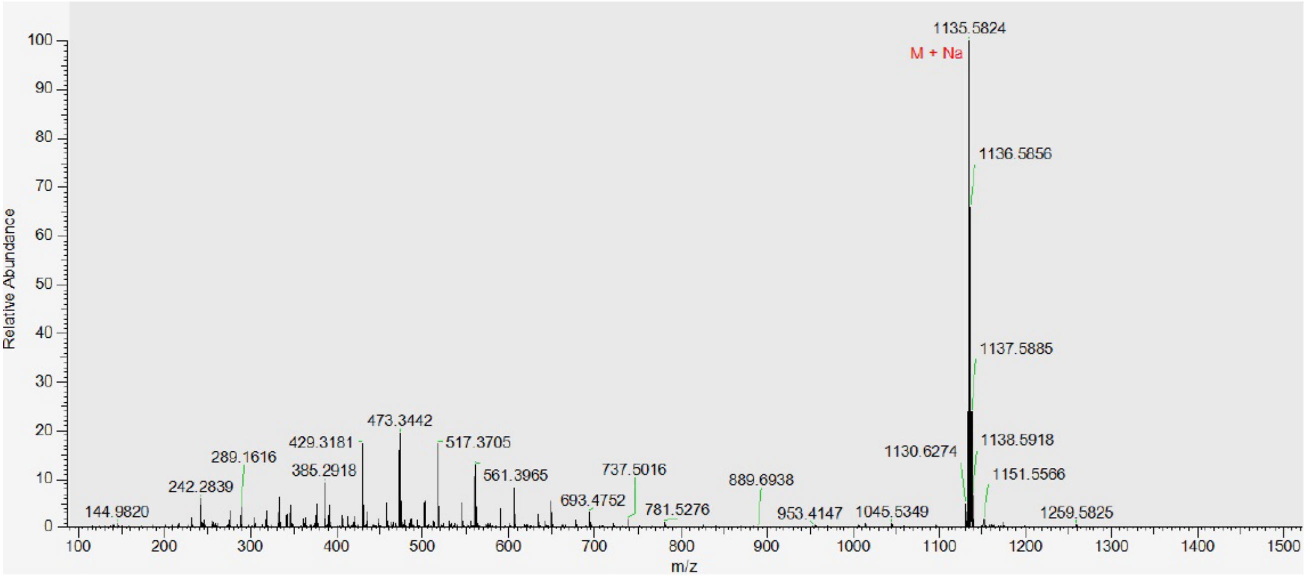

Figure S108 ESI-HRMS spectrum of **26.**

Compound 1a

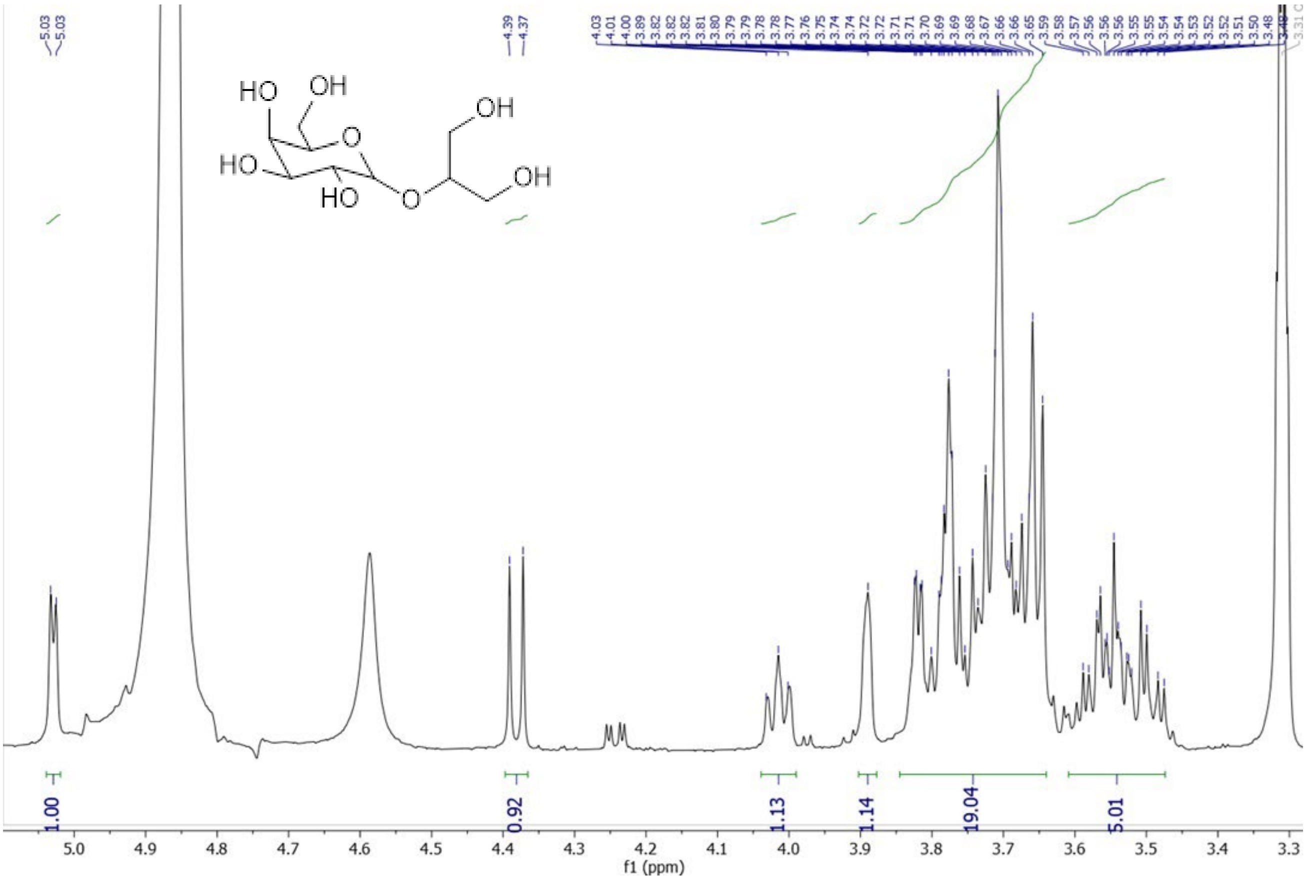

Figure S109 ^{1}H NMR (MeOD) spectrum of **1a.**

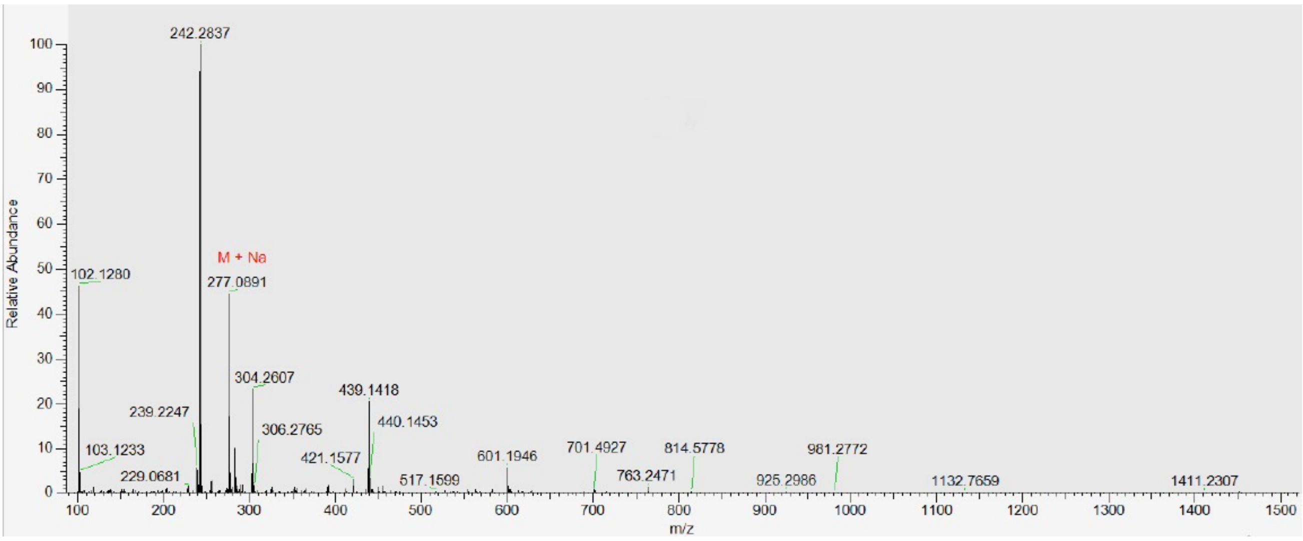

Figure S110 ESI-HRMS spectrum of **1a.**

Compound 1b

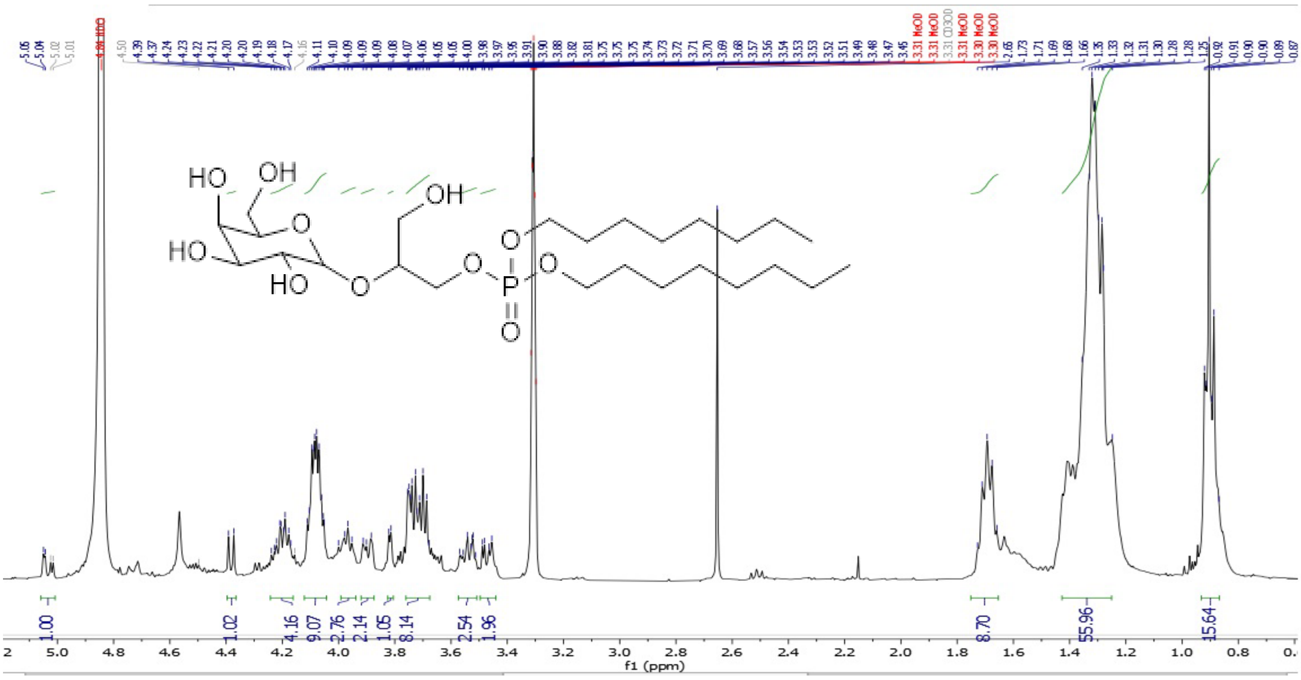

Figure S111 ^{1}H NMR (MeOD) spectrum of **1b.**

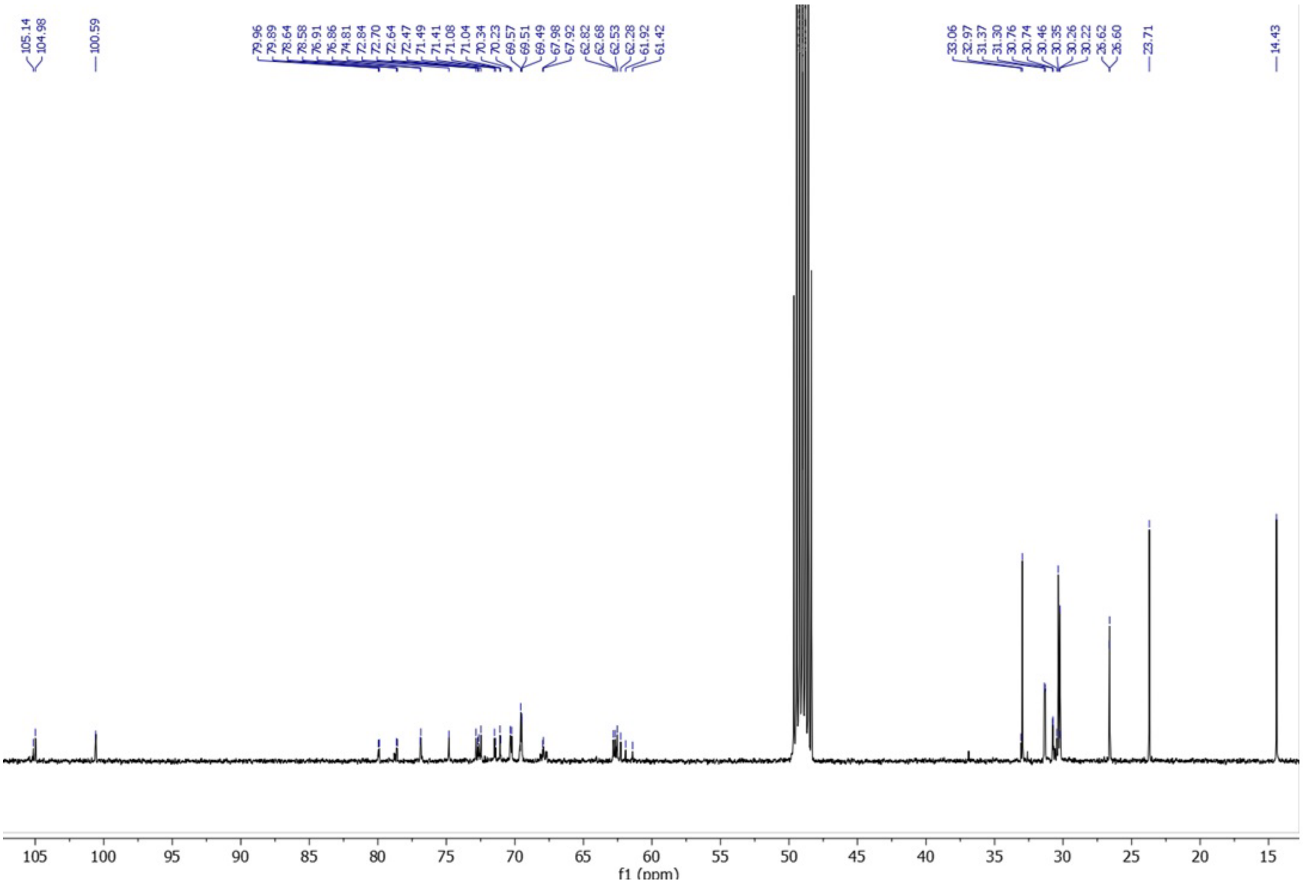

Figure S112 ^{13}C NMR (MeOD) spectrum of **1b.**

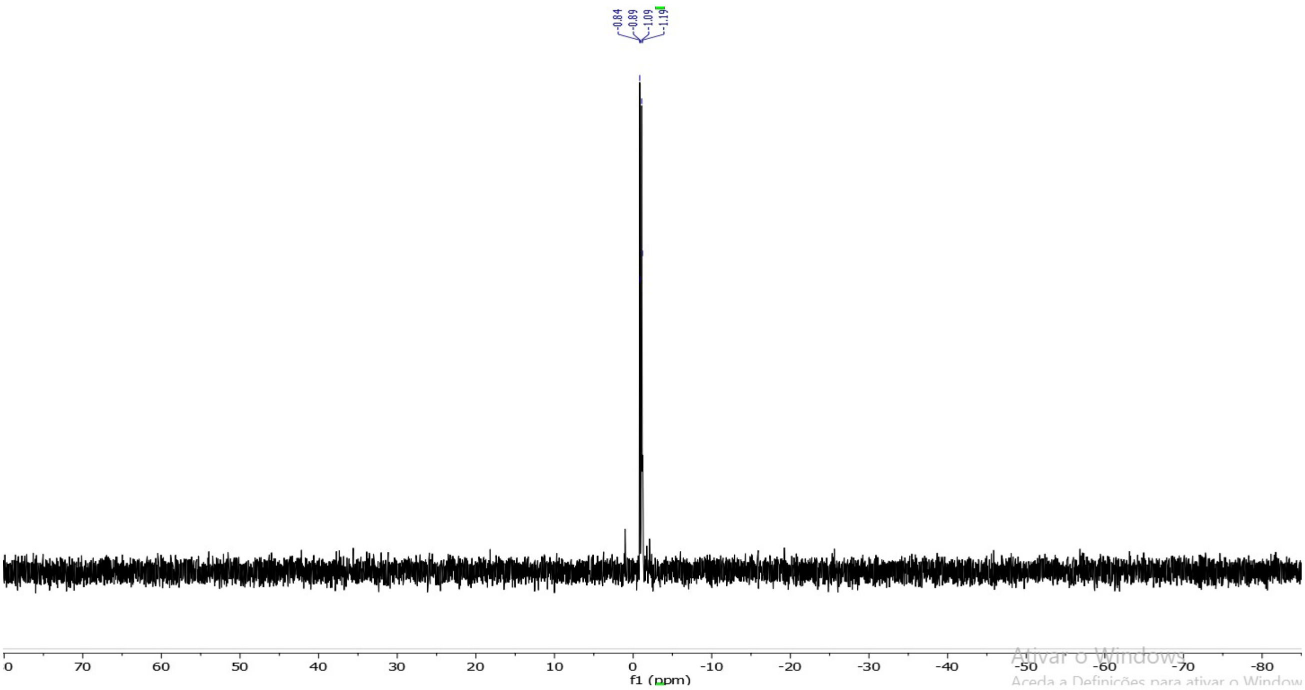

Figure S113 ^{31}P NMR (MeOD) spectrum of **1b.**

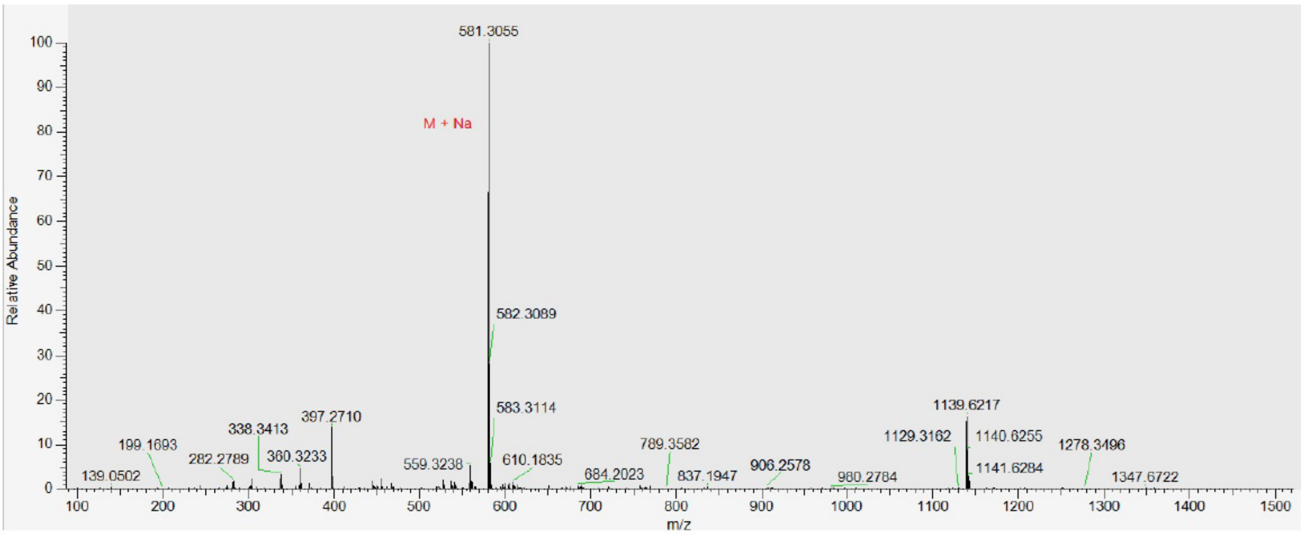

Figure S114 ESI-HRMS spectrum of **1b.**

Compound 1c

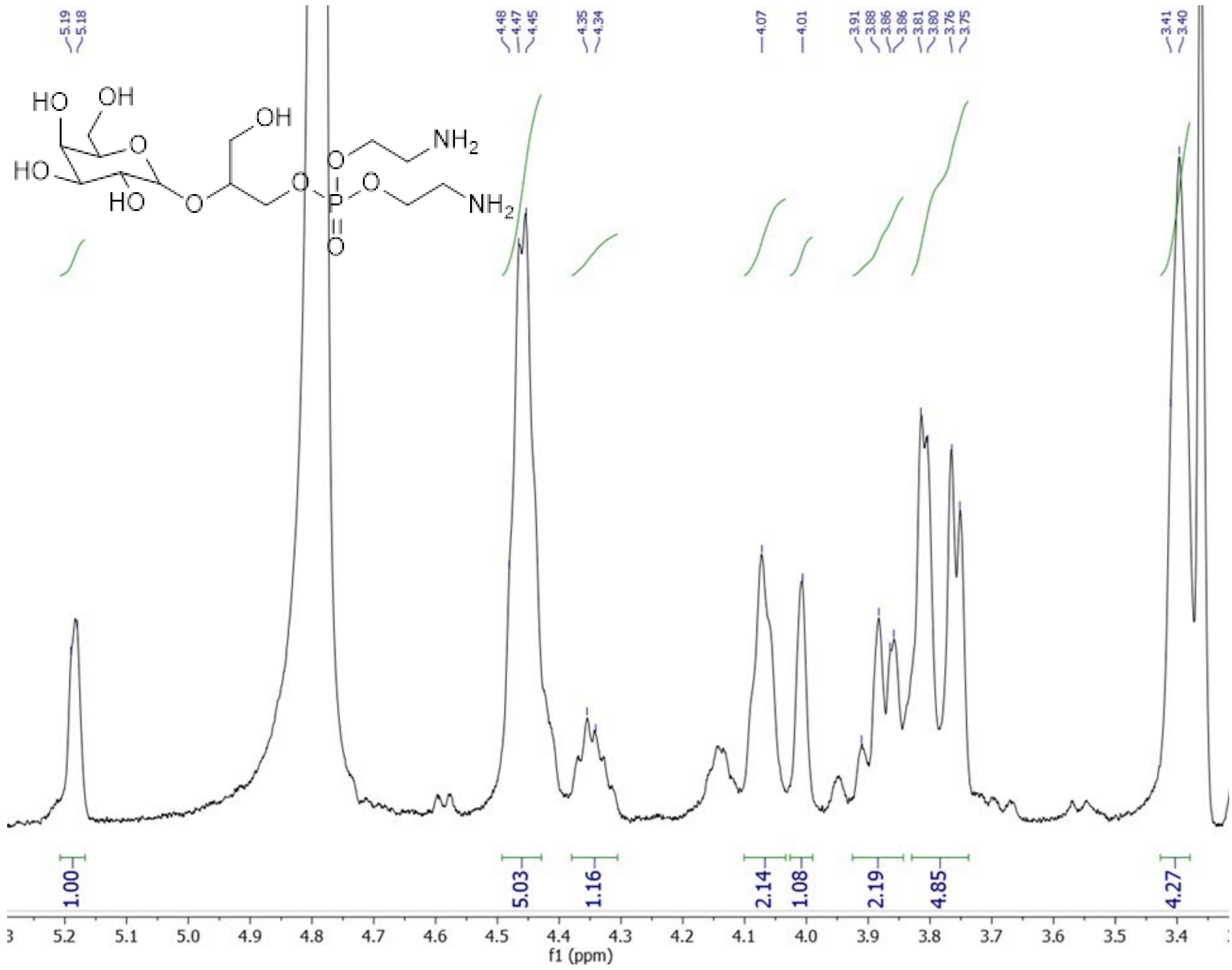

Figure S115 ^{1}H NMR (D_2O) spectrum of **1c.**

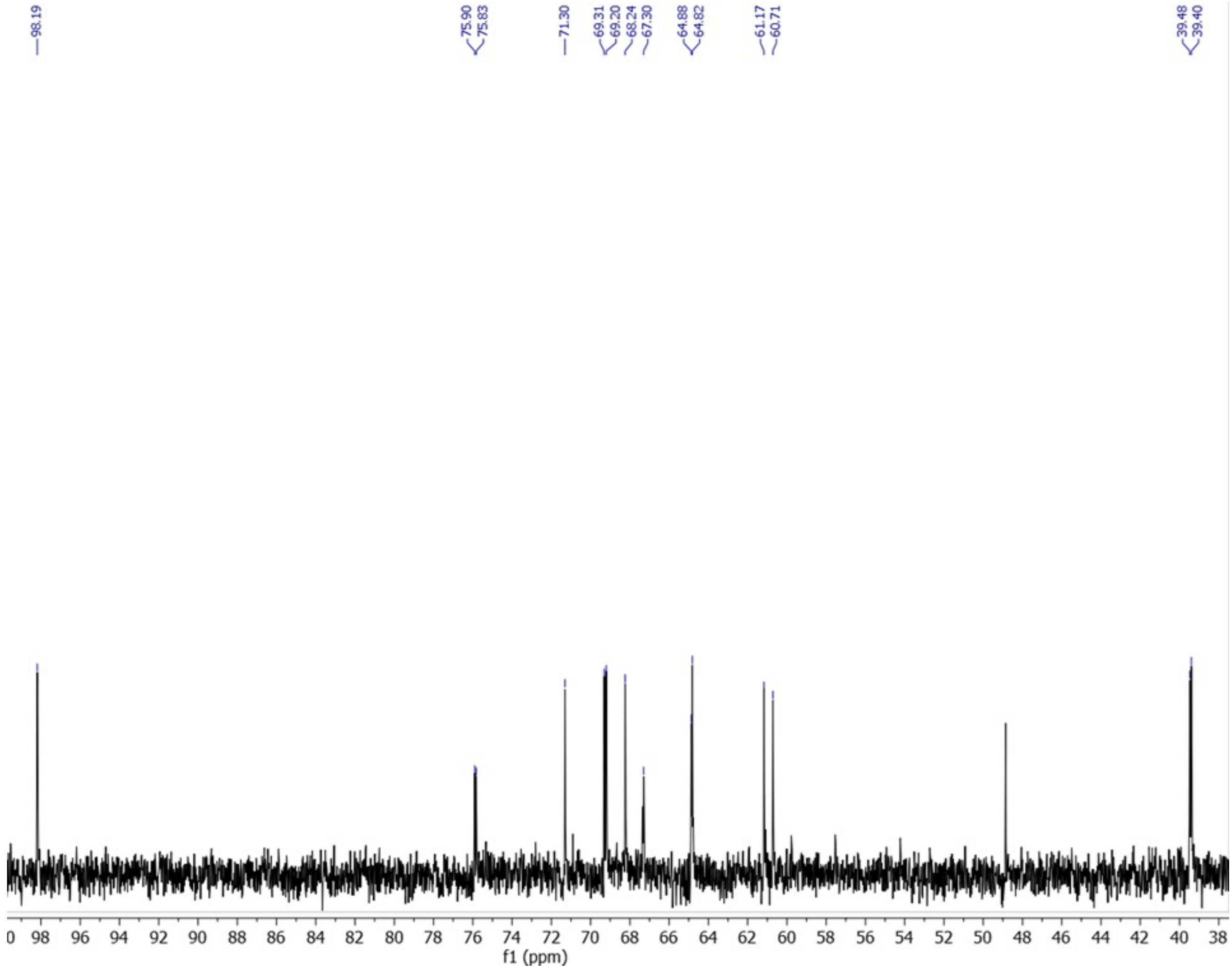

Figure S116 ^{13}C NMR (D_2O) spectrum of **1c.**

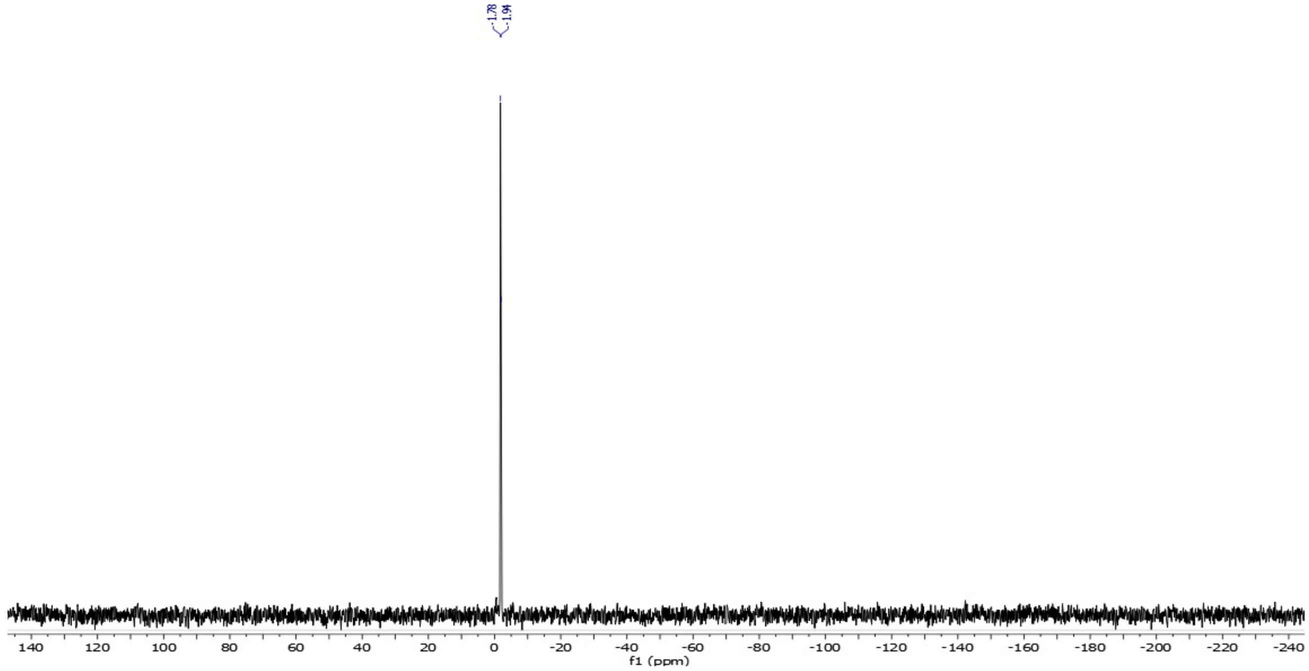

Figure S117 ^{31}P NMR (D_2O) spectrum of **1c.**

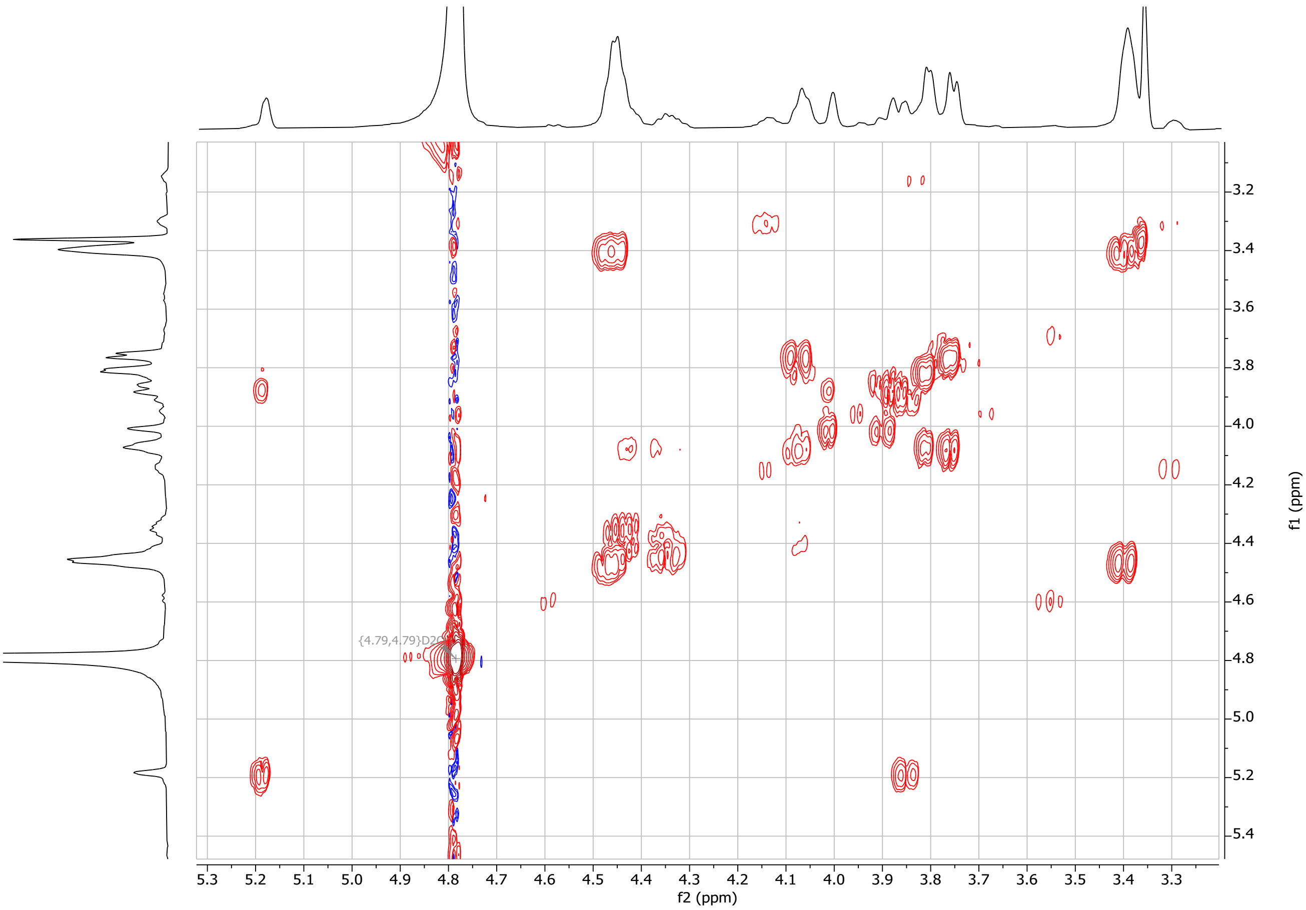

Figure S118 2D NMR COSY (D_2O) spectrum of **1c.**

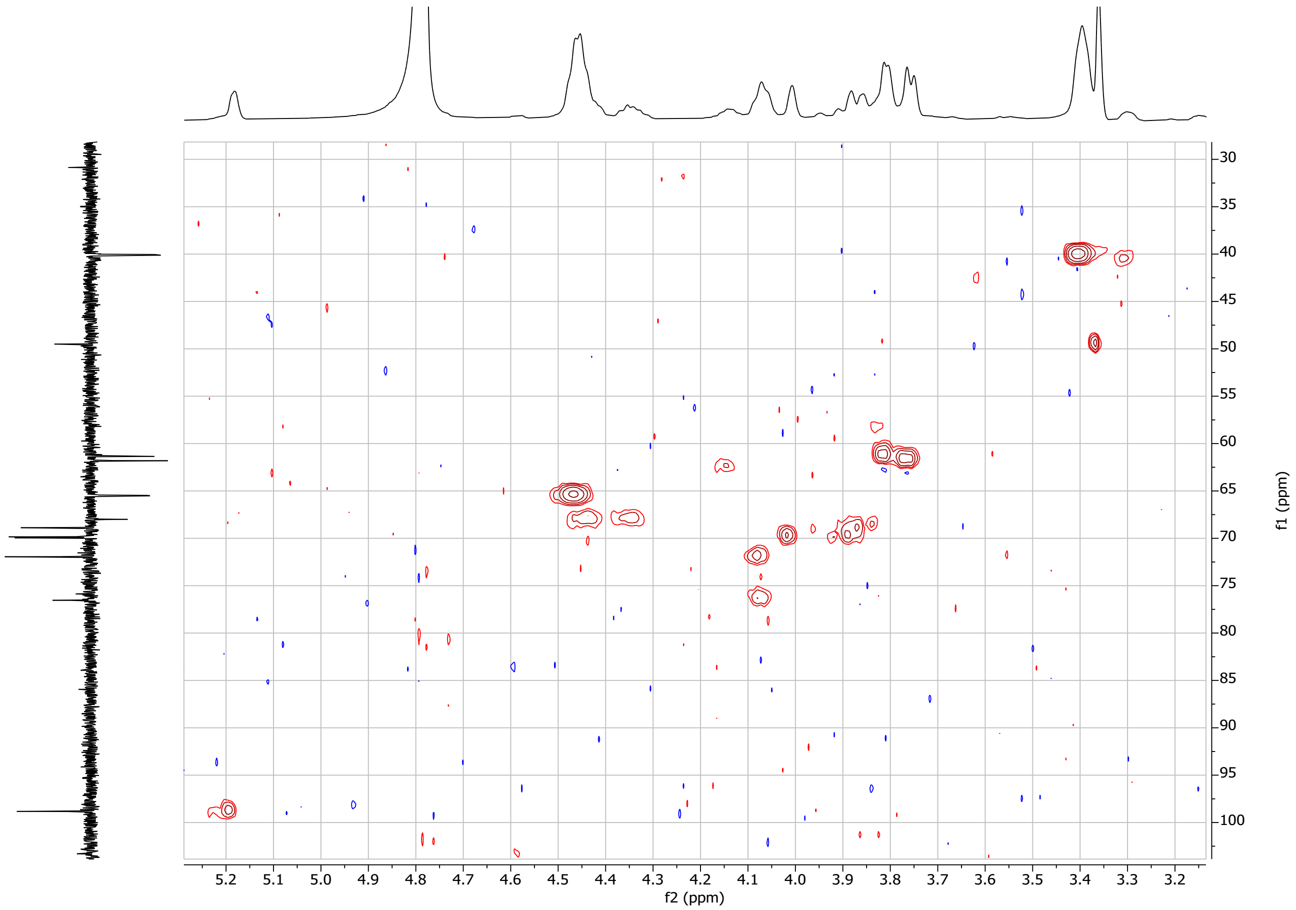

Figure S119 2D NMR HSQC (D_2O) spectrum of **1c.**

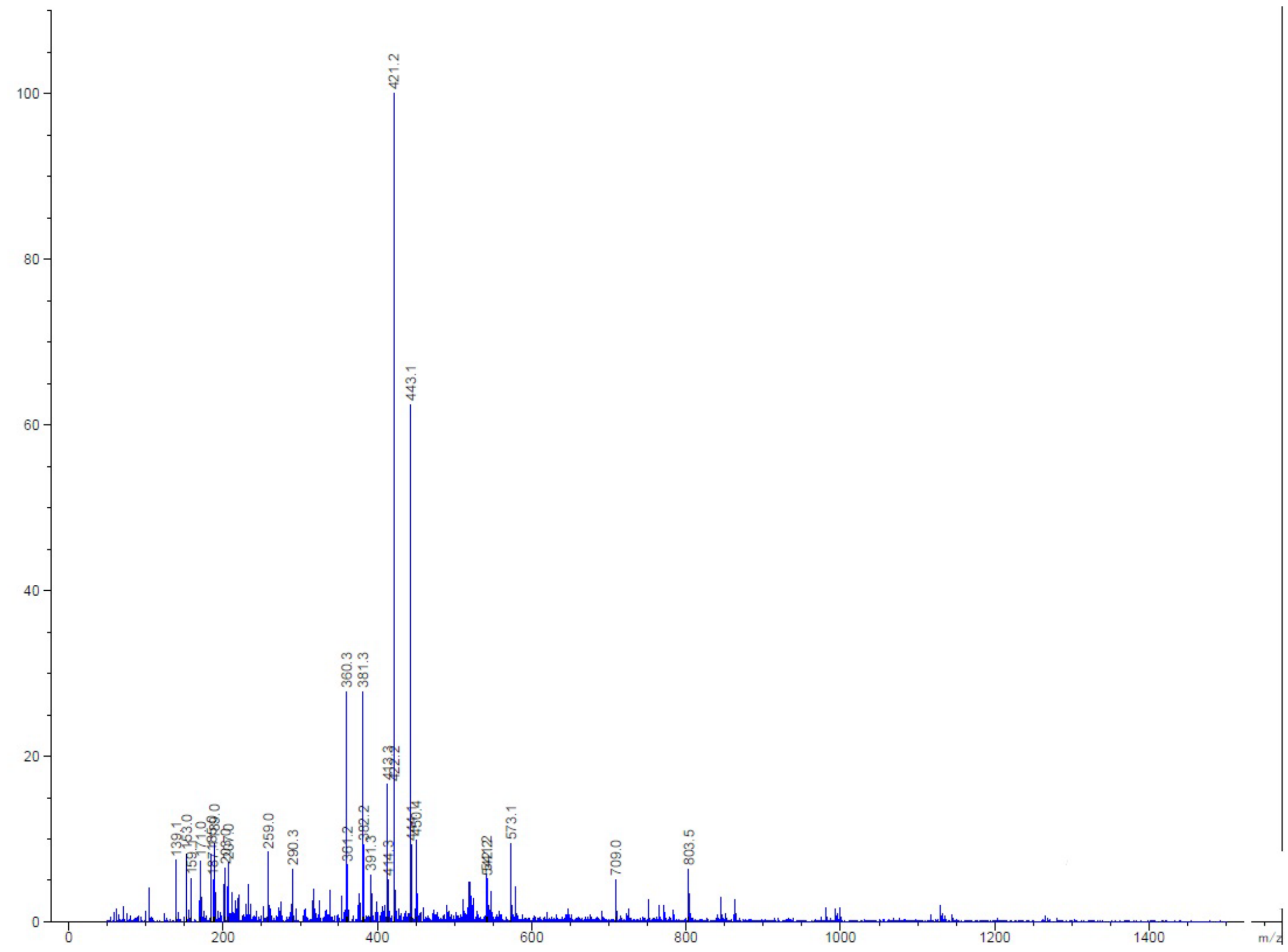

Figure S120 LCMS spectrum of **1c.**

Compound 1d

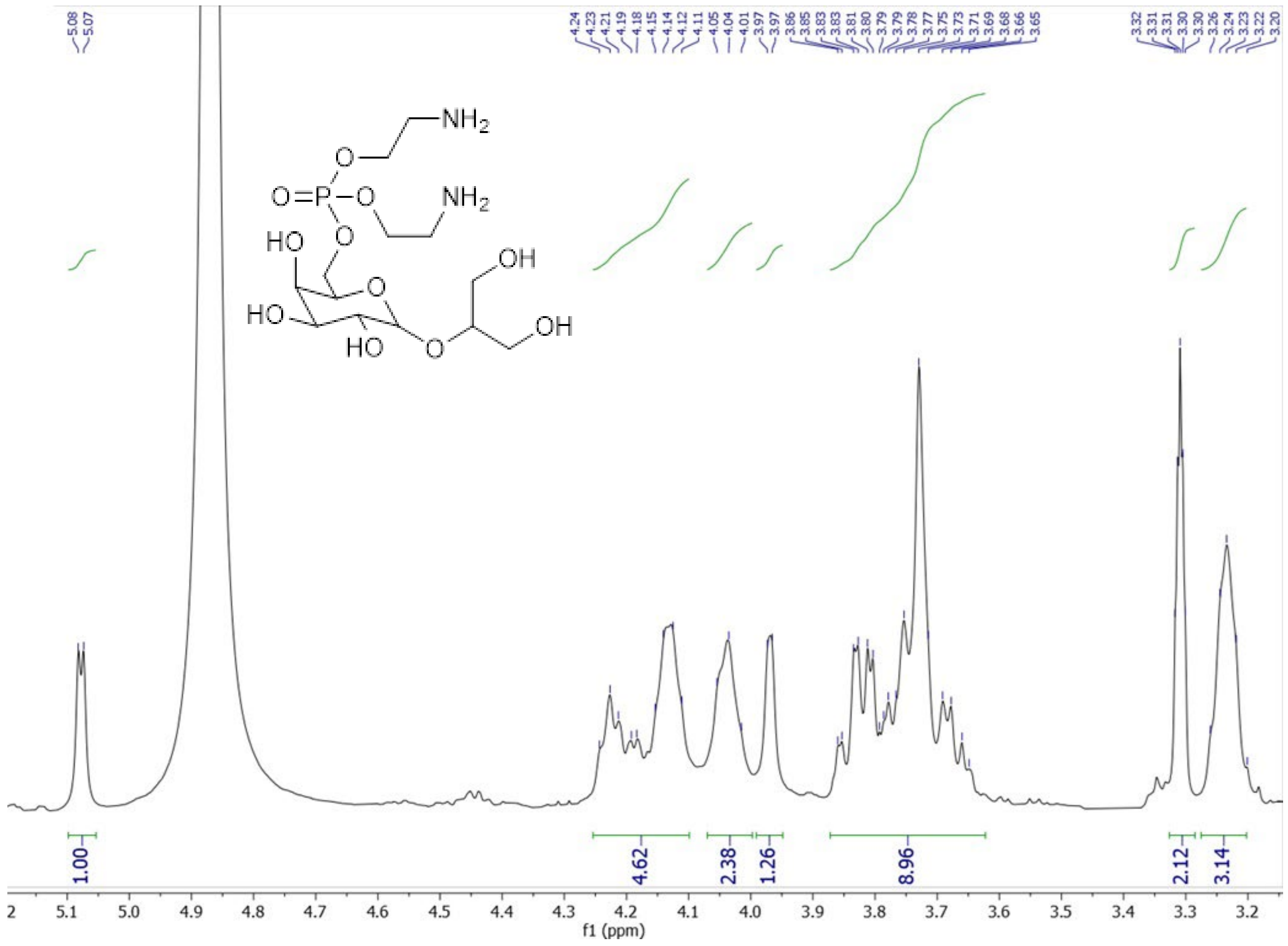

Figure S121 ^1H NMR (D_2O) spectrum of **1d.**

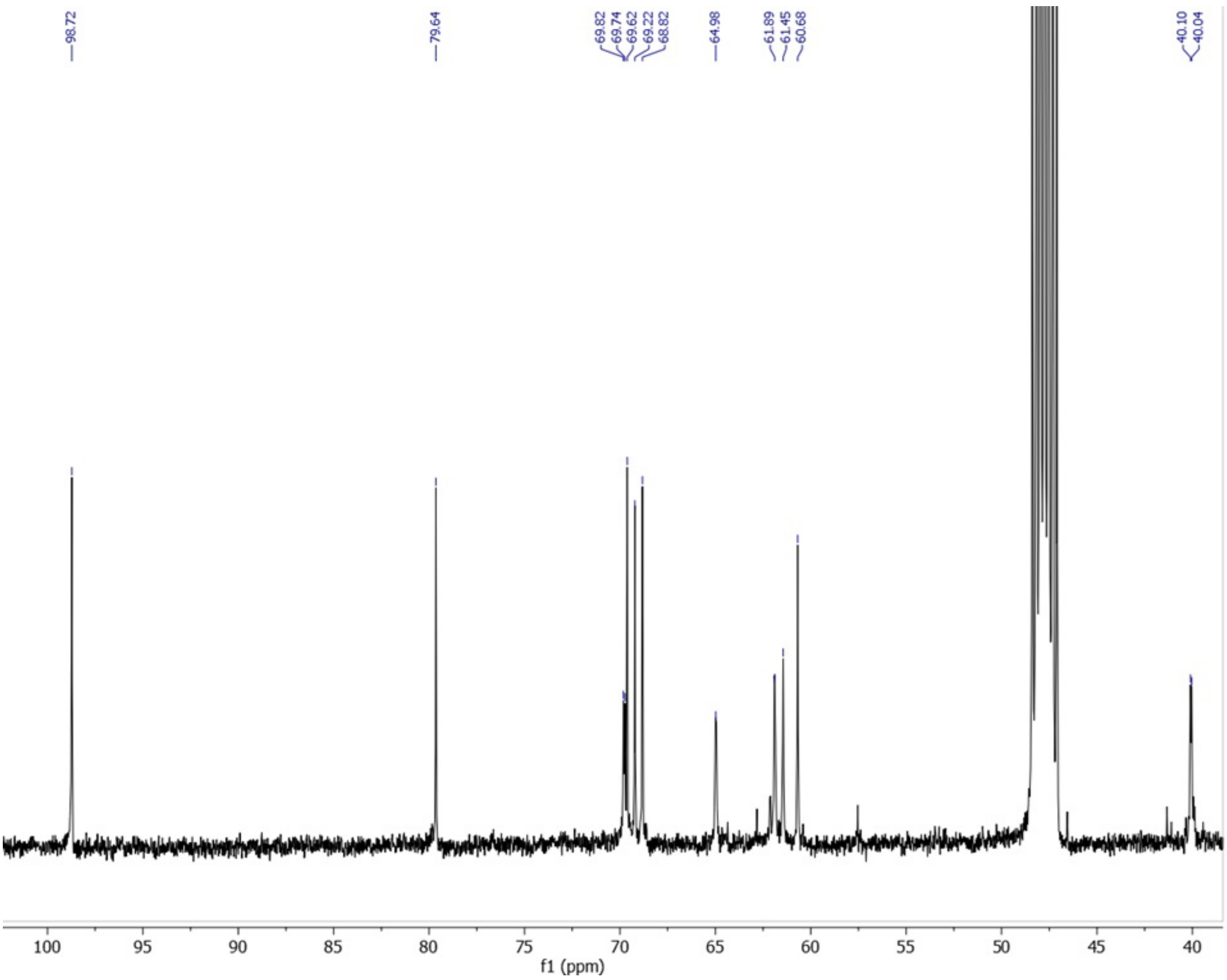

Figure S122 ^{13}C NMR (D_2O + MeOH) spectrum of **1d.**

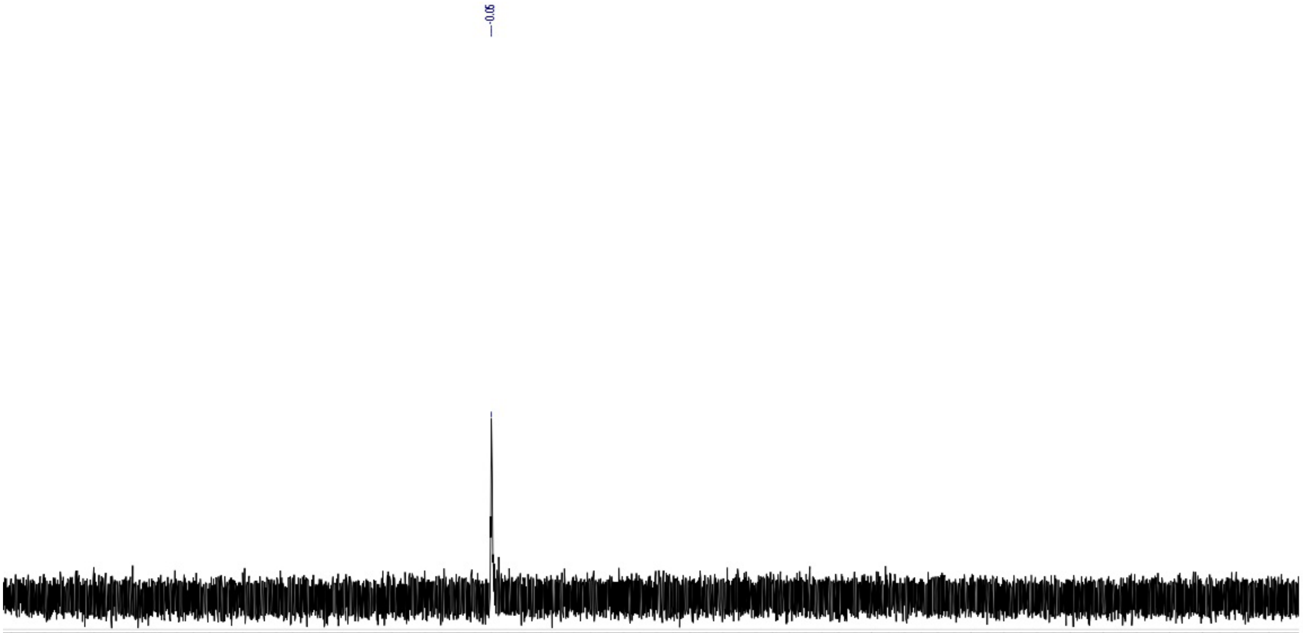

Figure S123 ^{31}P NMR (D_2O + MeOH) spectrum of **1d.**

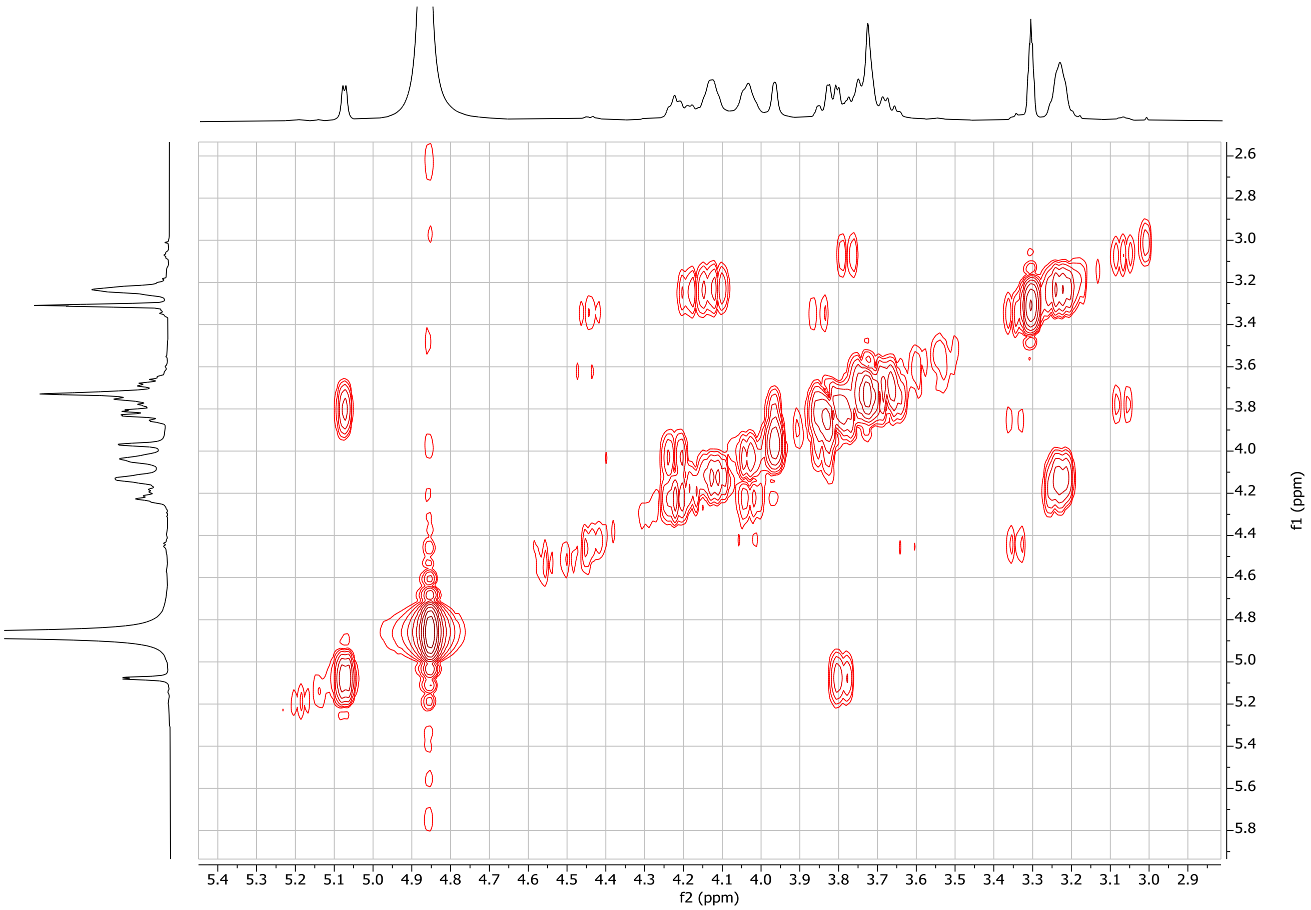

Figure S124 2D NMR COSY (D_2O + MeOH) spectrum of **1d.**

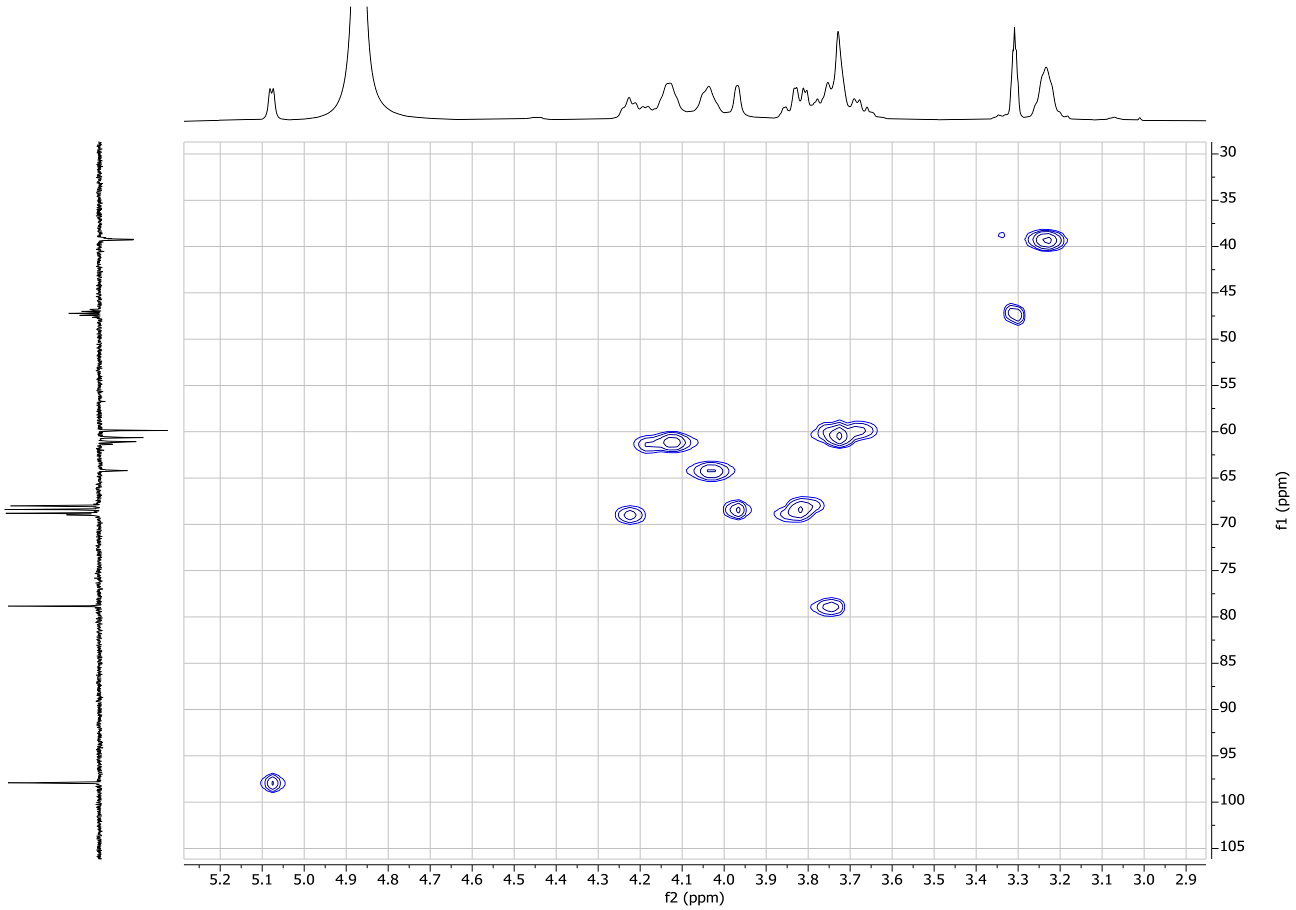

Figure S125 2D NMR HSQC (D_2O + MeOH) spectrum of **1d.**

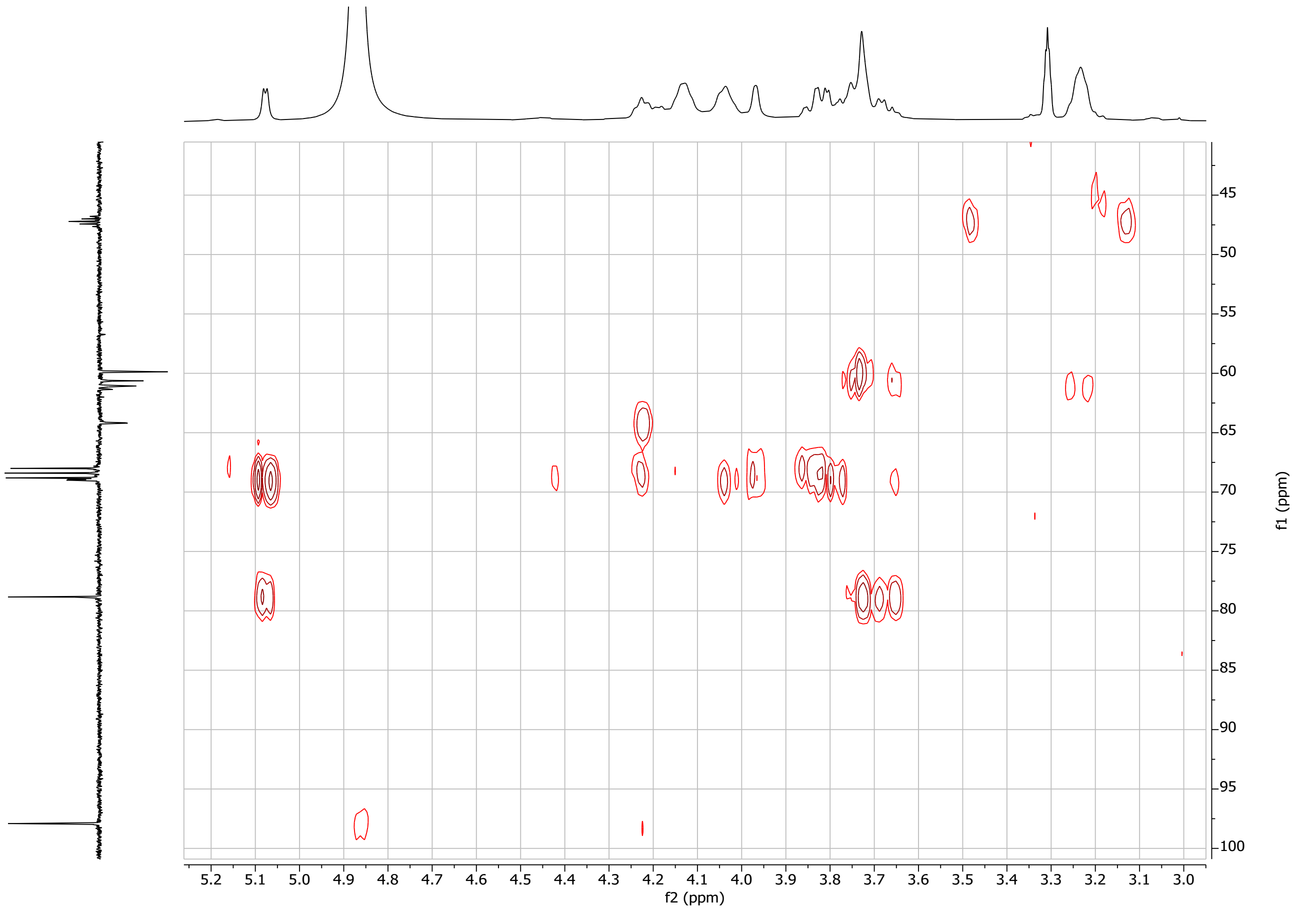

Figure S126 2D NMR HMBC (D_2O + MeOH) spectrum of **1d.**

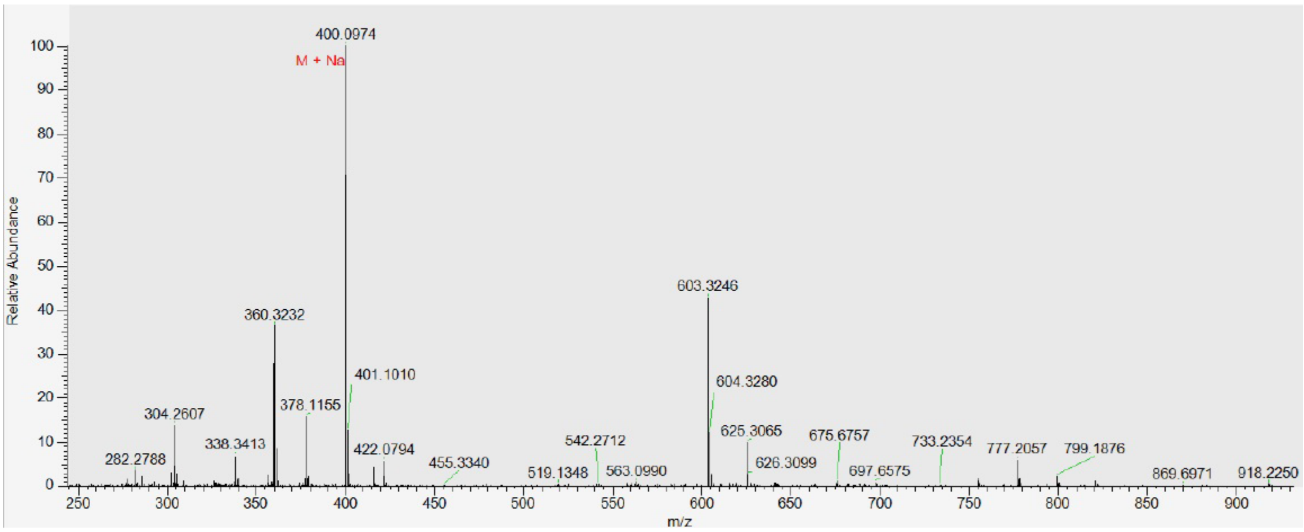

Figure S127 ESI-HRMS spectrum of **1d.**

Compound 1e

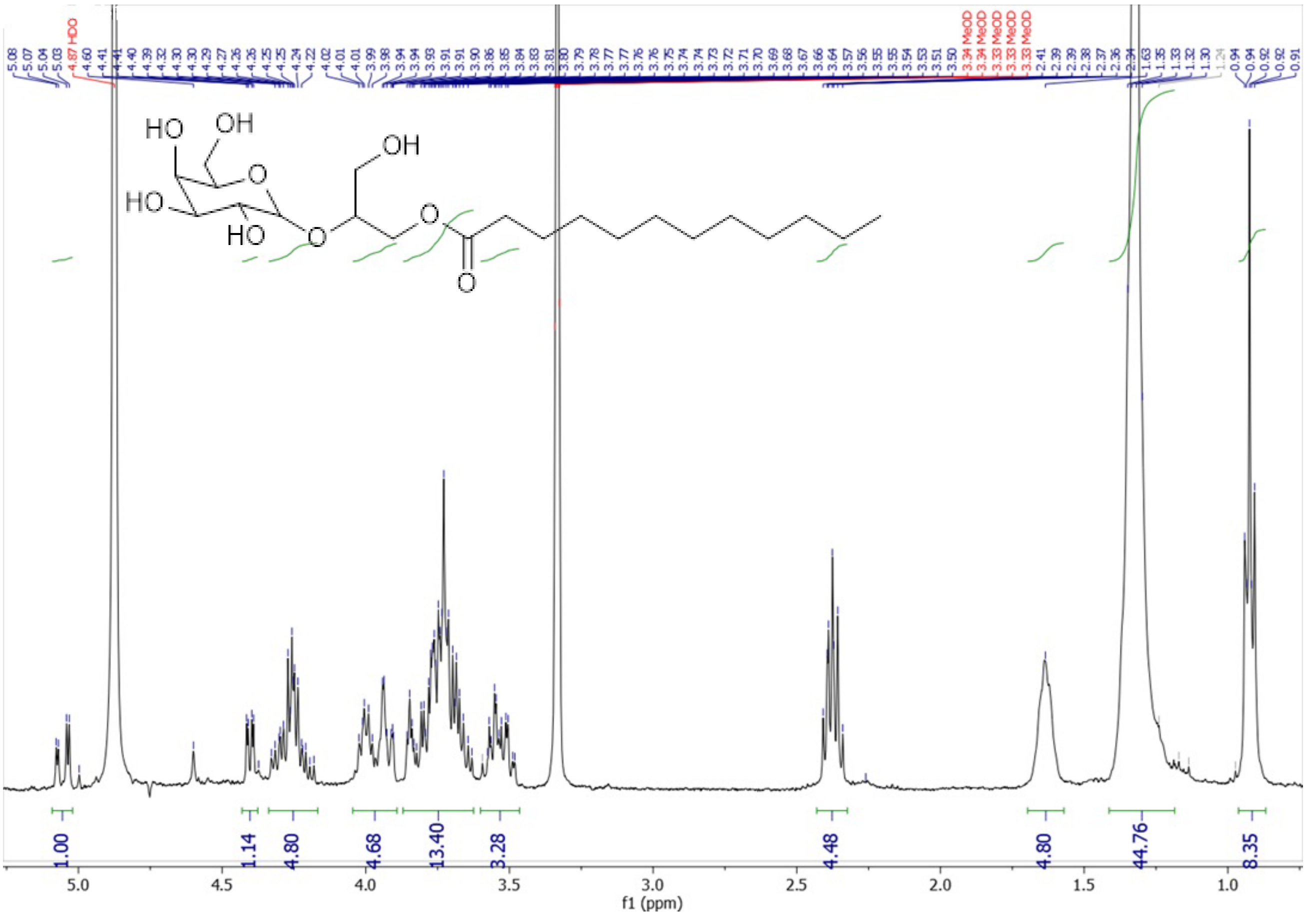

Figure S128 ^{1}H NMR (MeOD) spectrum of **1e.**

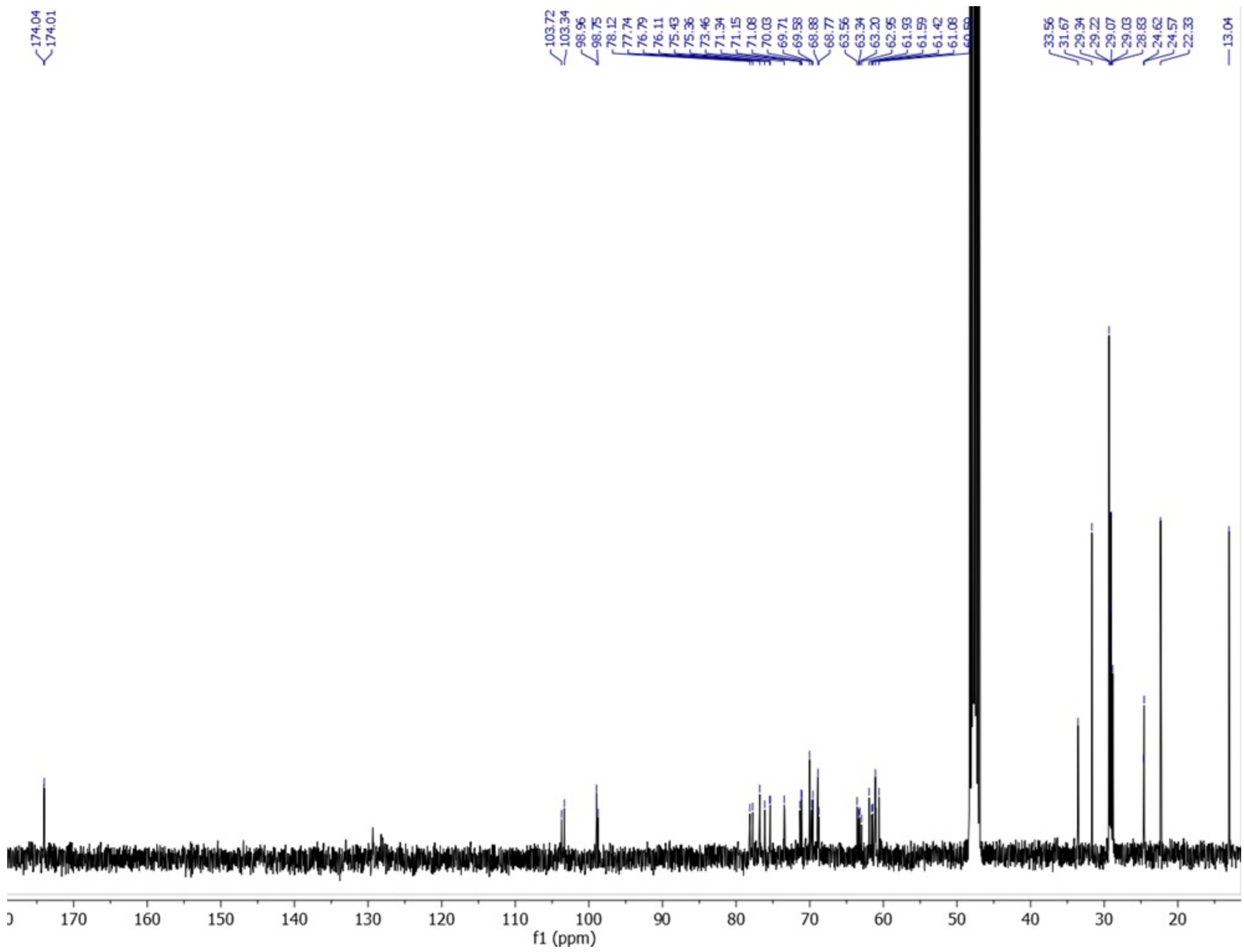

Figure S129 ^{13}C NMR (MeOD) spectrum of **1e.**

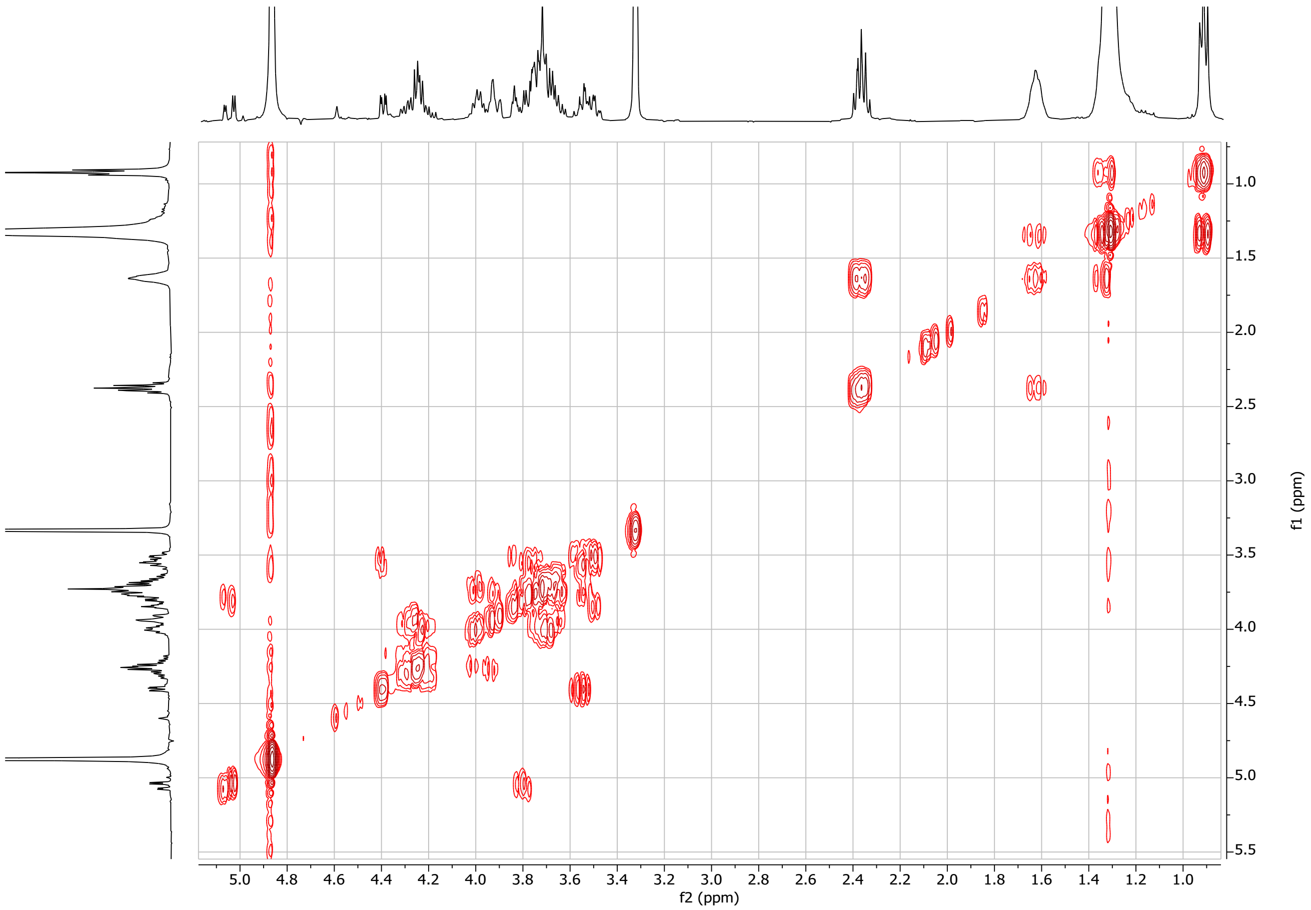

Figure S130 2D NMR COSY (MeOD) spectrum of **1e.**

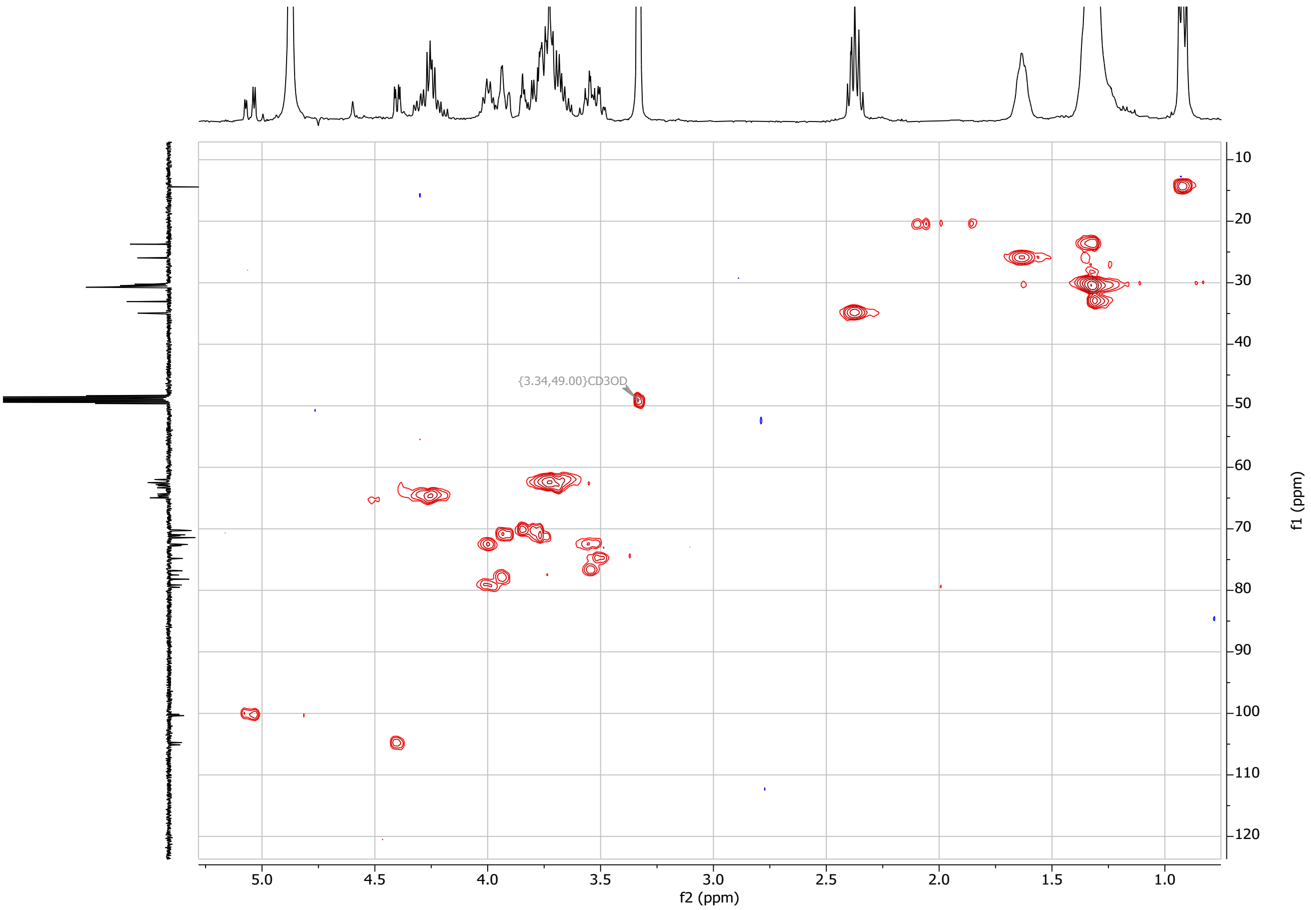

Figure S131 2D NMR HSQC (MeOD) spectrum of **1e.**

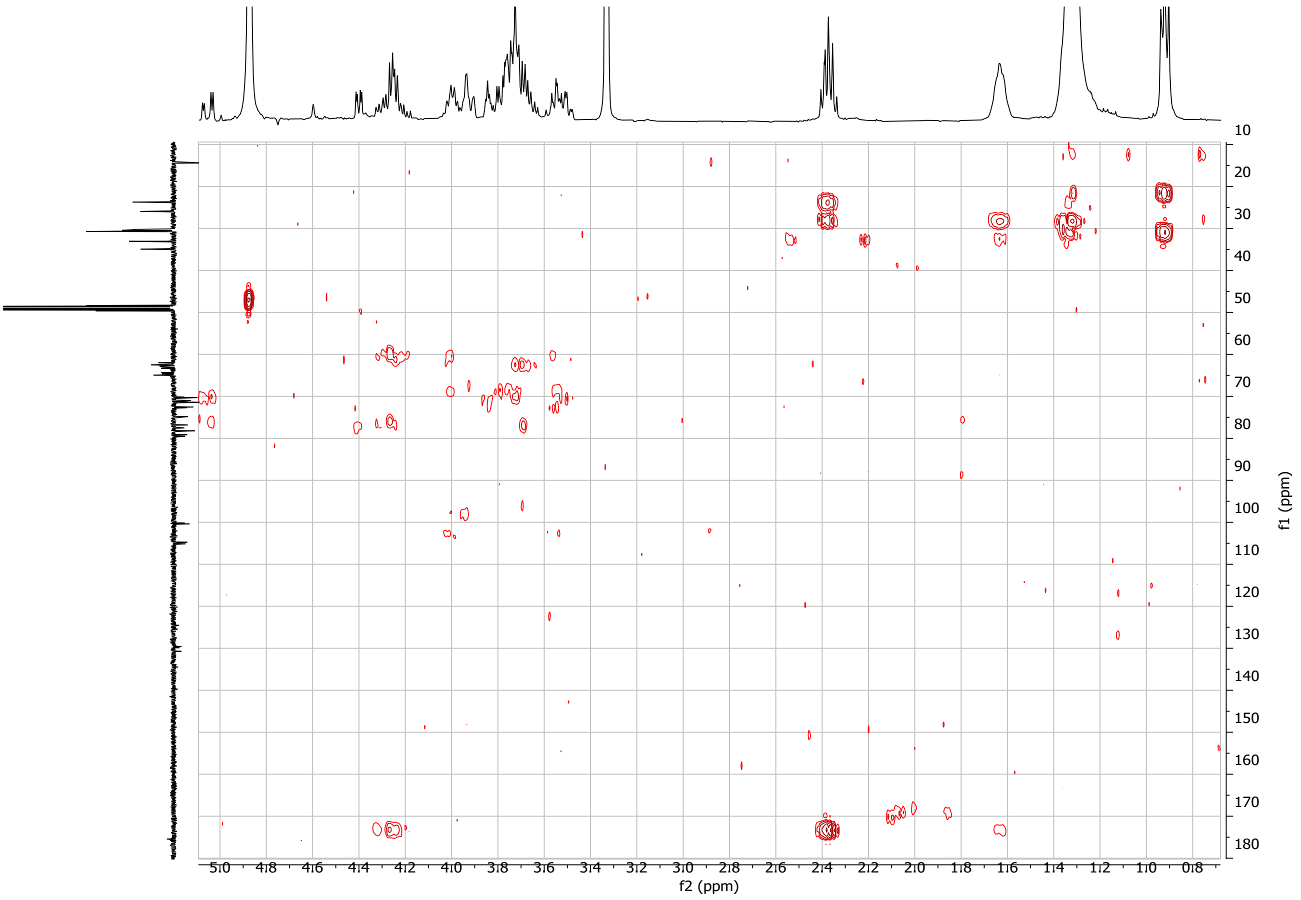

Figure S132 2D NMR HMBC (MeOD) spectrum of **1e.**

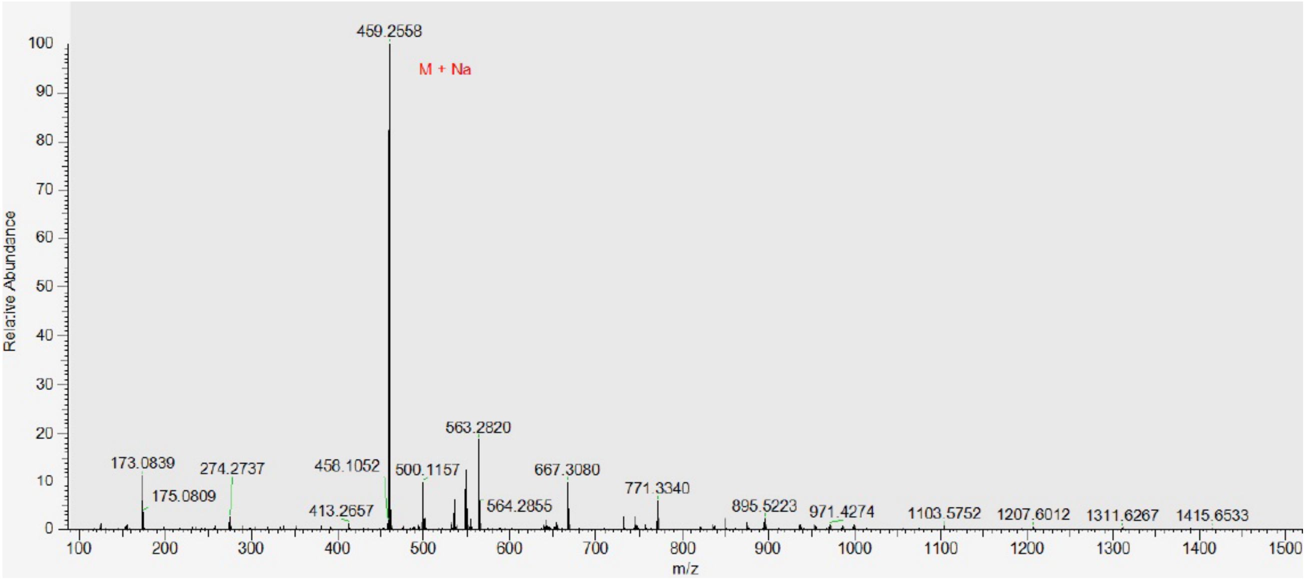

Figure S133 ESI-HRMS spectrum of **1e.**

Compound 1f

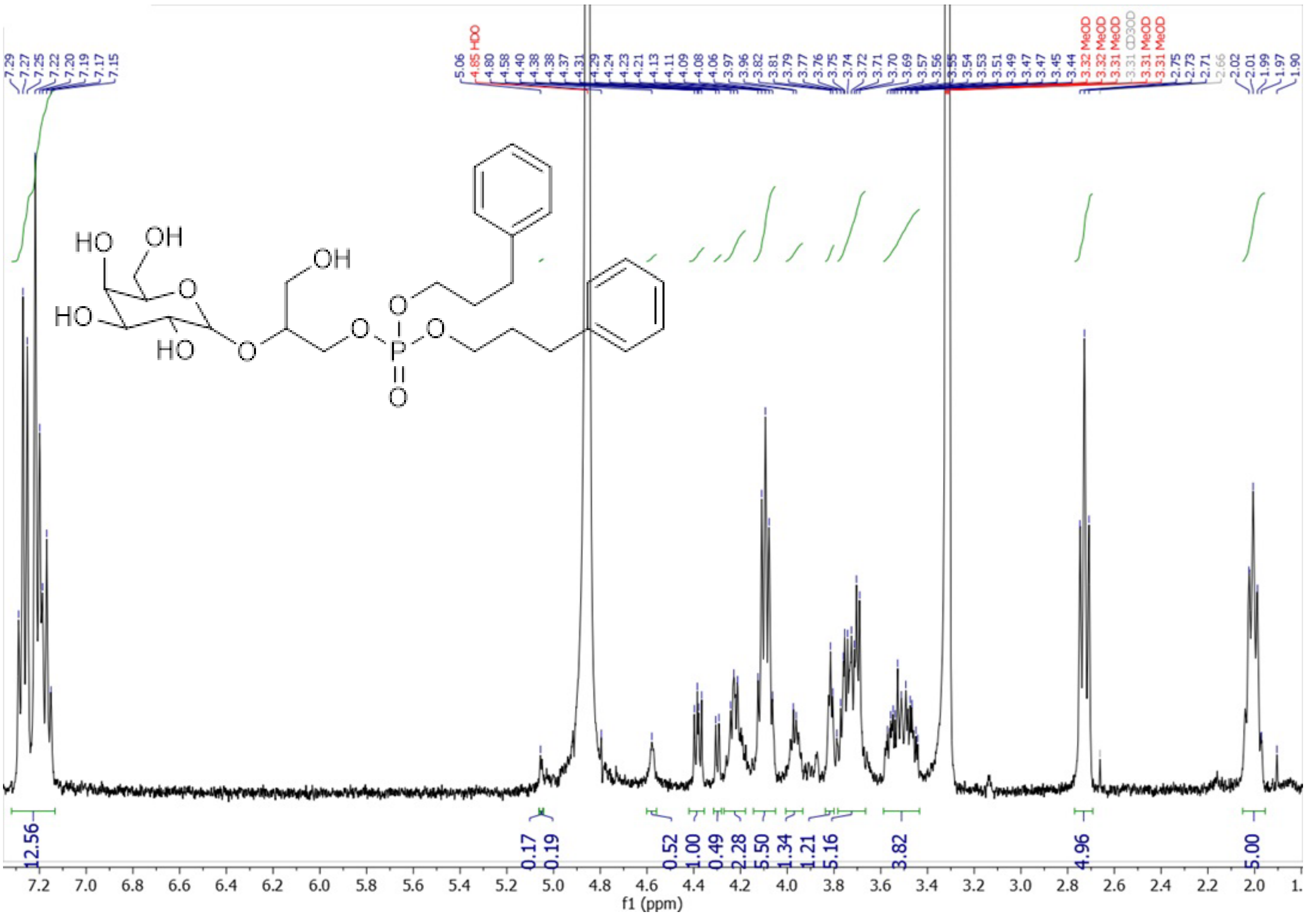

Figure S134 ^{1}H NMR (MeOD) spectrum of **1f.**

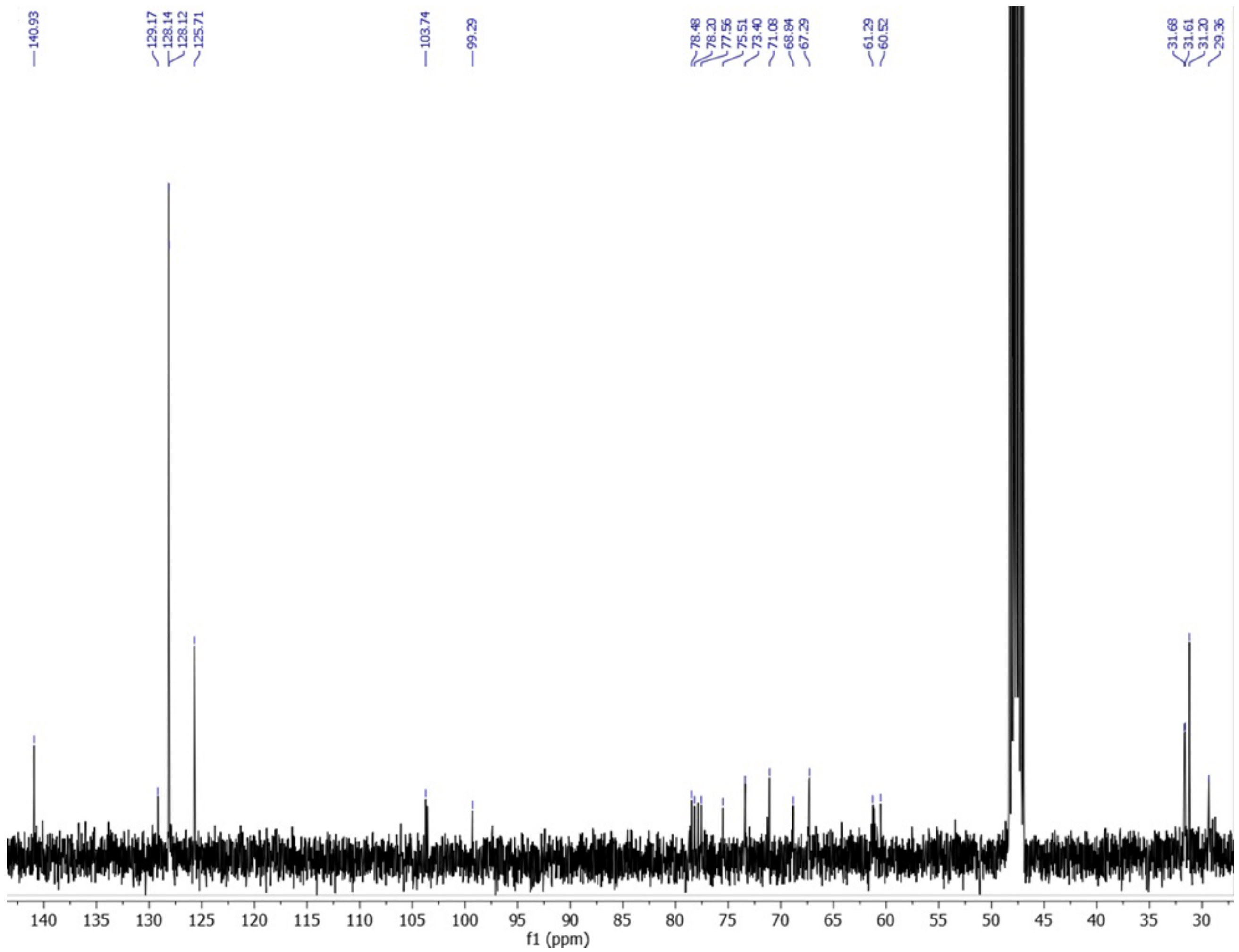

Figure S135 ^{13}C NMR (MeOD) spectrum of **1f.**

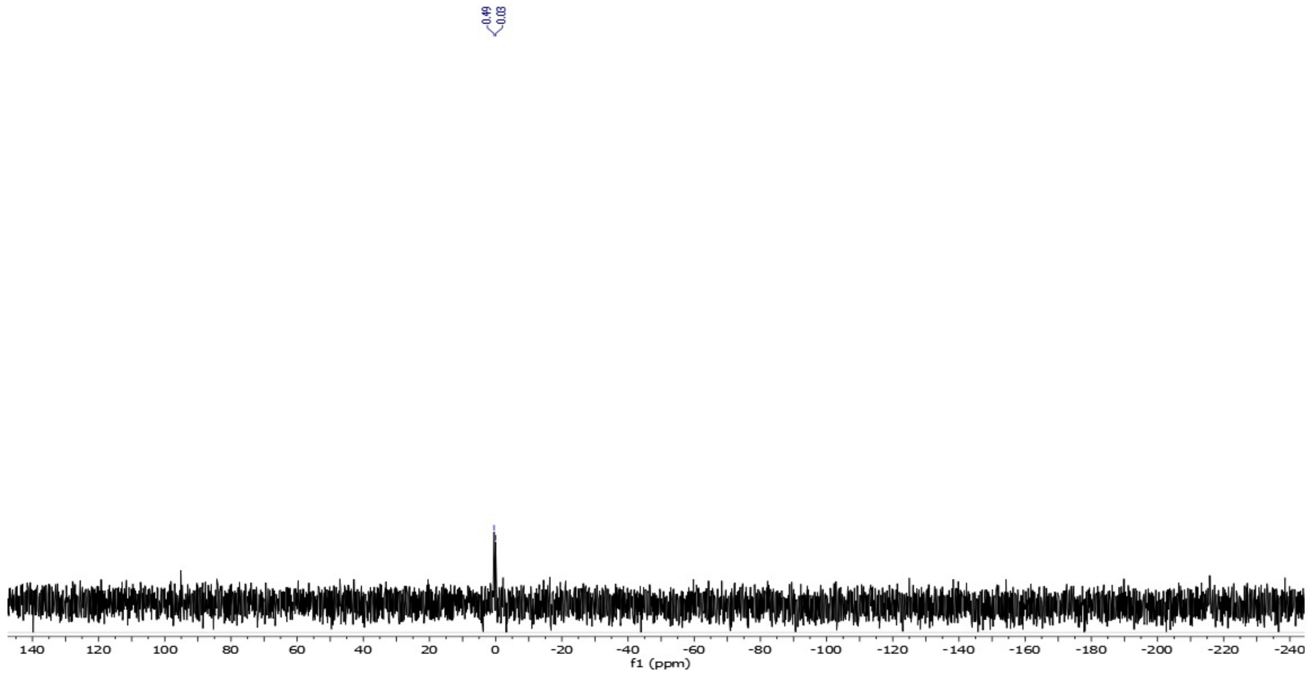

Figure S136 ^{31}P NMR (MeOD) spectrum of **1f.**

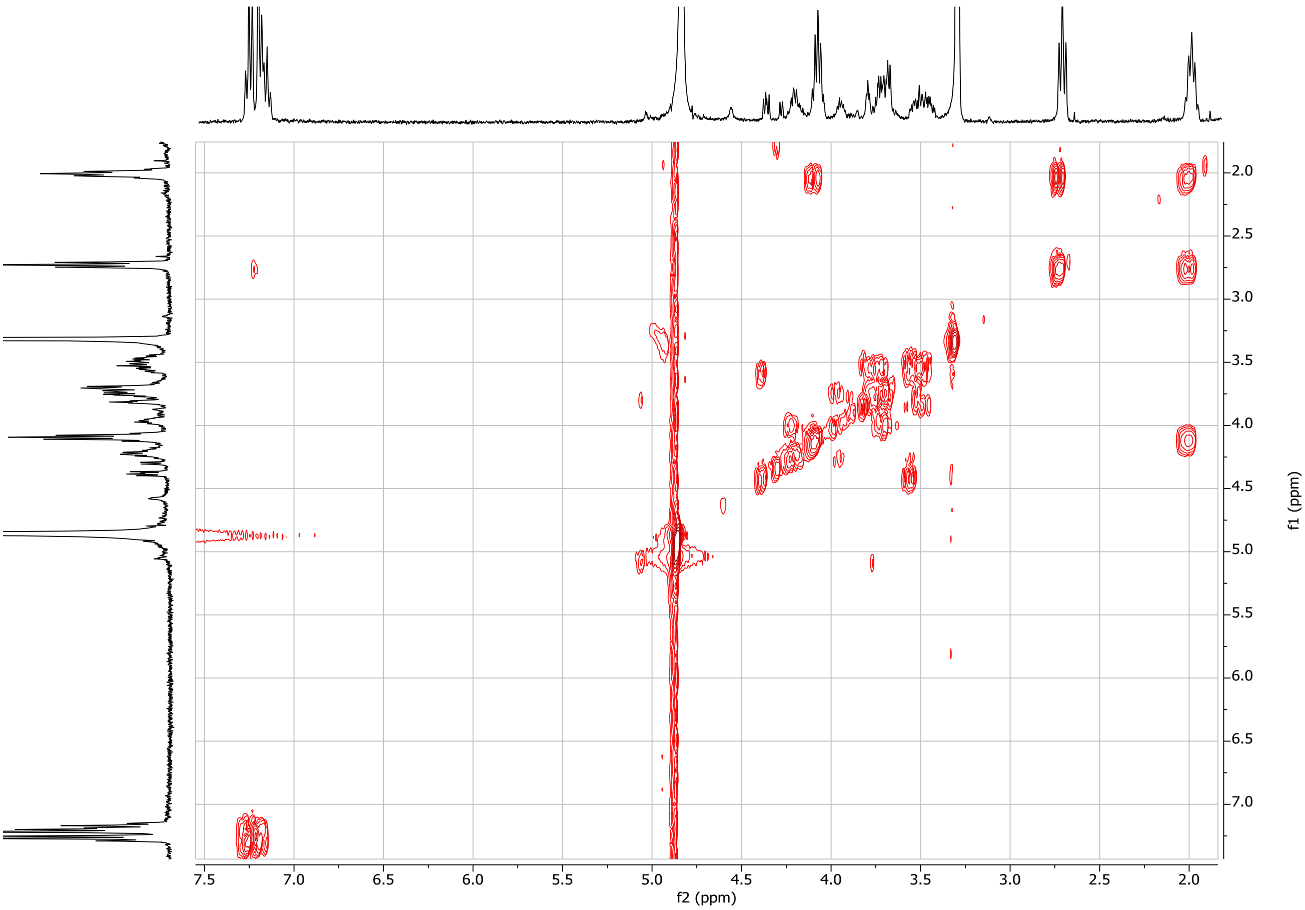

Figure S137 2D NMR COSY (MeOD) spectrum of **1f.**

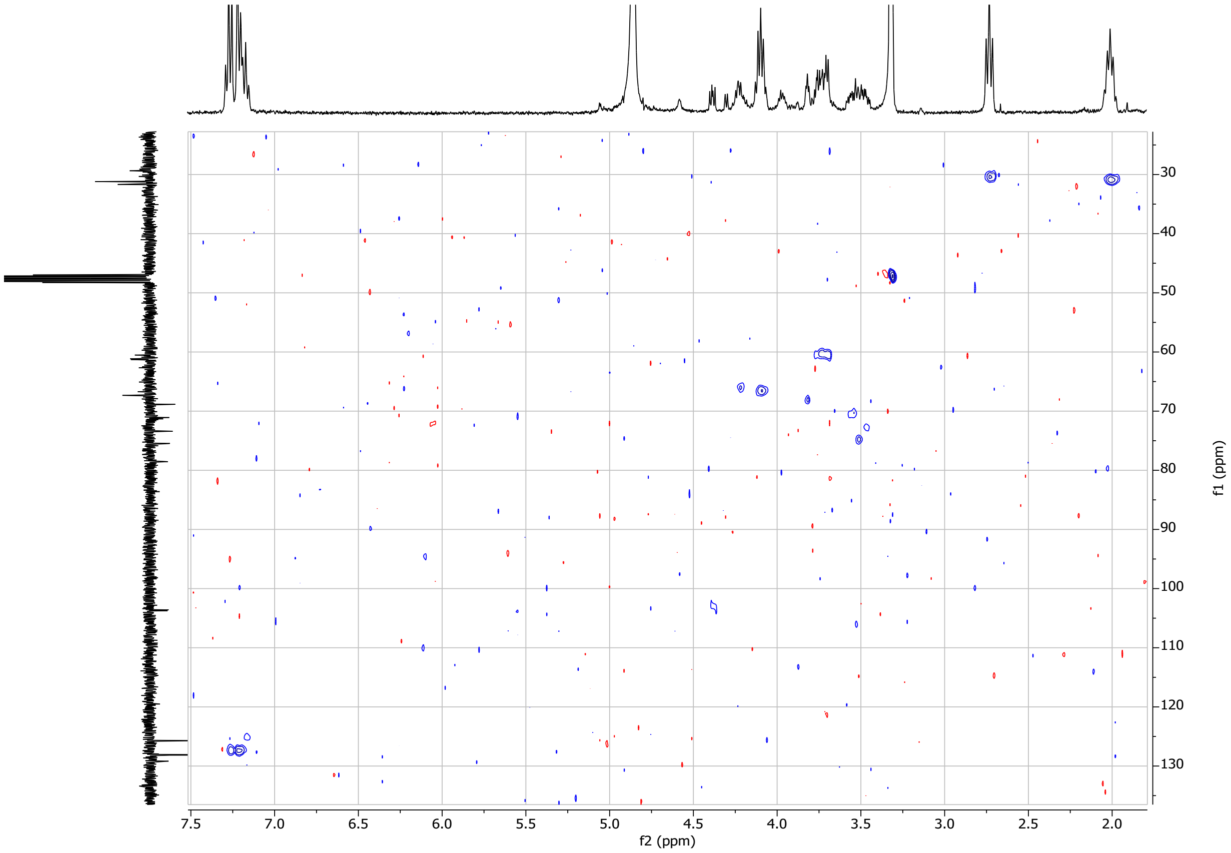

Figure S138 2D NMR HSQC (MeOD) spectrum of **1f.**

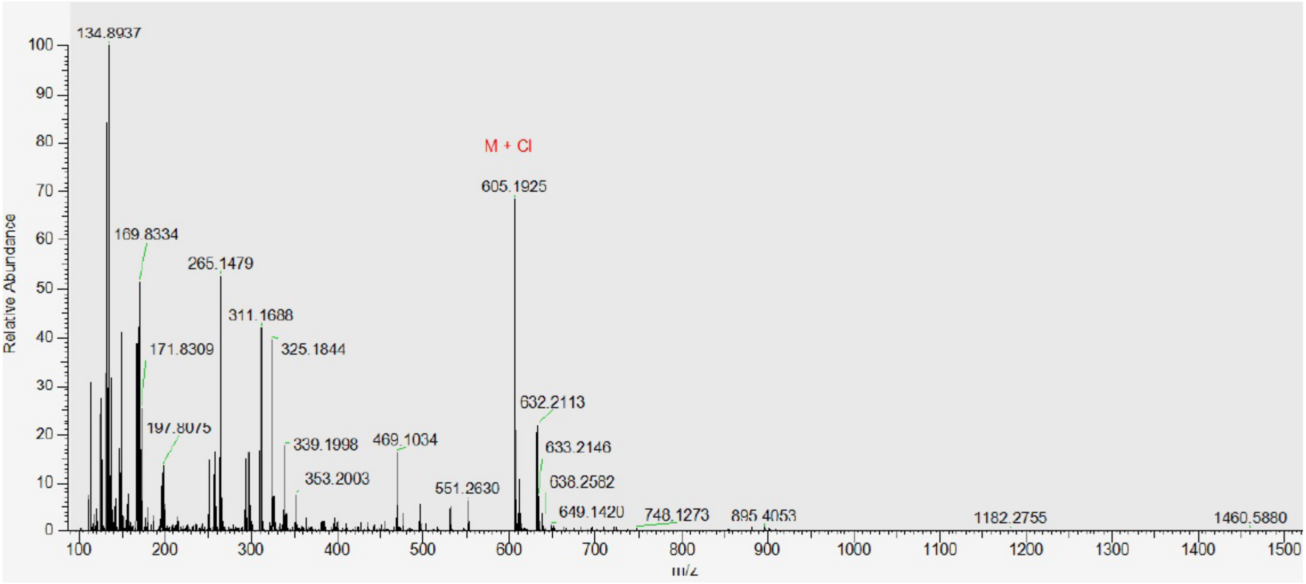

Figure S139 ESI-HRMS spectrum of **1f.**

Compound 1g

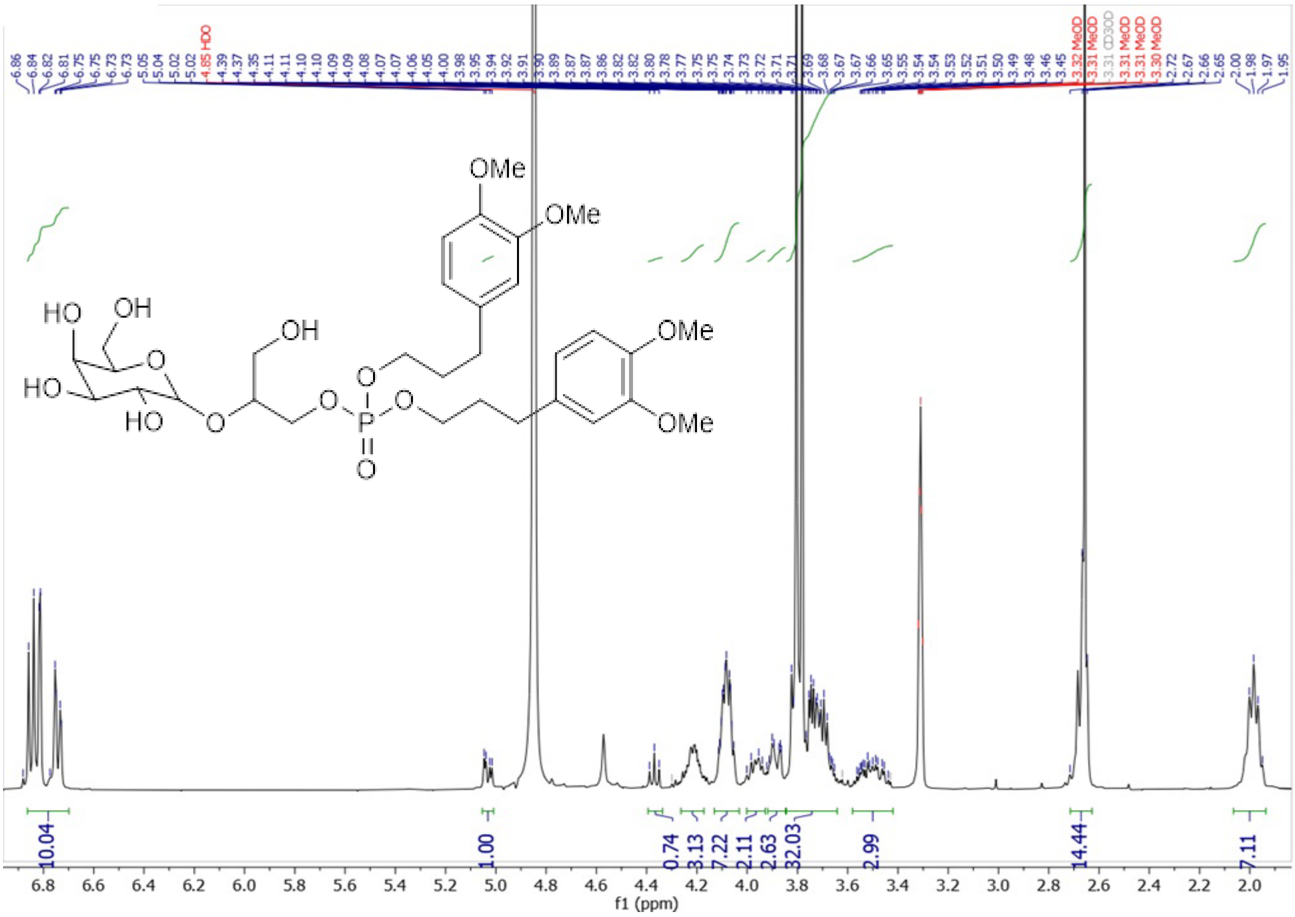

Figure S140 ^{1}H NMR (MeOD) spectrum of **1g.**

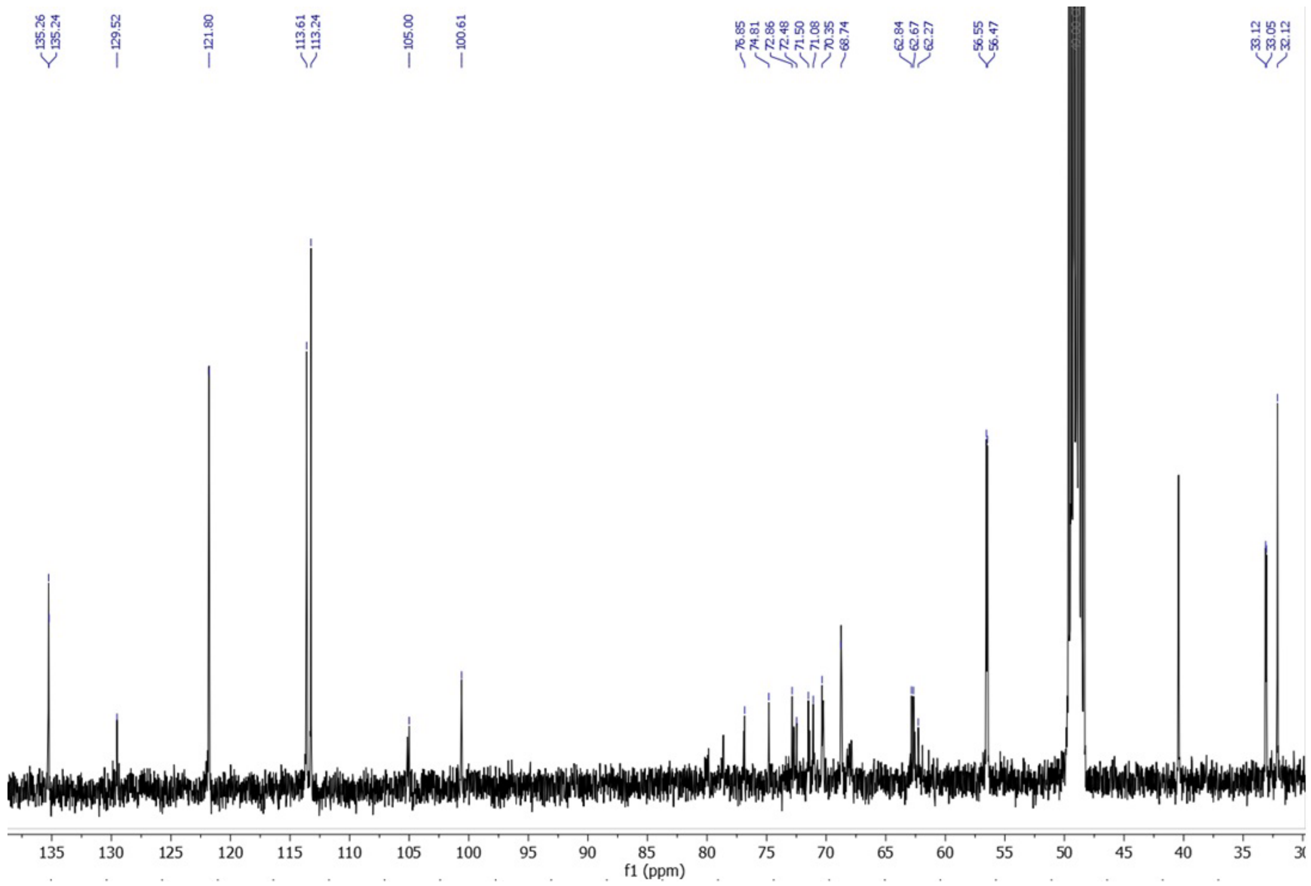

Figure S141 ^{13}C NMR (MeOD) spectrum of **1g.**

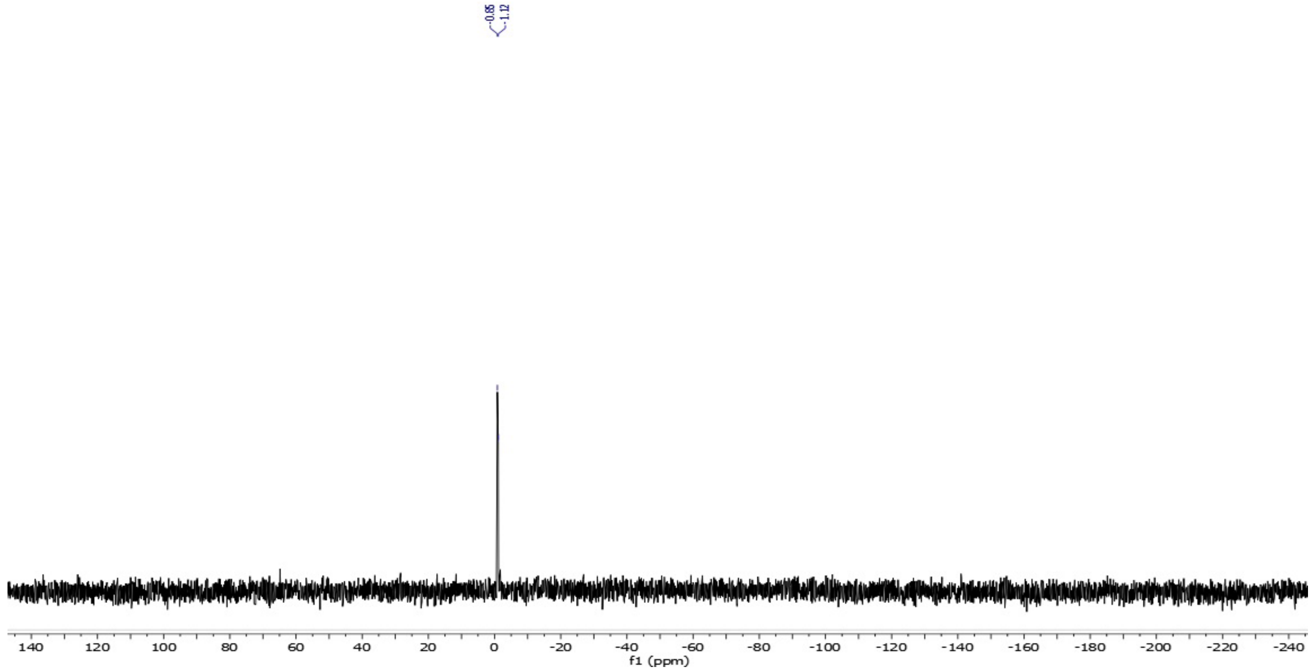

Figure S142 ^{31}P NMR (MeOD) spectrum of **1g.**

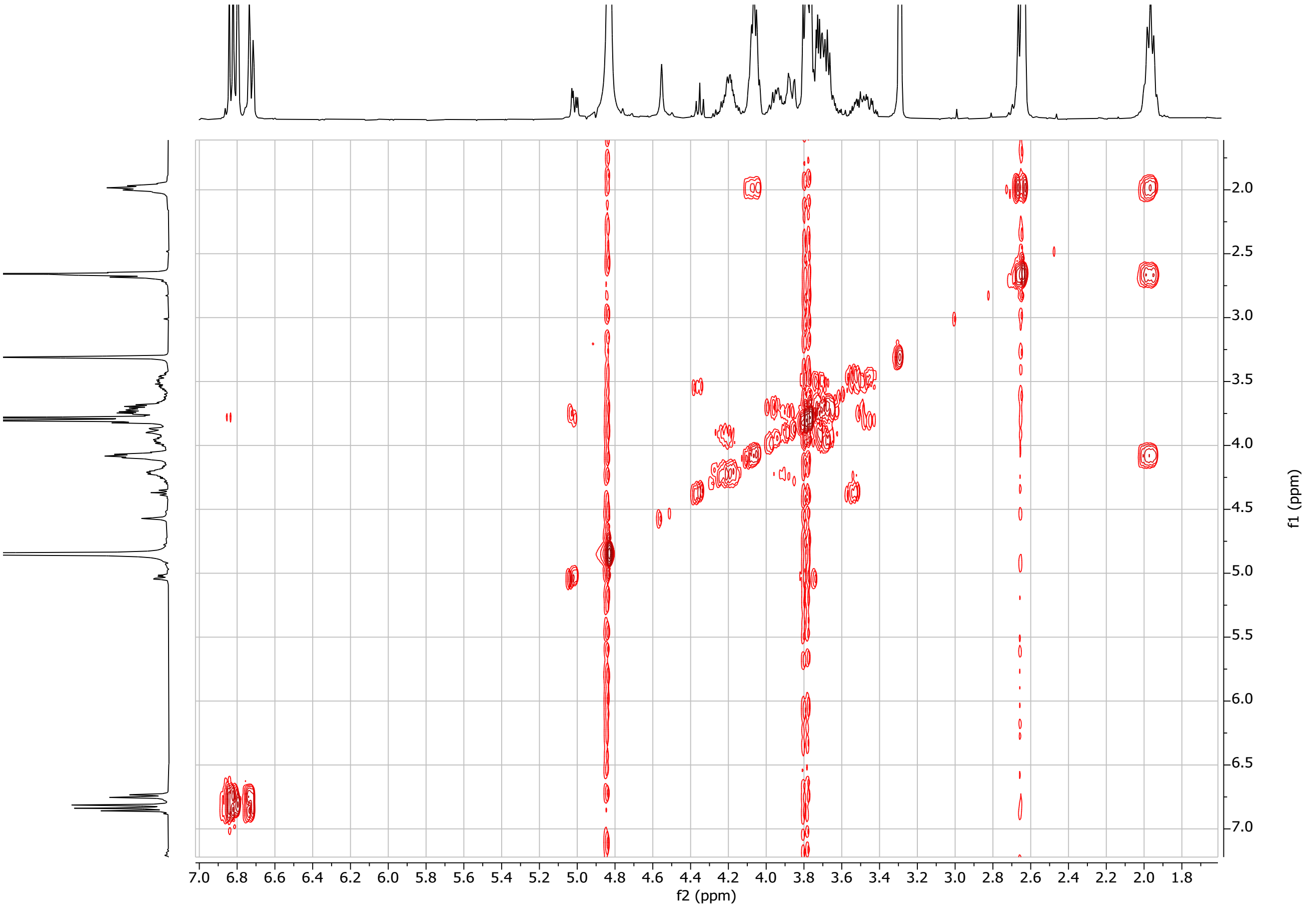

Figure S143 2D NMR COSY (MeOD) spectrum of **1g.**

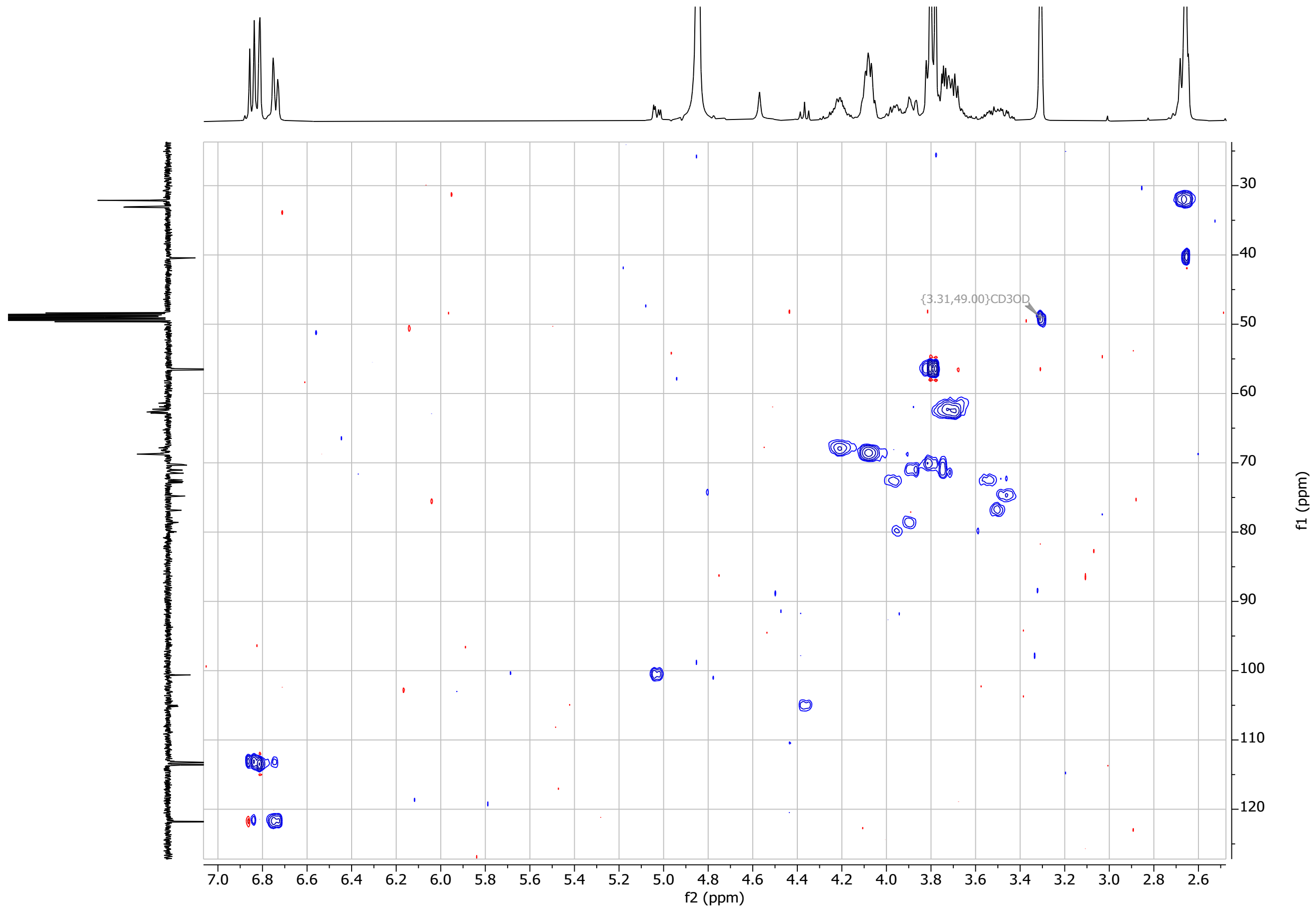

Figure S144 2D NMR HSQC (MeOD) spectrum of **1g.**

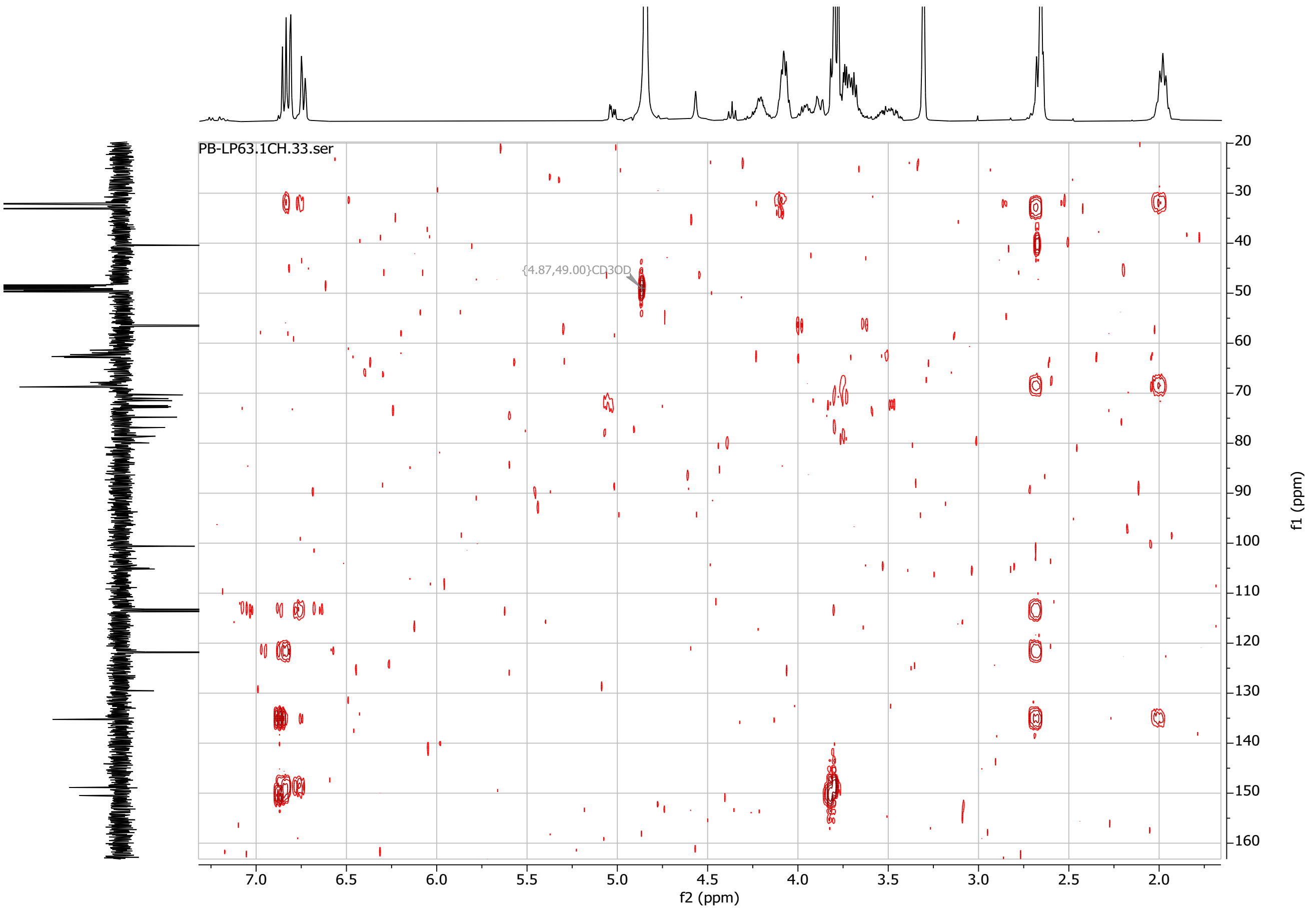

Figure S145 2D NMR HMBC (MeOD) spectrum of **1g**.

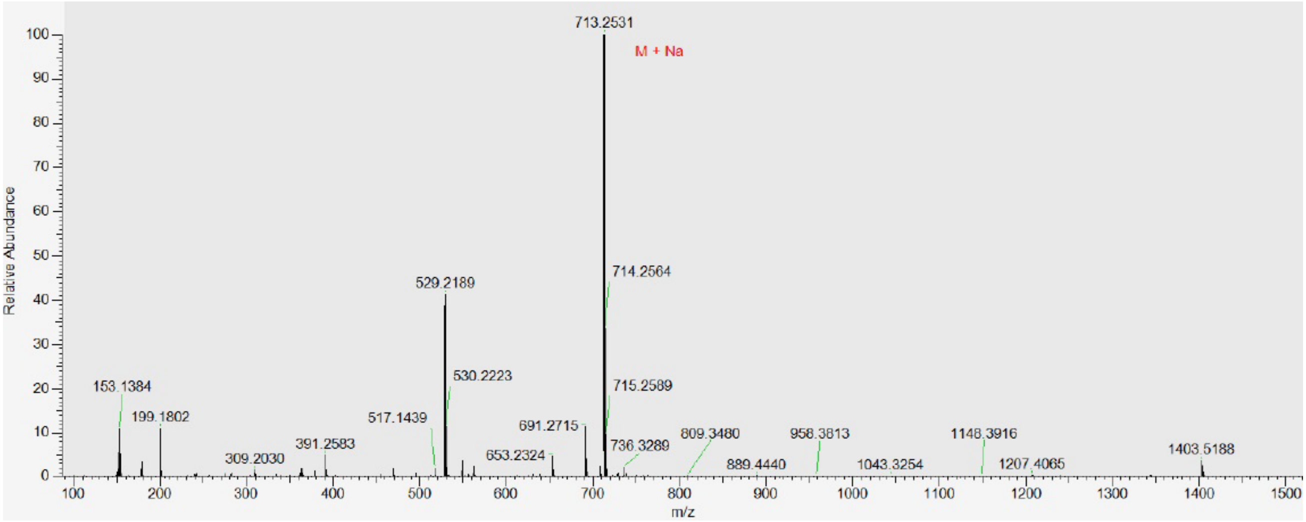

Figure S146 ESI-HRMS spectrum of **1g**.

Compound 1h

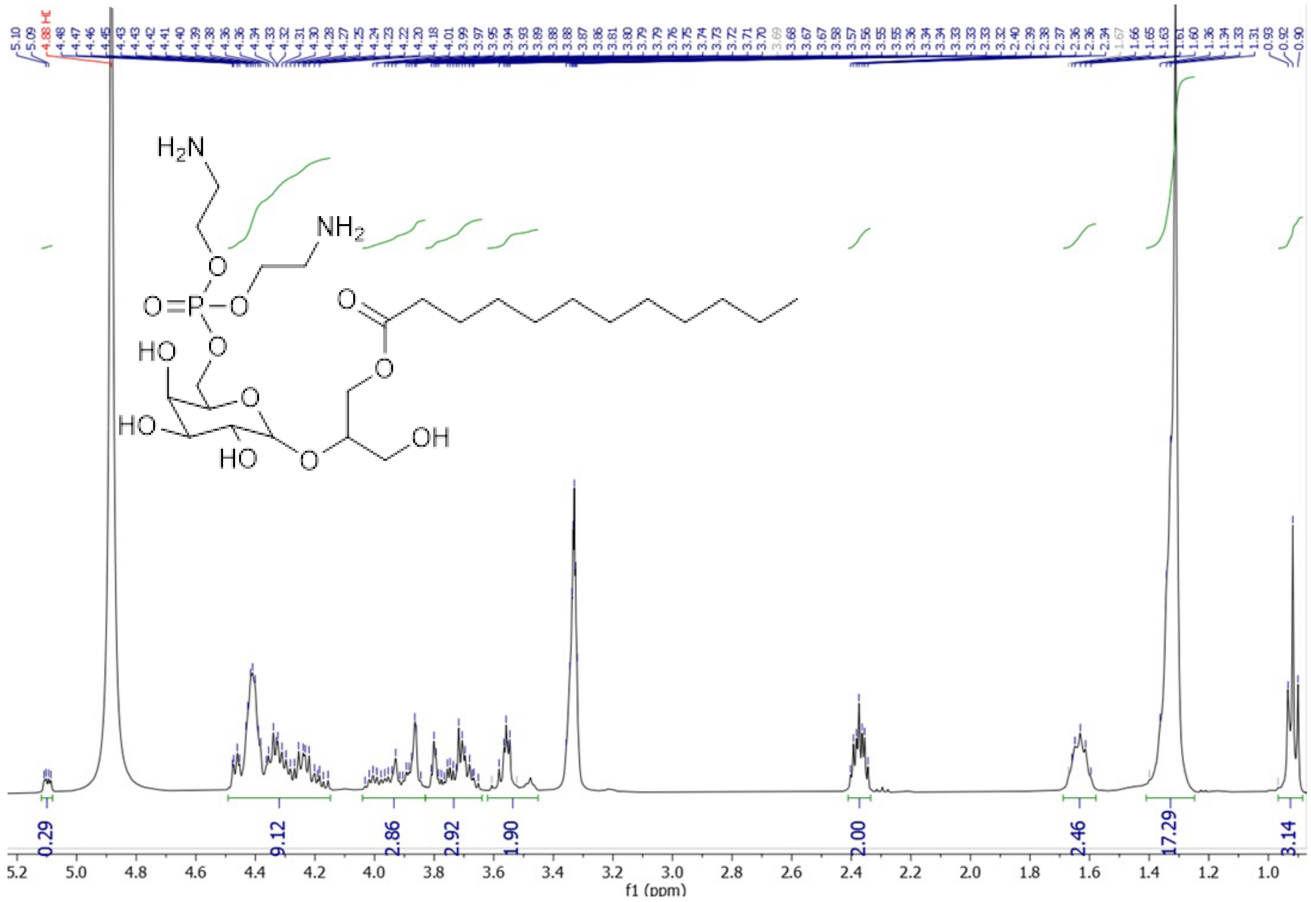

Figure S147 ^{1}H NMR (MeOD) spectrum of **1h.**

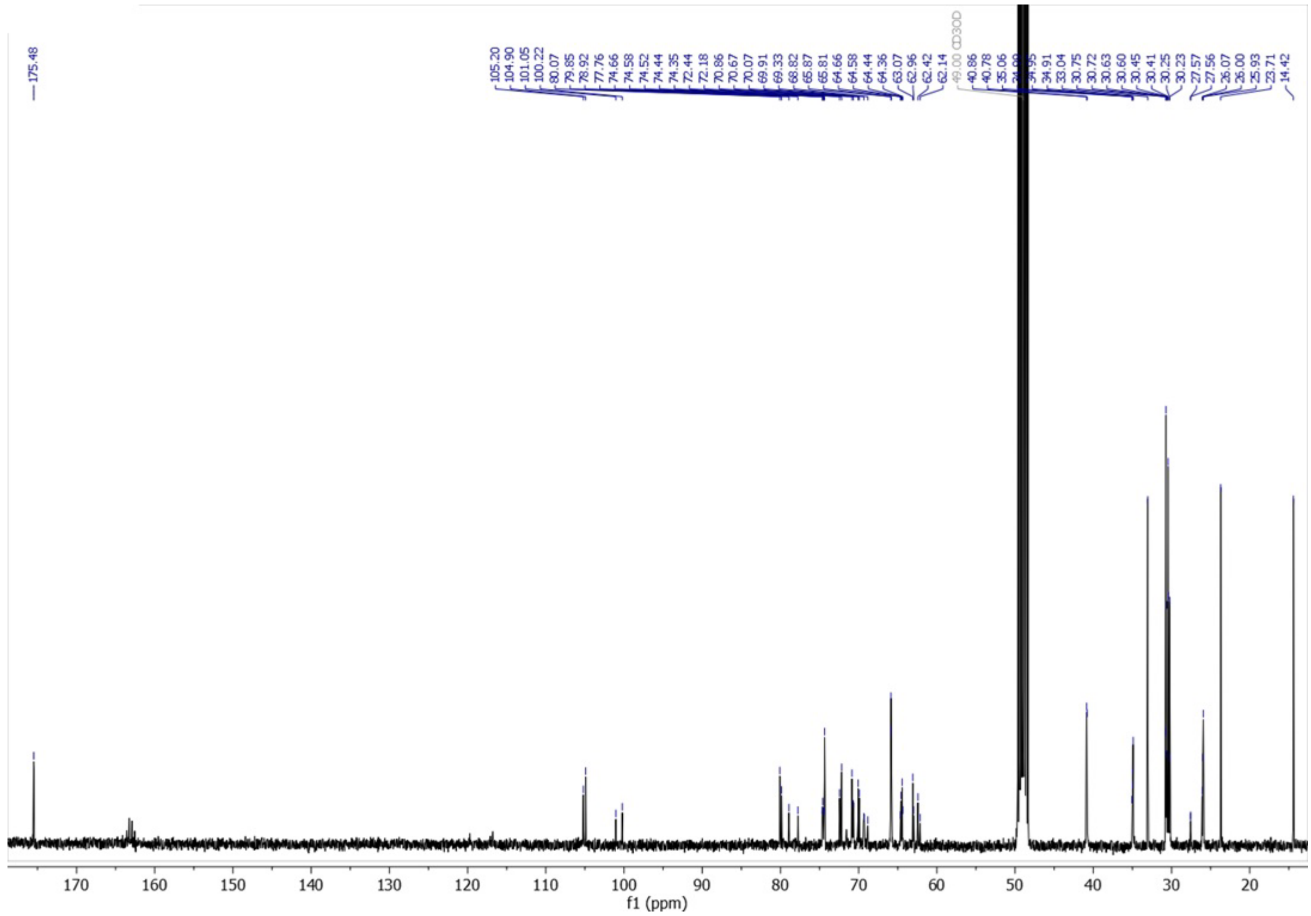

Figure S148 ^{13}C NMR (MeOD) spectrum of **1h.**

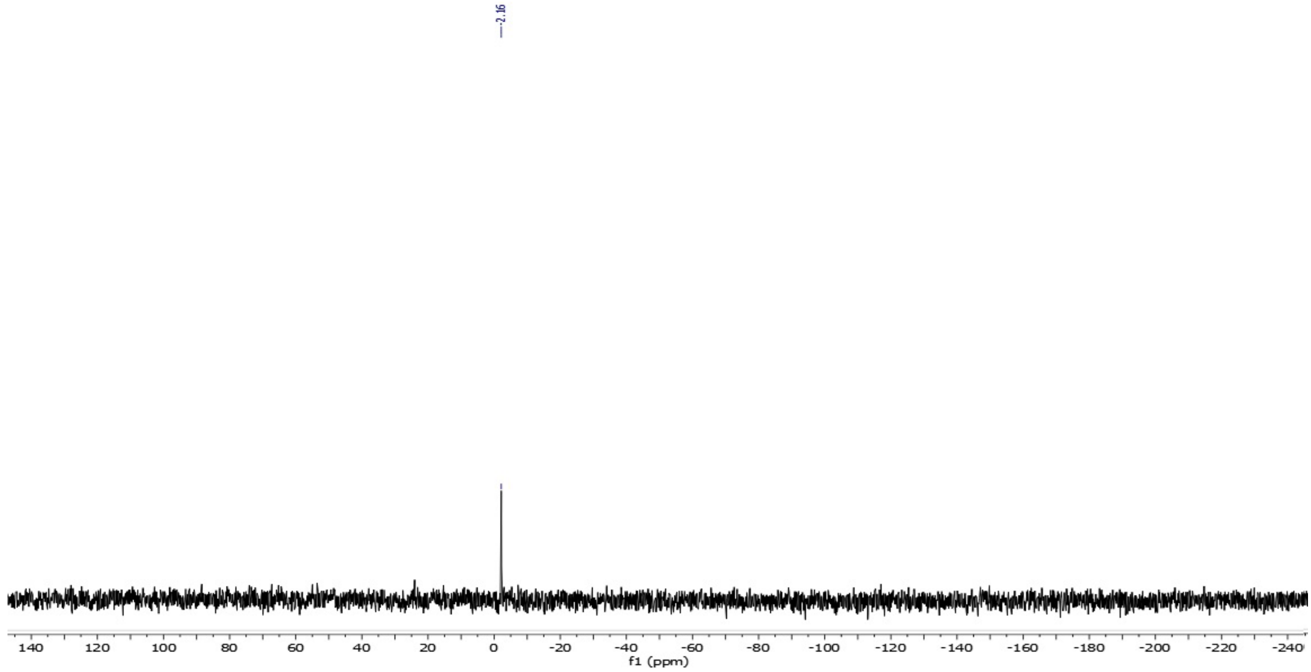

Figure S149 ^{31}P NMR (MeOD) spectrum of **1h.**

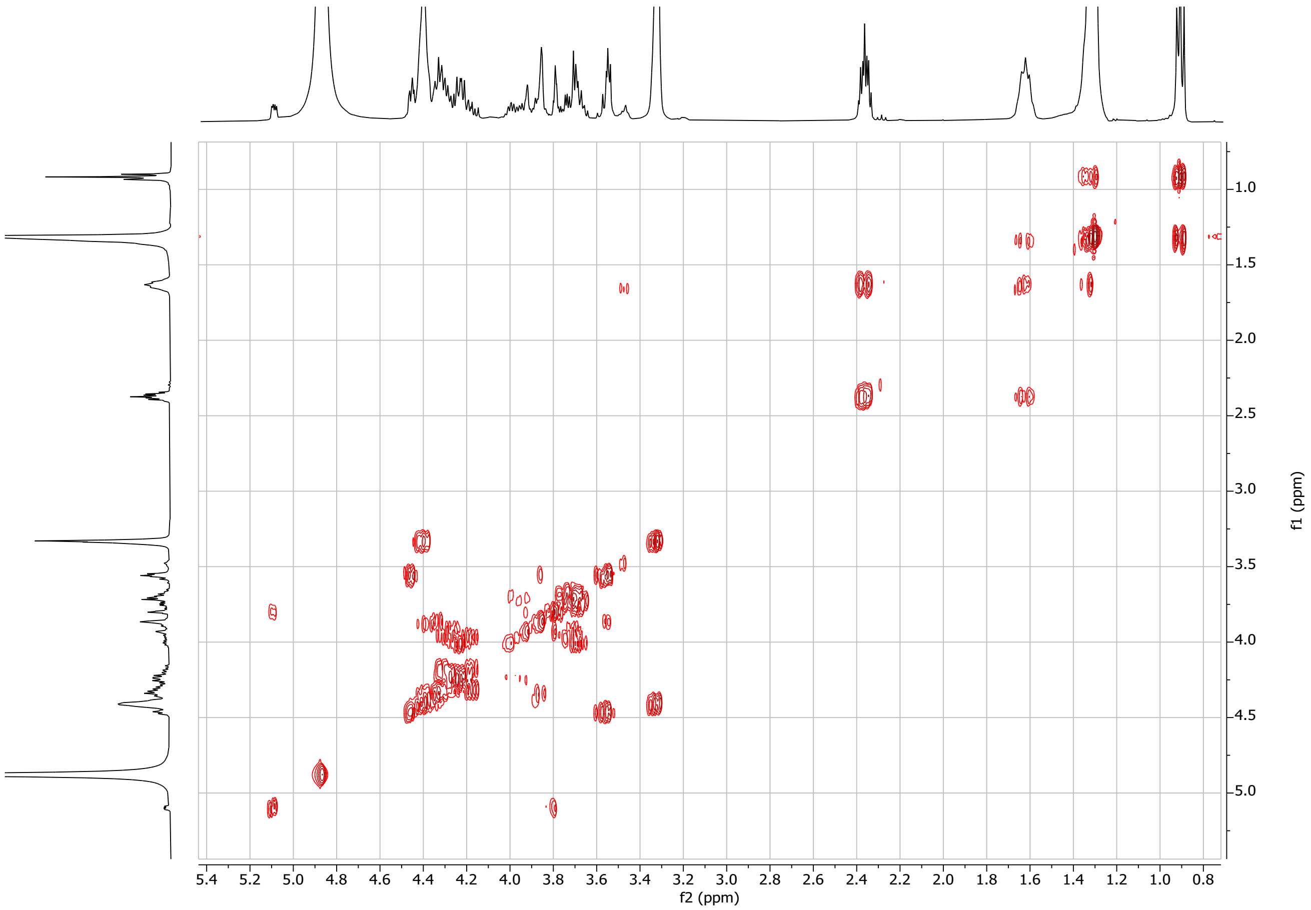

Figure S150 2D NMR COSY (MeOD) spectrum of **1h.**

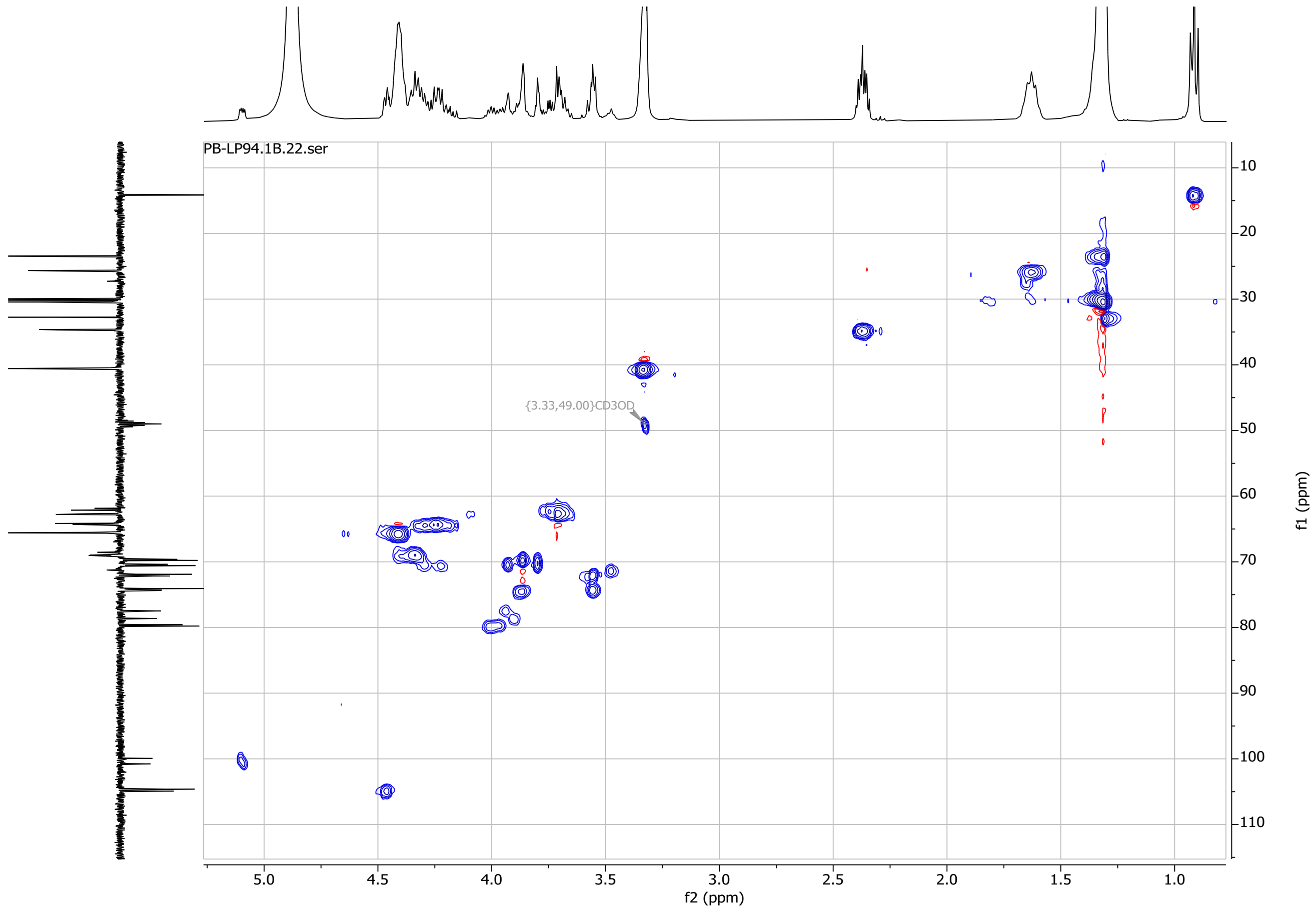

Figure S151 2D NMR HSQC (MeOD) spectrum of **1h.**

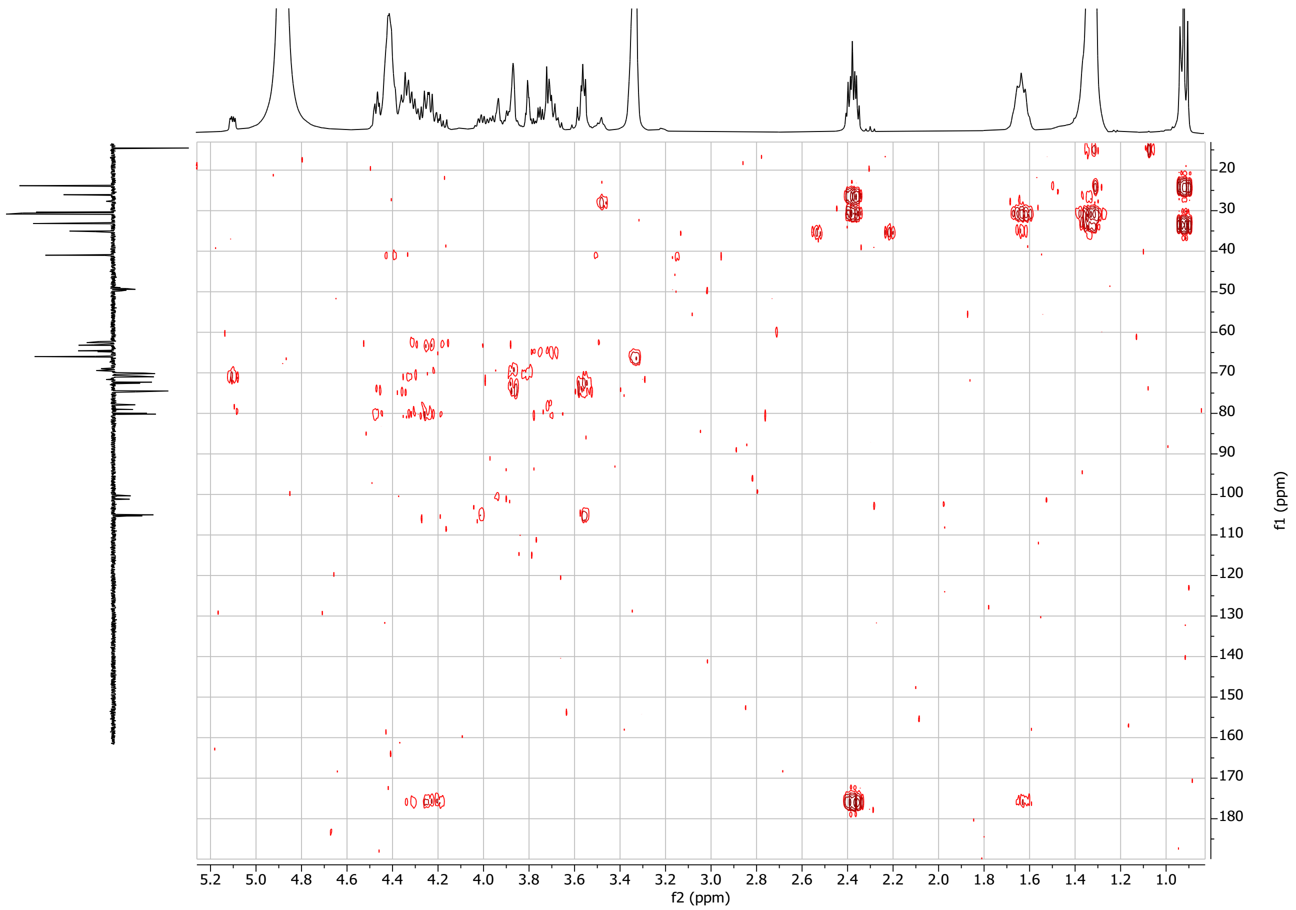

Figure S152 2D NMR HMBC (MeOD) spectrum of **1h.**

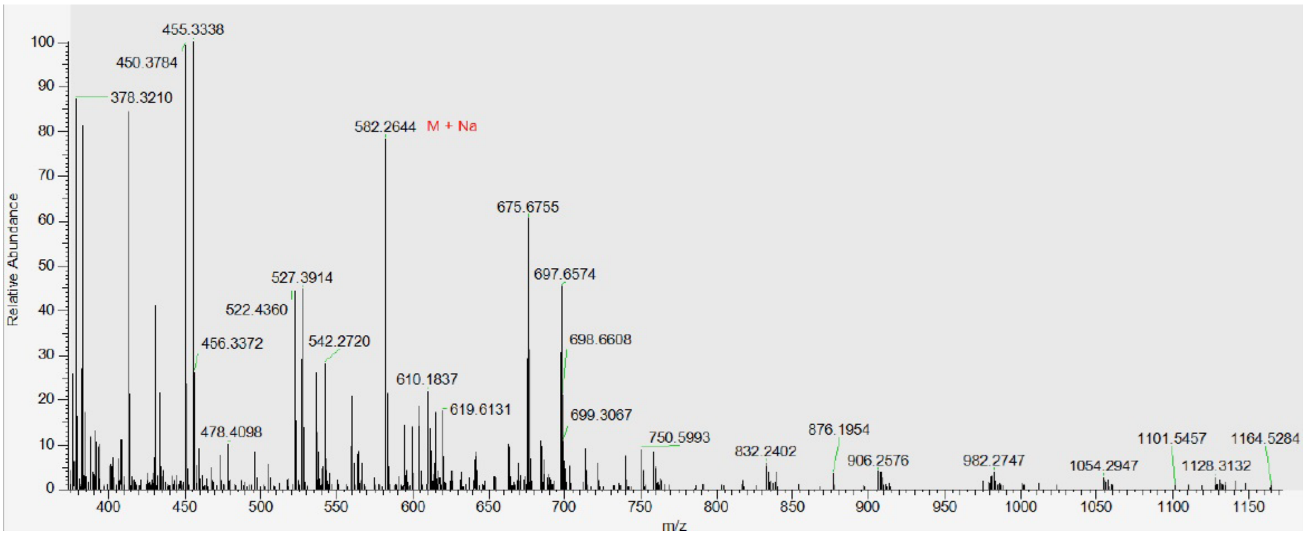

Figure S153 ESI-HRMS spectrum of **1h.**

3. Biological assays

3.1 General information for biological assays

The following reagents were purchased from Sigma-Aldrich Co. LLC (St. Louis, USA): phorbol-12-myristate-13-acetate (PMA), Triton X-100, diphenyleneiodonium chloride (DPI), *N*-acetyl-3,7-dihydroxyphenoxazine (Amplex Red), horseradish peroxidase (HRP), histopaque 1077, histopaque 1119, Dulbecco's Phosphate Buffer saline without calcium chloride and magnesium chloride (PBS), trypan blue solution 0.4%, dimethylsulphoxide (DMSO), trizma, D-(+)-glucose, and quercetin. 2-[6-(4-Aminophenoxy)-3-oxo-3H-xanthen-9-yl]benzoic acid (APF) was purchased from Invitrogen, Life Technologies Ltd. (Paisley, UK). Luminol was purchased from Fluka Chemie GmbH (Steinheim, Germany). Calcium chloride dihydrate and magnesium sulphate were purchased from Merck (Darmstadt, Germany). Potassium chloride was purchased from Pronalab (Abrunheira, Portugal). Sodium chloride from Honeywell Riedel-de Haën (Hanover, Germany). The FITC Annexin V Apoptosis Detection Kit was obtained from BD Pharmingen™ (Franklin Lakes, USA). All chemicals and solvents used in the synthesis procedures were obtained from commercial sources and used as received. *Tris*-glucose buffer (pH =7.4): [$CaCl_2$] = 1.26 mM, [KCl] = 5.37 mM, [$MgSO_4$] = 0.81 mM, [NaCl] = 140 mM, [Trizma] = 25 mM, [D-glucose] = 5.5 mM.

3.2 Isolation of human neutrophils

All patient-related procedures and protocols were performed in accordance with the Declaration of Helsinki and approved by the Ethics Committee of the *Centro Hospitalar do Porto, Portugal*. After informed consent, venous blood was collected from healthy human volunteers by antecubital venipuncture into K_3EDTA vacuum tubes, and the isolation of human neutrophils was performed as previously reported by the gradient density centrifugation method [16]. Approximately 3 mL of Histopaque 1077 was carefully added on top of approximately 3 mL of Histopaque 1119 in a centrifuge tube. The blood was slowly added to the top layer and centrifuged at 900g for 30 minutes at 20 °C. For the isolation of neutrophils, the granulocyte layer was collected, and the rest was discarded. PBS was added, and the tube was centrifuged at 850g for 5 minutes at 4 °C. The supernatant was discarded, the pellet was resuspended in 1.25 mL of PBS, and 4 mL of water was added to lyse red blood cells. The tube was homogenised and kept at room temperature for approximately 3 minutes, after which 2.2 mL of 3% NaCl was added to re-establish medium isotonicity. The cell suspension was centrifuged at 850g for 5 minutes at 4 °C. The supernatant was discarded, and the cells were suspended in *Tris*-glucose buffer and kept on ice under soft shaking. The trypan blue exclusion method was used to assess cell viability and determine cell yield, using a Neubauer chamber and an optical microscope. Cell density was further adjusted with *Tris*-glucose buffer according to the requirements of the performed assays.

3.3 Assessment of neutrophils' apoptosis versus necrosis

The effect of the compounds under study on neutrophil viability was evaluated using flow cytometry. This assessment involved staining with Annexin V (AV) and propidium iodide (PI) from the BD Pharmingen™ FITC Annexin V Apoptosis Detection Kit, following the manufacturer's protocol. Neutrophils (1 x10^6 cells/mL) were incubated in a 24-well plate in the dark at 37 °C for 45 minutes with the positive control (Triton X-100, 0.025%) or the tested compounds (in DMSO, 5% in the well) under various concentrations (up to 100 μM). Afterwards, the content of each well was transferred to a conical microtube and centrifuged at 400g, at 20 °C, for 5 minutes. The neutrophils' pellets were resuspended in PBS and centrifuged again under the previous conditions. The supernatant was removed, and the solution of Annexin V and PI from the kit was added, resuspending the pellet. The neutrophils were incubated in the dark at room temperature for 15 minutes and then diluted (dilution factor = 6) with the diluted binding buffer. The measurements were performed in an Accuri C6 flow cytometer (BD, Becton, Dickinson and Company, Franklin Lakes, NJ, USA), and the results were treated with BD Accuri™ C6 software. In the flow cytometer, the fluorescence signal of at least 10,000 neutrophils per sample was collected in logarithmic mode and followed in channels 1 and 3. A polygon gate, corresponding to the neutrophil's population, was drawn according to the light-scattering properties of neutrophils in a forward vs scatter plot. Thus, debris and other blood cells were unaccounted for in the samples. The green fluorescence corresponding to Annexin V was monitored in channel 1 and plotted as a histogram of FL1 staining, whereas the fluorescence corresponding to PI was monitored in channel 3 and plotted in the same histogram as FL3 staining. Neutrophils' cell death was expressed as a relative percentage of viable, apoptotic, late apoptotic, and necrotic cells compared to a negative control (untreated cells).

3.4 Evaluation of neutrophils' oxidative burst

3.4.1 Oxidation of luminol

Luminol is easily oxidized by reactive species produced during neutrophils' oxidative burst, resulting in a chemiluminescent signal [17]. The effect of the compounds under study on the modulation of neutrophil oxidative burst was determined after stimulation with PMA, a known neutrophil activator, because its effect is closely analogous to that obtained during the phagocytic process [18]. Following a previously reported method [19], in a 96-well plate, neutrophils were incubated in the dark at 37 °C for 5 minutes with luminol (500 μM) and the tested compounds (10 - 100 μM) or DMSO (final concentration 4%). Quercetin, a flavonoid known for its anti-inflammatory activity [16] (0.3 - 5.0 μM), and the inhibitor of NADPH oxidase, DPI (0.01 - 10 μM), were used as positive and negative controls, respectively. After incubation, PMA (160 nM) was added, and the chemiluminescence was measured in a microplate reader (Synergy HT, BIO-TEK Instruments, Inc., Winooski, VT, USA). Kinetic readings were initiated immediately after cell stimulation. Measurements were taken at the peak of the curve. This peak was observed at around 10 min. Effects are expressed as the inhibition percentage of luminol oxidation. The obtained results represent at least three independent experiments.

3.4.2 *Oxidation of APF*

APF is a non-fluorescent derivative of fluorescein, which is oxidized by HOCl, [20] $ONOO^-$, and $HO^\bullet$ [21] in a concentration-dependent manner. Its selectivity was tested using the myeloperoxidase (MPO) inhibitor, 4-aminobenzoic acid hydrazide (ABAH). The addition of ABAH to human neutrophils, stimulated with PMA, decreased the APF-dependent fluorescence signal to the level of the control assay, ruling out the involvement of $HO^\bullet$ and $ONOO^-$ [22]. The previously established methodology for monitoring oxidative burst modulation was adapted [19]. In a 96-well plate, neutrophils (2×10^6 cells/mL) were incubated in the dark at 37 °C for 5 minutes with APF (2 μM) and the tested compounds (10 - 100 μM) or DMSO (4%). Quercetin (0.3 - 5.0 μM) and DPI (0.01 - 10 μM) were used as positive and negative controls, respectively. After incubation, PMA (160 nM) was added, and the fluorescence (excitation at 495 nm and emission at 528 nm) was measured in a microplate reader (Synergy HT, BIO-TEK Instruments, Inc., Winooski, VT, USA). Kinetic readings were initiated immediately after the addition of APF. Obtained values correspond to the slope measured between 5 and 15 minutes. Effects are expressed as the percentage inhibition of APF oxidation. The obtained results represent at least three independent experiments.

3.4.3 *Oxidation of Amplex Red*

Amplex Red is a highly sensitive and chemically stable fluorescent probe for the extracellular detection of H_2O_2 [16]. The methodology used previously for monitoring the modulation of the oxidative burst was adapted [19]. In a 96-well plate, neutrophils (2×10^6 cells/mL) were incubated in the dark at 37 °C for 5 minutes with Amplex Red (25 μM), horseradish peroxidase (0.25 U/mL), and the compounds under study (10 - 100 μM) or DMSO (4%). Quercetin (0.3 - 5.0 μM) and DPI (0.01 - 10 μM) were used as positive controls for inhibition. After incubation, PMA (160 nM) was added, and the fluorescence (excitation at 530 nm and emission at 590 nm) was measured in a microplate reader (Synergy HT, BioTek Instruments, Inc., Winooski, VT, USA). Obtained values correspond to the slope measured between 5 and 10 minutes. Effects are expressed as the percentage inhibition of Amplex Red oxidation. The obtained results represent at least three independent experiments.

3.5 *Statistical Analysis*

GraphPad Prism 6 software was used to plot the inhibition percentage versus compound concentration curves, enabling the determination of the concentration required to achieve 50% inhibition (IC_{50}). GraphPad Prism 6 software was also used to perform all statistical analyses. Statistical comparison among the most active 2-SC was estimated by applying the one-way analysis (ANOVA), followed by the Bonferroni's multiple comparisons test. In all cases, p-values < 0.05 were considered statistically significant. The results are expressed as mean ± standard error of the mean (SEM).

3.6 *Cytotoxicity of* ***1b*** *and* ***1h***

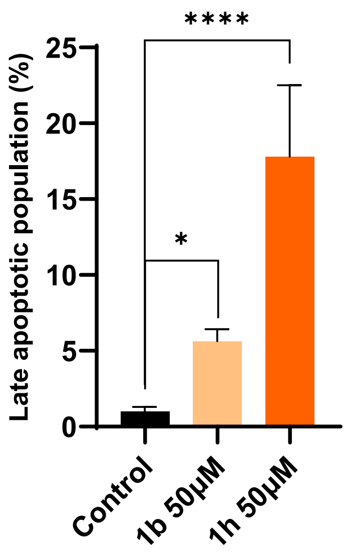

Figure S154 Percentages of late apoptotic populations induced by compounds 1b and 1h. *$p < 0.05$; ****$p < 0.0001$.

3.7 *IC*$_{50}$ *of* ***1e***

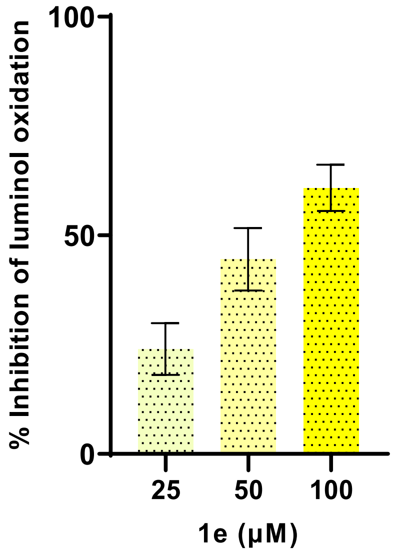

Figure S155 Percentage inhibition of luminol oxidation by compound **1e** at different concentrations.

4. References

[1] D.B.G. Williams, M. Lawton, Drying of Organic Solvents: Quantitative Evaluation of the Efficiency of Several Desiccants, J. Org. Chem. 75 (2010) 8351–8354. https://doi.org/10.1021/jo101589h.

[2] E. Stahl, M.R.F. Ashworth, Thin-Layer Chromatography, 2nd ed, Springer Berlin Heidelberg, Berlin, Heidelberg, 1969. https://doi.org/10.1007/978-3-642-88488-7.

[3] S.R. Vidadala, S.A. Thadke, S. Hotha, S. Kashyap, Synthesis of thioglycosides from propargyl glycosides exploiting alkynophilic gold catalyst, J. Carbohydr. Chem. 31 (2012) 241–251. https://doi.org/10.1080/07328303.2011.652789.

[4] A. Behera, D. Rai, D. Kushwaha, S.S. Kulkarni, Total Synthesis of Trisaccharide Repeating Unit of O - Specific Polysaccharide of Pseudomonas fluorescens BIM B-582, Org. Lett. 20 (2018) 5956–5959. https://doi.org/10.1021/acs.orglett.8b02669.

[5] J. López-Prados, F. Cuevas, N.-C. Reichardt, J.-L. de Paz, E.Q. Morales, M. Martín-Lomas, Design and synthesis of inositolphosphoglycan putative insulin mediators, Org. Biomol. Chem. 3 (2005) 764–786. https://doi.org/10.1039/B418041K.

[6] B. Herradón, S. Cueto, A. Morcuende, S. Valverde, Regio- and enantioselective esterifications of polyoxygenated compounds catalyzed by lipases, Tetrahedron: Asymmetry 4 (1993) 845–864. https://doi.org/10.1016/S0957-4166(00)80123-8.

[7] A.B.C. Simas, A.A.T. da Silva, T.J. dos Santos Filho, P.T.W. Barroso, Direct selective and controlled protection of multiple hydroxyl groups in polyols via iterative regeneration of stannylene acetals, Tetrahedron Lett. 50 (2009) 2744–2746. https://doi.org/10.1016/j.tetlet.2009.03.114.

[8] N.A.M. Ligthart, M.A.R. de Geus, M.A.T. van de Plassche, D. Torres García, M.M.E. Isendoorn, L. Reinalda, D. Ofman, T. van Leeuwen, S.I. van Kasteren, A Lysosome-Targeted Tetrazine for Organelle-Specific Click-to-Release Chemistry in Antigen Presenting Cells, J. Am. Chem. Soc. 145 (2023) 12630–12640. https://doi.org/10.1021/jacs.3c02139.

[9] J.M. Jacobson, P.I. Kitov, D.R. Bundle, The synthesis of a multivalent heterobifunctional ligand for specific interaction with Shiga toxin 2 produced by E. coli O157:H7, Carbohydr. Res. 378 (2013) 4–14. https://doi.org/10.1016/j.carres.2013.05.010.

[10] M. Häner, C. Herrstedt Hammelev, C.M. Pedersen, Conformational Distortion Using a Molecular Lever: Synthesis and Conformational Studies of Galactoside Derivatives, European J. Org. Chem. 2018 (2018) 5532–5537. https://doi.org/10.1002/ejoc.201801094.

[11] J. Lv, T. Luo, D. Zou, H. Dong, Using DMF as Both a Catalyst and Cosolvent for the Regioselective Silylation of Polyols and Diols, European J. Org. Chem. 2019 (2019) 6383–6395. https://doi.org/10.1002/ejoc.201901195.

[12] D. Pan, J. Sun, H. Jin, Y. Li, L. Li, Y. Wu, L. Zhang, Z. Yang, Supramolecular assemblies of novel aminonucleoside phospholipids and their bonding to nucleic acids, Chem. Commun. 51 (2015) 469–472. https://doi.org/10.1039/C4CC07538B.

[13] B. Bernardoni, L. Di Terlizzi, E.M. Galathri, C.G. Kokotos, M. Fagnoni, S. Protti, Visible photons for the regioselective nucleophilic ring opening of epoxides, Green Chem. 26 (2024) 9833–9839. https://doi.org/10.1039/D4GC02612H.

[14] D.I. Batovska, S. Tsubota, Y. Kato, Y. Asano, M. Ubukata, Lipase-mediated desymmetrization of glycerol with aromatic and aliphatic anhydrides, Tetrahedron: Asymmetry 15 (2004) 3551–3559. https://doi.org/10.1016/j.tetasy.2004.09.033.

[15] M. Sutter, W. Dayoub, E. Métay, Y. Raoul, M. Lemaire, 1-O-Alkyl (di)glycerol ethers synthesis from methyl esters and triglycerides by two pathways: catalytic reductive alkylation and transesterification/reduction, Green Chem. 15 (2013) 786. https://doi.org/10.1039/c3gc36907b.

[16] D. Ribeiro, M. Freitas, S.M. Tomé, A.M.S. Silva, G. Porto, E. Fernandes, Modulation of human neutrophils' oxidative burst by flavonoids, Eur. J. Med. Chem. 67 (2013) 280–292. https://doi.org/10.1016/j.ejmech.2013.06.019.

[17] R.C. Allen, L.D. Loose, Phagocytic activation of a luminol-dependent chemiluminescence in rabbit alveolar and peritoneal macrophages, Biochem. Biophys. Res. Commun. 69 (1976) 245–252. https://doi.org/10.1016/S0006-291X(76)80299-9.

[18] L. DeChatelet, P. Shirley, R.J. Johnston, Effect of phorbol myristate acetate on the oxidative metabolism of human polymorphonuclear leukocytes, Blood 47 (1976) 545–554. https://doi.org/10.1182/blood.V47.4.545.545.

[19] M. Freitas, V.M. Costa, D. Ribeiro, D. Couto, G. Porto, F. Carvalho, E. Fernandes, Acetaminophen prevents oxidative burst and delays apoptosis in human neutrophils, Toxicol. Lett. 219 (2013) 170–177. https://doi.org/10.1016/j.toxlet.2013.03.007.

[20] K. Sumitomo, N. Shishido, H. Aizawa, N. Hasebe, K. Kikuchi, M. Nakamura, Effects of MCI-186 upon neutrophil-derived active oxygens, Redox Rep. 12 (2007) 189–194. https://doi.org/10.1179/135100007X200317.

[21] K. Setsukinai, Y. Urano, K. Kakinuma, H.J. Majima, T. Nagano, Development of Novel Fluorescence Probes That Can Reliably Detect Reactive Oxygen Species and Distinguish Specific Species, J. Biol. Chem. 278 (2003) 3170–3175. https://doi.org/10.1074/jbc.M209264200.

[22] M. Freitas, J.L.F.C. Lima, E. Fernandes, Optical probes for detection and quantification of neutrophils' oxidative burst. A review, Anal. Chim. Acta 649 (2009) 8–23. https://doi.org/10.1016/j.aca.2009.06.063.
